# Analysis of plant leaf metabolites reveals no common response to insect herbivory by *Pieris rapae* in three related host-plant species

**DOI:** 10.1093/jxb/erv045

**Published:** 2015-02-24

**Authors:** A. C. Riach, M. V. L. Perera, H. V. Florance, S. D. Penfield, J. K. Hill

**Affiliations:** ^1^Department of Biology, University of York, York YO10 5DD, UK; ^2^Biosciences, College of Life and Environmental Sciences, University of Exeter, Exeter EX4 4QD, UK; ^3^John Innes Centre, Norwich Research Park, Norwich NR4 7UH, UK

**Keywords:** Brassicales, herbivory, induced metabolites, metabolic fingerprinting.

## Abstract

Studying the biochemical responses of different plant species to insect herbivory may help improve our understanding of the evolution of defensive metabolites found in host plants and their role in plant–herbivore interactions. Untargeted metabolic fingerprints measured as individual mass features were used to compare metabolite reactions in three Brassicales host-plant species (*Cleome spinosa*, *Brassica oleracea*, and *Lunaria annua*) to larval herbivore attack (*Pieris rapae*; Lepidoptera). Principal component analyses of metabolic fingerprints were able to distinguish among the three plant species and between uneaten control plants and plants that had been eaten. A large number of mass features (1186, 13% of mass features measured in control plants) were common to the three plant species. However, there were few similarities in the mass features that were induced (i.e. changed in abundance) following herbivory. Of the 87 and 68 induced mass features in *B. oleracea* and *C. spinosa*, respectively, there were only three that were induced in both plant species. By contrast, *L. annua* only had one mass feature induced by herbivory, and this was not induced in the other two plant species. The growth of the *P. rapae* larvae was poorer on the host plant *L. annua* than on *B. oleracea* and *C. spinosa*. The absence of common metabolites among the plants meant these induced responses could not be related to the performance of the herbivore. Thus, the response to herbivory by the same herbivore in these three host plants has evolved to be idiosyncratic in terms of the specific metabolites induced.

## Highlight

Herbivore attacks on three species of Brassicales result in different metabolic fingerprints. Furthermore, there is no overlap among the three plants in the individual metabolites that are induced by herbivory.

## Introduction

Plants contain thousands of metabolites, some of which have a defence function against herbivores. These metabolite defences can be either ‘constitutive’, with high levels of the metabolite maintained within the plant, or ‘induced’ where the metabolite is changed in abundance following herbivore attack (Bezemer and van Dam, 2005). The variation in herbivore growth rate observed on different plant species for polyphagous insects such as *Pieris rapae* L. (small white butterfly) (Hwang *et al.*, 2008) could be partially attributed to the metabolite composition of host plants.

Pierid butterfly larvae feed on a range of host plants (Braby and Trueman, 2006), many of which are within the family Brassicaceae (Asher *et al.*, 2001; Stevens, 2001 onwards; Beilstein *et al.*, 2008). Groups of structurally similar secondary metabolites tend to be common to plant species that are phylogenetically related (Wink, 2003). For example, the plant family Brassicaceae is characterized by glucosinolates (Fahey *et al.*, 2002), which are known to have defensive functions against herbivores (Ahuja *et al.*, 2010). Common metabolites among related plants would suggest that the related host plants of a polyphagous insect would employ some of the same specific metabolites for defence because they share an evolutionary history. On the other hand, the chemical defences of plants and the resistance of their herbivore attackers could be engaged in an evolutionary arms race (Mithofer and Boland, 2012). This means it is possible that any successful defence chemical originating in a common ancestor would be overcome by the insect during evolutionary time and lost, leaving few common chemical defences among related modern-day plant species.

There is little evidence to determine the extent to which plant metabolite defences have been shaped by a shared evolutionary history and the extent to which each plant species has evolved its own set of unique metabolites. This question has not been addressed before because of the vast number of metabolites in plants (Davies *et al.*, 2010) and the difficulty in simultaneously measuring a large proportion of those metabolites. However, metabolic fingerprinting, which is an untargeted, high-throughput method, can measure a large number of metabolites to gain a ‘snapshot’ of an organism’s metabolome (Fiehn, 2001; Overy *et al.*, 2005). Metabolic fingerprints have already been used to assess the effects of herbivory on plants. For example, the metabolic fingerprints of *Plantago lanceolata* differed in response to different stresses (including herbivory), even though no difference was observed using a targeted analysis (Sutter and Müller, 2011). An untargeted metabolomic method has also been used to investigate induced metabolites in *Zea mays* (Marti *et al.*, 2013) and *Arabidopdsis thaliana* (Kutyniok and Müller, 2012). However, none of these studies has considered herbivory of multiple host-plant species, and the numbers of induced metabolites that are shared or unique among host species have not been investigated. Such interspecific measurements could provide information on the evolution of metabolites in plants, for example whether there are particular defensive compounds that have been successful in defending plants against herbivory and which have been retained in many plant species over evolutionary time.

Although untargeted studies are lacking, targeted analyses have compared metabolites among different host plants, and in the Brassicaceae have focused on the glucosinolate group of metabolites (Rask *et al.*, 2000; Kroymann, 2011). Some studies have compared multiple host plants after insect attack to examine differences in the abundances of glucosinolates among plant species (Koritsas *et al.*, 1991; van Dam and Raaijmakers, 2006). From these studies, we know that glucosinolates vary qualitatively and quantitatively among host species. However, it is unknown if these differences in glucosinolates can be extrapolated to the rest of the metabolome.

In this study, we investigated three host-plant species that are eaten by *P. rapae* larvae in order to examine whether herbivore-induced metabolites (measured as mass features) are common to these host plants. By studying mass features that are induced by insect attack, we focused on those metabolites that were more likely to have a defensive role, although such induced mass features could also be associated with cell damage or the processes repairing cell damage. We also quantified insect growth to determine if the metabolite composition of the plants was reflected in herbivore performance. A search for induced compounds that are likely to be glucosinolates was also made in order to enable comparisons with previous work on induced metabolites in Brassicaceae. Quantifying the shared and unique induced mass features among the three plant species provides information on the evolution of defence metabolites in plants; for example, shared metabolites that are induced by multiple host plants may imply that these metabolites have a vital function in the *P. rapae*–plant interaction.

## Methods

### Insect and plant rearing

**Fig. 1.**
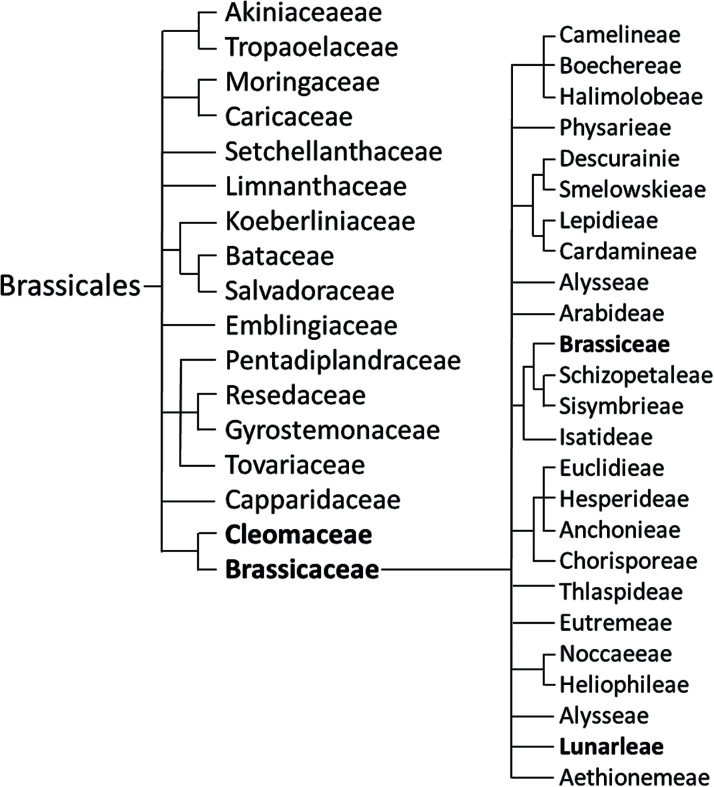
Taxonomic relationships of the three experimental plant species. Bold text indicates families and tribes of the three species. The species *C. spinosa* is placed in the family Cleomaceae, whereas the species *B. oleracea* is in the family Brassicaceae within the Brassiceae tribe. *L. annua* is also in the family Brassicaeae but in the tribe Lunarleae.

The three species of host plant that were investigated were *Brassica oleracea* L. cultivar ‘stonehead’ (cabbage), *Cleome spinosa* L. (spider flower) and *Lunaria annua* L. (honesty or money plant). These plants are in the order Brassicales, and provide two study species, *B. oleracea* and *L. annua*, that are in the same family (Brassicaceae) and one species *C. spinosa* in another family (Cleomaceae), which is more distantly related (Fig. 1). This enabled a comparison between plant relatedness and the number of shared metabolites. These three species have very different characteristics and vary with respect to the current knowledge of glucosinolate content (Table 1); the latter reflects the greater number of studies that have examined *B. oleracea* compared with *C. spinosa* and *L. annua*.

**Table 1.** Species characteristics and summary of the plant parts glucosinolates have been reported from in the three experimental plants

Characteristic	*B. oleracea*	*C. spinosa*	*L. annua*
Perennation	Perennial^*a*^	Annual^*b*^	Biennial^*c*^
Raunkiaer life form	Nanophanerophyte^*a*^	Therophyte^*a*^	Hemicrytophyte^*a*^
Herbacious/woody	Semi-woody^*a*^	Herbaceous^*b*^	Herbaceous^*c*^
Native status	Native or alien^*a*^	Alien casual^*d*^	Neophyte^*c*^
Glucosinolates	Glucosinolates recorded in seeds, leaves and roots^*e*,*f*^	Glucosinolates recorded in seeds and leaf surface^*g*,*h*^	Glucosinolates recorded in seeds^*i*^

^a^ Hill *et al.* (2004); ^b^ Stace, 2010; ^c^ Preston *et al.* (2002); ^d^ Clement and Foster (1994); ^e^ Pérez-Balibrea et al. (2011); ^f^ Kabouw *et al.* (2010); ^g^ Daxenbichler *et al.* (1991); ^h^ Griffiths *et al.* (2001); ^i^ Vaughn *et al.* (2006).

Plants were grown from seeds sourced from Groves Nurseries, Dorset, UK (*B. oleracea* ‘stonehead F1’) and Chiltern Seeds, Oxfordshire, UK (*C. spinosa* ‘Cherry Queen’ and *L. annua*). Seeds were planted in Levington F2+S seed and modular compost and plants were not fertilized. Seedlings were grown in a greenhouse in trays for 2 weeks and were then potted into 10cm pots and grown in temperature-controlled cabinets (Sanyo MLR 350) at a constant temperature (21 °C), a photoperiod of 16h light:8h dark and approximate light of 60 μmol m^2^ s^–1^. Pots were randomized among different growth cabinets until the plants had been growing for 7 weeks, at which point each plant was assigned as either control (*n*=10) or infested (*n*=10) and were grown in different cabinets to prevent plant volatile organic compounds affecting other plants.

The specialist *P. rapae* was chosen to invoke responses in the plants, as specialists are thought not to suppress induced plant responses (Ali and Agrawal, 2012) as generalists are able to do (Diezel *et al.*, 2007; Travers-Martin and Müller, 2007). *P. rapae* larvae were the offspring of four adult butterflies caught in York, UK (53°95′N, 1°08’W) in July. Female butterflies were kept in a greenhouse in 31×42cm keep nets, fed a honey solution soaked on cotton wool and given potted *B. oleracea* seedlings to oviposit on. Larvae laid over a period of 2 d were allowed to feed from the three host species until they reached the fourth or fifth instar (14 d after hatching) when they were transferred with a paintbrush to experimental plants. There was one larva per plant and 10 infested plants per plant species. Larvae were placed on a middle-aged leaf in all replicates. Larvae were confined to leaves using organza bags and hair crocodile clips to prevent the plant stem being crushed. Empty bags and clips were also placed on the equivalent leaves of control plants. Larvae were left on *B. oleracea* and *L. annua* plants for 67h but were taken off *C. spinosa* plants after 44h due to its smaller leaves and to ensure that some of the leaf was left for sampling. The remains of the eaten leaves in the infested treatment and the equivalent leaves in the control plants were cut at the stem and put in Eppendorf tubes immediately after removal of larvae, flash frozen and stored at –80 °C.

*P. rapae* larvae used to measure the performance of the insects on each of the plant species were all laid on the same day by two female butterflies. The leaf around the egg was cut and pinned to one of the three host-plant species in a greenhouse so that from hatching the larvae had experience of the host-plant species that it would develop on. After 7 d, the larvae were large enough to move with a paintbrush onto host plants in cabinets. There was one larva per plant and between 15 and 19 plants per species. These plants were grown under the same cabinet conditions as described above until larvae pupated. Pupae were kept in an individual plastic pot with the lid on loosely and with a small piece of damp paper towel until adult emergence. Adults were killed by freezing within 12h of emergence. Measurements of insect performance comprised growth rate calculated from the adult dry weight divided by the number of days from hatching to pupation. Growth rates of the larvae raised on different host plants were tested for statistical significance using one-way analysis of variance (ANOVA) and Tukey post-hoc tests.

### Metabolic fingerprint analyses

Stored leaf material was freeze dried for 16h and then ground for 2min at 20 Hz in a ball mill. Samples (10mg) were extracted twice with 400 μl of 80% methanol on ice, using umbelliferone as an internal standard. Samples were sonicated, vortexed, and the supernatant removed. The two supernatants were combined and filtered through a 0.4 μm (polyvinylidene difluoride) syringe filter.

Metabolite profiling of leaf material was performed using a QToF 6520 mass spectrometer coupled to a 1200 series Rapid Resolution LC system. Sample extract (5 µl) was loaded onto a Zorbax StableBond C18 1.8 µm, 2.1×100mm reverse-phase analytical column (liquid chromatography (LC)/mass spectrometry (MS) and column; Agilent Technologies, Palo Alto, USA). Features were detected in positive ionization mode. Mobile phase A comprised 5% acetonitrile with 0.1% formic acid in water, and mobile phase B was 95% acetonitrile with 0.1% formic acid in water. The following gradient was used: 0min, 0% B; 1min, 0% B; 5min, 20% B; 20min, 100% B; 30min, 100% B; 31min, 0% B; 7min, post time. The flow rate was 0.25ml min^–1^ and the column temperature was held at 35 °C for the duration. The source conditions for electrospray ionization were as follows: gas temperature was 325 °C with a drying gas flow rate of 9 l min^–1^ and a nebulizer pressure of 35 psig. The capillary voltage was 3.5kV. The skimmer and fragmentor voltages were 115 and 70V, respectively.

### Metabolic fingerprint data pre-processing

The Molecular Feature Extractor (MFE) in MassHunter software (Agilent Technologies) identified features (potential metabolites) from peaks produced by the LC/MS. MFE ‘recognizes’ signal patterns and accounts for these features representing isotopes and adducts (Soloman and Fischer, 2010). Features eluting within the first minute are contained within the ‘dead’ volume, and features after 27min are within the re-equilibration period; therefore, these features were excluded. The alignment of features across samples, filtering out noise, and missing value imputation (Hrydziuszko and Viant, 2012) were performed using an in-house alignment algorithm, ‘Kernel Feature Alignment’ (Perera, 2011). Features that were not detected in at least five of the 10 replicates were considered noise and excluded from the dataset, reducing the number of features detected from 36 432 to 11 649. The 11 649 mass features each had an associated retention time and *m*/*z* value (Supplementary Table S1 at *JXB* online). Principal component analysis (PCA) was performed on both datasets to ensure that the exclusion of features had no effect on the results. The number of mass features (11 649) was large and therefore it was likely that it included individual metabolites as well as fragments of metabolites. This is because the noise-filtering technique was not exhaustive in removing all fragments. In addition, this large number of mass features was partly explained by the greater number of metabolites found within three plant species compared with in a single species. Prior to data analysis, missing value imputation was applied in those cases where metabolites were detected in at least five but fewer than 10 replicates (Hrydziuszko and Viant, 2012). Data were log transformed and mean centred before multivariate statistical analyses.

### Statistical analysis

To determine if the three different plant species could be distinguished by their metabolic fingerprints, the data were analysed using PCA. To investigate differences between infested and control plants, each plant species was analysed separately by PCA, followed by *t*-tests of principal component (PC) scores. The lists of mass features recorded in each species of host plant were cross-referenced and Venn diagrams used to summarize the numbers of mass features that plant species had in common. This was carried out separately for mass features measured in control plants and infested plants.

To determine the metabolites induced by herbivory in the three plant species, it was established whether or not a mass feature differed in abundance between the infested and control plants. Data were tested for normality (Anderson Darling test) and equality of variance (Levene’s test) with data transformation if necessary (natural log, inverse or square root transformation) followed by *t*-tests, otherwise non-parametric Mann-Whitney *U* tests were used. These *P* values were corrected for false discovery rate by conversion to *q* values (Benjamini and Hochberg, 1995). For each mass feature, the difference in abundance was quantified using mean abundance in infested plants divided by the mean abundance in control plants, which indicated if the mass features had increased or decreased. This process produced lists of significantly increased and decreased mass features according to *q*<0.05, for each plant species. These were then cross-referenced to find common mass features induced (increased and decreased) by *P. rapae* herbivory. The numbers of common and unique mass features are summarized in Venn diagrams.

### Glucosinolates

Molecular weights for glucosinolate compounds (Fahey *et al.*, 2002) were obtained from databases for 99 out of 120 glucosinolates and a search made within the data sets for mass features with the same molecular weights (accurate to 2 decimal places). From this list of mass features, further validation of these putatively identified glucosinolates was achieved by ground proofing the relevant peaks in negative ion mode. Glucosinolates fragment to produce a sulfate product ion of particular mass, which, along with retention time, was used as an identifier for glucosinolates. A literature search determined if these glucosinolates had been recorded previously in any of the three plant species. To test for significant differences in the abundances of these glucosinolate-matching metabolites between control and infested plants, a *t*-test was used or a Mann–Whitney *U* test when assumptions of normality could not be met.

## Results

Thousands of mass features were measured by high-performance (HP)LC/MS from foliar samples (*n*=60) of three species of host plant both infested (eaten by *P. rapae*) and controls (not eaten) to obtain metabolic fingerprints. This allowed us to compare the effect of herbivory among the three plant species using PCA, and to quantify the number of induced mass features that were common among the host-plant species. Some mass features that matched the molecular weights of glucosinolates were evaluated to examine if any were induced by herbivory. The growth rates of insects on each host plant were measured to examine if there was any relationships between the metabolite reaction in plants and herbivore performance.

### Metabolic fingerprints are changed by herbivory in all three host plants

**Fig. 2.**
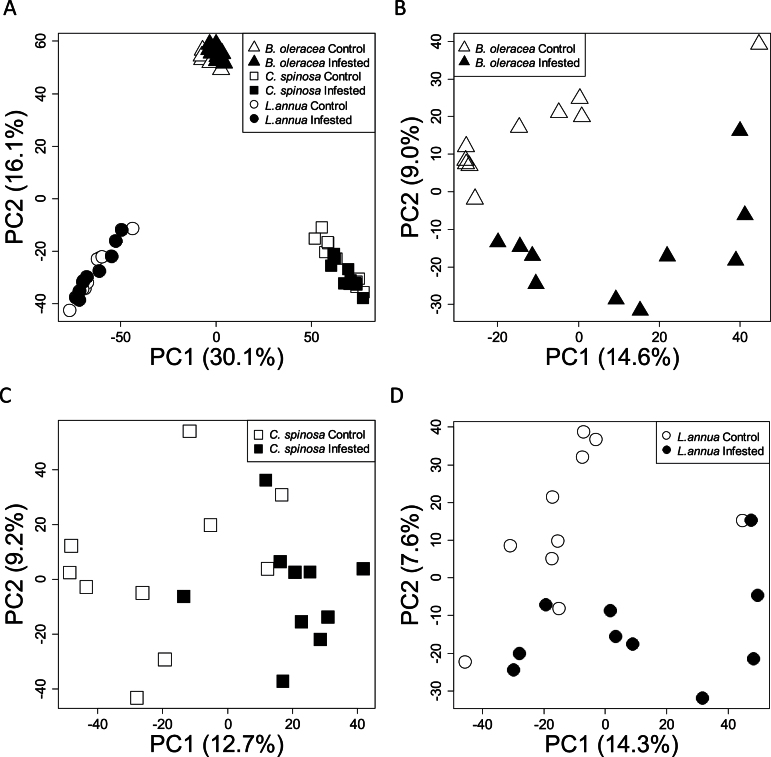
PC1 and -2 from PCA models fitted to metabolic fingerprints of all three host plants (A) and separately for *B. oleracea* (B) *C. spinosa* (C) and *L. annua* (D). The variation explained by each PC is shown in parentheses on the axes labels. In (A), different species clustered together, but the control and infested plants within a species were indistinguishable. A total of 51.8% of the variation in this PCA was explained by four PCs. In (B–D), the infested and control plants were distinguishable, indicating differences in metabolic fingerprints following herbivory.

Regardless of whether or not a plant had been eaten, the three plant species had different metabolic fingerprints when analysed by PCA (Fig. 2A), and thus the effects of *P. rapae* herbivory on the metabolic fingerprints were smaller than interspecific differences among plants. Subsequent PCAs performed separately on the three plant species showed that control plant samples and infested plant samples were distinguished in PC score plots (Fig. 2B–D), and there were statistically significant differences in PC scores (*t*-tests: *B. oleracea* PC2 scores, *t*_18_=5.52, *P*<0.001; *C. spinosa* PC1 scores, *t*_18_=–4.62, *P*<0.001; *L. annua* PC2 scores, *t*_18_=3.66, *P*<0.01). This demonstrated that the metabolic fingerprints had been changed by *P. rapae* herbivory in all three species of plant.

### Common mass features exist among the three plant species but induced mass features are unique

**Fig. 3.**
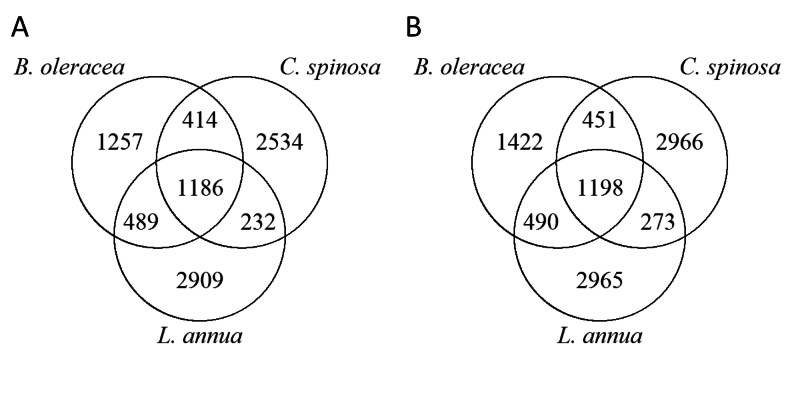
Venn diagrams summarizing the number of shared and unique mass features in three plant species. (A) Mass features measured in control plants. (B) Mass features measured in plants infested with *P. rapae*. In control plants, 74% of the 9021 mass features measured were unique to one plant, 13% were shared by two species, and were 13% shared by three species. In infested plants, a total of 9765 mass features were measured and the equivalent percentages were 75% unique, 12% shared by two plants, and 12% shared by three plants.

When the mass features detected in the three plant species were cross-referenced, there were a large number of mass features (1186; 13% of mass features measured in control plants) that were found in the control plants of all three species (Fig. 3A). There was a similar number of mass features (1198; 12% of mass features measured in the infested plants) that were common to infested plants from all three plant species (Fig. 3B). However, the majority of mass features (74% of measured mass features in control and 75% in infested plants) were unique to only one plant species (Fig. 3).

**Fig. 4.**
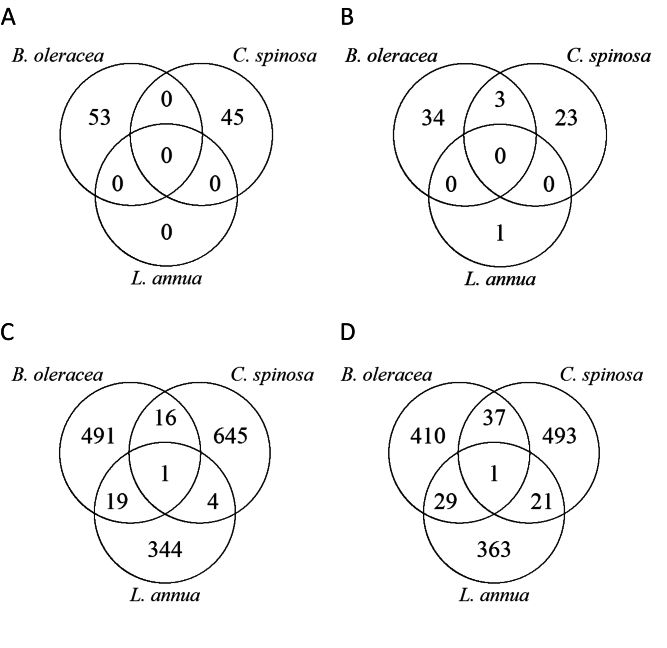
Number of mass features that increased (A) and decreased (B) in infested plants according to *q*<0.05 (*P* values corrected for false discovery rate). *L. annua* only had one mass feature that decreased after *P. rapae* herbivory under these criteria. The numbers of mass features found to have changed in more than one plant species are indicated by overlapping circles. Also shown are the number of increased (C) and decreased (D) mass features that changed significantly according to *P*<0.05 (before false discovery rate correction). This confirmed that the results were not due primarily to the false discovery rate correction applied.

There was an expectation that the two more closely related species (*B. oleracea* and *L. annua*) would have more metabolites in common than with the more distantly related *C. spinosa*. However, the results did not support this, with the number of mass features in common between *B. oleracea* and *C. spinosa* (414) approaching the number in common between *B. oleracea* and *L. annua* (489). Nor were these taxonomic relationships between the three species reflected in the common mass features induced by herbivory (Fig. 4): *B. oleracea* and *C. spinosa* had a larger number of induced mass features in common (3; 53 before correction for false discovery rate) than *L. annua* and either *B. oleracea* or *C. spinosa* (respectively 0 and 0; 48 and 25 before correction for false discovery rate).

Of those mass features that were considered to have changed significantly in abundance following herbivory (induced mass features), there were no induced mass features that were common to all three host-plant species (Fig. 4A, B). This result was partly due to the low number of mass features (one) found to be changed by herbivory in *L. annua* (after correction for false discovery rate). However, even before false discovery rate correction there were only two mass features in common among the three plant species (Fig. 4C, D; 0.07% of all the mass features found to change according to *P*<0.05). Thus, we concluded that there was no evidence for a common response to herbivory in these host-plant species.

### One glucosinolate is induced in *B. oleracea*

**Fig. 5.**
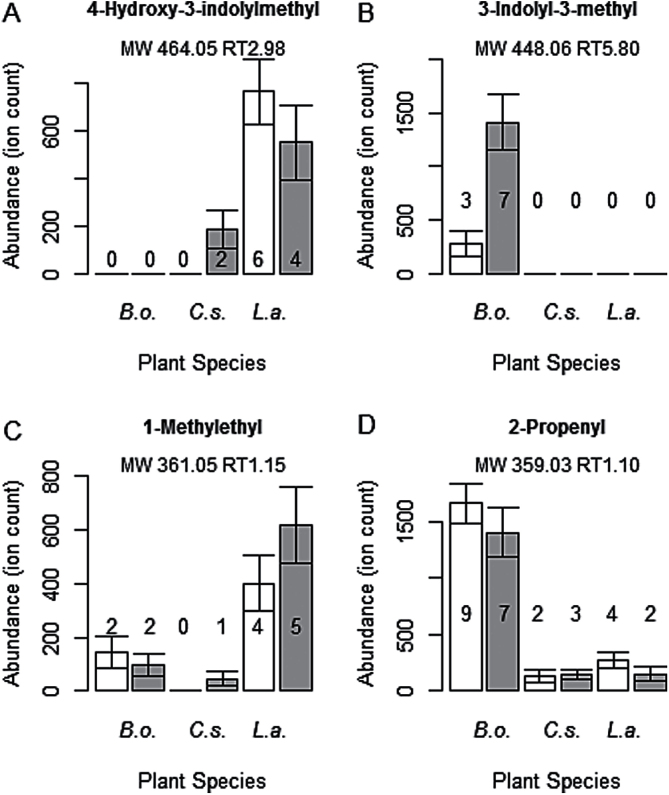
Average abundances of four glucosinolates in *B. oleracea* (*B.o.*) *C. spinosa* (*C.s.*), and *L. annua* (*L.a.*). White bars are control plants, grey bars are infested plants. Means±standard deviation are plotted. The molecular weight (MW) and retention time (RT) of the metabolite is shown above the graphs. Numbers on the bars are the number of samples out of 10 replicates that the metabolite was detected in.

A total of 13 mass features detected in this study had the same molecular weight as 13 glucosinolates. Four of these preliminary identifications were confirmed as glucosinolates, based on the presence of sulfate ions. These were 4-hydroxy-3-indolylmethyl, 3-indolyl-3-methyl (also known as glucobrassicin), 1-methylethyl, and 2-propenyl (also known as sinigrin). The mean abundances of these glucosinolates as measured by HPLC/MS and the number of plant replicates in which the metabolites were detected are shown in Fig. 5. These four metabolites showed variation in presence and abundance among the three plant species. The only glucosinolate to increase in abundance between control and infested plants was 3-indolyl-3-methyl in *B. oleracea* (Fig. 5B; Mann–Whitney *U* test, *P*<0.001). 2-Propenyl was detected in all three species of plant but at the highest abundance in *B. oleracea* (Fig. 5D). These two glucosinolates, 3-indolyl-3-methyl and 2-propenyl, have been recorded previously in *B. oleracea* (Poelman *et al.*, 2008; Kabouw *et al.*, 2010; Gutbrodt *et al.*, 2012). 1-Methylethyl was detected in all three plants; however, it was recorded at highest abundance in *L. annua* (Fig. 5C), a species it has been recorded in previously (Daxenbichler *et al.*, 1991; Vaughn *et al.*, 2006). 4-Hydroxy-3-indolylmethyl was more abundant in *L. annua* compared with the other two plant species (Fig. 5A), but there have been no other previous studies reporting it in *L. annua*.

### Plant metabolites cannot be related to the insect growth rate

**Fig. 6.**
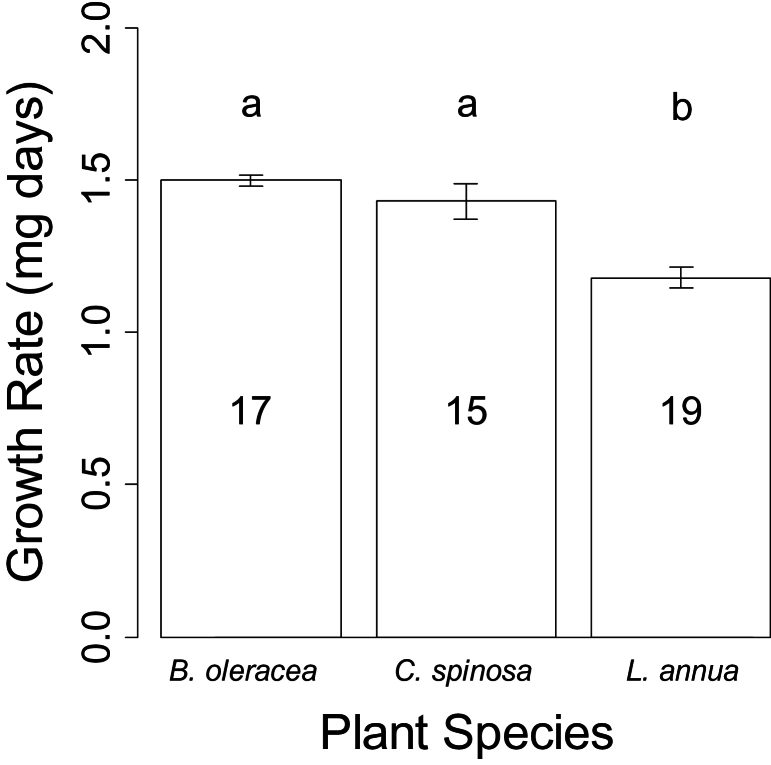
Mean growth rate of *P. rapae* raised on three host plant species. The growth rates on host plants with different letters are statistically different from one another at the 1% level according to post-hoc Tukey tests. Means±standard deviation are plotted. The number of replicates is denoted in each bar.

To evaluate if insect growth success reflected any similarities or differences in metabolite composition among host plants, the growth rate of larvae on the three plants was recorded. *P. rapae* larvae grew at least 21.36% faster on *B. oleracea* and *C. spinosa* than on *L. annua* (Fig. 6; one-way ANOVA, *F*_2,48_=12.41, *P*<0.001; post-hoc Tukey test, *B. oleracea* and *C. spinosa P*=0.61, *B. oleracea* and *L. annua P*<0.001, and *C. spinosa* and *L. annua P*<0.01). There were no induced mass features common to all three plants (Fig. 3) and therefore these cannot be related to the variation in insect performance. Of the three plant species studied, *L. annua* had the fewest mass features changed by herbivory (Fig. 4) and the lowest insect growth rate. However, it is problematic to relate the insect performance to the number of mass features induced because insect performance may be driven by individual components of the metabolic fingerprint rather than the total quantity of metabolites. These results demonstrated that between-species variation in *P. rapae* insect performance was equal to or greater than the extent of variation reported from single plant species. The range in mean pupae weights in this experiment was 19mg (lowest 152mg; highest 171mg). Another study measuring *P. rapae* pupal weights among plant species found a range of approximately 35mg (Hwang *et al.*, 2008). By contrast, the ranges of *P. rapae* pupae weights from within-species studies on cultivars and populations of *B. oleracea* were smaller (approximately 14mg, Gols *et al.*, 2008; 13mg, Harvey *et al.*, 2007; 19mg, Poelman *et al.*, 2008).

## Discussion

The metabolic fingerprints of all three plant species were altered by insect herbivory (Fig. 2B–D). Cross-referencing of mass features among host plants revealed a large number of mass features that were common to all three plants (Fig. 3). Despite this, cross-referencing of mass features induced by herbivory revealed that there were no induced mass features that were common in all three host plants (Fig. 4).

### Plant responses to herbivory by *P. rapae*

Previous studies encompassing a wide range of plant species have shown that herbivory elicits a change in the concentration of individual plant metabolites (for examples, see reviews by Textor and Gershenzon, 2009; Pavarini *et al.*, 2012; Zhang *et al.*, 2012). Far fewer studies have shown collective changes in the overall metabolic fingerprints of plants induced by herbivory (Widarto *et al.*, 2006; Sutter and Müller, 2011; Kutyniok and Müller, 2012; Plischke *et al.*, 2012; Marti *et al.*, 2013). In this study, we extended these previous studies to show herbivore-induced changes in metabolic fingerprints of three host-plant species, *B. oleracea*, *C. spinosa*, and *L. annua* eaten by *P. rapae* (Fig. 2). Previous research on *B. oleracea* has shown that *P. rapae* induces genome-wide changes in *B. oleracea* by inducing the transcription of a number of genes (Broekgaarden *et al.*, 2007), and our results confirmed that those transcriptional changes were translated into changes in metabolite composition.

Herbivory by *P. rapae* has been found previously to increase glucosinolate metabolites in *A. thaliana*, *Brassica nigra*, *Lepidium virginicum*, *Raphanus raphanistrum*, *R. sativus*, and *B. oleracea* (Agrawal *et al.*, 2002; Traw, 2002; Agrawal and Kurashige, 2003; Shelton, 2005; Mewis *et al.*, 2006). Our results found one glucosinolate that increased in infested *B. oleracea* plants (Fig. 5B), supporting findings from a previous study (Agrawal and Kurashige, 2003). The increase in glucosinolates that are effective against attacking generalist herbivores (Li *et al.*, 2000; Müller *et al.*, 2010) suggests that *B. oleracea* mounts a defence response when attacked by the specialist *P. rapae*, despite this insect being undeterred by glucosinolates (Renwick and Lopez, 1999). Few studies have measured glucosinolates in *C. spinosa* or *L. annua* (Daxenbichler *et al.*, 1991; Griffiths *et al.*, 2001; Vaughn *et al.*, 2006) and the detection of 4-hydroxy-3-indolylmethyl is a new glucosinolate recording for *L. annua*. Collectively, these results suggest the extent to which glucosinolate presence and abundance varies among these Brassicales plant species.

### Shared metabolites in plants

The large number of mass features (over 1100) present in all three species regardless of herbivory (Fig. 3) demonstrates the presence of chemicals that are shared among these plant species. Given that the study species are all in the order Brassicales, and considering that metabolites are the end expression of the genome (Sumner *et al.*, 2003), we expected a degree of metabolite similarity among the related plant species we studied. However, the extent of the similarities (13% of all mass features measured in our control plants) has not been shown before using an untargeted method such as metabolic fingerprinting. Primary metabolites may account for some of these shared 1100 mass features (Pichersky and Lewinsohn, 2011), representing metabolites fundamental for key functions essential to life. These shared mass features could also contain signalling hormones such as jasmonates (Wasternack, 2007) or the precursors, intermediates, and derivatives of primary metabolites or phytohormones. However, it is difficult to determine if these common mass features also include secondary metabolites that have a defensive role against herbivores. For this reason, we measured mass features induced by herbivory in order to focus on a group of metabolites that were more likely to contain metabolites with a defensive function.

In comparison with the shared mass features found in the three plants (Fig. 3), the number of shared mass features induced by herbivory was very low (Fig. 4). This lack of shared induced mass features implies that the metabolic reaction to herbivory in these plants is species specific. A previous study showed that in *B. oleracea* (cabbage) and *Tropaeolum majus* (nasturtium), the release of volatile organic compounds (gases that attract parasitoids) in reaction to herbivory by *P. rapae* is plant species specific (Geervliet *et al.*, 1997). In addition, species-specific transcription of genes has been found in *Nicotiana attenuata* (tobacco) and *Solanum nigrum* (nightshade) following attack by *Manduca sexta* (moth) (Schmidt *et al.*, 2005). Therefore, a similar pattern may apply to non-volatile metabolites that are the end result of gene activity. High species specificity of metabolites induced by herbivory would support the suggestion that plants continually produce novel metabolites, including new defence compounds (Jones and Firn, 1991), through mechanisms such as gene mutations and duplication (Kliebenstein *et al.*, 2001; Kampranis *et al.*, 2007; Kliebenstein, 2008), enabling plants to compete successfully in evolutionary arms races with their insect herbivores (Mithofer and Boland, 2012). We conclude that these evolutionary processes may explain why the study species did not contain a large proportion of shared mass features. This is supported by the fact that *C. spinosa* is in the plant family Cleomaceae, which diverged from the family Brassicaceae around 65 million years ago, and *B. oleracea* and *L. annua* are in different tribes, which split around 50 million years ago (Beilstein *et al.*, 2010).

This study examined one cultivar of each plant species and therefore did not quantify within-species variation in metabolic fingerprints. Within-species differences with respect to metabolites have been recorded (Kabouw *et al.*, 2010; Houshyani *et al.* 2012). In a study of barley (*Hordeum vulgare*) infected with a fungus, there were greater differences between the metabolic fingerprints of the control and infected plants than between the nine barley varieties (Cajka *et al.*, 2014). This contrasts with this study where infection, albeit from an insect and not a fungus, did not invoke a larger difference in the metabolic fingerprint than was observed between the plant species. This suggests that metabolite changes resulting from attack are greater than within-species plant variation but smaller than variation among different plant species. Few studies have simultaneously measured metabolite differences within and between plant species (Poelman *et al.* 2008), and only one study of four pepper species (*Capsicum* spp.) encompassing 32 accessions has examined this using metabolic fingerprinting. This confirmed that within-species metabolic fingerprinting variation is smaller than variation between species, but in one of the species, there was considerable variation among accessions (Wahyuni *et al.*, 2013). This raises the possibility that plant genotype is important, and that other varieties of *B. oleracea*, *L. annua*, or *C. spinosa* might have more similar metabolomes to the other two plant species and share more mass features than were recorded here.

### Insect performance and plant metabolites

There were no common induced mass features among the three host-plant species, and therefore these common mass features could not be compared with the variation in insect performance on the different plants. The magnitude of the induced mass features is too general a measurement to predict insect performance for two reasons. First, is it just as likely that one or a small number of metabolites are determining the effect on the insect. Secondly, the effect of those metabolites induced that are intended by the plant to be defensive and negative on the insect may actually be positive to a specialist insect, for example the phagostimulant effects of glucosinolates on *P. rapae* (Renwick and Lopez, 1999). In addition, insect performance may be driven by factors other than metabolites such as the presence of leaf trichomes (Clauss *et al.*, 2006), and total nitrogen or water content (Coley *et al.*, 2006).

## Conclusion

The results from this study revealed that three host plants of *P. rapae* share mass features, but virtually none of those mass features was induced by herbivory. Therefore, there is no support for the idea that these related host plants induce the same metabolites following *P. rapae* attack. This study compared large numbers of mass features between plant species and introduced an approach that could be applied in other studies to examine the evolution of metabolite diversity and how that diversity is shaped by the evolutionary pressures of insect herbivore attack.

## Supplementary data

Supplementary data are available at *JXB* online.

Supplementary Table S1. Retention times and *m*/*z* ratios of the detected mass features as measured by LC-MS.

Supplementary Data

## Abbreviations:

ANOVA: analysis of variance
HPLC: high-performance liquid chromatography
MS: mass spectrometry
PC: principle component
PCA: principle components analysis.
