# Supplementary material for: Analysis of plant leaf metabolites reveals no common response to insect herbivory by Pieris rapae in three related host-plant species
Source: J Exp Bot. 2015 Feb 24;66(9):2547–56. doi: 10.1093/jxb/erv045 (PMC4986865; doi:10.1093/jxb/erv045)
Supplement: Supplementary Data [file supp_66_9_2547__index.html]

Analysis of plant leaf metabolites reveals no common response to insect herbivory by Pieris rapae in three related host-plant species — Analysis of plant leaf metabolites reveals no common response to insect herbivory by Pieris rapae in three related host-plant species — Supplementary Data 

# Analysis of plant leaf metabolites reveals no common response to insect herbivory by *Pieris rapae* in three related host-plant species

## Supplementary Data

Data files

**Files in this Data Supplement:**

- Supplementary Data - Supplementary Data
