# Supplementary material for: Analysis of plant leaf metabolites reveals no common response to insect herbivory by Pieris rapae in three related host-plant species
Source: J Exp Bot. 2015 Feb 24;66(9):2547–56. doi: 10.1093/jxb/erv045 (PMC4986865; doi:10.1093/jxb/erv045)
Supplement: Supplementary Data [file supp_erv045_jexbot129676_file001.pdf]

Analysis of plant leaf metabolites reveals no common response to insect herbivory by *Pieris rapae* in three related host plant species.

A.C. Riach, M.V.L. Perera, H.V. Florance, S.D. Penfield and J.K. Hill

Table S1 The mass feature/compound number (CPD No.), the retention time (RT) and mass to charge ratio (m/z ratio) of mass features measured in the study. A total of 11,649 mass features were detected from control and infested plants of *B. oleracea* , *C. spinosa* and *L. annua* .

CPD No.	RT	m/z ratio	CPD No.	RT	m/z ratio	CPD No.	RT	m/z ratio
CPD1000	1.1323332	664.92868	CPD29849	18.1950002	327.2193	CPD50664	7.19588404	320.184105
CPD10001	33.117739	417.864639	CPD2985	16.7866	298.24871	CPD50668	7.21300361	745.146973
CPD10003	33.1285	383.873967	CPD29850	18.2031669	427.204275	CPD5067	33.153943	87.944497
CPD10004	33.1265499	671.768	CPD29851	18.1971658	405.173717	CPD50670	7.22167279	737.159117
CPD10005	33.1278503	272.92117	CPD29852	18.1931108	191.100522	CPD50671	7.18784841	858.238446
CPD10007	33.1285787	461.841337	CPD29856	18.2040548	402.299194	CPD50673	7.16156111	1253.30798
CPD10008	33.1299117	421.872409	CPD29857	18.2091496	660.481885	CPD50674	7.22399723	168.0423
CPD10016	33.125846	571.822281	CPD29861	18.239231	738.285285	CPD50676	7.22369466	192.0066
CPD1002	1.1123932	542.12234	CPD29862	18.2670998	675.388255	CPD50677	7.22093733	347.120856
CPD10021	33.1330477	581.802138	CPD29863	18.276517	627.389455	CPD50678	7.23964821	885.221092
CPD1003	1.1281334	840.03877	CPD29867	18.3326254	672.27812	CPD50679	7.24513844	788.182493
CPD10030	33.1296399	348.402904	CPD29869	18.1615338	423.33356	CPD5068	33.155354	287.92056
CPD10034	33.1302223	483.82415	CPD29870	18.2901941	331.251067	CPD50680	7.23135333	934.237979
CPD10040	33.1372917	538.808108	CPD29871	18.3729091	411.333622	CPD50681	7.25577632	868.380907
CPD10041	33.1407581	333.84699	CPD29878	18.4840694	373.262028	CPD50682	7.22588915	772.186406
CPD10044	33.1367588	437.856086	CPD2988	16.819453	425.33277	CPD50683	7.22660413	1062.26296
CPD1005	1.1292147	594.0795	CPD29880	18.4098182	661.373918	CPD50684	7.25635295	868.8811
CPD10050	33.1304376	599.790012	CPD29882	18.40455	684.481615	CPD50686	7.26193388	1042.25956
CPD10052	33.1331502	414.872315	CPD29889	18.446532	329.23506	CPD50688	7.26625542	648.647225
CPD10061	33.199229	159.9977	CPD29891	18.48334	388.283176	CPD50689	7.25099508	1112.26483
CPD10067	33.1811412	170.999111	CPD29893	18.42845	651.389565	CPD5069	33.208287	180.98077
CPD1007	1.1360715	402.98959	CPD29894	18.4337635	363.218229	CPD50690	7.2557318	950.224
CPD10071	33.1714438	273.044422	CPD29898	18.4470526	378.297342	CPD50691	7.26371717	648.147664
CPD1008	1.1297133	129.04125	CPD29899	18.4053548	299.245617	CPD50692	7.26421549	1274.31518
CPD1009	1.1159057	119.05799	CPD2990	16.962032	293.23645	CPD50693	7.26352683	656.63382
CPD10090	33.2251249	78.0124444	CPD29905	18.4995001	303.220825	CPD50694	7.27378143	656.132458
CPD10099	33.2667808	129.954169	CPD29906	18.5296249	347.246237	CPD50695	7.25594184	1042.45788
CPD1010	1.1202119	105.04305	CPD29908	18.5240987	428.34779	CPD50696	7.26731723	664.11895
CPD10100	33.3671754	94.0048087	CPD2991	16.796516	322.15659	CPD50697	7.26111415	1042.05872
CPD10102	33.3446227	335.900154	CPD29914	18.5272956	373.261539	CPD50698	7.25984657	868.548769
CPD10108	33.5528993	161.962008	CPD29915	18.6934603	349.261186	CPD50699	7.25493105	869.047271
CPD1011	1.1473641	87.031687	CPD2992	16.9733	342.22189	CPD5070	33.18766	326.91143
CPD10111	33.2457473	133.071168	CPD29920	18.547904	411.333367	CPD50700	7.27093032	664.62152
CPD10113	33.5078334	108.940678	CPD29926	18.6904373	369.267913	CPD50701	7.25942528	1042.85668
CPD10114	33.2910584	958.957886	CPD2993	17.002249	356.31058	CPD50702	7.26992681	869.21502
CPD10119	33.3970644	71.9840636	CPD29930	18.6959277	957.342479	CPD50703	7.28111288	669.354989
CPD1012	1.099446	153.02222	CPD29935	18.7294999	305.23634	CPD50706	7.28509098	802.19605
CPD10120	33.4380277	72.9821087	CPD29938	18.8041287	405.3234	CPD50708	7.24381048	1008.438
CPD10121	33.4105739	213.912653	CPD2994	16.858795	519.33189	CPD5071	33.165426	359.84339
CPD10123	33.4608597	191.968888	CPD29942	18.8308667	438.3491	CPD50715	7.37532888	733.293587
CPD10126	33.2899482	166.005521	CPD29947	18.8872348	485.407163	CPD50716	7.37407305	410.190657
CPD10127	33.5289141	304.895444	CPD29949	18.8523167	428.347425	CPD50717	7.38561895	897.361873
CPD10130	33.5508378	108.939925	CPD2995	17.052809	315.31178	CPD50718	7.40493054	902.315131
CPD1014	1.1236992	516.00324	CPD29950	19.1549881	471.3856	CPD50719	7.40120386	678.252585
CPD10140	33.6247706	153.966757	CPD29951	18.9763594	372.3191	CPD5072	33.163868	275.94338
CPD10146	33.3807123	296.874916	CPD29952	18.8644996	236.17455	CPD50720	7.38948917	502.1803
CPD10148	33.482612	94.96049	CPD29956	19.0235275	313.261279	CPD50722	7.39153378	1011.25026
CPD1016	1.1319474	824.05436	CPD29958	19.0732057	196.1177	CPD50727	7.41507243	832.871508
CPD1017	1.1401134	262.15043	CPD2996	17.070994	293.23636	CPD50728	7.41392711	999.446753
CPD10187	1.11611309	61.0559083	CPD29961	18.9382999	337.26029	CPD50729	7.41046807	1000.04635
CPD10207	1.01187352	282.126218	CPD29965	18.9590694	485.406947	CPD50730	7.41080914	478.09857
CPD1021	1.1457923	673.96878	CPD29966	19.0898131	425.350256	CPD50731	7.40765806	192.148687
CPD1022	1.1263513	147.05452	CPD29970	19.1532769	754.2808	CPD50732	7.4156683	833.03804
CPD1023	1.5352353	198.02987	CPD29972	19.2161864	447.331758	CPD50733	7.42068873	833.205215
CPD10237	1.30193275	264.274394	CPD29974	19.1380445	466.269506	CPD50735	7.41335758	349.086182
CPD1024	1.2034701	260.13365	CPD29975	19.0568002	730.320087	CPD50738	7.41653503	999.84428

CPD1026	1.2893415	151.02953	CPD29976	19.0957501	357.267535	CPD5074	33.178166	243.0508
CPD1027	1.1143384	101.04827	CPD29979	19.1099547	506.260143	CPD50743	7.44688108	626.1465
CPD1029	1.1368813	336.0762	CPD2998	17.033153	453.28576	CPD50744	7.44815456	836.331992
CPD1030	1.1424	777.06059	CPD29981	19.1362031	989.368389	CPD50746	7.47677669	834.374
CPD1031	1.4243067	332.07948	CPD29983	19.0716966	471.391315	CPD50747	7.46462709	1001.44896
CPD10323	1.00658548	362.018868	CPD29988	19.1755831	613.400067	CPD50748	7.45928737	1001.24832
CPD10327	1.01772233	417.870828	CPD29989	19.132226	196.116682	CPD50749	7.47142855	834.707858
CPD10328	1.02116676	633.791	CPD29990	19.1235327	313.261453	CPD50750	7.46539996	1000.84943
CPD10331	1.01609928	549.8295	CPD29991	19.1483337	682.264711	CPD50751	7.46415086	1001.04862
CPD10338	1.03128572	287.868336	CPD29997	19.1999251	662.239677	CPD50752	7.477991	1001.64952
CPD1034	1.1235334	115.06432	CPD29998	19.2010499	672.279235	CPD50754	7.47353391	834.208388
CPD1036	1.1017072	418.96911	CPD3000	17.10183	346.22871	CPD50755	7.46390052	523.152109
CPD1037	1.1430001	68.026933	CPD30001	19.2443052	629.372115	CPD50760	7.54940212	574.153243
CPD10374	1.0425883	362.899941	CPD30002	19.2379997	388.281171	CPD50761	7.48723156	842.377175
CPD10385	1.03944441	479.836689	CPD30003	19.2976912	396.252983	CPD50764	7.5753706	1010.45094
CPD1039	1.1385016	132.05118	CPD30004	19.2654475	329.235632	CPD50766	7.62652569	1010.65115
CPD1040	1.1466045	434.94381	CPD30007	19.3109166	684.479725	CPD50768	7.57394472	934.2444
CPD1041	1.1391699	475.15551	CPD30008	19.3927518	674.498189	CPD50770	7.56592123	1010.85281
CPD10411	1.04428603	1001.64114	CPD3001	17.146574	283.22995	CPD50771	7.61090337	1011.25553
CPD10417	1.04742861	489.874086	CPD30010	19.1797285	425.350168	CPD50773	7.57869375	1096.26771
CPD1042	1.1184031	83.037391	CPD30011	19.3133637	997.484318	CPD50777	7.61783032	744.166808
CPD1043	1.1236605	350.06352	CPD30012	19.1978719	345.267269	CPD50778	7.65862958	1014.05701
CPD10437	1.04799999	473.86915	CPD30015	19.3162296	324.264931	CPD5078	33.166818	118.03804
CPD1044	1.1370739	489.16967	CPD30017	19.3230005	586.280754	CPD50780	7.61388769	1110.30244
CPD1045	1.1617188	175.09639	CPD30019	19.3601382	407.339118	CPD50781	7.63199215	802.204208
CPD1047	1.1811684	403.15473	CPD3002	17.19238	293.23462	CPD50782	7.5996302	1140.31449
CPD10485	1.05543923	576.845037	CPD30025	19.3377271	357.422255	CPD50783	7.67893276	844.713553
CPD10494	1.05839656	221.981335	CPD3003	17.145075	364.23604	CPD50784	7.62429608	752.16913
CPD10499	1.07019299	322.934878	CPD30030	19.3388747	204.149713	CPD50785	7.77518282	831.375
CPD1050	1.1472152	345.04328	CPD30032	19.3514738	378.4057	CPD50787	7.66367857	1112.28332
CPD10507	1.15191166	59.0718579	CPD30033	19.3418999	355.251425	CPD50788	7.67393865	1013.25628
CPD1051	1.1556126	447.15973	CPD30034	19.3562556	151.099064	CPD5079	33.167286	60.00074
CPD10511	1.09500002	353.064514	CPD30038	19.3457223	417.175222	CPD50790	7.65692601	964.238368
CPD10524	1.14849997	522.048631	CPD3004	17.198246	156.10964	CPD50791	7.65247282	516.594753
CPD10530	1.24057893	403.1719	CPD30040	19.2462567	957.34365	CPD50793	7.68099641	844.546787
CPD10532	1.15435715	361.057386	CPD30042	19.3628459	371.295808	CPD50795	7.66755006	640.162545
CPD10534	1.16418786	347.06047	CPD30048	18.9246023	445.31535	CPD50796	7.62727981	1011.05195
CPD10535	1.13944412	273.170122	CPD3005	17.241026	510.15082	CPD50799	7.69549828	1013.85562
CPD10545	1.14872022	517.043994	CPD30050	19.4242312	423.340431	CPD5080	33.165867	101.02677
CPD10547	1.23021263	112.024662	CPD30051	19.4313119	410.316679	CPD50800	7.6639866	845.047246
CPD10554	1.16085184	419.149241	CPD30056	19.4534617	333.264938	CPD50801	7.67239668	1126.27951
CPD10555	1.18300019	291.016343	CPD30057	19.4487133	674.498754	CPD50802	7.66969072	786.203326
CPD10556	1.31759003	343.114771	CPD30059	19.4890997	471.38978	CPD50803	7.70066727	531.097567
CPD10557	1.24245135	281.092305	CPD3006	17.239157	526.12398	CPD50804	7.68834685	846.219641
CPD10558	1.50143384	433.18679	CPD30060	19.48515	343.41274	CPD50805	7.67588689	844.880406
CPD10559	1.31731866	211.007936	CPD30061	19.4878999	343.2511	CPD50806	7.69277567	663.142878
CPD10562	1.33472721	197.019841	CPD30062	19.4876365	973.376218	CPD50807	7.69224552	1008.27212
CPD10564	1.18775	385.186442	CPD30063	19.5741396	331.252111	CPD50808	7.69331908	832.205279
CPD10565	1.21170907	194.114288	CPD30065	19.424913	438.34985	CPD5081	33.131812	557.80225
CPD10566	1.18232468	371.172671	CPD30067	19.5176925	472.353192	CPD50810	7.68728457	655.154843
CPD10569	1.26652219	218.9898	CPD30068	19.5184444	512.349572	CPD50811	7.6955415	1288.33058
CPD1057	1.1447029	786.09556	CPD3007	17.24474	505.19563	CPD50813	7.69535796	948.251282
CPD10572	1.21581724	285.106836	CPD30070	19.5571452	499.422413	CPD50814	7.67255124	816.2097
CPD10574	1.32968197	460.000789	CPD30073	19.5576666	1044.48772	CPD50815	7.69810797	1013.45644
CPD1058	1.124598	69.058498	CPD30074	19.5532309	1065.47187	CPD50816	7.69521899	1010.44878
CPD10584	1.39031853	129.041855	CPD30075	19.5645457	1062.48244	CPD5082	33.17284	77.015261
CPD10585	1.39165962	73.0251571	CPD30077	19.5731996	656.22531	CPD50824	7.70205566	523.111824
CPD10588	1.34634266	276.024445	CPD30079	19.6236008	1019.48782	CPD5083	33.162272	81.017146
CPD1059	1.1530002	540.09583	CPD3008	17.239802	352.12931	CPD50838	7.69900458	1156.2904
CPD1060	1.133118	133.03993	CPD30081	19.5475816	696.481521	CPD50839	7.70360495	994.24563
CPD10601	1.52057214	324.106558	CPD30084	19.644714	662.7285	CPD50841	7.70974612	1318.34227
CPD10605	1.57534504	130.023933	CPD30085	19.6379332	353.397656	CPD50844	7.71135127	670.659855
CPD10608	1.56209528	209.230476	CPD30088	19.6287706	331.252086	CPD50845	7.71489702	1345.80664
CPD10609	1.62835973	151.0423	CPD3009	17.268569	672.27873	CPD50848	7.71515214	670.160111
CPD10614	1.54550443	111.042643	CPD30091	19.6968926	351.277161	CPD50849	7.71742162	1337.31926
CPD10615	1.5586751	261.132031	CPD30093	19.6640498	390.29734	CPD5085	33.170173	187.91127
CPD10616	1.57834272	188.237221	CPD30094	19.6884531	407.339283	CPD50851	7.71594847	678.145335

CPD10617	1.56559996	650.09484	CPD30095	19.6387696	499.4217	CPD50852	7.7088528	1337.82239
CPD10618	1.5677308	452.070115	CPD30096	19.6292134	309.228871	CPD50853	7.71616186	686.132542
CPD10619	1.56332426	209.181403	CPD30097	19.7043356	991.50783	CPD50854	7.72443517	814.324906
CPD1062	1.1465555	945.01134	CPD30098	19.7312599	497.402415	CPD50856	7.71564226	686.633725
CPD10628	1.46527284	133.037083	CPD30099	19.7461925	941.34696	CPD50857	7.71765251	678.64739
CPD10629	1.55329154	301.130174	CPD30101	19.6602435	650.497835	CPD50858	7.6386703	1010.65258
CPD1063	1.1488749	476.12369	CPD30102	19.7437273	735.301718	CPD50859	7.725	1348.34821
CPD10634	1.56588879	628.056296	CPD30106	19.7934276	142.099	CPD5086	33.176324	424.84069
CPD10636	1.45887934	342.114356	CPD30108	19.7923748	514.362725	CPD50860	7.73221069	190.064432
CPD10639	1.56989985	812.04655	CPD30109	19.7896667	456.359561	CPD50861	7.74781732	303.082209
CPD10646	1.57205532	843.996683	CPD3011	17.190409	346.22815	CPD50865	7.70507113	1014.05649
CPD1065	1.1530013	498.10637	CPD30111	19.7939999	474.364921	CPD50866	7.64067013	1010.8509
CPD10653	1.19028043	557.078393	CPD30112	19.8986758	339.2747	CPD50868	7.75947724	844.713667
CPD10657	1.5772163	310.01203	CPD30113	19.8269746	413.313685	CPD50869	7.76449741	730.173338
CPD1066	1.1508332	548.02208	CPD30114	19.8228904	343.264331	CPD5087	33.1665	89.020758
CPD10664	1.56828699	111.105076	CPD30115	19.980253	485.405557	CPD50870	7.94255897	1054.28201
CPD10666	1.69879111	241.069957	CPD30118	20.0910008	377.236057	CPD50871	7.69363363	1013.65737
CPD10667	1.65064578	52.0123467	CPD30119	19.8399999	866.619189	CPD50875	7.852283	892.092471
CPD1067	1.1707131	170.0224	CPD3012	17.241917	342.22039	CPD50876	7.72170707	842.215544
CPD10671	1.67673781	116.012022	CPD30122	19.8749773	281.198683	CPD50877	7.85750175	835.375033
CPD10672	1.69349995	392.038233	CPD30128	19.8758567	730.320029	CPD50878	7.85752223	892.2533
CPD10673	1.63626725	262.134978	CPD30133	19.8907001	801.64093	CPD5088	33.163571	262.92243
CPD10674	1.66126999	450.051718	CPD3014	17.219976	364.23628	CPD50880	7.86163179	733.500095
CPD10675	1.69744102	376.049482	CPD30147	19.9066999	327.27741	CPD50881	7.87125988	733.358011
CPD10676	1.6838232	131.094779	CPD3015	17.220788	315.31167	CPD50882	7.84774956	1002.24823
CPD10678	1.70229608	289.980093	CPD30150	19.9309315	203.124782	CPD50883	7.83926227	641.5633
CPD1068	1.1377115	682.13157	CPD30158	19.9616154	1302.55832	CPD50884	7.8624475	855.252495
CPD10681	1.74277729	273.167011	CPD3016	17.549164	911.35144	CPD50885	7.85734963	855.087176
CPD1069	1.1513	186.17174	CPD30160	19.9682858	302.135071	CPD50886	7.86587447	855.918047
CPD10692	2.07041515	52.0116957	CPD30161	19.9671177	320.144912	CPD50887	7.85764041	733.784633
CPD10693	1.84633137	202.129894	CPD30162	19.9908453	622.244538	CPD50888	7.85494701	732.931813
CPD10696	2.06880154	83.0308529	CPD30164	20.1919363	331.250974	CPD50889	7.86317541	1002.64904
CPD1070	1.1508243	461.17534	CPD30165	20.1963228	555.433367	CPD50890	7.86235185	855.585058
CPD10701	1.94399819	268.1049	CPD3017	17.356458	283.28576	CPD50891	7.86256985	733.642858
CPD10706	2.03029187	309.132186	CPD30172	20.1430184	439.363542	CPD50892	7.86008161	1026.70155
CPD10707	1.52402231	221.025264	CPD30173	20.0695001	377.28979	CPD50893	7.85626196	733.073761
CPD1071	1.164096	502.02684	CPD30175	20.0925384	682.263985	CPD50894	7.86606943	733.215789
CPD10710	2.0322386	307.105002	CPD30176	20.1019999	543.447167	CPD50896	7.85219415	1026.50207
CPD1072	1.1515162	512.07125	CPD30179	20.1182142	496.3529	CPD50897	7.85736347	1027.10221
CPD10733	2.23062566	349.087591	CPD3018	17.335056	456.17802	CPD50898	7.85904866	1026.30233
CPD10737	2.19800603	317.161156	CPD30180	20.121278	491.392778	CPD50899	7.86489167	855.751168
CPD1074	1.1484708	334.06629	CPD30181	20.1344168	485.406542	CPD5090	33.184859	224.19883
CPD10742	2.51524308	130.041067	CPD30183	20.1706355	308.266373	CPD50901	7.87405581	855.41827
CPD10743	2.49244065	464.18052	CPD30185	20.1635999	250.22385	CPD50902	7.85361421	856.083847
CPD10745	2.51223593	166.1968	CPD3019	17.441467	356.31329	CPD50903	7.87562057	892.425325
CPD10746	2.51724625	102.048032	CPD30190	20.2884747	516.365745	CPD50904	7.85884909	1026.90186
CPD10747	2.51231932	148.052697	CPD30193	20.2488666	355.252893	CPD50906	7.86077278	1002.44888
CPD10748	2.58030575	259.153119	CPD30194	20.1305063	472.338813	CPD50907	7.8693298	835.207217
CPD1075	1.1552821	126.03161	CPD30197	20.2963849	467.38512	CPD50908	7.94752459	1283.62739
CPD10767	2.92009496	242.125491	CPD3020	17.429243	616.50975	CPD50909	7.84575771	1282.87666
CPD1077	1.1427144	941.0361	CPD30203	20.2629491	331.25135	CPD5091	33.166507	81.095439
CPD10777	3.31460985	345.18905	CPD30205	20.3244818	770.502118	CPD50912	7.88628513	892.591229
CPD1078	1.1485291	204.10787	CPD30207	20.3912284	708.502579	CPD50913	7.85245312	835.042867
CPD10797	4.48759973	327.129587	CPD30208	20.3505	499.421012	CPD50915	7.85458146	733.926835
CPD10807	5.06921156	204.214736	CPD3021	17.426658	319.25063	CPD50917	7.87693837	892.757867
CPD1081	1.1535708	286.07533	CPD30212	20.3583997	1019.53772	CPD50919	8.27907017	740.6452
CPD10827	5.63042267	359.204762	CPD30213	20.3578235	688.514941	CPD5092	33.183646	229.88269
CPD1083	1.1423257	303.14315	CPD3022	17.434824	335.22473	CPD50925	7.89769098	868.7552
CPD1084	1.1494667	802.17372	CPD30221	20.3792001	546.8829	CPD50928	7.92563326	544.188584
CPD10841	5.84464816	294.155879	CPD30224	20.4017968	715.097736	CPD50929	7.88269104	864.57486
CPD10848	6.07726972	494.154482	CPD30225	20.3789001	726.01893	CPD5093	33.220555	131.96483
CPD1085	1.1595004	561.06216	CPD30226	20.3757501	912.139938	CPD50930	7.90834639	864.24251
CPD10859	6.10777461	529.763079	CPD3023	17.420695	279.25571	CPD5094	33.1755	297.90954
CPD10869	6.23861983	176.047014	CPD30231	20.3803753	798.488437	CPD50941	7.98833386	438.096367
CPD1087	1.1548984	276.02651	CPD30232	20.3826661	716.766583	CPD50944	7.98361517	1110.28386
CPD10873	6.49282861	567.636061	CPD30233	20.3765626	912.639913	CPD50947	8.02164817	678.64545
CPD10874	6.39741349	244.09224	CPD30234	20.3805	716.86329	CPD50949	8.04504304	670.15185

CPD1088	1.1344298	75.033125	CPD30237	20.3811665	368.752994	CPD5095	33.18758	288.91616
CPD10885	6.56971737	194.058017	CPD30238	20.3771115	1155.74773	CPD50951	8.01285751	978.246357
CPD10887	6.4346681	433.193433	CPD30239	20.3791251	554.873013	CPD50954	8.05729461	1156.30903
CPD10897	6.62676755	333.1334	CPD30247	20.3698419	134.074318	CPD50956	8.06810458	1381.3452
CPD10900	6.65184925	343.123325	CPD30249	20.3884116	198.108912	CPD50958	8.06532932	1389.33269
CPD10903	6.61568804	460.218611	CPD30252	20.3866363	1101.75697	CPD50961	8.06471533	1362.83954
CPD10908	6.58396864	354.184442	CPD30254	20.3797226	376.74215	CPD50963	8.06805167	1362.3698
CPD10910	6.65092569	363.18704	CPD30255	20.4339851	379.7985	CPD50966	8.06860341	1200.31579
CPD10914	6.54506858	294.072646	CPD30257	20.3950002	419.18986	CPD50967	8.066842	1389.83409
CPD10916	6.69582854	340.172717	CPD30258	20.3941501	704.544865	CPD50968	8.02861576	802.19598
CPD1094	1.3140544	287.05875	CPD30261	20.3961052	447.236768	CPD50969	8.07355462	1366.83996
CPD10940	6.93351418	262.132075	CPD30262	20.4063687	677.490774	CPD50972	8.05351748	1170.30448
CPD10943	6.88356076	381.195475	CPD30265	20.3999258	472.337853	CPD50974	8.06808945	1367.35368
CPD1095	1.10427	359.03988	CPD30266	20.4101668	377.293542	CPD50975	8.06865045	1373.85362
CPD10950	7.11017154	794.2238	CPD30267	20.3987372	441.220426	CPD50977	8.06949655	1381.84728
CPD1096	1.1583434	84.021944	CPD30268	20.4204234	347.283284	CPD50978	8.07120371	1038.26258
CPD10965	7.23118072	242.125748	CPD3027	17.515752	618.2876	CPD50979	8.06911721	1374.34253
CPD10969	7.41414052	212.033757	CPD30271	20.3082999	467.384877	CPD5098	33.175564	61.000549
CPD1097	1.1968473	56.027044	CPD30272	20.42725	416.315795	CPD50982	8.0768032	708.646425
CPD10972	7.099745	449.227106	CPD30274	20.4221579	402.324768	CPD50983	8.0761207	707.646422
CPD10976	7.42325025	126.031303	CPD30278	20.560047	612.2591	CPD50984	8.07099569	832.20502
CPD1098	1.186654	430.11347	CPD3028	17.580864	266.16444	CPD50985	8.07454469	994.25773
CPD10983	7.84098441	324.049543	CPD30280	20.4624	365.292835	CPD50987	8.07489686	708.14509
CPD10985	7.62113113	772.203693	CPD30287	20.4906968	327.277311	CPD50989	8.07546112	1374.83769
CPD1099	1.1660532	313.0986	CPD3029	17.580586	342.32262	CPD50991	8.06615804	1359.33802
CPD10992	7.64314866	443.289958	CPD30290	20.565625	702.530169	CPD50998	8.09400197	626.144531
CPD10994	7.70731961	222.107538	CPD30295	20.5639999	730.319706	CPD50999	8.07092939	1008.25238
CPD1100	1.2696928	214.0091	CPD30297	20.5189202	708.5029	CPD5100	33.198344	233.99377
CPD11004	7.92211049	278.160078	CPD30298	20.5708574	724.512607	CPD51000	8.08774711	693.158431
CPD11005	7.95426102	317.173029	CPD30299	20.6808975	622.258175	CPD51001	8.07584403	530.126795
CPD11006	7.9212557	448.100532	CPD30300	20.7475584	513.431856	CPD51003	8.11537445	858.085146
CPD11007	7.9422449	632.133475	CPD30304	20.6076667	523.4209	CPD51004	8.11889373	1039.50169
CPD11012	7.84809658	324.087587	CPD30305	20.5964999	345.26697	CPD51006	8.11125563	1029.69978
CPD1102	1.1581024	371.11069	CPD30308	20.6483662	874.450173	CPD51008	8.10387403	842.321444
CPD11028	8.22949203	317.173008	CPD3031	17.644431	448.27985	CPD5101	33.164881	323.91715
CPD1103	1.1367083	445.17817	CPD30313	20.6090235	385.310478	CPD51010	8.28453123	1030.09985
CPD11031	8.24906789	193.109293	CPD30317	20.7206177	335.293308	CPD51012	8.10910668	464.095589
CPD11038	8.46178244	700.150589	CPD30319	20.683623	712.513918	CPD51013	8.11387976	858.249611
CPD11039	8.49950658	365.194633	CPD3032	17.634039	598.40131	CPD51014	8.14498268	858.416521
CPD11043	8.55125716	452.335423	CPD30320	20.7060829	586.278567	CPD51016	8.11428869	866.255911
CPD11047	8.64500757	159.068589	CPD30321	20.7066246	678.5224	CPD51017	8.18337573	861.743662
CPD1105	1.1608333	94.005667	CPD30324	20.715125	684.501981	CPD51024	8.10857931	858.58345
CPD11055	8.67217663	206.150405	CPD30327	20.7127986	453.38233	CPD51026	8.13385823	1080.14423
CPD1106	1.1710805	284.08702	CPD3033	17.409864	356.31209	CPD51027	8.19942298	732.2114
CPD1107	1.1646668	114.03254	CPD30330	20.7268	333.26748	CPD51028	8.15037069	440.224838
CPD11072	9.21295682	381.16426	CPD30340	20.7580557	355.413606	CPD51029	8.13699558	1079.8136
CPD11079	9.33862695	659.25555	CPD30341	20.7589504	355.250605	CPD5103	33.182849	270.90925
CPD1108	1.1550651	510.07079	CPD30342	20.7633579	584.264714	CPD51030	8.13273422	1079.98151
CPD11081	9.39469598	494.1737	CPD30345	20.7745715	629.496043	CPD51031	8.15738371	865.922287
CPD11084	9.4653808	527.188875	CPD30346	20.7812728	383.287445	CPD51033	8.13764066	1080.31129
CPD11089	9.5958541	246.122471	CPD30347	20.7903499	499.423015	CPD51034	8.16309713	1026.89839
CPD1109	1.1572941	235.17744	CPD30348	20.78348	732.43861	CPD51035	8.13711121	1079.65258
CPD11092	9.71883773	680.266507	CPD30349	20.8158461	608.486492	CPD51036	8.14418343	1296.37346
CPD11096	9.89956293	778.1561	CPD3035	17.725745	219.12531	CPD51037	8.14297356	884.752725
CPD1110	1.1565841	199.02487	CPD30350	20.7988579	513.434214	CPD51039	8.1429975	1296.1765
CPD11103	9.99314576	230.044594	CPD30352	20.8087502	586.500842	CPD5104	33.176046	127.97674
CPD11104	8.88271322	530.146122	CPD30353	20.8313736	1069.74354	CPD51040	8.16387779	462.207462
CPD11109	10.1359375	344.153631	CPD30355	20.9024439	594.530169	CPD51041	8.14439229	1295.97377
CPD11117	10.2237508	192.126125	CPD30356	20.8727506	941.576288	CPD51044	8.14844475	866.088
CPD11119	10.3681238	662.25845	CPD30358	20.8582993	328.23827	CPD51045	8.17139786	853.251694
CPD1112	1.1562355	556.11117	CPD30359	20.9916964	341.293189	CPD51046	8.18100802	730.930515
CPD11120	10.3597113	922.246921	CPD3036	17.722804	185.14013	CPD51048	8.18811382	897.681978
CPD11121	10.5195524	614.161262	CPD30361	20.8644166	969.595808	CPD51049	8.17951824	852.9188
CPD11122	10.3001879	429.16441	CPD30363	20.9931326	654.267917	CPD5105	33.212282	291.91746
CPD1113	1.1927616	137.99684	CPD30366	20.9219969	373.297793	CPD51050	8.17936577	852.75262
CPD11130	10.7766752	320.174035	CPD30369	20.9508668	624.259907	CPD51053	8.18136797	731.35715
CPD11131	10.8713047	476.215382	CPD30373	20.9702219	227.151322	CPD51054	8.22223764	861.250707

CPD11135	11.2279851	221.144767	CPD30377	20.9651428	243.129157	CPD51055	8.16540214	853.418159
CPD11143	12.0532068	776.288258	CPD30378	21.077646	732.596767	CPD51057	8.17405925	730.788342
CPD1115	1.1489445	329.1111	CPD3038	17.711458	324.22931	CPD51058	8.19863248	886.736863
CPD11153	13.7505093	518.195438	CPD30381	21.0042326	746.424944	CPD51059	8.18220736	853.08536
CPD11166	14.5224706	287.28095	CPD30382	20.9369971	391.309516	CPD5106	33.235792	227.8918
CPD11169	14.8867777	505.757344	CPD30383	20.9636003	826.56638	CPD51062	8.22309999	865.589918
CPD1117	1.1685127	246.04208	CPD3039	17.722948	199.19287	CPD51063	8.20475537	856.41389
CPD11170	14.8868176	513.243991	CPD30390	20.8015545	353.292506	CPD51064	8.17805588	865.755538
CPD11178	15.1675189	676.354433	CPD30392	21.0113635	770.6063	CPD51066	8.17995214	852.586375
CPD11179	15.0227106	698.663875	CPD30395	20.98868	435.274646	CPD51068	8.17766585	1042.91822
CPD1118	1.1547498	376.0483	CPD3040	17.685393	266.16498	CPD51069	8.18733558	1023.30233
CPD11189	15.1530011	486.282362	CPD30406	21.074402	698.29428	CPD5107	33.179979	69.00251
CPD1119	1.1899762	573.04238	CPD3041	17.749487	326.06944	CPD51070	8.17855253	1063.9472
CPD11190	15.219497	394.245114	CPD30414	21.1431295	708.272489	CPD51072	8.17686052	762.64998
CPD11195	15.2610364	256.181432	CPD30415	21.2169174	521.401929	CPD51073	8.17053561	912.600189
CPD11206	16.1234676	1015.52703	CPD30418	21.1172719	379.308639	CPD51074	8.19404601	868.754936
CPD11209	16.7509933	352.261932	CPD3042	17.786084	654.02234	CPD51075	8.16860513	731.499806
CPD1121	1.1461674	414.09607	CPD3043	17.803398	372.30911	CPD51078	8.18882642	868.589625
CPD11210	16.6286208	268.142155	CPD3044	17.723186	219.12524	CPD51079	8.16569182	1022.90416
CPD11211	16.4865225	449.169532	CPD30445	21.1225624	694.582506	CPD51080	8.18079672	1023.10153
CPD11218	16.8264091	276.207145	CPD30446	21.1010026	507.38158	CPD51083	8.17834991	1023.50219
CPD1122	1.153875	704.07276	CPD30447	21.1122225	1039.52829	CPD51084	8.1784198	762.791395
CPD11221	16.8336624	408.266013	CPD30448	21.0650246	131.0946	CPD51085	8.17987557	1042.47201
CPD11229	17.2470956	584.345524	CPD30449	21.1171875	969.525844	CPD51086	8.12945323	866.420277
CPD1123	1.177266	96.021772	CPD3045	17.736195	448.27981	CPD51087	8.19583214	1047.12391
CPD11234	17.6296034	408.284484	CPD30453	21.1275999	720.596673	CPD51088	8.18084556	731.07291
CPD11238	17.8012711	248.175286	CPD30457	21.1277153	342.144971	CPD5109	33.187202	278.90323
CPD11239	17.7546624	372.307204	CPD30458	21.1326151	507.238285	CPD51090	8.18210897	731.215055
CPD1124	1.1570725	183.06442	CPD30459	21.1424508	385.259365	CPD51091	8.17900111	897.958379
CPD1125	1.1599524	279.07805	CPD3046	17.8019	357.9724	CPD51093	8.18050199	1042.11294
CPD11259	18.3128335	716.378011	CPD30461	21.0651078	341.293273	CPD51094	8.22085303	1043.10833
CPD1126	1.1759241	160.03711	CPD30463	21.1485455	1031.55787	CPD51095	8.18638583	1047.29073
CPD11265	18.6450031	818.478815	CPD30464	21.1402188	697.436989	CPD51096	8.18352328	1047.95446
CPD11268	18.92354	329.256076	CPD30465	21.1481575	323.280779	CPD51097	8.18245336	1023.70252
CPD11269	18.8564518	1469.42246	CPD30466	21.0817276	594.528871	CPD51098	8.17159613	1063.78127
CPD1127	1.167952	181.07383	CPD30467	21.1437691	1157.79611	CPD51099	8.18612527	868.256225
CPD1128	1.1562502	723.11707	CPD30468	21.1420001	1015.55074	CPD5110	33.183786	282.89943
CPD11282	19.1537084	657.405105	CPD30469	21.1385455	386.344673	CPD51101	8.17747584	731.642472
CPD11283	19.221575	160.089273	CPD3047	17.740399	185.1403	CPD51103	8.16690081	1276.34767
CPD11285	19.3818184	688.377045	CPD30470	21.1434001	1201.75909	CPD51105	8.18129467	1047.62524
CPD11287	19.4065006	348.287023	CPD30471	21.1596126	674.263038	CPD51106	8.18503222	1033.69825
CPD11288	19.4034642	365.3081	CPD30474	21.1484998	210.13365	CPD51107	8.18158553	1047.78861
CPD1129	1.3535609	304.03642	CPD30478	21.1785453	1056.50375	CPD51108	8.18216808	1047.45979
CPD11298	19.7520379	676.232818	CPD3048	17.758157	335.24257	CPD5111	33.162867	299.91089
CPD1130	1.1528573	459.19506	CPD30482	21.2092223	693.225733	CPD51112	8.19998217	911.937117
CPD11300	19.7201323	814.451175	CPD30483	21.2096947	463.361022	CPD51113	8.17977272	1023.90237
CPD11302	19.6513669	566.324057	CPD30485	21.2380001	1086.49042	CPD51114	8.19560489	1064.11268
CPD11305	20.6237757	990.54984	CPD30489	21.3725618	379.308767	CPD51115	8.18057362	639.81375
CPD11306	20.0000894	511.407237	CPD3049	17.831973	333.21378	CPD51116	8.18420333	1257.14625
CPD11309	20.2180291	298.249958	CPD30492	21.2636498	430.33097	CPD51117	8.17916295	1257.54936
CPD1131	1.2488171	389.10133	CPD30494	21.2098547	371.283588	CPD51118	8.18533177	1256.94659
CPD11311	20.3781618	351.239822	CPD30495	21.2791007	752.543769	CPD51120	8.11627511	887.248375
CPD11312	20.2995374	277.240403	CPD30496	21.2694	432.3286	CPD51121	8.17207158	639.563707
CPD1132	1.1591897	191.15114	CPD30497	21.3482048	359.282824	CPD51123	8.1698016	898.10332
CPD11320	20.4340937	384.284715	CPD30500	21.2698572	730.563143	CPD51124	8.1841087	639.688392
CPD11321	20.3434974	476.42844	CPD30501	21.2856315	399.330095	CPD51126	8.22294618	1075.69048
CPD11330	20.5008192	423.354295	CPD30503	21.2999002	725.25333	CPD51128	8.21372711	1064.29057
CPD11336	20.472199	362.302265	CPD30508	21.3277855	760.332114	CPD51129	8.18177701	1276.74522
CPD11348	20.844945	218.199892	CPD30509	21.356763	367.308225	CPD5113	33.485564	94.959905
CPD11354	20.7063164	367.251769	CPD3051	17.678867	538.31138	CPD51133	8.17510055	912.267064
CPD1136	1.3087066	159.12256	CPD30514	21.3758181	327.276827	CPD51134	8.17913367	1042.29773
CPD1137	1.1346503	196.03929	CPD30517	21.4343523	1095.75834	CPD51135	8.20166252	861.9104
CPD11372	21.4544249	692.420609	CPD30518	21.4369373	620.545363	CPD51137	8.19826326	1063.47315
CPD11377	21.4570831	178.169426	CPD30519	21.4494801	527.441293	CPD51139	8.19532842	738.354564
CPD11381	21.5774025	549.881689	CPD30520	21.4061725	752.539338	CPD5114	33.187736	237.87711
CPD11386	21.6448901	528.379206	CPD30521	21.4700002	660.514811	CPD51141	8.18714646	1256.7541
CPD1140	1.3592433	216.11263	CPD30522	21.4700001	658.551262	CPD51142	8.19786282	1056.09806

CPD11409	21.9230431	456.303943	CPD30523	21.4800002	381.43455	CPD51143	8.19491555	895.91275
CPD1141	1.15815	71.074192	CPD30527	21.4119486	379.30895	CPD51145	8.20684402	880.080921
CPD1142	1.2043913	192.04907	CPD30529	21.4930004	688.25706	CPD51146	8.2077861	879.581678
CPD11420	22.0022342	466.285755	CPD3053	17.886054	527.40304	CPD51147	8.38801772	1056.73096
CPD11423	22.2033661	262.230109	CPD30530	21.4129239	359.282547	CPD51149	8.19657544	1278.87577
CPD11424	22.2154727	280.247211	CPD30531	21.4917373	418.326768	CPD51155	8.19843125	1033.89248
CPD11425	22.2236304	794.489616	CPD30534	21.5294059	202.16989	CPD51157	8.20299848	880.248269
CPD11429	22.5619359	828.497888	CPD30538	21.5236876	764.600231	CPD51158	8.19937615	1055.29625
CPD1143	1.1614441	443.15958	CPD30539	21.5202223	784.621789	CPD5116	33.202212	238.21161
CPD11432	22.4337992	608.264057	CPD30542	21.5267273	668.282909	CPD51165	8.20320787	879.7476
CPD11433	22.5557461	675.460908	CPD30543	21.5460002	1057.57826	CPD51166	8.20447322	879.914888
CPD1144	1.1411938	572.10442	CPD30544	21.5330589	746.611741	CPD51168	8.1990741	1075.29607
CPD11441	22.6606775	559.3757	CPD3055	17.852178	354.2049	CPD51169	8.2212839	717.33401
CPD11444	22.6978636	556.396228	CPD30550	21.6157682	145.109965	CPD51171	8.20399388	879.413265
CPD11447	22.7527333	597.448709	CPD30552	21.5393333	854.604567	CPD51174	8.20418918	1055.49766
CPD11450	22.8351054	593.899875	CPD30553	21.5426252	387.310687	CPD51176	8.20611591	1055.89805
CPD11455	22.9674316	622.277271	CPD30554	21.5389473	726.586542	CPD51177	8.19412939	912.101964
CPD11457	23.1577489	440.124167	CPD30563	21.54275	405.324535	CPD51178	8.20583666	889.923386
CPD1146	1.1752631	283.09521	CPD30564	21.6617401	708.593507	CPD51179	8.22362813	889.587843
CPD11460	22.9953451	363.326204	CPD30567	21.5445	772.6291	CPD5118	33.175437	201.02488
CPD11465	22.9996384	700.310464	CPD30569	21.5439999	635.275079	CPD51180	8.19281781	896.081818
CPD11467	23.1704464	926.572425	CPD30571	21.5535455	1183.81324	CPD51181	8.22208907	994.856928
CPD1147	1.1597715	117.0789	CPD30573	21.54915	646.562925	CPD51184	8.17600046	890.256887
CPD11471	23.1484086	568.547624	CPD30576	21.5565497	389.46293	CPD51186	8.19317365	869.086664
CPD11473	23.1389923	563.045675	CPD30579	21.532621	367.309006	CPD51187	8.2401957	843.22161
CPD1148	1.1617929	357.12647	CPD3058	17.951433	372.24523	CPD51188	8.19752822	868.920508
CPD11484	23.2303967	610.27896	CPD30580	21.5609338	449.290647	CPD51189	8.19817701	890.091082
CPD11485	23.227317	787.564828	CPD30581	21.5632255	622.2644	CPD5119	33.1985	187.01208
CPD1150	1.1398557	160.08276	CPD30582	21.5583851	1227.77676	CPD51191	8.21593856	1255.33382
CPD11501	23.7241534	523.408088	CPD30583	21.5623001	223.14406	CPD51194	8.23107406	995.456836
CPD11507	23.6194717	587.885546	CPD30585	21.6038398	325.298425	CPD51196	8.24147727	826.724606
CPD1151	1.171771	260.0325	CPD3059	17.972253	390.25827	CPD5120	33.211835	260.88206
CPD11512	23.6968322	930.910132	CPD30591	21.6375715	706.556871	CPD51201	8.22870088	821.55916
CPD11519	23.8102666	484.337737	CPD30592	21.617823	583.420506	CPD51203	8.23068643	1230.09234
CPD1152	1.1555589	331.12645	CPD30594	21.6570001	347.28328	CPD51205	8.21249401	896.243692
CPD11523	24.0263957	600.416854	CPD30597	21.662923	369.282554	CPD51206	8.25023936	994.658912
CPD11527	24.0868165	850.569493	CPD30598	21.7263616	754.269686	CPD51207	8.2022899	1055.69656
CPD1153	1.1623998	765.05976	CPD30599	21.676583	310.244603	CPD51208	8.23095	836.89067
CPD11533	24.3384316	316.294349	CPD3060	18.088926	317.2343	CPD51209	8.20717644	889.75612
CPD11536	24.9771638	445.352392	CPD30602	21.6923341	519.391367	CPD5121	33.351639	416.83861
CPD1154	1.1357329	457.17711	CPD30604	21.6484785	684.278431	CPD51210	8.22591304	1011.66042
CPD11545	24.8991137	605.421663	CPD30607	21.7317779	760.627633	CPD51213	8.19227396	1034.09401
CPD1155	1.2393902	137.04799	CPD30609	21.7369001	722.614555	CPD51214	8.2276145	1230.59252
CPD11553	25.0835235	606.734557	CPD3061	17.942452	446.22319	CPD51216	8.23420259	1003.66886
CPD1156	1.2648131	532.02657	CPD30611	21.7356363	766.568345	CPD51218	8.19899818	912.76731
CPD1158	1.171425	146.02163	CPD30614	21.7620005	416.343355	CPD5122	33.176191	220.96657
CPD11585	26.0213764	731.788925	CPD30619	21.7582223	1109.8865	CPD51220	8.22477965	1254.58403
CPD1159	1.1717098	267.04561	CPD3062	17.959204	483.37663	CPD51221	8.23225966	837.056884
CPD1160	1.2072168	123.03072	CPD30621	21.7663333	758.407133	CPD51224	8.23169893	1004.06812
CPD1161	1.1611426	307.08614	CPD30625	21.6715281	708.59478	CPD51225	8.20399843	1319.62135
CPD11611	26.4777296	795.581248	CPD30628	21.7681578	754.578153	CPD51226	8.23335047	984.07562
CPD11612	26.4660341	800.540027	CPD3063	17.876884	495.33239	CPD51227	8.23459199	836.39133
CPD11614	26.208701	950.442212	CPD30630	21.7702353	217.142418	CPD51228	8.23410062	984.474965
CPD1162	1.1586877	417.13246	CPD30631	21.7685556	355.309089	CPD51229	8.2333545	836.723735
CPD11625	27.1507788	796.581653	CPD30633	21.7655	634.56273	CPD51230	8.23389869	820.063575
CPD11631	26.957433	616.4916	CPD30634	21.7707855	1215.77505	CPD51231	8.26479738	1068.10342
CPD11634	27.5439728	387.384033	CPD30636	21.7642146	1171.81371	CPD51232	8.23194285	702.911526
CPD1164	1.1541945	598.06032	CPD3064	17.952014	295.25095	CPD51233	8.23458563	1003.46839
CPD11646	27.8757895	556.086937	CPD30642	21.876375	748.62945	CPD51234	8.23314473	984.275235
CPD1165	1.1670508	128.01145	CPD30643	21.7904155	489.3741	CPD51235	8.22510828	1230.34327
CPD11651	28.5267818	858.593685	CPD30644	21.8319843	415.306442	CPD51236	8.22700517	1254.83445
CPD1167	1.16575	117.0244	CPD30647	21.8085671	478.824925	CPD51237	8.22705366	703.194911
CPD11671	28.595172	683.5486	CPD30648	21.8088	385.299015	CPD51238	8.23035098	703.339016
CPD11683	28.9669474	612.781221	CPD3065	17.856337	335.24303	CPD5124	33.193021	111.98075
CPD11684	28.9864634	613.002821	CPD30650	21.8199999	444.343671	CPD51240	8.25088983	1003.86882
CPD11687	29.0668986	798.75656	CPD30652	21.8692313	682.296238	CPD51241	8.23237826	820.562356
CPD11688	29.0869976	864.496747	CPD30656	21.8577858	455.403086	CPD51242	8.23498444	703.624953

CPD1169	1.1541591	327.12231	CPD30658	21.8632105	397.319463	CPD51243	8.23389739	820.229715
CPD11690	29.7550245	753.519059	CPD30659	21.8419447	370.25908	CPD51244	8.23394654	820.89528
CPD11691	29.4290801	666.429524	CPD3066	17.999742	672.31559	CPD51246	8.23151642	836.557184
CPD11693	29.4783677	715.552917	CPD30661	21.8782499	738.49185	CPD51247	8.22600176	829.3842
CPD1170	1.152426	266.09026	CPD30663	21.9109501	829.67563	CPD51248	8.23330272	1004.268
CPD11700	30.6829292	488.419635	CPD30665	21.9223999	857.555015	CPD51249	8.24477093	992.669665
CPD11701	30.8413064	746.504082	CPD30666	21.9236991	753.61943	CPD51250	8.23062985	1230.84303
CPD1171	1.1750907	144.04239	CPD30668	21.9284543	142.099382	CPD51251	8.23353998	984.675
CPD11716	31.4960072	809.569925	CPD3067	18.025672	439.35051	CPD51252	8.2288916	1255.08375
CPD11718	31.4962795	976.470842	CPD30670	21.9289999	512.332026	CPD51253	8.23320349	1004.46784
CPD1172	1.1664553	98.034352	CPD30673	22.0370979	456.359018	CPD51254	8.2339511	837.223421
CPD11727	32.3408127	798.878581	CPD30675	21.9322632	496.357442	CPD51255	8.23354198	820.39583
CPD11728	32.3474148	793.811463	CPD30676	21.9311112	537.381767	CPD51258	8.2300558	820.729316
CPD1173	1.1632086	214.13074	CPD30678	21.9307896	970.720742	CPD51259	8.23235427	984.875055
CPD1174	1.1603335	753.09478	CPD30680	21.93565	608.26349	CPD51260	8.23885935	992.869579
CPD11740	33.074	117.080012	CPD30682	21.9558947	741.619905	CPD51261	8.22654222	821.723136
CPD11751	33.1231252	479.842125	CPD30684	21.9585383	1164.63508	CPD51262	8.27156765	819.898092
CPD11753	33.105755	235.188655	CPD30687	21.969688	622.559663	CPD51263	8.23135423	702.769429
CPD11758	33.1349981	201.928136	CPD30689	21.992268	681.243136	CPD51266	8.23027421	836.225072
CPD1176	1.1662237	245.05475	CPD3069	18.020257	444.30553	CPD51268	8.23432069	703.053784
CPD11763	33.1156387	138.984145	CPD30690	21.9938995	1023.57118	CPD51270	8.33860625	1243.31387
CPD11767	33.1273002	523.8237	CPD30692	22.0252442	867.691121	CPD51272	8.24384247	992.269821
CPD11768	33.1279431	242.95302	CPD30693	22.0633539	660.575374	CPD51273	8.23627775	827.059294
CPD1177	1.163325	586.09216	CPD30695	21.9583787	792.59095	CPD51277	8.22724066	995.05784
CPD11774	33.1294585	577.795163	CPD30698	21.9975999	1083.58903	CPD51279	8.30794288	921.7557
CPD11776	33.2178268	114.952813	CPD30702	22.0071335	1253.79074	CPD5128	33.202112	98.026207
CPD1178	1.1607333	772.20189	CPD30704	22.0119377	455.249312	CPD51280	8.23983434	992.070475
CPD11782	33.1326216	429.863387	CPD30705	22.0107143	830.609764	CPD51282	8.25599337	907.261192
CPD11789	33.1428998	271.94811	CPD30707	21.9985	847.604767	CPD51283	8.24832787	993.0711
CPD1179	1.47065	176.03666	CPD30710	22.0081	846.57618	CPD51284	8.34025245	1038.69785
CPD11791	33.1339999	246.941971	CPD30711	22.02757	672.566257	CPD51285	8.24033635	826.892072
CPD11796	33.1547515	289.927785	CPD30713	22.0170501	437.288685	CPD51289	8.24332621	827.391922
CPD1180	1.174795	420.04083	CPD30714	22.0142141	416.487079	CPD5129	33.214522	137.96625
CPD11800	33.1889445	239.874378	CPD30715	21.9946717	393.32509	CPD51290	8.2108189	1088.31082
CPD11802	33.1898234	300.126924	CPD30716	21.9995733	748.63075	CPD51291	8.2816929	422.118546
CPD11805	33.1723175	277.906825	CPD30722	22.0829001	1067.5767	CPD51292	8.25699923	1088.51417
CPD11809	33.1833399	89.1061875	CPD30725	22.1224736	648.575774	CPD51295	8.2460034	843.719944
CPD1181	1.1735542	227.04304	CPD30728	22.294965	377.292731	CPD51296	8.25084709	906.9286
CPD11810	33.4876096	196.996824	CPD30729	22.1110883	660.574678	CPD51298	8.26524786	907.428175
CPD11816	33.2808647	149.971837	CPD3073	18.104784	395.32469	CPD51299	8.23386726	1067.7041
CPD11817	33.1536022	142.952281	CPD30730	22.2820016	877.673593	CPD5130	33.23631	191.03753
CPD1182	1.1556702	240.14673	CPD30732	22.2008355	736.630244	CPD51300	8.26153638	907.096146
CPD11822	33.1729918	60.0005895	CPD30733	22.1237	774.646435	CPD51301	8.24754662	827.22568
CPD11824	33.224881	191.037967	CPD30736	22.1280827	774.886233	CPD51302	8.26538171	1067.50006
CPD1183	1.1825749	416.14976	CPD30737	22.1275833	818.600467	CPD51303	8.240368	992.470281
CPD11830	33.4626497	89.0046609	CPD3074	18.094192	614.39716	CPD51304	8.23798381	1067.90531
CPD11838	33.4130418	114.955375	CPD30743	22.0565753	668.28375	CPD51306	8.25350355	1088.91686
CPD1184	1.1597146	463.16599	CPD30744	22.1334002	784.98538	CPD51307	8.24669596	843.38565
CPD11841	33.3615111	326.909045	CPD3075	18.102659	400.27958	CPD51308	8.27474119	1088.71429
CPD11847	33.4467367	324.171063	CPD30754	22.1352857	1071.58795	CPD51309	8.21300505	906.763764
CPD11849	33.4127444	91.998	CPD30755	22.1378885	1241.79058	CPD51311	8.28343178	854.413758
CPD11853	33.4807251	126.958182	CPD30757	22.1423125	806.610056	CPD51312	8.28026498	861.084745
CPD11857	33.4489339	73.9814625	CPD3076	18.032574	390.25787	CPD51313	8.28019708	340.130785
CPD11858	33.5598893	97.09218	CPD30760	21.6372973	767.304756	CPD51316	8.27136634	889.415433
CPD1186	1.1611033	135.15791	CPD30761	22.1619593	381.3248	CPD5132	33.253198	132.96488
CPD11864	33.6090439	102.005567	CPD30764	22.1618135	764.512	CPD51321	8.29354576	280.079882
CPD11877	33.5211093	298.874893	CPD30767	22.2236668	452.350043	CPD51324	8.27190702	1067.29844
CPD1188	1.2405568	264.08056	CPD30768	22.0947856	690.264446	CPD51326	8.29289779	854.24797
CPD11880	33.6207113	148.971904	CPD30769	22.1599097	733.574045	CPD5133	33.19029	268.91162
CPD11886	33.529941	69.98465	CPD30773	22.1745297	453.378071	CPD51330	8.34589558	851.247014
CPD1189	1.1686062	452.07177	CPD30775	22.1680822	551.414092	CPD51331	8.34213494	235.008325
CPD1190	1.1622424	654.08814	CPD30777	22.3227778	826.624489	CPD51332	8.32054746	168.042439
CPD1191	1.1692588	174.0163	CPD3078	18.12045	617.41887	CPD51335	8.32134009	227.02234
CPD1193	1.1713571	269.09505	CPD30780	22.1983636	1051.56233	CPD51337	8.29531892	851.07935
CPD1195	1.1714346	406.02858	CPD30782	22.1174985	867.69197	CPD51338	8.37852065	863.587041
CPD1196	1.1614828	145.08189	CPD30784	22.3544726	911.646644	CPD5134	33.165478	189.96385
CPD11961	1.00428161	520.911317	CPD30785	22.2642225	1039.56272	CPD51340	8.37948322	740.359117

CPD1197	1.1482798	343.09473	CPD30787	22.2455737	855.693068	CPD51341	8.36457177	860.917367
CPD1198	1.1691738	193.1079	CPD30788	22.2547777	899.656367	CPD51343	8.39704468	740.076445
CPD1200	1.165094	394.06242	CPD30789	22.4275963	456.360775	CPD51345	8.36131651	454.237069
CPD1201	1.1783427	70.005954	CPD3079	18.028398	372.24508	CPD51346	8.38060355	512.22586
CPD12013	1.06949711	129.0783	CPD30790	22.3149469	438.351778	CPD5135	33.196469	277.90381
CPD1203	1.1657	358.09838	CPD30792	22.2554998	411.3174	CPD51350	8.37499808	884.753455
CPD12039	1.04787167	462.96676	CPD30795	22.255769	715.533423	CPD51351	8.38322667	863.919527
CPD1204	1.162421	135.05487	CPD30797	22.2596665	450.335225	CPD51353	8.38000777	1061.30419
CPD1205	1.1593584	229.98354	CPD30801	22.2215238	707.548745	CPD51354	8.28285546	858.246587
CPD12060	1.04508335	379.764242	CPD30803	22.2524797	774.555514	CPD51355	8.40864143	1040.49298
CPD1208	1.177694	473.20812	CPD30804	22.3001559	1039.56349	CPD51358	8.44760308	1045.10919
CPD1210	1.1725004	255.07639	CPD30805	22.3302728	715.209245	CPD5136	33.2126	136.96248
CPD1211	1.1591999	373.12878	CPD30806	22.3612179	642.269293	CPD51360	8.3770897	740.229569
CPD1213	1.0935614	129.07601	CPD30807	22.3606039	503.392039	CPD51361	8.42146669	1211.57271
CPD12139	1.0638169	376.904018	CPD30808	22.3529999	1351.46285	CPD51363	8.38374348	863.752818
CPD1214	1.1728634	142.02715	CPD30809	22.246304	736.629061	CPD51367	8.44381482	865.923243
CPD1215	1.3319152	482.06295	CPD30810	22.3612502	674.221975	CPD51368	8.42265194	569.245835
CPD12154	1.06952519	538.071425	CPD30815	22.369975	1294.49673	CPD5137	33.224555	91.017221
CPD1216	1.1715371	188.03276	CPD30816	22.3562495	693.232987	CPD51370	8.41906427	968.8586
CPD1217	1.1570364	589.02813	CPD30817	22.353259	1013.54808	CPD51371	8.41561811	969.267538
CPD1218	1.1604544	162.0544	CPD30818	22.275519	355.308773	CPD51372	8.42312623	413.22525
CPD12180	1.06681238	222.868256	CPD3082	18.068335	295.25107	CPD51375	8.42253085	1211.06938
CPD12181	1.05399864	139.01075	CPD30820	22.3870851	826.626856	CPD51376	8.43163812	969.064794
CPD12184	1.08657149	220.177157	CPD30821	22.3043198	636.25798	CPD51377	8.44385857	822.89215
CPD1219	1.1857548	85.089273	CPD30822	22.41625	1189.57665	CPD51379	8.42589985	797.385545
CPD12190	1.10452364	155.063967	CPD30825	22.4021817	728.305673	CPD5138	33.216684	71.00334
CPD12204	1.14333357	728.09305	CPD30826	22.427695	873.64094	CPD51380	8.14711049	857.745411
CPD12207	1.40003192	502.054794	CPD30827	22.4263162	1132.60945	CPD51381	8.43766044	964.656667
CPD12208	1.1195378	324.032115	CPD3083	18.160998	356.25447	CPD51382	8.42698807	1211.32212
CPD1221	1.1609305	299.1176	CPD30830	22.4320003	889.666019	CPD51383	8.4272485	1196.07926
CPD12210	1.16491791	284.009364	CPD30833	22.4612102	1014.67441	CPD51384	8.42804812	956.66316
CPD12211	1.13941704	319.0334	CPD30834	22.4317224	1104.61501	CPD51385	8.42868684	957.06357
CPD12217	1.30078762	303.143386	CPD30836	22.4726191	1055.69723	CPD51386	8.42749763	810.89462
CPD12218	1.15518161	468.090491	CPD30837	22.43445	829.67831	CPD51388	8.42271953	810.727618
CPD12219	1.18950305	550.034325	CPD30838	22.435857	1489.05424	CPD51389	8.42254639	807.899178
CPD12220	1.06814898	106.034192	CPD3084	18.163233	334.26635	CPD51390	8.42904473	957.462947
CPD12221	1.17408338	408.028583	CPD30840	22.4784259	1028.67904	CPD51391	8.42567248	855.249792
CPD1223	1.1842211	111.99408	CPD30841	22.4285454	829.928736	CPD51394	8.42918821	956.86422
CPD12231	1.16690884	203.076756	CPD30842	22.4536654	1031.6824	CPD51395	8.43246028	1196.32919
CPD12232	1.22957002	404.001829	CPD30843	22.4297499	608.565762	CPD5140	33.358699	959.96094
CPD12234	1.22544474	165.07831	CPD30844	22.4441332	1148.64077	CPD51400	8.43042264	961.292089
CPD12235	1.22358434	295.096307	CPD30849	22.5315993	877.92876	CPD51401	8.42962841	797.886095
CPD12236	1.40115226	361.11276	CPD3085	18.171467	351.29705	CPD51402	8.38432567	827.893244
CPD12237	1.30655763	541.956875	CPD30850	22.5086655	855.694207	CPD51404	8.42600219	810.562013
CPD12248	1.19701763	259.11812	CPD30852	22.4431395	855.944209	CPD51405	8.42584377	807.731774
CPD1225	1.1968403	270.07392	CPD30855	22.4581113	954.74645	CPD51406	8.42788739	957.262855
CPD12252	1.36172185	302.018106	CPD30856	22.4641296	1467.07475	CPD51408	8.42431563	798.053626
CPD12253	1.36247809	52.0115105	CPD30859	22.4550664	915.68138	CPD51409	8.42989103	797.719061
CPD12260	1.44740201	152.99637	CPD3086	18.114079	1040.5744	CPD5141	33.137126	155.97681
CPD1227	1.2096361	179.07078	CPD30864	22.4503125	419.336062	CPD51410	8.43039201	797.220985
CPD12270	1.23763394	224.05788	CPD30865	22.4458671	652.286467	CPD51411	8.43134495	962.091745
CPD12272	1.60249199	199.0256	CPD30867	22.4588573	968.21875	CPD51413	8.42337355	1195.82775
CPD12274	1.52210296	514.075713	CPD30869	22.4777057	497.549686	CPD51414	8.44589711	987.4677
CPD12276	1.60825847	145.110929	CPD3087	18.196257	290.24486	CPD51415	8.42873448	961.691421
CPD1228	1.1636254	671.0076	CPD30871	22.4670631	220.179462	CPD51416	8.42825933	801.076932
CPD12293	1.46458379	336.082176	CPD30872	22.474587	972.992991	CPD51417	8.42868329	962.290863
CPD12294	1.53222641	170.025089	CPD30875	22.4690667	234.16616	CPD51418	8.42144864	813.549611
CPD12298	1.24764819	449.193725	CPD30877	22.463067	893.689267	CPD51419	8.42905094	961.4918
CPD1230	1.2500986	342.11763	CPD30880	22.4616	456.96161	CPD5142	33.228386	169.00235
CPD12303	1.59554571	389.179895	CPD30883	22.467	519.425492	CPD51420	8.42904591	961.891147
CPD12307	1.58623348	148.033327	CPD30884	22.4684003	248.177927	CPD51421	8.42973177	801.409432
CPD12309	2.37669765	73.0496	CPD30885	22.46575	970.724275	CPD51422	8.42957964	802.075279
CPD1231	1.0922086	106.03287	CPD30886	22.2372164	563.417325	CPD51425	8.4282743	797.552483
CPD1232	1.152133	108.0227	CPD30888	22.4676667	1043.66421	CPD51426	8.42659934	810.395
CPD12320	2.10741047	103.099568	CPD30889	22.4691111	972.385645	CPD51427	8.42915751	801.742374
CPD12322	1.48113241	302.153007	CPD30890	22.487085	457.0123	CPD51428	8.41504885	1202.61276
CPD12323	2.07832726	220.09315	CPD30891	22.4796594	202.171208	CPD51429	8.44363315	822.391955

CPD1233	1.182333	97.977844	CPD30893	22.5982125	381.324606	CPD51430	8.42925949	801.575905
CPD12334	1.59991006	174.100662	CPD30896	22.4666677	605.452122	CPD51431	8.48056529	813.711293
CPD12339	1.70492722	259.157284	CPD30897	22.4761056	1092.63154	CPD51432	8.42994691	1201.86336
CPD1234	1.1611847	405.13144	CPD30898	22.4827628	533.419076	CPD51433	8.43974573	972.474183
CPD12346	1.98137865	311.118008	CPD3090	18.21325	192.14928	CPD51434	8.43733248	981.481667
CPD12348	1.80864691	175.02982	CPD30900	22.4564464	438.350593	CPD51435	8.51007377	814.046673
CPD1236	1.1543924	186.09825	CPD30901	22.4722667	200.156193	CPD51436	8.43046993	1202.11343
CPD1237	1.1998794	210.05976	CPD30906	22.4749501	474.371455	CPD51437	8.43094669	1202.3632
CPD12373	3.11462616	208.0918	CPD30908	22.4729504	222.161385	CPD51439	8.42999999	801.909211
CPD1239	1.1694665	355.1482	CPD30909	22.480111	700.309078	CPD51442	8.43171182	1196.57903
CPD12390	4.73838611	293.138162	CPD3091	18.149384	1386.7634	CPD51443	8.43010392	801.243332
CPD1240	1.4851838	168.02945	CPD30911	22.5063853	413.339346	CPD51445	8.33482477	858.41431
CPD1241	1.186647	131.09464	CPD30912	22.5011428	1164.63413	CPD51446	8.42889316	810.23025
CPD1243	1.1764751	437.97374	CPD30913	22.5051104	634.244833	CPD51449	8.37268647	855.584681
CPD12433	6.12792674	373.218883	CPD30915	22.5119999	566.255579	CPD5145	33.243851	105.03624
CPD12436	6.15309369	313.173515	CPD30916	22.5089994	909.695006	CPD51450	8.44572567	1026.70108
CPD12438	6.25476472	340.130869	CPD30918	22.5123573	863.653857	CPD51451	8.44714117	855.751393
CPD1244	1.2747653	305.1363	CPD30919	22.5925635	1056.69257	CPD51452	8.43725791	1026.89954
CPD12443	6.71109546	368.110154	CPD30921	22.5355001	361.298405	CPD51454	8.45182193	976.452671
CPD12448	6.44884296	403.14737	CPD30923	22.5394493	768.63171	CPD51456	8.44099623	973.074454
CPD1245	1.1617362	385.12576	CPD30929	22.5343749	1065.57997	CPD51457	8.446397	987.27047
CPD12453	6.42454638	378.09733	CPD3093	18.122343	295.25102	CPD51458	8.45451579	976.851071
CPD12455	6.52998747	357.1977	CPD30930	22.5402665	1097.60537	CPD51459	8.45373776	976.650219
CPD12457	6.5366771	288.11858	CPD30931	22.542375	882.966012	CPD5146	33.222711	505.7742
CPD12459	6.63195948	244.176789	CPD30935	22.5471763	452.389347	CPD51461	8.45119943	260.16248
CPD1246	1.1735004	367.14653	CPD30938	22.5507501	445.479633	CPD51467	8.43380228	822.726525
CPD12464	6.94789437	218.013522	CPD3094	18.28643	456.26972	CPD51468	8.44589628	813.881436
CPD12467	6.9511428	192.077953	CPD30940	22.5466	881.710845	CPD51469	8.77487086	990.842689
CPD12469	7.02991586	359.201992	CPD30942	22.5575789	468.396689	CPD51471	8.43770358	733.21614
CPD12475	7.15953465	337.08114	CPD30947	22.6143172	390.316964	CPD51472	8.45865545	977.0505
CPD12476	7.2662668	370.194532	CPD3095	18.28387	434.28761	CPD5148	33.265178	88.001588
CPD1248	1.1793394	339.114	CPD30950	22.5488124	466.388875	CPD51480	8.45980085	1220.81069
CPD12480	7.28168974	424.07247	CPD30951	22.5593333	429.761767	CPD51483	8.4426896	855.91721
CPD12487	7.49404124	509.172012	CPD30952	22.5582499	407.34052	CPD51484	8.45088753	973.850033
CPD1249	1.1685225	173.0687	CPD30954	22.5571176	430.506076	CPD51487	8.53459229	1036.28141
CPD12490	7.64055464	767.159625	CPD30957	22.5504692	874.62038	CPD51488	8.46445624	1220.56565
CPD12492	7.63534834	759.173	CPD3096	18.3049	644.2855	CPD51489	8.47347281	1260.33307
CPD12497	7.89500396	502.29386	CPD30960	22.5569444	858.643306	CPD5149	33.280557	153.96499
CPD1250	1.1842773	369.16315	CPD30961	22.6872667	1012.65313	CPD51490	8.45263726	855.41765
CPD12507	8.09685428	536.285932	CPD30962	22.5619999	389.327122	CPD51491	8.52610295	805.208305
CPD12509	8.18914847	197.028207	CPD30963	22.5526467	1354.99242	CPD51492	8.48769813	1098.28544
CPD1251	1.177744	323.11913	CPD30965	22.5633997	925.93388	CPD51493	8.56806825	1217.31299
CPD12510	8.2013872	405.015062	CPD30966	22.4634729	938.593436	CPD51498	8.46542919	996.861514
CPD12513	8.35564573	537.260206	CPD30967	22.5501417	1095.74171	CPD51502	8.51726498	1237.53806
CPD12514	8.31340447	664.18705	CPD30969	22.557471	941.644882	CPD51504	8.52249527	1212.79401
CPD12516	8.1991997	539.2339	CPD3097	18.307607	310.1573	CPD51507	8.51486756	1237.7862
CPD1252	1.1622225	413.18081	CPD30970	22.5562633	925.674205	CPD51508	8.52099826	970.835606
CPD12520	8.48792263	610.154561	CPD30973	22.5612002	927.03958	CPD51510	8.5019222	808.700993
CPD12521	8.37559328	450.210925	CPD30979	22.6331116	491.396079	CPD51511	8.51889443	970.635805
CPD12523	8.42875288	354.143138	CPD3098	18.10493	1040.075	CPD51513	8.49762242	990.032562
CPD12524	8.47026182	375.072067	CPD30980	22.6048123	729.615356	CPD51514	8.51372658	1237.2899
CPD1253	1.1796037	353.13212	CPD30982	22.6168	843.94521	CPD51515	8.53268255	985.444253
CPD12533	8.96687034	330.15398	CPD30983	22.4978021	456.869033	CPD51516	8.52020043	1213.04395
CPD12536	8.71608765	353.16925	CPD30984	22.6747635	776.660421	CPD51517	8.51847766	1213.54413
CPD12540	8.76603399	826.375727	CPD30986	22.7162245	843.693763	CPD51519	8.52074479	970.436865
CPD12542	8.73791016	334.152309	CPD30988	22.5461682	200.155355	CPD5152	33.295597	92.999586
CPD1255	1.19514	88.016844	CPD3099	18.425469	324.31157	CPD51520	8.53375421	1295.34646
CPD12551	8.87670716	286.134679	CPD30990	22.5408504	1028.67829	CPD51522	8.53024118	804.874168
CPD12556	9.3484335	230.043333	CPD30991	22.6882148	887.656811	CPD51524	8.49757902	863.91231
CPD12558	9.18208651	472.193258	CPD30993	22.5738147	1055.69716	CPD51525	8.52810239	804.707558
CPD1256	1.1824637	257.2437	CPD30994	22.7450464	369.3245	CPD51526	8.53669953	1232.05056
CPD12567	9.29344911	493.109245	CPD30999	22.5941319	1008.68756	CPD51527	8.52119519	1213.29364
CPD1257	1.2584726	163.06623	CPD31004	22.5855351	855.69377	CPD51528	8.52193341	1238.0376
CPD12571	9.57178598	766.241617	CPD31005	22.4898697	899.657344	CPD5153	33.263122	193.96767
CPD1258	1.6883733	186.00065	CPD31007	22.6729977	915.64085	CPD51531	8.53785154	1231.55119
CPD12580	9.52494494	970.240549	CPD31008	22.6351863	788.661632	CPD51532	8.55477448	1045.10976
CPD12589	9.77075034	590.138813	CPD3101	18.583749	220.11403	CPD51533	8.50927081	808.865509

CPD1259	1.2110007	434.18847	CPD31010	22.6655384	1194.64685	CPD51536	8.53859723	966.448015
CPD12591	9.80600217	305.089715	CPD31012	22.6831464	750.6447	CPD51537	8.5393989	1232.30079
CPD12592	9.82807982	210.161047	CPD31015	22.6996667	698.294761	CPD51538	8.53739584	1231.80043
CPD12595	10.0255751	1574.44779	CPD31017	22.630779	381.324609	CPD51540	8.53915118	966.047975
CPD12597	10.0357006	987.75251	CPD3102	18.494034	398.34468	CPD51541	8.49871166	1306.63436
CPD1260	1.2853745	225.06405	CPD31020	22.6212553	564.346606	CPD51542	8.53465999	1306.13594
CPD12605	10.4361516	584.150357	CPD31021	22.7401415	324.261858	CPD51544	8.53780019	985.842255
CPD12611	11.3262877	614.243671	CPD31022	22.7279926	403.3064	CPD51545	8.53929554	965.84814
CPD12614	11.7485777	208.069906	CPD31023	22.7378114	665.23355	CPD51546	8.54055201	1207.30916
CPD12618	12.5154996	486.208036	CPD31029	22.7486429	362.286364	CPD51547	8.5359982	805.375862
CPD1262	1.1581047	257.10355	CPD31030	22.7500021	425.279868	CPD51548	8.5194128	863.7416
CPD1263	1.1938566	134.02362	CPD31034	22.7840001	1021.58939	CPD51549	8.54467124	986.043094
CPD12637	15.0100749	242.202025	CPD31035	22.7921111	1088.62384	CPD5155	33.238702	91.999784
CPD12639	14.9736272	687.354625	CPD31038	22.7937221	995.57495	CPD51550	8.54099806	1207.55888
CPD1264	1.1898773	425.18545	CPD3104	18.449568	225.20883	CPD51551	8.52655216	805.041345
CPD12643	14.99564	369.142364	CPD31041	22.8071821	469.415191	CPD51555	8.54005136	1207.80875
CPD12645	15.0438151	249.992219	CPD31042	22.8072777	1184.53549	CPD51556	8.68536102	1055.87986
CPD12648	15.2008144	352.424782	CPD31044	22.9556266	730.32021	CPD51557	8.53754507	965.648275
CPD1265	1.1893343	157.07248	CPD31047	22.8641715	750.64447	CPD51558	8.53632039	870.925262
CPD12651	15.1981152	1048.3234	CPD3105	18.44914	208.17777	CPD51559	8.54155088	1207.05881
CPD12652	15.2248081	315.277797	CPD31050	22.8863001	983.573595	CPD5156	33.257594	148.94778
CPD12653	15.2308564	991.640486	CPD31052	22.7647912	887.656854	CPD51560	8.49231794	1045.31066
CPD12656	15.3172781	208.179677	CPD31054	23.0721689	698.294414	CPD51561	8.7855834	1055.67445
CPD12658	15.3925463	293.234373	CPD31055	22.8171954	776.658167	CPD51562	8.53304772	985.64301
CPD1266	1.205635	244.00031	CPD31056	22.7902774	843.693579	CPD51565	8.52565361	1036.48042
CPD12663	15.7211742	292.201716	CPD31059	22.8847493	387.30925	CPD51567	8.48989747	1044.91157
CPD12668	15.7588145	336.229527	CPD31062	22.9151246	1057.57506	CPD51569	8.55899825	1054.25751
CPD1267	1.5001888	219.11303	CPD31064	22.9081322	798.598727	CPD51570	8.54253548	1306.38357
CPD12675	16.3613853	506.26343	CPD3107	18.489186	326.22435	CPD51571	8.55837439	874.89235
CPD1268	1.1932622	239.06736	CPD31070	22.9078999	782.60587	CPD51573	8.53942695	1036.67757
CPD12687	16.6146348	536.579483	CPD31071	22.915375	430.370513	CPD51574	8.53492865	1036.07886
CPD1269	1.1903888	220.05532	CPD31076	22.9228743	351.310556	CPD51575	8.56130758	1053.45799
CPD12692	17.2920763	315.310375	CPD31077	22.912	849.680405	CPD51576	8.49041298	870.758073
CPD12702	17.7454581	408.283453	CPD31080	22.8232137	369.324656	CPD51577	8.56139612	1119.49475
CPD12703	17.7431092	219.124222	CPD31083	22.9250579	369.91097	CPD51578	8.54799941	674.278374
CPD12709	18.1245481	582.404955	CPD31084	22.9178768	1009.59048	CPD5158	33.266021	152.94277
CPD12711	18.2953064	295.250746	CPD31085	22.914059	938.725047	CPD51582	8.56254564	290.172611
CPD12723	18.5304188	432.226944	CPD31086	22.928704	414.378447	CPD51588	8.48733584	863.578264
CPD1273	1.1911536	560.03472	CPD31087	22.9188421	190.169979	CPD51589	8.53198974	1035.87856
CPD12730	19.3656492	251.2163	CPD3109	18.53813	446.24251	CPD51590	8.51758364	1294.8485
CPD12734	19.0321572	404.2394	CPD31090	22.9147999	440.365415	CPD51592	8.56838811	1336.07626
CPD12736	19.6964058	442.2306	CPD31091	22.9168999	624.578365	CPD51593	8.56849636	1137.27636
CPD12737	19.6185136	334.2516	CPD3110	18.538422	662.36852	CPD51594	8.56852553	1048.66912
CPD12746	19.9895967	746.419438	CPD31100	22.9247334	999.57338	CPD51595	8.55601942	863.410245
CPD1275	1.217713	207.05564	CPD31104	23.0002041	891.681175	CPD51596	8.51182751	871.256378
CPD12753	20.3118908	152.084283	CPD31106	22.9378001	808.623673	CPD51598	8.58170473	884.1125
CPD1276	1.2149456	492.03175	CPD3111	18.570256	363.31353	CPD5160	33.300686	278.9024
CPD12763	20.316782	227.225086	CPD31110	22.9312497	871.652758	CPD51600	8.56247041	1053.65673
CPD1277	1.173113	504.01129	CPD31111	22.9262494	480.358367	CPD51601	8.56990075	330.165
CPD12783	20.8573712	158.118659	CPD31112	22.9598749	764.662541	CPD51602	8.55905269	325.2092
CPD12791	21.7730735	800.467644	CPD31115	22.954125	846.634163	CPD51604	8.56634202	1311.83316
CPD12798	21.451512	676.437273	CPD31116	22.9474276	489.397843	CPD51605	8.57169871	1068.66348
CPD1280	1.5379788	484.02654	CPD31117	22.9590908	802.667664	CPD51606	8.5782064	943.74063
CPD12803	21.5466877	582.704245	CPD3112	18.590833	329.32753	CPD51607	8.56413283	1415.85583
CPD12804	21.4773488	155.130296	CPD31120	22.99678	395.340245	CPD51609	8.56688875	1415.35474
CPD1281	1.3005539	283.11698	CPD31121	22.9711499	433.35059	CPD5161	33.233848	123.95573
CPD12810	21.6001127	520.491479	CPD31122	22.9659106	897.668491	CPD51611	8.57940359	851.16537
CPD12811	21.6487224	338.299707	CPD3113	18.594684	202.1679	CPD51616	8.5694932	1049.46696
CPD12825	22.0401508	642.268119	CPD31131	23.0550625	638.27485	CPD51617	8.56873432	1311.08328
CPD12839	22.3241037	227.224536	CPD31136	23.2878771	688.250267	CPD51618	8.57084665	1049.66731
CPD12841	22.4312225	640.288143	CPD31137	23.0402144	1212.5287	CPD51619	8.56920085	1049.2671
CPD1285	1.2781972	74.068316	CPD31138	23.0297784	427.364322	CPD5162	33.413701	107.95485
CPD12854	22.6974579	814.484789	CPD3114	18.662326	364.12891	CPD51620	8.56795191	1311.3327
CPD12857	22.694637	554.168067	CPD31140	23.0528946	1011.56826	CPD51621	8.5677392	1335.57488
CPD1286	1.5631689	519.97424	CPD31142	23.0699284	1286.53006	CPD51622	8.56533352	874.558873
CPD12861	22.7430064	537.370937	CPD31143	23.0634031	650.594689	CPD51624	8.56921577	1317.07035
CPD12867	22.9626861	623.277825	CPD31144	23.2435464	730.320956	CPD51625	8.68909124	1039.49574

CPD1287	1.1979961	190.01747	CPD31145	23.0911124	755.629778	CPD51626	8.57017146	1068.86144
CPD12877	23.1184732	882.56535	CPD3115	18.589218	198.0871	CPD51627	8.5687018	1311.58236
CPD12878	23.1734794	565.878396	CPD31152	23.105028	764.661428	CPD51628	8.5694035	1048.86784
CPD12881	23.1524762	565.048692	CPD31156	22.9350182	720.278722	CPD51630	8.570245	1049.0675
CPD1289	1.2687314	597.97771	CPD3116	18.591531	160.08833	CPD51631	8.5735497	1119.69123
CPD12901	23.5119468	521.854247	CPD31166	23.1802664	676.936579	CPD51632	8.56553265	1069.06262
CPD12902	23.578301	968.571572	CPD31167	23.1704999	676.834828	CPD51633	8.56867486	1335.82484
CPD1291	1.2321736	515.99653	CPD31168	23.1722498	676.611255	CPD51635	8.57363977	1132.68604
CPD1292	1.2662736	234.96486	CPD3117	18.602932	220.11265	CPD51636	8.58700459	477.148475
CPD12923	23.8416679	524.828425	CPD31170	23.1715834	1069.60665	CPD51638	8.57353351	1069.26376
CPD12924	24.0924073	586.449067	CPD31171	23.1631094	869.705413	CPD51639	8.56958267	1132.8859
CPD1293	1.2703577	365.91068	CPD31172	23.1750003	1037.58403	CPD5164	33.323096	94.003897
CPD12940	24.3177708	669.209623	CPD31178	23.2011865	850.611163	CPD51640	8.56878284	1132.48635
CPD12947	24.5477426	713.534844	CPD3118	18.592082	202.099	CPD51641	8.57064852	1132.28605
CPD1297	1.2620858	445.9891	CPD31182	23.2057779	834.641256	CPD51642	8.57042606	1415.10602
CPD12978	25.3346171	457.418194	CPD31185	23.1644329	395.340441	CPD51643	8.56579292	1053.85532
CPD1298	1.287295	379.96932	CPD31190	23.2144136	396.765078	CPD51644	8.56799357	1132.08815
CPD1299	1.2637304	73.052846	CPD31193	23.2490001	856.621881	CPD51646	8.57043754	874.224331
CPD1301	1.2666846	349.93232	CPD31194	23.2172034	674.2729	CPD51647	8.57446981	443.05774
CPD13024	26.4880419	908.54676	CPD31197	23.3678553	638.273974	CPD51648	8.56358829	1415.60731
CPD13027	26.6222098	590.4834	CPD31198	23.2694497	489.3789	CPD5165	33.303271	271.91412
CPD1303	1.3321144	209.05303	CPD3120	18.592311	276.17372	CPD51650	8.58650503	943.575083
CPD13031	26.6747209	882.458421	CPD31202	23.2820007	348.269778	CPD51651	8.57767865	643.117279
CPD13032	26.7836807	665.943474	CPD31203	23.2302873	720.565314	CPD51654	8.57056135	874.064738
CPD13040	27.0605265	366.307937	CPD31208	23.3311769	770.497112	CPD51655	8.56893963	1336.32333
CPD13050	27.7873097	754.4438	CPD31210	23.2816469	439.30478	CPD51656	8.5710085	1068.46994
CPD13067	29.0080869	797.111283	CPD31212	23.3098748	1009.59129	CPD51660	8.59209835	922.26645
CPD1307	1.3796415	238.06477	CPD31215	23.4025784	656.284837	CPD51663	8.56471275	1038.90682
CPD13071	28.9510528	819.581995	CPD31217	23.3119994	455.287175	CPD51664	8.57387624	1215.90092
CPD1308	1.2793394	100.01544	CPD31218	23.3134988	773.568475	CPD51665	8.58294237	168.041926
CPD13089	30.4044463	936.836383	CPD31223	23.2953693	752.303915	CPD51666	8.58341731	435.0773
CPD1310	1.2892257	175.87648	CPD31225	23.4778746	483.428853	CPD51667	8.54797071	262.987438
CPD1311	1.3040208	173.87839	CPD3123	18.705928	617.41881	CPD51668	8.58188622	344.054129
CPD13113	32.1168589	966.567529	CPD31230	23.3752604	386.277514	CPD51669	8.57755507	643.61895
CPD1312	1.3005367	494.00474	CPD31232	23.5157512	427.307293	CPD5167	33.272018	150.94283
CPD13126	33.2009648	84.9348176	CPD31233	23.5555275	495.293507	CPD51671	8.58324788	870.135483
CPD1313	1.2939327	447.89659	CPD31235	23.4646501	778.67716	CPD51673	8.57558149	230.042194
CPD13135	33.13047	330.8989	CPD3124	18.727695	232.18105	CPD51674	8.58324523	416.0953
CPD1314	1.561284	556.10436	CPD31241	23.4775	1065.57647	CPD51675	8.58438087	212.030633
CPD13144	33.1249093	525.82807	CPD31246	23.2398258	790.918615	CPD51676	8.59450707	560.315675
CPD13156	33.132	591.794943	CPD31248	23.4995453	857.707536	CPD51677	8.58145204	886.10895
CPD1316	1.2943742	223.06838	CPD3125	18.668919	364.12878	CPD51679	8.57236221	1317.31869
CPD13175	33.1392772	183.17071	CPD31251	23.4929499	1025.5846	CPD5168	33.273844	71.983824
CPD13176	33.1392277	367.852162	CPD31253	23.5166928	767.482811	CPD51680	8.58556325	384.07015
CPD1318	1.2638196	160.03701	CPD31258	23.5081112	810.642078	CPD51681	8.58044321	433.381264
CPD13180	33.1739308	77.0156353	CPD31259	23.501182	423.303382	CPD51682	8.5682496	874.393419
CPD13182	33.2336553	291.918286	CPD3126	18.785545	317.23441	CPD51683	8.58879277	511.03907
CPD13186	33.1852612	238.211271	CPD31260	23.5075781	826.625353	CPD51684	8.58120245	433.303432
CPD1319	1.4136433	348.02121	CPD31262	23.5017698	767.259085	CPD51685	8.5823148	881.104437
CPD13194	33.2619052	134.962522	CPD31263	23.5051543	830.662508	CPD51686	8.59199603	300.049253
CPD1320	1.3344197	183.06301	CPD31266	23.5132935	428.391747	CPD51688	8.58208546	856.144085
CPD13204	33.1120731	179.018869	CPD31268	23.5137696	425.354323	CPD5169	33.280741	72.982123
CPD13211	33.4380056	208.938562	CPD3127	18.80224	673.49391	CPD51690	8.58073338	227.021942
CPD13212	33.4715484	111.967316	CPD31271	23.51285	383.340345	CPD51692	8.57971271	438.259414
CPD1323	1.3199313	135.92347	CPD31275	23.518579	444.374011	CPD51693	8.58361932	890.090831
CPD13249	33.5503094	224.021217	CPD31277	23.5134537	383.939718	CPD51694	8.57424571	470.0163
CPD1327	1.2707001	167.01994	CPD31279	23.5206498	664.610355	CPD51696	8.59595508	308.03705
CPD1330	1.3500227	389.88328	CPD3128	18.858898	1101.817	CPD51697	8.57612537	635.127862
CPD1331	1.3705361	253.95967	CPD31280	23.4657375	773.541836	CPD51698	8.58540398	158.020365
CPD1335	1.3614898	294.98862	CPD31282	23.5349444	848.646822	CPD51702	8.59855092	632.04595
CPD13354	1.02218441	521.903216	CPD31283	23.542639	804.691278	CPD51703	8.60768408	865.757563
CPD1336	1.4216717	271.99782	CPD31285	23.5416999	421.344385	CPD51705	8.60146587	389.053358
CPD1338	1.408668	105.04233	CPD31287	23.5439999	459.314412	CPD51707	8.58675867	922.432175
CPD13388	1.00599931	406.940935	CPD31288	23.5529015	1083.59284	CPD51708	8.5915816	866.257531
CPD1339	1.3391671	351.93617	CPD3129	18.85848	1469.0894	CPD51709	8.60086966	551.202844
CPD1341	1.422385	168.02725	CPD31290	23.5288123	686.576025	CPD5171	33.279881	73.98157
CPD1342	1.4474899	142.02937	CPD31295	23.6220198	656.828686	CPD51711	8.58235135	866.42135

CPD1343	1.5513005	135.05703	CPD31299	23.6842665	1120.61293	CPD51714	8.60730553	602.089715
CPD1344	1.0053681	443.96855	CPD31304	23.624071	987.605075	CPD51715	8.61610698	865.924122
CPD1345	1.4007577	234.96451	CPD31307	23.6092009	959.57583	CPD51716	8.60089248	742.36213
CPD13458	1.09820616	1231.11553	CPD31308	23.6255714	833.399943	CPD51717	8.58733324	866.090233
CPD1347	1.4064533	119.02054	CPD31309	23.6325711	636.207371	CPD51719	8.61422468	742.078367
CPD13474	1.05000002	582.78078	CPD3131	18.880446	686.29428	CPD51720	8.6283413	1088.07481
CPD13479	1.03386394	368.981013	CPD31312	23.5919951	563.2801	CPD51724	8.70828237	1066.26807
CPD1351	1.4117861	387.98087	CPD31313	23.6325365	715.334108	CPD51727	8.70195052	1054.87919
CPD1352	1.2371167	203.00658	CPD31316	23.635933	1276.50812	CPD51728	8.70094191	879.565206
CPD1353	1.3214578	74.072085	CPD31319	23.6455455	336.298118	CPD51729	8.7022449	879.232365
CPD13534	1.06584606	218.149456	CPD31323	23.5579646	483.428487	CPD51730	8.70283222	879.066522
CPD13535	1.43248355	169.054536	CPD31324	23.7108381	836.654712	CPD51732	8.70326621	1055.07761
CPD13544	1.09628572	581.975279	CPD31326	23.8643977	1120.61295	CPD51733	8.69994287	879.399426
CPD13546	1.12899995	497.92215	CPD31327	23.7118226	830.705565	CPD51735	8.70067577	1055.27794
CPD1355	1.3402234	426.02661	CPD31328	23.7104704	690.624412	CPD51736	8.7002121	1055.47795
CPD13550	1.13492283	684.109977	CPD31329	23.7406619	792.693715	CPD51737	8.69724372	879.732969
CPD13559	1.53155381	198.020114	CPD3133	18.867092	659.41734	CPD51738	8.70431617	1055.67912
CPD1356	1.448561	165.00657	CPD31330	23.8331365	453.320363	CPD51739	9.13055469	1210.80711
CPD13561	1.12193597	140.0123	CPD31335	23.7664778	1029.58303	CPD51741	8.70758136	1318.59525
CPD1357	1.4187047	61.055065	CPD31337	23.7357331	979.579373	CPD51742	8.70558344	1318.84534
CPD13573	1.24309876	120.114065	CPD31339	23.7444995	1109.60204	CPD51745	8.70780511	1319.34681
CPD13575	1.19987951	518.014262	CPD31341	23.682897	655.276327	CPD51746	8.70526802	1319.09715
CPD1358	1.4232368	181.0165	CPD31343	23.7824268	826.677029	CPD51747	8.71359515	1074.47645
CPD13586	1.66938942	256.008271	CPD31344	23.8172693	579.4439	CPD51748	8.68104967	1078.69963
CPD1359	1.4064232	248.03163	CPD31345	23.7592937	1097.60365	CPD51749	8.81185701	1066.07336
CPD13590	1.31146265	462.049522	CPD31348	23.81102	409.842975	CPD51751	8.77814113	998.846178
CPD13593	1.37034833	346.001373	CPD3135	18.959127	312.14167	CPD51753	8.56469885	1066.47371
CPD13598	1.2870648	185.0123	CPD31350	23.7787014	824.71265	CPD51754	8.73049867	1093.68104
CPD1360	1.3153656	403.96405	CPD31351	23.7943865	410.787417	CPD51755	8.75649486	996.4407
CPD13600	1.39408027	227.969306	CPD31352	23.752496	1051.58913	CPD51757	8.73438918	911.735111
CPD13604	1.39817395	243.02821	CPD31354	23.7780001	819.32255	CPD51758	8.74121465	911.23415
CPD13617	1.39725494	144.041011	CPD31355	23.781	447.496143	CPD51759	8.73249457	1094.08044
CPD1362	1.4243925	190.01746	CPD31356	23.7843334	468.4116	CPD51761	8.69010654	1039.10298
CPD13628	1.32143359	230.266273	CPD31360	23.7847	862.67375	CPD51762	8.73320818	911.566405
CPD1363	1.4220731	419.93287	CPD31363	23.7884997	409.356125	CPD51763	8.73466901	1094.28174
CPD13631	1.56676917	357.132208	CPD31365	23.7854496	878.646995	CPD51765	8.7402336	1367.34949
CPD13632	1.55149106	588.070074	CPD31369	23.7913563	552.2779	CPD51767	8.81664686	1065.8741
CPD13633	1.56831231	717.989594	CPD31374	23.8095387	965.604877	CPD51768	8.73806752	1093.48201
CPD13647	1.65031051	279.19985	CPD31377	23.8141332	934.71186	CPD51769	8.73383592	1093.88168
CPD13650	1.19871652	333.022821	CPD31378	23.7955055	454.410689	CPD51771	8.78578588	1252.54206
CPD13652	1.72527835	197.01881	CPD3138	18.980586	585.4435	CPD51775	8.78046191	991.652
CPD13658	1.75367784	324.164917	CPD31381	23.8268719	884.647218	CPD51777	8.77587261	1239.31369
CPD1366	1.4810418	218.02219	CPD31384	23.8111049	1067.59399	CPD51779	8.78521414	999.64235
CPD13670	1.59058913	207.0924	CPD31385	23.8316872	481.411425	CPD5178	33.498753	78.934726
CPD13674	2.03615533	324.139319	CPD31389	23.833444	678.623617	CPD51781	8.7964967	991.451931
CPD1368	1.441414	228.9673	CPD3139	19.36597	299.28095	CPD51782	8.80467391	826.044122
CPD13686	2.70093061	202.130452	CPD31390	23.8419776	876.78685	CPD51783	8.80265926	1239.56319
CPD1370	1.4491424	122.00448	CPD31396	23.8002752	836.651647	CPD51784	8.79546298	991.851709
CPD1371	1.4168936	241.07874	CPD31398	23.9809477	384.34095	CPD51785	8.79131908	992.051387
CPD13710	4.33091853	230.161172	CPD3140	19.047921	853.57345	CPD51788	8.77505756	1064.68846
CPD13725	5.89452079	190.05715	CPD31409	23.9712926	405.32354	CPD51789	8.77817625	1239.81386
CPD13726	6.09354983	289.142373	CPD31412	23.9427786	891.707478	CPD51790	8.79929079	1217.88367
CPD13727	5.83838118	366.08705	CPD31415	23.8456069	453.3205	CPD51791	8.75723816	826.541511
CPD13728	5.85289608	870.08223	CPD31417	23.9697712	589.296262	CPD51793	8.77797241	1078.49022
CPD1373	1.4446239	238.97711	CPD31419	23.9283692	521.3125	CPD51796	8.78569435	999.841878
CPD13735	6.0388429	206.056395	CPD3142	19.216126	672.38268	CPD51799	8.71029557	1219.30689
CPD13737	6.54823484	656.142446	CPD31428	23.9815544	664.431533	CPD5180	33.387307	148.97393
CPD13739	6.4145977	236.10672	CPD31430	23.9972312	482.381008	CPD51800	8.7820818	1034.07274
CPD13741	6.65900391	405.162119	CPD31432	23.9938302	338.3496	CPD51802	8.95187835	994.243767
CPD13754	6.76157961	241.041377	CPD31433	24.0143547	489.413863	CPD51804	8.78932685	1292.59098
CPD1376	1.4181347	285.10638	CPD31434	24.0552196	447.349933	CPD51806	8.73625559	1078.90026
CPD13762	7.4561575	222.106621	CPD31435	24.0376658	1053.61434	CPD51809	8.79077932	819.37115
CPD13766	7.6705191	450.208222	CPD31436	24.0492731	1104.65025	CPD51811	8.77218698	1064.49085
CPD13768	7.51344115	222.108573	CPD31437	24.0538336	426.326633	CPD51812	8.64796968	1064.29202
CPD1377	1.4441235	336.05501	CPD3144	19.07087	541.41787	CPD51816	8.78990484	1052.48219
CPD13777	7.85913295	499.969187	CPD31440	24.1547498	435.37037	CPD51818	9.35843145	1236.80515
CPD1378	1.446623	368.00222	CPD31442	24.1590999	1079.62956	CPD51819	8.77633308	1314.85065

CPD13783	7.93353499	382.121419	CPD31443	24.3262421	679.246127	CPD5182	33.453777	154.97069
CPD13786	8.44805285	208.144628	CPD31444	24.1648749	547.332438	CPD51820	8.80701005	1245.05927
CPD1379	1.4398599	305.99093	CPD31447	24.2229147	644.476767	CPD51822	8.83039224	1652.7488
CPD13796	8.17127187	394.072275	CPD31448	24.1711535	362.283923	CPD51825	8.78199626	1051.67903
CPD13799	9.07712715	370.124256	CPD31452	24.1888328	681.322083	CPD51826	8.8589539	811.376543
CPD1380	1.4773069	498.1038	CPD31454	24.1961766	712.249635	CPD51827	8.78414496	1052.08139
CPD13819	8.64108411	163.126505	CPD31456	24.1999111	794.349987	CPD51829	8.76756968	1040.27638
CPD1382	1.554325	203.99666	CPD31457	24.2312133	711.254036	CPD51831	8.75439709	871.9106
CPD1383	1.4379454	262.00866	CPD31462	24.5634987	497.431713	CPD51832	8.8487228	1052.27729
CPD13830	9.06717227	212.024287	CPD31463	24.1591436	383.339395	CPD51834	8.83021423	1002.43598
CPD1384	1.4589643	241.01825	CPD3147	19.091271	344.11868	CPD51838	8.77808016	1051.88153
CPD13845	9.89740736	634.26255	CPD31470	24.4954546	777.636509	CPD51839	8.82337912	813.212087
CPD1385	1.4707008	199.01319	CPD31474	24.523875	806.704025	CPD51845	8.79803736	694.04215
CPD13851	9.83259498	404.078243	CPD31476	24.5371499	397.355765	CPD51848	8.79681655	1131.52294
CPD13854	9.56202842	1238.28649	CPD31477	24.5376243	838.66315	CPD51849	8.88315509	818.213886
CPD13855	9.97724396	367.183844	CPD31479	24.5491577	441.319121	CPD5185	33.24723	231.17263
CPD13859	10.1615435	964.260664	CPD31480	24.5961989	509.308883	CPD51851	8.77600373	1033.67076
CPD13860	10.169389	964.7594	CPD31481	24.6012918	577.300229	CPD51854	8.87746784	1039.48103
CPD13861	10.1943873	1877.57963	CPD31482	24.5954611	638.273908	CPD51855	8.80029214	975.642936
CPD13865	10.3953585	225.042255	CPD31485	24.6883886	461.385211	CPD51857	8.76656381	1039.68126
CPD13866	10.3953675	233.028009	CPD31486	24.6851308	497.438667	CPD51858	8.79994685	798.33062
CPD13867	10.39644	434.118322	CPD3149	19.179381	148.01623	CPD51859	8.82212046	1045.88308
CPD1387	1.4621949	440.0147	CPD31490	24.7539439	335.31605	CPD51860	8.82450412	976.451075
CPD13872	10.9883128	159.068492	CPD31491	24.9482647	449.377773	CPD51862	8.78567712	1034.27282
CPD13877	11.7097771	360.178467	CPD31499	24.8513998	458.3766	CPD51864	8.81854282	1626.74735
CPD13878	11.6833814	232.147264	CPD3150	19.120272	809.54609	CPD51865	8.81437596	983.042663
CPD13879	11.8685076	204.010687	CPD31500	24.8130502	423.370765	CPD51866	8.80377642	1131.72206
CPD1388	1.4517761	290.01189	CPD31501	24.8452139	440.362464	CPD51867	8.81505136	815.873393
CPD13882	12.407276	276.134567	CPD31502	24.8463332	480.360175	CPD51868	8.69980424	993.634789
CPD1389	1.4347776	249.02608	CPD31503	24.8421807	548.350055	CPD51870	8.81864336	1228.30228
CPD1390	1.4205525	246.03536	CPD31506	24.9075553	877.705067	CPD51871	8.80955284	982.442395
CPD1391	1.4558599	232.9448	CPD31509	24.9089143	820.720058	CPD51872	8.80208854	816.872556
CPD13912	15.9446161	345.229125	CPD3151	19.134687	644.25125	CPD51873	8.8070826	871.738708
CPD1393	1.4434533	243.0255	CPD31510	24.9044193	509.309006	CPD51874	8.80324898	975.245392
CPD1394	1.4809206	262.15103	CPD31512	24.8541807	577.29962	CPD51875	8.80373979	1131.9222
CPD13940	17.8241043	181.091391	CPD31515	24.9443496	397.35575	CPD51877	8.82055604	1224.30798
CPD13946	17.9552267	488.33299	CPD31516	24.9172324	441.326365	CPD51879	8.81875973	819.035067
CPD1395	1.4650976	70.005488	CPD31518	24.9672499	1086.64092	CPD51880	8.80591605	975.447592
CPD13952	18.360397	521.348097	CPD31519	25.0032003	1025.61938	CPD51882	8.82080708	1228.55108
CPD13955	18.7201106	295.250837	CPD3152	19.142598	259.17577	CPD51883	8.82185423	1214.32188
CPD13969	19.7136774	319.252281	CPD3153	19.176709	278.29966	CPD51884	8.82358814	1307.601
CPD13979	19.919049	657.401375	CPD31534	25.1627001	1051.63571	CPD51885	8.84746394	1218.5641
CPD1398	1.4653955	424.03389	CPD31535	25.178831	415.35998	CPD51887	8.79900428	1040.07738
CPD13985	20.2966934	408.266425	CPD3154	19.159357	497.39152	CPD51888	8.81427776	809.882095
CPD1399	1.459339	321.96204	CPD31541	25.2016491	423.371	CPD51889	8.81474046	810.548037
CPD14007	20.764933	500.823654	CPD31542	25.2905001	855.515386	CPD5189	33.381931	294.87597
CPD14009	20.9544642	654.382754	CPD31544	25.2874615	860.471231	CPD51890	8.81347682	975.84848
CPD1401	1.4737932	380.06899	CPD31546	25.2938241	950.440741	CPD51891	8.82938037	1224.80754
CPD14013	21.1496215	842.481762	CPD31547	25.3479816	638.774733	CPD51892	8.82193402	982.642256
CPD14014	21.1323035	824.496923	CPD31548	25.2943741	1018.43354	CPD51893	8.79503353	1218.80697
CPD1402	1.4654709	88.016127	CPD3155	19.147	134.03687	CPD51894	8.82199821	1215.07209
CPD14028	21.95555	564.392255	CPD31550	25.5696795	606.284269	CPD51895	8.82124757	809.549725
CPD1403	1.444067	209.93294	CPD31552	25.378588	638.775144	CPD51898	8.8352493	976.0496
CPD14042	21.9322996	420.257661	CPD31554	25.4146077	511.454532	CPD51899	8.82205319	1214.82232
CPD1407	1.4470076	496.05114	CPD31559	25.4505315	430.3441	CPD5190	33.570537	99.003946
CPD14073	22.3701164	582.411731	CPD3156	19.150754	56.06289	CPD51900	8.82157201	1224.0588
CPD14087	22.6317673	544.388185	CPD31561	25.6500855	711.529506	CPD51901	8.82268718	1224.55812
CPD14088	22.6429046	539.430419	CPD31562	25.6416486	449.386085	CPD51902	8.8126818	812.704389
CPD14093	22.9044637	826.484438	CPD31566	25.6796555	711.527775	CPD51903	8.8242008	1943.90698
CPD14100	22.7933632	534.875428	CPD31569	25.7467981	823.629315	CPD51905	8.42126428	855.086511
CPD14106	23.0006559	558.390875	CPD3157	19.187654	199.07751	CPD51906	8.82319735	971.858315
CPD14119	23.3039612	720.454569	CPD31574	25.7531991	1206.51589	CPD51907	8.81931446	809.715911
CPD1412	1.5001057	379.96846	CPD31575	25.7616966	449.337317	CPD51908	8.87980862	1244.80498
CPD14121	23.3786208	660.445627	CPD31577	25.7738399	866.701637	CPD51909	8.81633466	1228.05216
CPD1413	1.5223289	144.16128	CPD31579	25.7757486	411.371645	CPD51910	8.82144864	1215.32201
CPD14135	23.6012646	646.458647	CPD3158	19.19635	323.2095	CPD51911	8.82359911	971.459495
CPD14147	23.8921829	740.454339	CPD31581	25.7813822	455.338508	CPD51912	8.81235132	810.381641

CPD14148	24.03438	391.328655	CPD31583	25.7996037	523.32565	CPD51913	8.81749737	1228.80222
CPD14152	24.1578648	894.588305	CPD31584	25.8130007	591.318164	CPD51914	8.82279832	972.0576
CPD14156	24.2506312	556.39162	CPD3159	19.18309	278.15181	CPD51915	8.8205038	810.21498
CPD14157	24.2678787	814.518233	CPD31591	25.8824346	898.428886	CPD51916	8.83732925	1045.68569
CPD1416	1.5572172	394.06088	CPD31593	25.9452223	848.755344	CPD51917	8.8224975	1214.57227
CPD1417	1.4454885	134.02103	CPD31596	25.9379456	387.314265	CPD51918	8.81963709	972.457889
CPD14173	25.1476518	608.042015	CPD31599	25.988492	445.352558	CPD51919	8.82070213	810.04825
CPD14174	24.9790792	454.328689	CPD31601	26.0117989	437.3869	CPD5192	33.429171	246.93443
CPD14185	25.4874746	606.4904	CPD31608	26.1143634	588.546609	CPD51920	8.82494912	979.848617
CPD1419	1.5320721	348.01885	CPD31609	26.1889289	510.373407	CPD51921	8.8277676	1220.06651
CPD1421	1.6402603	140.01041	CPD3161	19.198866	626.28482	CPD51922	8.82190227	971.65934
CPD14211	26.4729398	821.52838	CPD31610	26.1950004	470.363213	CPD51924	9.13979724	1249.80064
CPD14221	27.1104993	344.325088	CPD31611	26.2788172	475.400573	CPD51925	8.82219011	1220.31566
CPD14250	29.1028171	854.469329	CPD31613	26.4402487	463.401535	CPD51926	8.81422215	1046.48471
CPD1426	1.5585006	142.02714	CPD31614	26.4500034	507.367814	CPD51927	8.8199433	976.650806
CPD14260	28.9612829	594.465122	CPD31616	26.5791664	769.564665	CPD51929	8.83571938	979.249772
CPD14267	30.3758578	994.536579	CPD3162	19.198906	674.35493	CPD51931	8.82579084	979.448465
CPD1427	1.255901	243.08402	CPD31620	26.8111399	375.311243	CPD51933	8.82756645	979.64879
CPD1428	1.5500263	71.073837	CPD31623	27.1394684	401.32952	CPD51934	8.86571342	1636.74659
CPD14299	33.1333131	517.823844	CPD31624	27.2064466	489.41752	CPD51935	8.8134177	980.048968
CPD1430	1.201055	237.06889	CPD31628	27.3244204	425.389184	CPD51937	8.81363639	983.241574
CPD1431	1.4208619	196.03926	CPD3163	19.201419	765.52125	CPD51938	8.82199996	816.373421
CPD1432	1.5821317	117.07802	CPD31632	27.5165923	680.461888	CPD51939	8.83461042	811.210226
CPD14324	33.1286667	584.799733	CPD31633	27.5415828	864.498167	CPD5194	33.494737	141.17221
CPD1433	1.5570001	582.05561	CPD31637	27.637895	473.379836	CPD51941	8.8264289	1626.41237
CPD1434	1.562925	244.07344	CPD31638	27.7167501	642.246667	CPD51942	8.81777374	819.202441
CPD14342	33.1359092	303.932955	CPD31639	27.7076274	607.514175	CPD51943	8.82347633	1307.35176
CPD14346	33.2278944	170.99529	CPD31641	27.7146045	638.483267	CPD51944	8.8334643	1307.85453
CPD1435	1.56275	123.03788	CPD31645	27.7153062	308.304708	CPD51947	8.8681251	1155.04533
CPD14369	33.24465	227.888289	CPD31648	27.724437	371.297087	CPD51948	8.84623488	1092.49881
CPD1437	1.6305188	130.02684	CPD31650	27.7177248	84.0939455	CPD51955	8.81547553	1046.08481
CPD14394	33.4702309	162.966587	CPD31652	27.7209141	870.564783	CPD51958	8.85976219	825.873609
CPD14399	33.4304576	61.9971727	CPD31653	27.7209982	628.26655	CPD5196	33.328695	339.89719
CPD1440	1.5653846	202.04875	CPD31654	27.7174708	110.109512	CPD51960	8.88838882	826.042455
CPD1444	1.5701482	333.1487	CPD31655	27.717598	152.154915	CPD51962	8.87634817	1248.55063
CPD1446	1.5666924	660.08035	CPD31657	27.7175312	418.307	CPD51963	8.89919803	1253.29519
CPD1447	1.5723334	671.00567	CPD31658	27.7183737	351.239075	CPD51964	8.88500536	1155.54322
CPD1449	1.579533	251.08173	CPD3166	19.206168	326.15337	CPD51965	8.83512381	1239.06317
CPD14493	1.01583313	435.85475	CPD31660	27.7143474	138.13968	CPD51966	8.87580763	1155.29765
CPD1450	1.5614102	129.07729	CPD31661	27.7299111	732.543818	CPD51967	8.81427776	1248.79922
CPD1451	1.4858702	692.03771	CPD31662	27.7279417	332.140559	CPD51970	8.90039703	770.03035
CPD1453	1.5615	146.02199	CPD31665	27.7256818	432.155171	CPD51972	8.87808734	1002.83624
CPD14557	1.07364383	321.961386	CPD31667	27.7515715	456.274657	CPD51973	8.89340307	770.197941
CPD1456	1.5615789	110.001	CPD3167	19.200076	770.48082	CPD51974	8.86342984	1002.63523
CPD1457	1.5738998	239.06867	CPD31671	28.014084	735.758	CPD51975	8.84998205	811.545241
CPD14574	1.08425	567.090194	CPD31672	27.9918	533.475647	CPD51978	8.89185189	924.03625
CPD14577	1.49538906	186.06522	CPD31674	27.9749967	713.54322	CPD5198	33.511689	224.02209
CPD1458	1.5776132	362.97185	CPD31676	27.9790425	695.53236	CPD51982	8.78245973	811.373256
CPD14583	1.08595975	564.095104	CPD31679	28.0250469	425.387195	CPD51983	8.89521296	1245.55682
CPD1460	1.5681579	135.05453	CPD31685	28.1757845	451.399842	CPD51985	8.81530857	818.702371
CPD14601	1.14713645	898.225986	CPD3169	19.372721	672.38943	CPD51988	8.90422214	924.436883
CPD14608	1.16247788	804.169205	CPD31693	28.3905426	695.532455	CPD51989	8.86048662	826.209163
CPD1461	1.5616511	161.06898	CPD31694	28.3955615	713.542694	CPD5199	33.453125	276.85481
CPD14610	1.15636372	770.113418	CPD31697	28.4530984	934.562585	CPD51990	8.81074843	995.453144
CPD14617	1.1404688	485.13654	CPD31698	28.6353844	598.424306	CPD51992	8.90538165	999.6421
CPD14618	1.15280001	657.11615	CPD3170	19.212525	148.01609	CPD51993	8.9137942	1252.54475
CPD1462	1.5472809	114.0321	CPD31702	28.7264468	477.4157	CPD51994	8.86955753	819.36695
CPD14621	1.1561818	523.187418	CPD31707	29.0682558	682.477642	CPD51999	8.87501747	1249.05202
CPD14625	1.1670746	92.0469368	CPD3171	19.242269	458.32134	CPD5200	33.372938	171.95015
CPD14627	1.15629991	533.22748	CPD31710	29.509089	884.474457	CPD52002	8.84850806	813.045233
CPD1463	1.5622973	307.08309	CPD31713	29.2972444	439.402855	CPD52005	8.90275615	770.364257
CPD14630	1.15804985	471.18774	CPD31714	29.5209739	862.4888	CPD52007	8.90183805	999.04158
CPD14636	1.15400551	195.055887	CPD31715	29.447904	392.356971	CPD52008	8.91604849	826.375907
CPD14637	1.21723966	219.109179	CPD31717	29.4423384	465.41719	CPD52010	8.9545891	1226.56725
CPD1464	1.5704234	253.07528	CPD31718	29.842738	884.474392	CPD52011	8.90164774	924.63708
CPD14645	1.19221369	238.074014	CPD31719	29.471277	352.3699	CPD52012	8.87724415	995.65086
CPD14646	1.15108617	670.081431	CPD31720	29.5967501	414.342545	CPD52013	8.8613053	999.244447

CPD14649	1.09353827	519.9852	CPD31721	29.5772087	885.4772	CPD52014	8.88724616	1002.23644
CPD1465	1.5619821	174.01654	CPD31722	30.0562928	491.432795	CPD52015	8.89358152	1239.81339
CPD14653	1.18549334	185.067529	CPD31723	30.1802785	391.371072	CPD52016	8.87441723	1245.31057
CPD14659	1.22185796	209.054276	CPD31725	30.3318525	334.253076	CPD52017	8.91846757	826.541836
CPD1466	1.5691276	260.0476	CPD31727	30.339449	312.263306	CPD52018	8.92635442	998.843991
CPD14662	1.25681018	409.941991	CPD31729	30.6205501	459.406706	CPD52019	8.8964313	1239.56382
CPD1467	1.5662778	287.04881	CPD3173	19.246979	453.3672	CPD52020	8.91383447	1239.31342
CPD14671	1.32843521	162.052423	CPD31734	31.8145928	475.3877	CPD52022	8.90557627	991.65165
CPD14672	1.22242439	110.03222	CPD31735	31.8340393	453.418064	CPD52024	8.90745078	1238.81257
CPD14674	1.08741449	189.0232	CPD31737	32.0385735	324.33892	CPD52026	9.06729112	1226.31961
CPD14676	1.44553427	265.02182	CPD31738	32.0363891	364.33089	CPD52027	8.91630857	991.851408
CPD14678	1.44087073	493.99696	CPD3174	19.235019	200.17703	CPD52029	8.92043385	992.051379
CPD14679	1.42723427	480.032388	CPD31741	32.9261705	453.417689	CPD52031	8.91900051	991.452118
CPD1468	1.562375	132.02445	CPD3175	19.266479	742.4375	CPD52035	8.92321739	1245.05881
CPD14684	1.43642242	324.16765	CPD31752	33.1325001	421.371925	CPD52036	8.8982605	1002.03722
CPD14686	1.64136624	429.995313	CPD3176	19.266538	202.17077	CPD52039	8.8813209	832.373283
CPD14688	1.18764274	313.103421	CPD31767	33.1364286	208.163579	CPD52044	8.90004792	996.048478
CPD14692	1.40446212	325.066945	CPD3177	19.279814	658.2945	CPD52050	9.000625	981.059442
CPD14693	1.47231605	166.039611	CPD3178	19.280122	726.45276	CPD52051	9.00270263	981.258415
CPD1470	1.56135	128.01124	CPD31782	33.5466147	249.966338	CPD52053	9.0897277	994.849327
CPD14704	1.57276926	238.073712	CPD3179	19.294692	721.4958	CPD52057	9.01400243	815.045344
CPD1471	1.4914636	119.02016	CPD31807	1.07842471	231.994453	CPD52058	9.00233526	980.858107
CPD14714	1.64709005	150.030782	CPD31816	1.01337515	433.008425	CPD5206	33.402267	119.96324
CPD14715	1.65214868	225.008121	CPD3182	19.467734	251.22243	CPD52062	9.02878861	956.252132
CPD1472	1.5572244	223.07035	CPD3183	19.367871	297.26734	CPD52063	9.01593335	787.372507
CPD1473	1.5638793	137.99604	CPD3184	19.370925	953.66905	CPD52064	9.02121159	1180.30709
CPD14732	1.95844232	318.070986	CPD31856	1.06720397	251.012887	CPD52066	8.96753762	1242.29556
CPD1474	1.5573273	355.1479	CPD3186	19.384757	356.31369	CPD52067	9.017409	787.038853
CPD14743	2.246761	246.11904	CPD31871	1.10393441	265.032794	CPD52068	9.02560561	978.4538
CPD14748	2.33963612	103.099635	CPD31879	1.10287471	175.213631	CPD52069	9.03024583	815.380994
CPD1475	1.562	723.11899	CPD3188	19.307386	675.40928	CPD52070	8.98772144	817.881845
CPD1476	1.5761275	293.09612	CPD31882	1.13109829	479.09344	CPD52071	9.02012275	787.205844
CPD1477	1.5637907	122.04724	CPD31889	1.15287474	258.080088	CPD52072	9.02234493	1225.07419
CPD14771	5.46848906	364.0793	CPD3189	19.344813	414.29687	CPD52073	9.02864762	955.651965
CPD14772	6.57999336	350.149738	CPD31890	1.13250108	512.118612	CPD52074	9.02531288	944.247037
CPD1478	1.5639999	192.19122	CPD3190	19.344086	250.19207	CPD52076	8.92188447	1007.05576
CPD14784	6.05400874	840.079056	CPD31909	1.15722233	275.295994	CPD52077	9.03481604	816.559818
CPD14786	6.10433359	365.06005	CPD3191	19.471492	382.25154	CPD52078	9.02559695	955.451235
CPD14787	6.62750276	327.156271	CPD31913	1.16883347	253.0637	CPD52079	9.00772087	981.457761
CPD1479	1.5633333	971.1829	CPD31914	1.15866666	427.158067	CPD52080	9.02228284	1180.80794
CPD14793	7.04358211	206.05959	CPD31917	1.41586754	191.04368	CPD52082	9.02378683	1180.55503
CPD14797	6.68066648	406.159927	CPD31918	1.17060759	192.03065	CPD52083	9.05355809	994.446388
CPD14798	6.0046624	206.091521	CPD3192	19.356082	698.3998	CPD52084	8.9995011	818.04773
CPD1480	1.5702332	171.05107	CPD31922	1.17933142	91.0101889	CPD52087	9.20642042	1225.31563
CPD14805	6.93999672	591.153171	CPD31925	1.27347905	389.135442	CPD52088	9.02904698	1194.31338
CPD14806	6.91410283	444.153467	CPD31928	1.28185709	118.026531	CPD52089	9.17437245	1225.56895
CPD14807	6.93910787	634.14425	CPD3193	19.362254	677.46977	CPD52090	9.03159736	1195.06497
CPD14808	6.9291543	606.142288	CPD31933	1.40209718	285.105336	CPD52091	9.02633753	817.220689
CPD1481	1.3655166	163.06628	CPD3194	19.361105	682.42561	CPD52092	9.0050302	978.854547
CPD14818	7.30231789	213.082728	CPD31940	1.17532205	172.132768	CPD52093	9.02683814	945.046463
CPD1482	1.5807025	129.03917	CPD31946	1.62240078	429.147387	CPD52094	9.02855336	1194.81415
CPD1483	1.5654445	704.07542	CPD31950	1.34241199	364.100036	CPD52095	9.0268436	956.052047
CPD14830	7.71445999	390.150925	CPD31955	2.10124243	630.13715	CPD52096	9.02510803	944.445995
CPD1484	1.56712	276.027	CPD31956	1.60970347	371.108658	CPD52097	9.0247405	944.847268
CPD14848	8.00137997	220.021563	CPD31965	1.68936351	236.012736	CPD52098	9.02694664	944.646945
CPD1485	1.5692502	334.06579	CPD31966	1.68174993	295.018981	CPD5210	33.444676	328.91135
CPD14855	8.12910155	474.318144	CPD31971	1.99708332	251.0401	CPD52101	9.0254173	955.852195
CPD1486	1.5665714	359.04396	CPD31975	1.88356884	123.029312	CPD52102	8.97785394	1059.4693
CPD14860	8.17167646	410.033534	CPD31977	1.68246137	118.027548	CPD52103	9.03028457	978.251844
CPD14863	8.50713839	472.193795	CPD3198	19.750363	371.28236	CPD52104	9.0289477	1194.56408
CPD1487	1.5109491	187.08691	CPD3200	19.422657	370.27302	CPD52105	9.04653611	817.061092
CPD14871	8.63411831	353.173157	CPD32016	4.90697284	328.078775	CPD52106	9.04185314	1046.08511
CPD14881	9.14662505	230.04234	CPD3202	19.447	633.44358	CPD52107	9.06411993	823.21515
CPD14888	9.53454632	334.152905	CPD32022	5.33235657	325.140086	CPD5211	33.337135	95.002761
CPD1489	1.5676186	291.12016	CPD32029	5.71932717	322.154993	CPD52111	9.07037023	812.711993
CPD14891	9.92677802	248.077693	CPD3203	19.440962	638.39874	CPD52112	9.30235455	1010.05566
CPD1490	1.5703636	619.06977	CPD32031	5.82124809	640.1298	CPD52115	9.01827505	978.65448

CPD14900	10.899001	414.15046	CPD32033	5.83211782	346.126582	CPD52116	9.03849879	816.727463
CPD14906	12.2925	263.22256	CPD32039	6.01237412	377.133987	CPD52117	9.06183326	1045.88331
CPD14909	12.186083	190.155474	CPD3204	19.593417	382.25098	CPD52119	9.18703181	1047.09386
CPD14914	13.4685638	202.096258	CPD32042	6.03666685	773.211767	CPD52120	9.05199623	812.378067
CPD1493	1.5716875	525.02347	CPD32048	6.11431284	361.137462	CPD52125	9.04721982	978.050447
CPD1494	1.5637647	117.02427	CPD32049	6.09217984	391.149143	CPD52126	9.03527326	812.211729
CPD14944	16.9448179	319.251636	CPD3205	19.387678	297.26721	CPD52127	9.06699954	812.5448
CPD1495	1.2288272	248.09728	CPD32051	6.16710803	736.217577	CPD52128	9.04565099	816.894629
CPD14964	17.9829341	157.11075	CPD32056	6.13644158	346.126233	CPD52129	9.05024957	1255.31596
CPD1497	1.566	932.06341	CPD32057	6.17734843	322.164412	CPD52130	9.0757854	812.878642
CPD1498	1.5627695	195.01541	CPD3206	19.477627	321.2853	CPD52132	9.0436496	812.043456
CPD1499	1.5700526	186.17063	CPD32069	6.56913361	423.973573	CPD52133	9.08185504	974.452407
CPD1500	1.4841691	145.07495	CPD3207	19.478605	326.24213	CPD52134	9.13201914	1039.87705
CPD15019	20.1976224	423.354975	CPD32073	6.57846646	789.023587	CPD52139	9.03182695	980.263709
CPD15022	20.2579263	370.346481	CPD32075	6.57593336	944.107847	CPD52142	8.9864827	994.037851
CPD15024	20.2366662	680.490956	CPD32077	6.57642496	600.043486	CPD52143	9.10977316	1295.59235
CPD15025	20.2563517	193.110207	CPD3208	19.424587	356.31384	CPD52145	9.10564713	1036.47575
CPD1503	1.5652857	373.10782	CPD32086	6.78966391	363.259633	CPD52146	9.03698918	811.880117
CPD1504	1.5668	279.07779	CPD32088	6.86512088	397.0823	CPD52147	9.16398209	973.0489
CPD15047	20.9232716	912.5368	CPD32095	6.63983406	192.08101	CPD52148	9.11326709	914.827682
CPD1505	1.5697647	604.98469	CPD32106	7.10913468	480.131011	CPD5215	33.542719	78.934712
CPD15059	21.455971	223.190464	CPD32111	6.9658186	248.053011	CPD52150	9.1552345	1144.28339
CPD1506	1.5647273	676.06745	CPD32115	7.18625287	351.153425	CPD52153	9.11262912	1036.87575
CPD15076	21.8805209	282.199409	CPD32117	7.18274702	896.129583	CPD52154	9.11072083	1296.34222
CPD1508	1.5670414	481.09941	CPD32118	6.93602004	221.046611	CPD52155	9.15607558	1143.78223
CPD15089	22.2407797	844.491342	CPD32129	7.33462416	538.134793	CPD52156	9.12698871	1143.53097
CPD1510	1.5652	192.02744	CPD32130	7.3405576	384.071444	CPD52157	9.10805233	974.85434
CPD15100	22.7885248	517.328678	CPD32133	7.02995759	329.11232	CPD52158	9.08673425	987.852855
CPD15105	22.8647418	814.485675	CPD32135	7.39366391	383.179467	CPD52159	9.08880727	1041.86714
CPD15106	22.9581108	473.793306	CPD32136	7.40698664	211.054913	CPD5216	33.497325	959.96097
CPD1511	1.566	688.09275	CPD32138	7.4048984	462.1734	CPD52160	9.06118997	994.645186
CPD1512	1.5641814	112.02696	CPD32139	7.42362273	407.982175	CPD52161	9.12077608	1037.27539
CPD15120	23.298	818.438657	CPD3214	19.690236	251.22366	CPD52162	9.10450058	1036.67627
CPD15131	23.5028944	620.263263	CPD32140	7.42716955	415.113275	CPD52163	9.15843962	1306.36123
CPD15137	23.8447626	269.271806	CPD32148	7.55987416	918.246494	CPD52164	9.11055195	1037.07593
CPD15138	23.8956353	638.273917	CPD32149	7.54714519	353.114818	CPD52165	9.13867653	993.230925
CPD1514	1.5657353	331.12826	CPD3215	19.539451	610.34793	CPD52166	9.07810901	985.060867
CPD1515	1.5435009	663.10682	CPD32150	7.55962302	487.263875	CPD52167	9.06773184	1320.33654
CPD15153	25.0745554	1212.85799	CPD32153	7.62449683	365.13301	CPD52168	9.11285345	1295.84196
CPD15157	25.087029	1292.50717	CPD32156	7.71315162	538.133408	CPD52172	9.11340449	1296.09375
CPD1516	1.5685	781.0331	CPD32157	7.72372551	554.158273	CPD52173	9.15994581	1044.48861
CPD15161	25.1268342	778.355707	CPD32158	7.73410701	401.095211	CPD52174	9.12675716	987.438287
CPD15162	25.2074915	783.407192	CPD32159	7.7236422	790.114529	CPD52175	9.16543425	993.630927
CPD15164	25.1154688	606.875722	CPD3216	19.532918	589.41819	CPD52179	9.1182496	1320.59084
CPD15170	25.5163893	1028.55732	CPD32160	7.73237464	406.0499	CPD52180	9.09629513	974.653118
CPD15173	25.5821075	960.83293	CPD32161	7.73468728	422.021415	CPD52181	9.12038958	1144.03333
CPD1518	1.5779782	207.05702	CPD32165	7.77023409	350.063406	CPD52182	9.11792023	1320.08841
CPD15182	25.9227553	561.441083	CPD32169	7.83324754	337.068294	CPD52184	9.09634931	1049.08174
CPD15189	26.2200866	405.373178	CPD3217	19.535651	594.37391	CPD52185	9.08720872	1252.29611
CPD1519	1.5678865	327.11566	CPD32171	7.81720384	1032.15054	CPD52186	9.19152165	1241.28672
CPD15195	26.4317266	616.496778	CPD32173	7.828941	432.106371	CPD52187	9.11553518	975.254546
CPD15201	26.555748	590.485628	CPD32174	7.85222075	613.139462	CPD52189	9.13475462	806.363105
CPD15202	26.5581939	790.507891	CPD32175	7.85099892	187.085985	CPD52191	9.16441962	990.433225
CPD15203	26.5268028	842.510743	CPD32177	7.89180896	629.624837	CPD52192	9.14949178	1311.8546
CPD1521	1.5579625	220.06163	CPD32179	7.91320014	332.037287	CPD52193	9.11964832	987.235543
CPD15215	27.518253	653.539328	CPD3218	19.612773	187.1154	CPD52194	9.12608057	806.696817
CPD1522	1.5308293	186.09709	CPD32182	8.01227751	740.214625	CPD52195	9.21830871	1237.2927
CPD1523	1.5605356	192.14919	CPD32183	8.02338279	902.248239	CPD52197	9.19345057	989.637121
CPD15233	28.6665588	666.412706	CPD32185	8.14265453	415.118925	CPD5220	33.413944	199.99031
CPD1524	1.57	413.16126	CPD32190	8.1504774	265.075127	CPD52203	9.18211001	1040.47873
CPD15243	29.2371434	512.404937	CPD32193	8.15100856	526.16475	CPD52205	9.13676953	975.454221
CPD15254	30.3676449	936.957395	CPD32195	8.18889028	429.1282	CPD52207	9.12791044	1233.78936
CPD1526	1.565875	267.09692	CPD32199	8.16917317	558.136882	CPD52208	9.16219859	1305.86032
CPD1527	1.5667297	181.07381	CPD3220	19.584369	282.22329	CPD52209	9.06696586	1056.47541
CPD1528	1.5666923	307.01485	CPD32200	8.1807932	359.05404	CPD5221	33.521168	94.958852
CPD15302	33.1286666	454.847467	CPD32202	8.19098516	543.120927	CPD52210	9.16141692	1045.28938
CPD1532	1.5704706	493.07904	CPD32204	8.21632909	1048.30172	CPD52213	9.11694044	975.054568

CPD1533	1.5914357	311.12209	CPD32205	8.18752871	118.041727	CPD52214	9.15070882	966.837865
CPD1534	1.56835	765.05686	CPD32206	8.19308767	886.250538	CPD52215	9.13900902	806.02909
CPD1535	1.5685883	797.01158	CPD32208	8.19740012	390.0561	CPD52218	9.13364764	1208.79491
CPD15353	33.5460578	363.7918	CPD32209	8.19338821	774.097307	CPD52219	9.15659567	1624.75143
CPD15357	33.3623178	958.95785	CPD32210	8.19580013	406.029207	CPD52220	9.07278409	1056.88079
CPD15358	33.4493678	153.96935	CPD32211	8.1402933	444.161533	CPD52221	9.12950242	1209.54377
CPD1537	1.5252862	417.14505	CPD32213	8.24414955	542.229708	CPD52222	9.14027924	967.036811
CPD15375	33.2355273	251.96744	CPD32216	8.26076847	502.183613	CPD52224	9.13902015	805.864042
CPD1538	1.5692852	297.0802	CPD32217	8.26418068	537.218674	CPD52225	9.14040994	986.631618
CPD1539	1.5915544	180.04703	CPD32218	8.2978306	713.226333	CPD52226	9.10246154	1231.07399
CPD1540	1.5646038	306.07595	CPD3222	19.57444	260.22326	CPD52227	9.14158616	1234.04081
CPD1542	1.5764159	226.0345	CPD32221	8.23160771	456.1944	CPD52228	9.13002456	967.435984
CPD1543	1.5206048	603.99317	CPD32223	8.37953223	636.277382	CPD52229	9.13160932	967.635665
CPD1544	1.5293499	433.17489	CPD32226	8.29135269	340.145275	CPD52230	9.13463173	1233.53751
CPD1545	1.5334383	181.19304	CPD32228	8.4285072	392.070635	CPD52231	9.13801025	967.236511
CPD1546	1.5713635	474.01582	CPD32232	8.45484561	383.178957	CPD52232	9.13527006	1209.04488
CPD1547	1.5449254	354.07313	CPD32233	8.46057179	348.142264	CPD52233	9.13616274	1209.29448
CPD1548	1.6510859	381.19623	CPD32236	8.49373129	398.089022	CPD52234	9.18901939	1237.54021
CPD15501	1.0039585	262.930156	CPD32239	8.48153595	420.067611	CPD52235	9.13817962	806.196274
CPD1551	1.5345637	630.01106	CPD3224	19.625956	195.10506	CPD52238	9.15300553	1233.28948
CPD1553	1.5706046	484.02021	CPD32244	8.62370205	440.16862	CPD52239	9.16235594	427.241115
CPD1554	1.559048	392.97695	CPD32249	8.62174495	654.147358	CPD5224	33.566171	76.99545
CPD1555	1.5511407	573.03072	CPD3225	19.582224	203.09223	CPD52241	9.15688173	805.698765
CPD1556	1.6124091	263.08235	CPD32251	8.56916361	965.254019	CPD52243	9.14958076	1208.54653
CPD1557	1.5670771	343.10032	CPD32253	8.65399404	1460.44057	CPD52244	9.13316961	806.530065
CPD1558	1.579882	221.10085	CPD32255	8.65007515	965.751462	CPD52245	9.13778268	967.835625
CPD1559	1.5709999	203.01302	CPD32256	8.88644791	973.24045	CPD52247	9.17147491	915.027207
CPD1560	1.5452722	345.04544	CPD32259	8.67240698	578.163324	CPD52248	9.17409833	1330.35131
CPD1561	1.5690562	299.12436	CPD3226	19.673192	352.26139	CPD5225	33.535594	99.928656
CPD1564	1.5662203	598.05048	CPD32264	8.77349463	382.162121	CPD52250	9.01865974	1219.81318
CPD15641	1.06655536	221.021711	CPD3227	19.81398	436.22098	CPD52252	9.15755586	1625.08524
CPD1566	1.3790402	448.20488	CPD32270	8.87280089	540.1673	CPD52253	9.16778854	1241.53801
CPD15665	1.07868504	313.978607	CPD32271	8.90992226	646.657607	CPD52258	9.14866742	1201.31704
CPD1567	1.559973	265.18604	CPD32272	8.90566314	822.256058	CPD52259	9.17663978	1503.64161
CPD1568	1.5701355	437.97439	CPD32275	8.9158561	646.158771	CPD5226	33.519101	258.88722
CPD15681	1.1212409	373.998243	CPD32277	8.94606091	474.11888	CPD52260	9.1834628	982.842005
CPD1569	1.5711388	406.20001	CPD32280	9.06032967	334.105789	CPD52262	9.13272728	1219.3167
CPD15692	1.14293921	269.938859	CPD32287	9.26100064	681.238762	CPD52263	9.15572755	1217.04598
CPD15697	1.155	449.154025	CPD32291	9.32688954	634.277389	CPD52264	9.06583139	1040.08121
CPD15703	1.15689992	132.04494	CPD32293	9.62518565	514.167875	CPD52265	9.173699	1064.48235
CPD1571	1.5773975	361.15307	CPD32296	9.47350073	588.183217	CPD52268	9.10780115	974.246125
CPD15710	1.51946301	200.0223	CPD32297	9.44949641	417.144056	CPD52269	9.16179748	1217.29566
CPD15711	1.15857827	357.108053	CPD32298	9.46537026	623.220825	CPD5227	33.608989	90.001561
CPD15714	1.55437631	130.029993	CPD3230	19.688284	369.28792	CPD52270	9.16610787	1216.79481
CPD15719	1.35231809	230.118618	CPD32300	9.45874549	416.809162	CPD52271	9.16947332	1038.26301
CPD1572	1.5837756	238.08917	CPD32303	9.44936591	482.21226	CPD52273	9.16229518	1044.89015
CPD15721	1.25292922	435.930164	CPD3231	19.799187	504.31196	CPD52274	9.16129427	1218.56666
CPD15724	1.21172438	149.053383	CPD32310	9.5874721	988.283071	CPD52276	9.17884643	1212.54919
CPD15727	1.16830266	343.982722	CPD32319	9.71737667	286.04765	CPD52277	9.13097341	1064.88537
CPD1573	1.5685358	629.00942	CPD3232	19.61336	904.47893	CPD52278	9.15794004	1305.35747
CPD15734	1.09193612	101.046583	CPD32321	9.80882812	493.249975	CPD52279	9.16488993	1306.11104
CPD15735	1.44010756	150.028408	CPD32323	9.81287425	1326.38247	CPD52280	9.1767006	802.87325
CPD15746	1.57083308	329.111421	CPD32324	9.88199599	220.178736	CPD52282	9.14909195	1044.28564
CPD15753	1.57776182	285.076143	CPD3233	19.619539	545.39248	CPD52283	9.16445788	1218.81756
CPD15754	1.56314287	384.120505	CPD32330	9.97286284	292.130988	CPD52284	9.17981678	970.040275
CPD1576	1.5711006	489.52635	CPD32336	10.0514269	988.282607	CPD52287	9.15024161	1243.05557
CPD1577	1.575641	189.0621	CPD32338	10.1184877	194.162833	CPD52289	9.17490821	1616.40521
CPD15774	1.35350594	221.08391	CPD3234	19.612415	528.3677	CPD52290	9.18410113	982.641995
CPD1578	1.5695963	503.99502	CPD32340	10.1197041	482.21181	CPD52291	9.18400988	1636.73389
CPD1579	1.5788998	397.18623	CPD32346	10.4495826	516.128208	CPD52292	9.1828786	970.639011
CPD1580	1.2701981	218.12262	CPD32350	10.6498066	1162.3362	CPD52293	9.17624977	1203.31024
CPD1581	1.5731499	589.00905	CPD32351	10.639799	1000.28447	CPD52294	9.18530583	1203.55976
CPD1582	1.5798944	240.14715	CPD32353	10.7063325	662.187167	CPD52295	9.18036461	1503.89201
CPD15836	7.96700857	218.093615	CPD32355	11.0046432	1030.29373	CPD52296	9.17650467	1203.06049
CPD15838	7.96634996	196.036418	CPD3236	19.661894	239.22594	CPD52297	9.17590142	802.206845
CPD1584	1.5712338	432.97337	CPD32360	11.1689974	482.151447	CPD52298	9.1847841	1637.39982
CPD15848	8.26883319	1519.92357	CPD32363	11.2396332	492.126725	CPD52299	9.17755525	962.449125

CPD1585	1.5720181	436.96793	CPD32364	11.2468753	487.169662	CPD5230	33.496033	109.01124
CPD15855	8.26312167	230.042503	CPD32366	11.0278925	516.12815	CPD52300	9.17675475	963.248075
CPD15858	8.40251635	530.163329	CPD32367	11.294858	703.171271	CPD52301	9.17865457	1202.81042
CPD1586	1.5727232	586.08661	CPD32368	11.3466565	284.1613	CPD52302	9.17619975	1203.80985
CPD15865	8.45648938	366.152744	CPD32373	11.7101721	432.1106	CPD52305	9.17654852	802.539835
CPD15868	8.20907304	338.099856	CPD32375	11.743754	239.087275	CPD52306	9.17665426	963.048225
CPD15869	8.17293691	677.169059	CPD32378	11.8813258	478.1804	CPD52307	9.176834	1213.05061
CPD1587	1.5779998	235.17798	CPD3238	19.700422	522.29614	CPD52308	9.1764021	802.373125
CPD15871	8.56304928	197.121114	CPD32381	12.1498433	238.085056	CPD52310	9.20117801	1252.54382
CPD15880	8.96579629	181.07342	CPD32382	12.2337126	390.0959	CPD52313	9.17780055	802.04016
CPD1590	1.5822077	226.12984	CPD32397	13.6152894	502.688615	CPD52314	9.17825426	970.240245
CPD15902	9.37760917	458.272412	CPD3240	19.714187	501.5614	CPD52316	9.17759877	801.8739
CPD1591	1.2008642	401.15597	CPD32401	13.8274273	388.115957	CPD52317	9.17143565	1299.84586
CPD1592	1.5766668	211.00249	CPD32405	14.0718992	690.25355	CPD52319	9.21479463	1300.09858
CPD15921	9.57694244	182.127267	CPD32411	14.3086429	344.251707	CPD5232	33.503018	80.930398
CPD1593	1.5702859	468.99412	CPD32414	14.1622636	471.671029	CPD52321	9.18811551	982.442253
CPD1594	1.571514	353.13243	CPD32419	14.8940582	737.419765	CPD52322	9.16832634	1212.79911
CPD15946	10.0606649	218.09305	CPD3242	19.70043	638.27335	CPD52323	9.1913756	982.242474
CPD15951	10.190503	706.43801	CPD32421	14.8946994	695.3643	CPD52324	9.19410092	1308.85929
CPD1596	1.5865455	321.09769	CPD3243	19.701508	501.36528	CPD52325	9.16852561	1227.30273
CPD1597	1.5857498	399.20383	CPD32434	14.9924283	662.059543	CPD52326	9.18626704	1309.35324
CPD15978	14.470737	480.672645	CPD3244	19.709754	506.32155	CPD52327	9.1808942	1241.78732
CPD15980	14.6951654	488.665733	CPD32441	15.0903176	1041.51377	CPD5233	33.517605	284.88673
CPD1599	1.5642948	259.99846	CPD32443	15.1522099	1034.02922	CPD52330	9.18400602	1637.06575
CPD15991	15.0856238	503.308262	CPD32446	15.0202141	693.6183	CPD52331	9.18483155	1227.55157
CPD15993	15.338922	316.20275	CPD32448	14.9559552	687.85378	CPD52332	9.18518785	970.838753
CPD15999	15.6667465	414.204008	CPD3245	19.830313	203.09361	CPD52335	9.18839937	1228.05161
CPD1600	1.6071299	333.00007	CPD32450	15.0418193	698.831562	CPD52336	9.18758147	1227.80172
CPD16002	15.7590018	336.233054	CPD32457	15.4247985	369.227612	CPD52338	9.2049853	1299.59638
CPD16006	15.7987457	452.158933	CPD32461	15.5256465	348.252041	CPD52339	9.18750321	1228.30163
CPD1601	1.5805023	218.99038	CPD32463	15.755224	700.3605	CPD5234	33.517159	89.084469
CPD16015	17.1695408	1392.76616	CPD32467	16.1035065	349.26015	CPD52340	9.18499568	1212.30234
CPD16017	17.0656185	456.3602	CPD3247	19.771006	462.48406	CPD52344	9.21656196	1039.6768
CPD16026	18.0114275	390.257465	CPD32472	16.4685335	706.32862	CPD52345	9.02612873	1237.04228
CPD1603	1.5894168	410.17294	CPD32473	16.5723417	533.789	CPD5235	33.514783	243.86418
CPD16030	18.3436778	702.290094	CPD32477	16.5133337	541.278109	CPD52356	9.23567778	1039.8794
CPD16037	18.8474038	1476.41596	CPD32478	16.6246998	790.947142	CPD52357	9.22752564	439.240073
CPD1604	1.565622	255.0956	CPD32479	16.5990998	790.44333	CPD52358	9.18172274	989.833094
CPD16046	19.416492	297.267367	CPD3248	19.766584	462.29549	CPD52359	9.16295642	1243.31051
CPD1606	1.593569	323.1218	CPD32480	16.6166145	541.775864	CPD52361	9.23211015	1059.67429
CPD16061	20.3452199	277.240287	CPD32483	16.613174	533.301473	CPD52362	9.20949402	993.030821
CPD16062	20.307877	299.228313	CPD32486	17.0866289	239.052042	CPD52363	9.11569431	1223.06559
CPD1607	1.5955345	369.16448	CPD32489	16.9904003	624.25758	CPD52368	9.25299463	1246.79756
CPD16072	20.393491	298.250508	CPD3249	19.775393	457.34052	CPD52369	9.14066945	964.823582
CPD16073	20.4230706	638.27388	CPD32490	16.9387959	508.335827	CPD52370	9.21020086	990.03307
CPD1608	1.5657002	394.15923	CPD32493	17.1657144	341.397529	CPD52371	9.24749727	384.174567
CPD1610	1.5787498	171.00617	CPD32496	17.2349087	397.260791	CPD52372	9.297126	1061.87333
CPD16100	21.5666704	770.500461	CPD32497	17.3386138	339.219567	CPD52374	9.30060602	1062.07148
CPD16104	21.6266334	392.276463	CPD32499	17.3363205	675.814264	CPD52375	9.28305724	1042.27466
CPD16105	21.550744	334.15885	CPD3250	19.771889	478.27024	CPD52376	9.27342215	1043.07434
CPD1611	1.5979334	490.12853	CPD32505	17.5266322	707.362479	CPD52377	9.27949812	1303.09743
CPD16113	22.0015501	250.225205	CPD3251	19.760271	251.22348	CPD52378	9.30178786	1042.67758
CPD1612	1.5774095	153.07382	CPD32514	17.9484442	527.310756	CPD52379	9.29008801	1062.67439
CPD16121	22.2635636	712.4932	CPD32517	18.1873847	365.399185	CPD52380	9.27052438	1144.03635
CPD16124	22.1729403	954.5753	CPD32531	18.6391328	433.3144	CPD52382	9.28726822	1042.87548
CPD16125	22.3421208	638.392023	CPD32534	18.5134657	329.235429	CPD52383	9.26075089	914.827933
CPD16145	22.6694099	368.274182	CPD32544	18.5400109	327.222656	CPD52384	9.29483523	868.729933
CPD16148	22.85506	969.612887	CPD3255	19.820233	418.44828	CPD52385	9.30249772	1062.27322
CPD1615	1.5940176	286.97248	CPD32551	19.0470833	483.390742	CPD52387	9.29239617	1042.47729
CPD16152	22.9192507	412.308267	CPD32555	19.2264008	345.267285	CPD52388	9.23627936	1143.78544
CPD16154	22.904678	630.233738	CPD3256	19.820908	396.28746	CPD52389	9.30343148	1143.52982
CPD1616	1.6231266	281.18308	CPD32564	19.334	356.419817	CPD5239	33.518761	154.97037
CPD1617	1.6151201	283.09329	CPD32566	19.3611108	400.306406	CPD52392	9.28967342	1220.79832
CPD16175	23.15136	792.519755	CPD32568	19.3515986	687.38734	CPD52395	9.33664825	1174.77851
CPD16178	23.277405	679.31287	CPD3257	19.818206	418.27009	CPD52396	9.39920157	965.644663
CPD1618	1.6178592	201.10981	CPD32571	19.4974667	543.446553	CPD52398	9.35071594	939.827225
CPD16184	23.3172529	622.396543	CPD32573	19.6088508	696.4823	CPD5240	33.514385	179.98214

CPD1619	1.6432367	302.04977	CPD32579	19.7417856	672.479186	CPD52401	9.33688831	1211.3065
CPD16193	23.7990154	479.383026	CPD3258	19.879777	598.61475	CPD52402	9.36333398	1169.2951
CPD1620	1.6354988	264.07831	CPD32584	19.9841665	509.406367	CPD52407	9.35981504	1168.7926
CPD1621	1.6125385	137.08206	CPD32585	20.1386979	472.337287	CPD5241	33.51491	245.85522
CPD16215	25.0777892	636.259721	CPD3259	19.87154	598.40139	CPD52411	9.46341737	959.647685
CPD1622	1.5732309	290.94682	CPD32591	20.1340003	507.381763	CPD52412	9.36599575	1169.04451
CPD16226	25.4833712	1057.65302	CPD32593	20.190422	718.423617	CPD52414	9.39718006	965.845038
CPD16227	25.4827334	954.983042	CPD32599	20.2184611	503.3864	CPD52418	9.36989182	1193.53398
CPD1624	1.6481307	279.20209	CPD3260	19.857914	225.15162	CPD5242	33.511158	158.96331
CPD1625	1.6469381	417.30826	CPD32609	20.3981979	357.88767	CPD52421	9.36221015	935.435593
CPD1626	1.670177	131.09485	CPD32610	20.3833999	358.0777	CPD52422	9.36276809	1169.54289
CPD16261	26.7110164	790.5134	CPD32616	20.3481432	747.369843	CPD52424	9.32523254	1207.05792
CPD1627	1.6427687	85.08974	CPD32617	20.3961869	995.65706	CPD52425	9.02359531	1004.6577
CPD16274	27.9063476	550.391571	CPD32618	20.3840001	715.164983	CPD52427	9.37095996	965.443287
CPD16280	28.169002	986.593262	CPD32627	20.4137854	527.360679	CPD52428	9.11807746	976.249936
CPD1630	1.6407988	347.26538	CPD3263	19.939066	686.28829	CPD52429	9.35916602	935.236953
CPD16303	28.9605722	835.5796	CPD32637	20.5427998	654.284693	CPD5243	33.522379	96.961445
CPD16325	30.8349864	746.501657	CPD3264	19.812772	203.09205	CPD52430	9.42030204	1214.31006
CPD1634	1.5877576	232.13796	CPD32645	20.5697142	877.355386	CPD52432	9.43954537	1295.32657
CPD16352	32.5969523	468.35379	CPD32647	20.6044995	758.422236	CPD52437	9.31630184	1239.05709
CPD1636	1.5737128	426.0082	CPD32653	20.7777296	353.29245	CPD5244	33.560832	177.98586
CPD16368	33.117341	230.157358	CPD32654	20.715909	700.511182	CPD52440	9.4187066	1207.30667
CPD16373	33.13625	452.854175	CPD3266	19.764107	352.26146	CPD52443	9.51135601	1009.65499
CPD1638	1.6252556	394.05928	CPD32661	20.8169264	383.29461	CPD52444	9.41507954	1206.80691
CPD1641	1.674077	103.09964	CPD32662	20.8120083	405.267717	CPD52445	9.42315617	1270.32875
CPD1642	1.6152794	311.96801	CPD3267	19.860282	195.10506	CPD52446	9.32482835	1010.25271
CPD16421	33.1627107	52.0117739	CPD3268	19.861776	226.12914	CPD52447	9.42762415	1283.31295
CPD1643	1.6436744	348.01902	CPD32691	21.204071	652.2875	CPD52448	9.41939809	1270.57814
CPD1646	1.6706455	332.02518	CPD32694	21.3890458	437.372875	CPD52449	9.41033694	998.247315
CPD1648	1.6813587	363.9771	CPD3270	19.667622	436.21948	CPD5245	33.511134	67.986793
CPD1651	1.6019285	179.00574	CPD32700	21.4212018	398.30025	CPD52450	9.48880568	1262.31755
CPD1652	1.683162	187.04862	CPD3271	19.90833	325.25917	CPD52451	9.41338348	1244.31171
CPD16529	1.00660875	316.009709	CPD32718	21.6624996	648.522012	CPD52452	9.43343988	1270.07509
CPD1653	1.6773037	211.97762	CPD3272	19.938418	330.21679	CPD52454	9.41946102	1294.82576
CPD16567	1.04014296	673.8134	CPD32727	21.7895977	477.376964	CPD52455	9.44002295	1016.46261
CPD1657	1.7335574	260.13478	CPD32733	21.9916616	680.240233	CPD52457	9.1987592	1009.85099
CPD16573	1.04592018	303.979283	CPD32734	22.0085555	808.616078	CPD52458	9.41083718	1294.5721
CPD1658	1.67119	274.01738	CPD32739	22.137	762.885375	CPD52459	9.44633112	1014.24946
CPD16581	1.05590894	409.98023	CPD3274	20.059843	390.09978	CPD52460	9.48253426	1014.84285
CPD16584	1.05583327	330.887967	CPD32749	22.1192835	438.3563	CPD52462	9.43106447	1270.82726
CPD1660	1.7075121	237.96962	CPD3275	20.003222	302.2472	CPD52464	9.44559876	839.211833
CPD1661	1.7030331	185.99829	CPD32751	22.3604286	695.300657	CPD52465	9.44511521	1258.31735
CPD16617	1.10422386	234.015567	CPD32752	22.2739209	355.3084	CPD52466	9.44531829	1258.81769
CPD16618	1.12281089	129.0433	CPD32756	22.4313335	857.554508	CPD52467	9.4482924	823.384088
CPD1662	1.6958155	118.02614	CPD32759	22.4401657	1096.76692	CPD52468	9.44415653	1258.56761
CPD16621	1.10760982	372.029083	CPD3276	20.017731	324.22682	CPD52469	9.44961301	1247.5545
CPD16623	1.11554584	87.0340409	CPD32762	22.4761584	1334.83767	CPD5247	33.513131	363.79114
CPD16626	1.18578828	119.059995	CPD32764	22.4850002	1106.68983	CPD52471	9.44739862	1007.05518
CPD1663	1.7005789	258.02377	CPD3277	20.005859	284.23595	CPD52472	9.44520025	1234.32589
CPD16630	1.43844374	151.031393	CPD32770	22.4572855	967.7038	CPD52473	9.44534859	987.860885
CPD16632	1.12256235	190.0953	CPD32794	22.5544444	408.771944	CPD52474	9.44715224	987.46151
CPD1664	1.7011598	111.02151	CPD3280	20.0866	277.24054	CPD52475	9.46675765	997.845442
CPD16644	1.14215006	251.09449	CPD32800	22.6494165	899.6571	CPD52476	9.44514361	987.66118
CPD1665	1.6977838	102.02205	CPD32801	22.6836801	391.307338	CPD52477	9.41743725	998.045073
CPD16650	1.33783817	255.086633	CPD3281	20.052173	260.21373	CPD52478	9.44535107	1259.06744
CPD16653	1.14744418	626.14055	CPD32812	22.7263691	500.362971	CPD52479	9.44705268	1006.85539
CPD16658	1.57984357	438.08279	CPD32814	22.9059997	507.421225	CPD5248	33.51295	286.88159
CPD16660	1.13131351	625.147157	CPD32819	22.9650556	413.295472	CPD52480	9.44539716	1007.25491
CPD16662	1.1598077	176.054977	CPD3282	20.098844	242.20295	CPD52481	9.4703252	822.88488
CPD16666	1.55240059	123.032922	CPD32824	22.9661716	652.280156	CPD52482	9.44565417	1234.07606
CPD16668	1.17681638	117.079853	CPD32828	23.0722856	672.574814	CPD52483	9.45406999	839.379107
CPD1667	1.7005471	100.01624	CPD32829	23.096459	1308.51026	CPD52484	9.44861153	822.551711
CPD1668	1.7065642	320.06054	CPD32830	22.6090688	612.2625	CPD52486	9.4479984	1006.65591
CPD16688	1.19538023	244.004715	CPD32837	23.2114324	505.4118	CPD52487	9.4461814	1267.0755
CPD1669	1.7107348	163.06452	CPD3284	20.134397	227.22459	CPD52488	9.44264974	822.718476
CPD16691	1.16999948	304.024557	CPD32842	23.2223562	417.75313	CPD52489	9.44916275	1233.82514
CPD16697	1.53481881	372.013515	CPD3285	20.082154	323.28155	CPD5249	33.532166	271.90636

CPD1670	1.7019454	181.02187	CPD32851	23.175797	395.760729	CPD52490	9.44284827	987.061678
CPD16702	1.23376724	462.050855	CPD32856	23.2403001	440.39196	CPD52491	9.4453526	1234.576
CPD1671	1.7440039	203.07503	CPD3287	20.19746	622.27904	CPD52492	9.51114406	994.853554
CPD16712	1.34915815	73.0517	CPD3288	20.210152	702.39369	CPD52493	9.44805651	1007.45505
CPD16715	1.55330216	149.052668	CPD32880	23.5594842	842.51398	CPD52494	9.4493846	1247.30719
CPD16718	1.23284842	506.039867	CPD32882	23.5055813	631.266538	CPD52496	9.32113589	1207.55553
CPD16720	1.33437228	478.014627	CPD32886	23.7764319	688.2503	CPD52499	9.42153678	1295.0782
CPD16724	1.50266609	152.032042	CPD3289	20.229762	428.31107	CPD5250	33.500025	327.90758
CPD16731	1.56386195	145.082824	CPD32896	23.7831763	470.402865	CPD52500	9.44635528	823.051285
CPD16740	1.42058324	181.193882	CPD3290	20.36506	423.35443	CPD52502	9.44975271	823.217555
CPD16745	1.50952035	259.178277	CPD32902	23.8307499	503.39405	CPD52504	9.45465898	1017.63827
CPD16748	1.50704056	234.023492	CPD32903	23.7893873	792.693567	CPD52506	9.45192979	838.878164
CPD1675	1.7053042	305.97137	CPD32909	23.8711898	383.339823	CPD52507	9.45021169	822.385553
CPD16750	1.57690941	229.031891	CPD3291	20.152005	661.43203	CPD52508	9.71774722	1255.06623
CPD16752	1.56738464	445.151662	CPD32911	23.8004071	704.224325	CPD52509	9.4487569	839.046962
CPD16757	1.65391437	279.205109	CPD3292	20.310542	384.28505	CPD5251	33.514793	181.97787
CPD1677	1.7233805	179.0797	CPD32920	23.9761107	650.271544	CPD52510	9.42113155	1248.0573
CPD16770	1.78214172	264.130846	CPD32925	24.1763748	384.270625	CPD52511	9.4450022	1238.55323
CPD1678	1.7209761	341.13359	CPD3293	20.145676	686.29438	CPD52512	9.38878214	1214.81271
CPD1679	1.7326158	241.04359	CPD32931	24.2378536	814.52067	CPD52514	9.38021087	1243.55878
CPD1680	1.7164458	202.13027	CPD32938	24.5381408	645.274671	CPD52519	9.50823354	427.23955
CPD1681	1.7271952	252.1186	CPD3294	20.253154	370.35187	CPD5252	33.518918	138.95652
CPD16810	2.24168911	220.09482	CPD3295	20.244685	379.32818	CPD52527	9.18452922	1233.0409
CPD1685	1.7387007	185.06775	CPD32963	25.4105648	452.330564	CPD5253	33.505346	140.95129
CPD16852	5.85493424	138.032227	CPD32964	25.4385178	511.452825	CPD52531	9.52794207	979.2428
CPD1686	1.4958192	188.98748	CPD3297	20.252216	618.42709	CPD52534	9.52715522	979.042585
CPD16869	6.96805661	233.9847	CPD32974	25.8613879	860.471481	CPD52537	9.52668785	979.442369
CPD16874	7.28900763	190.138831	CPD32975	25.8589352	855.515862	CPD52538	9.53380717	1224.0525
CPD16877	7.27980621	190.137825	CPD32976	25.8190015	659.2952	CPD52539	9.55949925	1061.87086
CPD16878	7.00102981	440.041225	CPD3298	20.259104	617.4164	CPD5254	33.512358	121.95652
CPD16887	7.71334378	350.14564	CPD32980	25.9152001	674.27154	CPD52542	9.54143374	1631.06865
CPD16891	7.98566583	476.178133	CPD32983	25.7337905	898.428022	CPD52543	9.54005228	799.04169
CPD1690	1.714189	188.11545	CPD32984	26.3699141	826.519487	CPD52544	9.54331844	967.2406
CPD16907	8.985322	286.047573	CPD3299	20.271657	367.21281	CPD52545	9.542101	1212.5416
CPD16917	9.00430476	491.23718	CPD32993	27.2192856	483.4077	CPD52546	9.5342774	1223.05371
CPD16922	9.23134484	1503.38689	CPD32994	27.3363186	798.470652	CPD52547	9.53963412	978.84351
CPD16928	9.3913704	340.132494	CPD32996	27.4981434	828.481873	CPD52548	9.5465973	1047.28917
CPD16949	12.624382	204.13609	CPD3300	20.253881	388.30406	CPD52549	9.54693847	1631.40218
CPD16954	13.8395021	479.668548	CPD33005	27.6983362	890.76832	CPD52550	9.51904489	1308.35762
CPD16956	14.3888774	690.253603	CPD33009	27.7057018	650.651033	CPD52552	9.53415376	1223.30271
CPD1696	1.8255999	145.10711	CPD3301	20.313268	169.10996	CPD52555	9.53815472	1223.5531
CPD16966	15.1476855	471.671032	CPD33019	28.0346199	713.775073	CPD52556	9.54031377	978.642721
CPD16969	15.146738	472.174662	CPD3302	20.259629	608.40525	CPD52557	9.54214483	1308.85863
CPD1697	1.7453694	382.13858	CPD33020	27.9769163	551.481392	CPD52558	9.54592937	1046.88995
CPD16970	15.1876802	394.249683	CPD33023	27.9903448	447.36366	CPD52559	9.54255013	958.849775
CPD16974	15.3144984	248.175018	CPD3303	20.269878	351.23832	CPD52561	9.54013648	969.83522
CPD16978	15.3487246	251.223175	CPD3304	20.280838	171.12015	CPD52562	9.53512367	1205.05683
CPD1698	1.908459	302.07559	CPD33046	29.5755486	857.52981	CPD52563	9.54685001	1198.81003
CPD16997	17.6870721	277.140973	CPD3305	20.273431	967.64671	CPD52564	9.52563252	959.248847
CPD17003	17.9504915	335.242919	CPD33051	29.9493516	697.547527	CPD52565	9.54715795	799.707025
CPD1701	1.8347508	281.14915	CPD33053	30.0472852	535.396557	CPD52566	9.54834574	1199.56004
CPD1702	1.7812233	150.02828	CPD3306	20.26012	152.08303	CPD52567	9.54806109	1047.08989
CPD17022	19.4682782	816.463587	CPD3308	20.285658	298.25047	CPD52568	9.56015209	1062.27549
CPD17033	19.6126088	566.323188	CPD3309	20.275863	408.26626	CPD52569	9.55184835	1046.69208
CPD17041	19.7873747	700.278207	CPD3310	20.133882	260.21349	CPD5257	33.30634	337.89577
CPD17045	20.2736954	408.442396	CPD3311	20.457762	362.30254	CPD52570	9.51611694	1199.05916
CPD17046	20.1733576	690.396519	CPD3312	20.216518	277.24039	CPD52571	9.54621541	1308.61007
CPD17048	20.4237977	985.595555	CPD33136	1.0332727	367.980527	CPD52572	9.53473035	1199.31
CPD1705	1.6414641	237.04947	CPD3314	20.368443	714.39665	CPD52573	9.39598998	1207.05867
CPD17056	20.6298504	520.3546	CPD33142	1.02300007	617.85985	CPD52575	9.545404	959.44888
CPD17068	20.7540531	608.261817	CPD3315	20.306524	239.22611	CPD52577	9.54570859	799.373925
CPD1707	1.8826932	240.14601	CPD33158	1.03199997	276.932327	CPD52579	9.55235467	799.874055
CPD17072	21.2376234	352.341092	CPD3316	20.160534	430.23939	CPD5258	33.505419	66.989162
CPD17073	21.4445795	676.2506	CPD3317	20.347776	990.55073	CPD52580	9.54636901	799.540226
CPD1708	1.9572082	332.08771	CPD33184	1.22265552	423.057013	CPD52581	9.54215388	1198.55998
CPD17087	22.1487687	652.257085	CPD33187	1.06854569	492.893018	CPD52583	9.54999971	959.848805
CPD1709	1.9640292	171.97415	CPD33189	1.09450062	610.832512	CPD52584	9.52369642	959.647107

CPD17090	22.1423555	882.53466	CPD3319	20.285835	702.39447	CPD52585	9.54769719	959.049345
CPD17094	22.1194852	312.26562	CPD33194	1.09119023	510.15	CPD52586	9.54754834	799.207545
CPD17098	22.3020562	636.258095	CPD33196	1.10459983	544.860533	CPD52587	9.54263432	1309.11158
CPD1711	1.2603169	232.04913	CPD3320	20.214139	697.43922	CPD52588	9.52377956	1047.48915
CPD1712	1.8941084	183.04505	CPD33202	1.09488889	509.882422	CPD52589	9.54580078	978.443973
CPD17120	22.9443095	608.263862	CPD3321	20.332351	1006.544	CPD52590	9.55538316	1066.48611
CPD17126	23.2879846	700.30755	CPD33213	1.03112965	846.149323	CPD52592	9.54437792	1223.80245
CPD17134	23.2660681	684.2711	CPD33218	1.11864441	638.916179	CPD52595	9.55553246	800.040847
CPD17137	23.5009618	826.486097	CPD3322	20.336499	299.23044	CPD52596	9.55966258	246.064533
CPD17138	23.5036504	456.119375	CPD33223	1.11290909	779.905627	CPD52599	9.55214453	460.19159
CPD1714	1.696449	105.04262	CPD33228	1.12044438	981.034356	CPD5260	33.534429	71.93011
CPD17151	23.8924519	982.636673	CPD33229	1.1382674	503.0373	CPD52601	9.55244869	455.23584
CPD17152	23.9937681	470.356073	CPD33234	1.13074971	640.080412	CPD52604	9.56246621	1043.07472
CPD17159	24.3877013	352.25834	CPD3324	20.39419	265.23664	CPD52606	9.55612995	238.081963
CPD1716	1.994468	145.15647	CPD33243	1.11391978	1068.07696	CPD52609	9.55060119	1631.73575
CPD17160	24.6685519	586.414376	CPD33246	1.13309095	927.083109	CPD52611	9.55691264	1062.07006
CPD17169	25.0871433	954.461054	CPD33249	1.18495707	143.057611	CPD52612	9.55301911	1210.8113
CPD1718	1.8049622	150.02863	CPD33254	1.13121849	583.138186	CPD52613	9.5530706	1042.47734
CPD17180	25.6669164	644.239805	CPD33256	1.1420553	569.192339	CPD52614	9.55523538	1208.79908
CPD1720	2.0207508	145.05351	CPD33258	1.13328712	571.118814	CPD52615	9.56107894	966.8382
CPD17207	26.6475848	860.471575	CPD33259	1.14949998	597.14855	CPD52616	9.59958028	416.198433
CPD17209	26.7439315	650.446522	CPD3326	20.51857	164.10584	CPD52618	9.55614532	1042.87676
CPD1721	2.0149422	87.101817	CPD33261	1.14357144	559.108929	CPD5262	33.450919	108.94234
CPD17212	26.8890652	1002.54934	CPD33267	1.15011747	697.104612	CPD52620	9.52872002	1042.2762
CPD17235	29.0229873	798.866469	CPD33269	1.13492284	713.086269	CPD52622	9.54349197	1303.34219
CPD17243	29.3955912	668.498005	CPD3327	20.374772	153.09991	CPD52624	9.56212326	1042.67698
CPD17251	30.3826403	780.18247	CPD33270	1.30526218	450.098971	CPD52626	9.5383026	1074.90195
CPD17259	30.9425112	375.290887	CPD33275	1.10898105	682.931937	CPD52628	9.31886726	1205.79168
CPD17272	32.8033939	686.193764	CPD33276	1.15083355	189.222733	CPD52629	9.59475346	968.447587
CPD17303	33.1293334	575.80485	CPD33279	1.24844873	363.064153	CPD52630	9.54841199	966.640677
CPD17323	33.1511149	241.158068	CPD33282	1.15318666	795.078625	CPD52631	9.57400238	868.7309
CPD1735	2.0972645	264.1193	CPD33284	1.20349899	243.119963	CPD52634	9.5875658	1227.81583
CPD1736	2.1534725	302.07636	CPD33287	1.17479953	536.05524	CPD52636	9.60515524	1287.31713
CPD1740	2.2380661	340.05603	CPD33288	1.15491668	727.078475	CPD52638	9.61463642	1262.3234
CPD17408	1.14732219	275.1488	CPD3329	20.338855	227.22499	CPD52639	9.56821125	1227.56594
CPD17434	1.02299952	449.944621	CPD33290	1.16328559	729.066	CPD5264	33.50339	96.010293
CPD1746	2.2902065	171.02551	CPD33294	1.17230751	547.020215	CPD52641	9.60791906	1286.56679
CPD17460	1.02922339	421.928633	CPD33297	1.18716656	507.192833	CPD52643	9.60467453	1287.06759
CPD1749	2.3407362	327.15134	CPD33298	1.17970073	644.99392	CPD52646	9.59519237	1262.82206
CPD1750	2.2725065	310.12093	CPD3330	20.363718	1254.8032	CPD52647	9.58729618	1010.06031
CPD1751	2.3422213	225.06286	CPD33306	1.21491472	467.06544	CPD52648	9.60631986	1262.57308
CPD1754	2.3841499	291.13128	CPD3331	20.3755	504.4629	CPD52649	9.71096695	542.305329
CPD1756	2.3731753	111.06694	CPD33312	1.22098663	392.95972	CPD5265	33.586231	110.01281
CPD1760	2.6508732	319.12504	CPD33315	1.28083394	544.852742	CPD52650	9.60308654	1009.65784
CPD17618	1.0823333	308.921217	CPD3332	20.39528	638.39297	CPD52651	9.59145337	1262.07039
CPD17619	1.09949937	555.064594	CPD33320	1.36503044	543.972673	CPD52652	9.58615308	1286.81513
CPD1762	2.4931888	337.05829	CPD33327	1.56078858	400.094921	CPD52653	9.6194646	1009.85849
CPD17622	1.13285327	86.0373652	CPD33332	1.55980005	739.0416	CPD52655	9.62472621	1010.25684
CPD1763	2.5141853	165.07885	CPD33334	1.58068435	243.119596	CPD52657	9.68159365	1255.56648
CPD17634	1.32019958	420.9857	CPD33335	1.56528563	523.1896	CPD52662	9.6833817	1231.32523
CPD17639	1.15298127	308.043889	CPD33341	1.55891722	132.03935	CPD52663	9.61293653	1230.32304
CPD1764	2.5761992	165.19261	CPD33343	1.56370007	521.07746	CPD52664	9.67181636	931.636664
CPD17643	1.14833332	365.045833	CPD33361	1.61488995	165.078272	CPD52666	9.68289722	1230.82583
CPD17646	1.59149999	191.098521	CPD33365	1.66509687	392.04345	CPD52667	9.67130007	1164.79496
CPD1765	2.5162495	119.07336	CPD33367	1.69562212	220.037887	CPD52668	9.64110128	985.2606
CPD17651	1.18782886	320.103068	CPD33375	1.58727135	465.09687	CPD5267	33.628564	127.94883
CPD1766	2.4879749	137.04602	CPD33376	1.41134394	354.9917	CPD52670	9.67375441	932.0382
CPD1767	2.6569928	357.08304	CPD3338	20.44903	580.39064	CPD5268	33.541189	89.003586
CPD1768	2.6296444	203.03381	CPD3339	20.560861	169.10955	CPD52681	9.6859423	984.863475
CPD17681	1.47206409	331.137777	CPD3340	20.398345	298.2506	CPD52682	9.68510845	1230.57626
CPD1769	2.4951021	321.0817	CPD33405	2.93366673	502.005939	CPD52683	9.68106935	1231.0767
CPD17692	1.23943718	176.045806	CPD33406	2.98856789	368.103162	CPD52684	9.72293482	627.229967
CPD17707	1.31924374	244.001231	CPD33407	2.98589175	517.974411	CPD52686	9.69179196	984.66346
CPD17716	1.64411426	220.035022	CPD33409	2.96420006	486.02506	CPD52687	9.73200112	816.724053
CPD17727	1.56260577	147.052292	CPD33412	2.98129258	145.052771	CPD52688	9.84030054	988.66157
CPD17736	1.55037845	117.079549	CPD33416	3.2173885	293.218989	CPD52689	9.73869053	979.667563
CPD17737	2.02586649	493.1935	CPD33418	3.39980197	383.10562	CPD52690	9.71168889	1255.3181

CPD17738	1.55866668	309.108286	CPD3342	20.453941	382.06318	CPD52691	9.72072256	1224.07768
CPD17740	1.56422306	55.0428778	CPD33432	5.10700025	702.202375	CPD52692	9.72360218	1224.32899
CPD17742	1.36926432	118.03905	CPD33446	6.02449928	452.128875	CPD52693	9.66420745	962.245829
CPD17757	1.56609997	192.12261	CPD3345	20.501553	198.16158	CPD52694	9.72424749	1224.57932
CPD1776	2.7146335	343.07615	CPD33454	6.38668315	326.100773	CPD52696	9.32341331	1249.30805
CPD17762	1.19307727	510.117544	CPD33457	6.34751769	164.047515	CPD52697	9.7308462	999.461258
CPD1778	2.749531	357.08344	CPD33459	6.54932855	815.1181	CPD52698	9.73384146	999.061637
CPD17787	1.56624999	164.04856	CPD33476	6.87562365	1164.30258	CPD52699	9.72644243	979.065383
CPD1779	2.7796427	218.12565	CPD33480	6.92795024	598.650429	CPD52701	9.72121266	1224.8272
CPD17797	1.56479982	123.129713	CPD33482	6.93680817	606.643625	CPD52702	9.67966054	1255.06622
CPD17800	1.18204319	468.220571	CPD33483	6.93468116	791.213319	CPD52703	9.75667022	416.201511
CPD17834	1.52544075	314.024708	CPD33485	6.89766436	405.092873	CPD52704	9.54220985	1253.56138
CPD1784	2.8657767	266.13579	CPD33486	6.88849827	582.144383	CPD52705	9.71577561	995.055512
CPD17847	1.39058483	537.1189	CPD3349	20.49897	308.19648	CPD52706	9.72995316	815.89138
CPD17849	1.67958776	252.115367	CPD33500	7.15322299	960.267945	CPD52708	9.73250291	1249.07467
CPD17853	1.68026529	281.183616	CPD33507	7.18991984	354.111036	CPD52709	9.72878545	979.466879
CPD17855	1.63365113	232.109095	CPD3351	20.500807	286.21541	CPD5271	33.567126	97.006717
CPD1786	2.9881461	246.12291	CPD33511	7.39070506	208.145524	CPD52710	9.69647345	1237.81043
CPD17864	1.69779992	347.265913	CPD33515	7.43095701	336.08285	CPD52711	9.73893634	815.724019
CPD1788	2.9292112	194.11419	CPD33522	7.42492639	394.08895	CPD52712	9.73170297	816.057495
CPD17915	2.09099459	178.973188	CPD33525	7.41691168	164.048225	CPD52713	9.74145905	1248.06995
CPD1792	3.0069614	219.11007	CPD33526	7.42000105	438.206071	CPD52718	9.73357941	1248.82614
CPD1793	3.0139141	257.06875	CPD33527	7.43245039	923.665491	CPD52719	9.73372897	816.390442
CPD1794	2.9831159	384.09824	CPD3353	20.60051	432.2832	CPD5272	33.550384	112.95641
CPD17944	2.41715797	379.147232	CPD33531	7.63895701	478.109442	CPD52721	9.72988536	816.224044
CPD17948	2.42730563	475.206338	CPD33536	7.72264548	680.266675	CPD52722	9.73447465	1248.57492
CPD17953	2.51614236	507.195543	CPD33538	7.72550325	452.036013	CPD52723	9.73322707	998.661867
CPD17958	2.51857184	313.18865	CPD33549	7.8380738	613.63425	CPD52724	9.73329496	998.86192
CPD17963	2.53475028	484.204019	CPD3355	20.474633	714.3987	CPD52726	9.73346732	816.556306
CPD17965	2.53379754	236.07976	CPD33551	7.83123983	595.374242	CPD52727	9.62717187	982.248333
CPD1799	3.2345633	157.07283	CPD33554	7.84883325	678.111892	CPD52729	9.70034443	990.251589
CPD17991	2.16876432	167.03658	CPD33558	7.89398667	1258.23476	CPD52732	9.49950357	1255.56753
CPD1800	2.8662101	165.13126	CPD33562	7.90017154	610.366788	CPD52733	9.76079974	1259.32107
CPD1801	3.2555049	353.07112	CPD33567	7.95200179	508.5956	CPD52735	9.76541456	1258.56837
CPD1806	3.3973375	246.11967	CPD33569	8.02478987	428.13	CPD52736	9.79478588	1251.56413
CPD1809	3.4067873	317.14633	CPD33575	8.1661277	746.166387	CPD52739	9.77355996	1258.82098
CPD18094	5.03744709	205.219089	CPD33577	8.17809014	572.112191	CPD52740	9.78087745	1234.32838
CPD18098	5.06037574	187.18345	CPD33578	8.17050093	738.434212	CPD52741	9.49239922	987.262037
CPD1810	3.4665573	230.161	CPD3358	20.056768	427.36809	CPD52742	9.78165048	1234.07818
CPD1811	3.481894	291.07724	CPD33580	8.17998974	792.115642	CPD52743	9.77779788	987.863764
CPD18154	5.51982803	301.19686	CPD33585	8.24974689	580.122137	CPD52744	9.78210222	1234.57841
CPD18169	5.60060905	292.104346	CPD33586	8.2521544	596.129485	CPD52745	9.77806111	1259.07061
CPD1819	4.3934813	327.12867	CPD33588	8.27133161	442.112047	CPD52746	9.78539828	1234.82871
CPD18200	5.88783528	572.7781	CPD33594	8.45958086	507.197425	CPD52747	9.71608548	1230.82498
CPD18216	6.09725653	388.230618	CPD3360	20.584318	331.32445	CPD5275	33.568702	78.93495
CPD1825	4.9615542	480.19592	CPD33603	8.75665618	972.260934	CPD52750	9.78700411	987.463786
CPD18250	5.97924668	390.152257	CPD33610	8.65659696	973.73689	CPD52751	9.82076772	1212.06264
CPD1827	5.0205175	354.09446	CPD33614	8.6712977	484.12814	CPD52753	9.7844615	981.05881
CPD18284	6.14799947	390.19112	CPD33618	8.75141808	695.166	CPD52754	9.8348912	1212.81272
CPD1829	5.0475843	187.06264	CPD3362	20.538454	398.05808	CPD52755	9.74838859	987.663645
CPD18295	6.62680925	539.223855	CPD33627	8.86613467	636.169663	CPD52756	9.80659859	973.453369
CPD18297	6.64205906	513.27926	CPD3363	20.583949	331.93542	CPD52757	9.83195815	1212.56369
CPD1832	5.1021425	324.14267	CPD33634	8.91586569	654.650347	CPD5276	33.535367	316.10082
CPD18329	6.755357	302.043519	CPD33636	8.92416533	1224.35113	CPD52760	9.79979217	1215.31706
CPD1833	5.1192607	189.03249	CPD3364	20.404746	428.31149	CPD52762	9.83041269	973.653994
CPD1834	5.1103985	306.16027	CPD33647	9.33396324	454.155143	CPD52763	9.82609946	1215.56519
CPD1835	5.1140718	116.08287	CPD3365	20.59564	168.18719	CPD52764	9.83275186	1241.55819
CPD1837	5.1108652	181.04369	CPD33653	9.14720691	470.1006	CPD52765	9.57999796	1239.31182
CPD18374	6.8013502	444.252872	CPD3366	20.588499	680.49045	CPD52766	9.72187906	1216.81224
CPD1838	4.6083586	348.11673	CPD33664	9.3337445	336.11995	CPD52767	9.82210233	972.05346
CPD1839	5.1377458	238.14344	CPD33667	9.3514565	533.213069	CPD52773	9.90434531	1226.32236
CPD18395	6.55014936	355.131062	CPD3367	20.597452	360.247	CPD52775	9.8463663	973.253753
CPD1840	5.1409685	255.16685	CPD33671	9.40377986	581.126467	CPD52781	9.94835077	542.304236
CPD1841	5.1688747	297.08891	CPD33674	9.35769525	338.1287	CPD52782	9.90120964	1226.07279
CPD18412	6.30311972	328.019783	CPD33678	9.45374699	625.215562	CPD52784	9.93312535	416.200369
CPD18413	6.95504923	350.103193	CPD3368	20.538429	169.11004	CPD52785	9.93879266	322.24894
CPD1842	5.3065886	252.12041	CPD33681	9.66979551	600.18154	CPD5279	33.543489	198.99238

CPD1843	5.3650786	230.16162	CPD33693	9.76630873	988.262171	CPD52791	10.0453083	542.303956
CPD18439	6.85171351	448.100725	CPD33694	9.79386801	1296.37101	CPD52792	10.1605072	1212.31275
CPD1844	5.0310655	355.09955	CPD3370	20.575649	187.12039	CPD52793	10.1278779	441.256456
CPD1845	5.4798569	125.08286	CPD33701	9.89219972	565.14308	CPD52794	10.1369347	231.088344
CPD1846	5.507094	154.14568	CPD33709	10.0750024	462.207517	CPD52796	10.1705079	479.25762
CPD18467	6.87730792	242.125322	CPD33712	10.0922038	902.253955	CPD52798	10.1417005	1212.81455
CPD1847	5.5327482	191.06214	CPD33714	10.2015479	411.476229	CPD5280	33.510493	285.88598
CPD18471	6.96374757	212.0714	CPD33718	10.2519239	416.80842	CPD52801	10.2621147	1226.32351
CPD18473	6.99109739	224.139544	CPD33719	10.2773161	497.677492	CPD52802	10.2713341	1226.57172
CPD1848	5.5474454	470.18945	CPD3372	20.5974	367.2134	CPD52803	10.0944467	981.45735
CPD1851	5.7140129	222.10881	CPD33721	10.24258	625.213533	CPD52809	10.3687134	323.194386
CPD18512	7.07868776	368.11095	CPD33722	10.2790008	1138.31443	CPD5281	33.587078	115.96138
CPD1853	5.8867252	130.06002	CPD33723	10.3755715	411.477143	CPD52810	10.4728004	453.25586
CPD1854	5.7107592	239.14029	CPD33728	10.5214174	1192.34645	CPD52814	10.9655497	551.19955
CPD18558	7.25179089	284.116753	CPD33735	10.6266551	1132.32329	CPD52815	10.9662753	556.154489
CPD18559	7.42981514	448.193592	CPD3374	20.59181	112.1244	CPD52816	10.9649464	326.15177
CPD1856	5.8076997	448.06012	CPD33740	10.629717	600.144143	CPD5283	33.434945	260.88396
CPD1857	6.1088749	372.10646	CPD33744	10.6360144	616.207717	CPD52836	13.2642284	1098.5971
CPD18576	7.4257604	331.2092	CPD33745	10.6445655	616.709856	CPD52839	13.9460997	1003.48826
CPD1858	5.8344477	192.04225	CPD3375	20.527419	154.10107	CPD5284	33.578004	249.96585
CPD18595	7.75207511	363.182837	CPD33751	10.872903	633.211227	CPD52840	14.8560848	305.243678
CPD1860	5.8139052	210.05582	CPD33752	10.8421472	633.711871	CPD52845	15.1973048	923.712564
CPD1861	5.799718	225.08166	CPD33755	10.9030638	422.143112	CPD52848	15.1944976	1397.42679
CPD1862	5.8226762	564.01624	CPD33759	10.9799982	468.172125	CPD52850	15.9651974	920.711812
CPD18621	7.72038125	475.137875	CPD3376	20.592694	351.23876	CPD52851	15.7911186	920.512625
CPD1863	5.8359318	404.08194	CPD33765	11.1525678	1338.38323	CPD52852	15.7580293	920.310222
CPD1865	5.8473544	470.03749	CPD33766	11.2296585	633.710214	CPD52859	16.4211002	212.14057
CPD18651	7.55249847	412.20745	CPD33768	10.933662	305.199114	CPD52861	16.4513148	166.066794
CPD1866	5.8176154	434.08431	CPD33776	11.2548083	416.808844	CPD52867	16.9483337	279.255642
CPD18668	8.29888193	527.293922	CPD33777	11.1879522	1000.28533	CPD52868	17.1579087	1392.0967
CPD1867	5.8286387	501.9799	CPD3378	20.598724	171.12396	CPD5287	33.240745	79.011647
CPD18672	7.91461991	378.025591	CPD33781	11.2523332	417.143733	CPD52870	17.4362732	279.255718
CPD1868	5.8317281	129.05702	CPD33787	11.2351156	703.675412	CPD52871	17.8026369	690.257955
CPD18687	8.07771814	854.217761	CPD3379	20.601537	388.30136	CPD52873	17.9389868	1387.43445
CPD1869	5.8249937	368.10385	CPD33792	11.3001031	1368.39379	CPD52874	17.9405806	1040.32571
CPD18697	8.08518134	1332.3575	CPD3380	20.595114	56.062929	CPD52875	17.9451846	1040.57496
CPD1870	5.8435047	206.05127	CPD33802	11.5638014	417.45772	CPD52876	17.9513377	1386.76556
CPD18705	8.19951065	304.08291	CPD33803	11.5640035	418.1256	CPD52879	17.9813596	1387.09976
CPD18706	8.16645764	305.089315	CPD33804	11.572818	417.791427	CPD52892	18.9076328	1258.92808
CPD1871	5.8595078	479.15013	CPD33807	11.5797489	626.687838	CPD52894	18.927541	1258.67564
CPD1872	5.8373236	352.10811	CPD33809	11.5741599	626.188183	CPD52903	19.4485367	279.255336
CPD18729	8.52195071	746.206671	CPD3381	20.634579	379.32484	CPD52914	21.0334284	793.462114
CPD18739	8.11976832	693.653122	CPD33812	11.5991769	474.15219	CPD52915	21.0341489	771.469515
CPD1874	5.8531972	486.00845	CPD33815	11.6638535	504.183044	CPD5292	33.642694	68.988682
CPD18743	8.12202025	685.156076	CPD33821	11.6672868	503.678636	CPD52924	21.7229758	529.844517
CPD18757	8.11847021	677.664218	CPD33828	11.741601	439.2396	CPD5295	33.462149	76.008843
CPD18764	8.58376523	456.230333	CPD3384	20.548324	362.30228	CPD52966	24.7413314	717.533222
CPD1877	5.8161104	190.05598	CPD33840	11.9564179	625.714608	CPD52973	25.2814579	474.402
CPD18779	8.01756497	338.100787	CPD33841	11.9726207	625.213329	CPD5299	33.555449	163.96572
CPD1878	5.8651307	548.04365	CPD33842	11.9868806	416.808692	CPD53007	31.6019351	830.469729
CPD1879	5.8478807	350.09271	CPD33844	12.0158318	562.132461	CPD5301	33.557039	180.97998
CPD18797	9.06940681	477.116793	CPD3385	20.661143	946.52497	CPD5303	33.558321	258.88697
CPD18801	8.46753928	182.091614	CPD33855	12.0975969	576.14841	CPD5305	33.542347	106.01337
CPD1881	5.8697361	772.20451	CPD33857	12.1024498	488.67342	CPD5310	33.582165	89.110796
CPD18819	9.17203527	432.197082	CPD33858	12.0726358	489.17607	CPD5313	33.611735	201.00616
CPD1882	5.937998	405.07712	CPD33859	12.1077584	417.79416	CPD5314	33.536229	189.97171
CPD18821	9.10754542	462.12066	CPD3386	20.629212	520.34965	CPD53201	1.04572497	429.98389
CPD18826	9.01879225	219.161442	CPD33860	12.1292691	417.461377	CPD53222	1.07928576	358.057614
CPD18829	9.15199718	432.196942	CPD33862	12.1505	626.19065	CPD53223	1.06342845	394.004086
CPD1883	5.9152607	447.18609	CPD33863	12.1501855	626.689509	CPD53231	1.08871432	628.988443
CPD1884	5.9492499	794.185	CPD33864	12.1827343	418.124288	CPD53232	1.09744362	584.077833
CPD18846	9.56271956	159.068757	CPD33865	12.215799	410.80522	CPD53236	1.12862501	378.434675
CPD18848	9.80678355	588.132643	CPD33874	12.2964647	977.345033	CPD53237	1.12676938	378.263938
CPD1885	5.9196606	113.08369	CPD3388	20.624842	265.23703	CPD53245	1.28513346	409.157373
CPD1886	5.9598495	448.09849	CPD33884	12.4239999	642.202378	CPD53248	1.3047167	521.119829
CPD1889	5.9532718	296.14664	CPD33885	12.4327157	642.6998	CPD5325	33.641108	175.97899
CPD18906	11.2900797	711.159025	CPD3389	20.726232	283.32324	CPD53250	1.31854661	575.062362

CPD18909	11.5592444	274.192164	CPD33890	12.7207021	504.1812	CPD53251	1.31761126	573.084194
CPD1891	5.984598	934.25574	CPD33892	12.7302983	494.673525	CPD53253	1.32036349	405.010336
CPD18915	11.9122665	298.103196	CPD33893	12.7575008	420.106717	CPD53254	1.32625012	657.053662
CPD18918	12.4024544	662.222169	CPD33897	12.8403795	411.141538	CPD53256	1.31238458	366.165092
CPD18919	12.2625226	210.123608	CPD33898	12.835569	410.804986	CPD5327	33.612126	128.99781
CPD1892	5.9965053	216.14909	CPD33909	13.1304999	486.676	CPD53270	1.56409088	204.132736
CPD18921	12.4699826	662.223691	CPD33911	13.4069648	234.162125	CPD53273	1.56879984	305.085853
CPD1893	5.9895515	162.06544	CPD33912	13.3691684	503.190592	CPD53275	1.57091656	199.047383
CPD18939	14.8434211	616.223682	CPD33913	13.3789997	509.2156	CPD53277	1.58594596	364.183344
CPD18940	15.0076197	508.264733	CPD33916	13.5387226	325.307955	CPD53278	1.58149991	428.180336
CPD18941	14.9431903	519.25966	CPD33917	14.3611637	381.2285	CPD53279	1.5153505	128.130129
CPD1895	5.9965338	412.13441	CPD33919	13.6396652	487.177611	CPD53282	1.58387519	434.065312
CPD18951	15.2078751	292.20508	CPD3392	20.728479	979.661	CPD5329	33.471297	78.935369
CPD18953	15.1733319	496.30268	CPD33920	13.7044921	471.670887	CPD53292	1.69263627	374.032209
CPD18966	15.8656989	554.285033	CPD33924	13.8665996	517.69409	CPD53294	1.69609209	264.276018
CPD18968	16.0020879	180.115844	CPD33926	13.781749	480.172321	CPD53295	1.6891	297.03186
CPD1897	6.035023	282.16657	CPD33927	13.9195454	542.685273	CPD5330	33.626113	167.00732
CPD18991	16.5887619	536.49604	CPD33929	13.9381986	657.24237	CPD53301	1.80953544	364.182387
CPD18993	16.8467707	386.260144	CPD3393	21.166612	477.31744	CPD53310	1.70107979	264.132683
CPD1901	6.038306	268.09478	CPD33930	13.9703751	510.686688	CPD53322	2.12390846	109.001622
CPD19016	17.4356667	454.041117	CPD33931	14.1020455	471.671	CPD53336	2.10706939	364.2433
CPD19017	17.3793257	457.303108	CPD33934	14.1821182	510.686218	CPD53343	2.07398326	585.136562
CPD19019	17.3204466	658.262404	CPD3394	20.70081	318.27653	CPD53349	2.1141214	532.154513
CPD1902	6.0923789	219.04071	CPD33940	14.3579486	480.676123	CPD53355	2.10257638	458.2456
CPD19020	17.6225552	292.204145	CPD33943	14.3756995	487.17739	CPD53357	2.12189126	321.204244
CPD19022	17.4568286	404.257044	CPD33944	14.3769992	486.676071	CPD53363	2.10957573	596.151933
CPD19025	17.755514	335.240145	CPD33945	14.3984538	335.246255	CPD53364	2.14535337	599.161357
CPD1903	6.0950956	357.07377	CPD33946	14.4070617	481.176339	CPD53365	2.13705245	364.180583
CPD19032	17.8367023	278.223794	CPD33948	14.5538337	518.196417	CPD53367	2.15306827	705.189821
CPD1904	6.0945031	437.0228	CPD33949	14.5541338	517.694467	CPD53369	2.13282881	617.144075
CPD19058	18.7242485	504.2864	CPD3395	20.712026	622.27787	CPD53372	2.16106705	524.668453
CPD1906	6.0933003	584.08166	CPD33953	14.7606998	344.08982	CPD53374	2.17350794	640.731
CPD19078	19.34	554.30645	CPD33955	14.8648685	306.198645	CPD53376	2.16374977	506.391688
CPD19080	19.3430001	538.332857	CPD33958	14.6913238	448.375178	CPD53379	2.17237516	506.469625
CPD19081	19.3477269	294.159845	CPD3396	20.743584	500.82327	CPD53380	2.16131586	397.12
CPD19083	19.3472688	288.14212	CPD33969	15.0329324	517.694533	CPD53389	2.18466703	56.0259667
CPD19084	19.3455007	304.116567	CPD3397	20.74669	498.84684	CPD53398	2.23627273	571.2133
CPD19086	19.4819637	892.4713	CPD33970	15.039054	518.196373	CPD5341	33.611507	99.004174
CPD19089	19.5904283	379.227	CPD33981	15.399111	457.169589	CPD53412	2.51437194	73.0267429
CPD1909	6.0865996	433.04293	CPD33984	15.441401	494.67548	CPD53416	2.36676659	426.184206
CPD19099	19.8185806	334.250242	CPD33989	15.9723792	395.243275	CPD53421	2.42833387	294.094889
CPD19103	19.835705	408.264581	CPD33992	15.6527006	444.344044	CPD53454	4.56878248	256.99825
CPD19104	19.8576548	442.231956	CPD33993	15.9197288	551.22595	CPD53459	4.65940022	278.29428
CPD19105	19.8162269	374.243238	CPD33996	15.9457779	466.174456	CPD53461	4.68625018	256.9981
CPD19106	19.7497753	414.1886	CPD33998	16.0539574	471.670892	CPD53462	4.67983824	356.048763
CPD1912	6.0481537	146.03712	CPD3400	20.756889	366.25598	CPD53466	5.3639255	393.162285
CPD19123	20.3163265	612.258668	CPD34000	16.1983756	487.179737	CPD5347	33.555248	76.008052
CPD19127	20.5294295	234.195329	CPD34001	16.1908796	486.675625	CPD53470	5.72037528	276.304413
CPD19129	20.5754215	360.248961	CPD3401	20.779033	379.32588	CPD53473	5.88632397	325.134
CPD19130	20.7791041	578.46136	CPD34011	16.5898446	541.280986	CPD53475	5.90272965	222.103789
CPD19142	21.0903344	698.235833	CPD34012	16.4920925	533.305327	CPD53482	6.15571207	308.326743
CPD19146	21.1002912	182.130343	CPD34013	16.4681871	705.82525	CPD53485	6.41977734	674.180833
CPD19148	21.0862772	242.203244	CPD34016	16.5881582	432.311686	CPD53486	6.46371416	433.123521
CPD1915	6.0525959	408.06545	CPD3402	20.786116	196.11264	CPD53488	6.51699013	276.3054
CPD19150	21.0969296	578.414821	CPD34023	17.0431322	413.253586	CPD53491	6.64214872	402.116662
CPD19155	21.089111	594.392572	CPD34028	17.1823334	400.283133	CPD53494	6.69265241	348.082082
CPD19157	21.1001108	982.5316	CPD3403	20.763274	348.24555	CPD53497	6.7708177	626.365233
CPD1916	6.2572219	438.02575	CPD34031	17.3120003	911.74082	CPD53498	6.78243024	284.089814
CPD19162	21.134141	338.1644	CPD34032	17.3138458	911.607169	CPD53499	6.88343573	347.120875
CPD19166	21.2015341	262.230304	CPD34033	17.3169995	456.8627	CPD53502	7.04378596	1156.30997
CPD1917	6.3351492	384.10627	CPD34034	17.332603	467.167822	CPD53504	6.86821862	306.878425
CPD1918	6.0929946	422.05175	CPD34035	17.3953845	479.1825	CPD53508	6.96099208	812.69322
CPD1919	6.1028598	338.09945	CPD3404	20.851963	204.09846	CPD53509	6.99900756	812.85994
CPD1920	6.0953586	287.00857	CPD34040	17.5110015	768.214442	CPD5351	33.622713	159.97963
CPD19202	21.7214002	366.27751	CPD34043	17.5457148	246.069186	CPD53518	7.02924928	701.232925
CPD1921	6.1847405	424.09664	CPD34044	17.7520553	508.279531	CPD53520	7.05837076	546.170687
CPD19215	22.1829176	264.209042	CPD3405	21.003765	816.46058	CPD53523	7.09936133	788.195936

CPD1923	6.2746291	419.14129	CPD34058	18.1538583	668.447871	CPD53524	7.12559399	1074.87619
CPD19231	22.6343538	624.266991	CPD3406	20.799599	354.27727	CPD53525	7.15166684	1006.03955
CPD19239	22.7975546	1180.50433	CPD34066	18.5327205	411.333963	CPD53526	7.14648504	1008.43431
CPD19246	22.8810787	492.826258	CPD34070	18.1097509	351.219757	CPD53527	7.14958251	1005.43843
CPD1926	5.7432184	322.10099	CPD34072	18.519088	367.193167	CPD53528	7.14600017	841.030073
CPD19269	23.2010173	664.450146	CPD34075	18.4953751	388.283429	CPD53529	7.14638787	838.032731
CPD19274	23.6090331	567.43419	CPD34080	18.8694116	745.342271	CPD53530	7.13507213	835.039067
CPD1928	6.171222	1288.3506	CPD34083	19.1217611	506.264675	CPD53532	7.15476061	838.200056
CPD19289	24.3758608	757.560362	CPD34084	18.9947754	689.4256	CPD53533	7.15099532	1005.64033
CPD19290	24.7697742	666.446975	CPD3409	20.800168	336.27066	CPD53535	7.16969351	278.078377
CPD1930	6.1942579	516.14624	CPD34092	19.1937079	209.143071	CPD53537	7.14856711	1005.83894
CPD19308	25.6737561	1018.53393	CPD34098	19.350198	439.206627	CPD53539	7.26159719	1042.65783
CPD19315	26.0079999	811.5016	CPD3410	20.805732	262.23089	CPD53546	7.46250058	832.706475
CPD1932	6.1959178	350.15868	CPD34100	19.378706	441.183076	CPD53550	7.41772852	439.245055
CPD19320	26.3932068	569.484791	CPD34106	19.5556913	650.496908	CPD53552	7.40796488	999.645656
CPD19325	26.7575306	680.464087	CPD3411	20.794632	371.31075	CPD53555	7.51208735	297.089555
CPD1933	6.1521012	296.1464	CPD34116	19.7510782	351.27703	CPD53556	7.41775176	999.247267
CPD1934	6.194278	318.14074	CPD34119	19.6187178	892.462386	CPD53557	7.52756535	278.078483
CPD1935	6.220618	222.10885	CPD3412	20.929225	388.26171	CPD53559	7.50738044	1011.0544
CPD19350	28.3523816	864.498221	CPD34125	19.765856	393.271457	CPD53560	7.60779259	842.20985
CPD1936	6.4173397	567.28658	CPD34127	19.8924447	841.635433	CPD53563	7.66577353	516.095488
CPD19362	29.408954	686.50972	CPD3413	20.823945	274.25039	CPD53564	7.7107852	957.26042
CPD19374	30.3006187	937.136092	CPD34137	20.1466237	369.287317	CPD53565	7.691257	845.212269
CPD1938	6.1276998	244.09218	CPD34138	19.9666249	842.482675	CPD53571	7.94251893	864.737856
CPD1939	6.2457782	440.22636	CPD3414	20.83245	935.63704	CPD53572	7.85458535	1002.04944
CPD19392	32.868296	747.571223	CPD34150	20.3757	734.00766	CPD53573	7.86576243	864.07884
CPD1940	6.0889722	540.14479	CPD34153	20.2718622	488.337471	CPD53575	7.84599568	1283.12792
CPD1942	6.3208025	486.13622	CPD3416	20.83503	506.67625	CPD53577	7.86821032	1071.1103
CPD19427	33.1081966	222.981744	CPD3417	20.844284	236.21343	CPD53579	7.89490188	835.707727
CPD1943	6.2925018	278.05795	CPD3418	20.85106	253.24081	CPD53580	7.83941939	641.688017
CPD1944	6.4445855	196.07417	CPD34183	20.7268744	350.250288	CPD53583	7.89122192	546.205959
CPD19440	33.6742096	125.968373	CPD34190	20.9897499	353.292887	CPD53585	7.80448154	831.876322
CPD1945	6.1586336	222.10767	CPD34192	21.1329443	726.545728	CPD53586	7.9908293	856.910856
CPD1948	6.3886344	964.24592	CPD3420	20.843137	166.10686	CPD53590	8.05143978	742.646767
CPD1950	6.3478996	386.12304	CPD34200	21.3790215	359.282567	CPD53597	8.08931014	1364.70034
CPD19512	1.09132644	372.976507	CPD34201	21.4898	666.26883	CPD53599	8.20977064	964.24201
CPD1953	6.5583267	586.12831	CPD34202	21.551	756.589956	CPD53612	8.1828928	781.805078
CPD19534	1.02157906	366.982226	CPD34205	21.5884285	355.2772	CPD53614	8.20152845	878.2157
CPD19536	1.19601641	424.04148	CPD34209	21.601477	349.317665	CPD53616	8.30690918	1033.49893
CPD1954	6.3710718	632.12533	CPD3421	20.836075	478.81086	CPD53617	8.23933283	861.41676
CPD19548	1.02961547	201.021292	CPD34219	21.9482964	456.348989	CPD53621	8.20916715	1319.87194
CPD19549	1.16121605	200.029033	CPD3422	20.856399	353.16799	CPD53622	8.2044111	1074.90328
CPD1955	6.3639965	346.10628	CPD34227	22.0040036	355.308629	CPD53624	8.18138225	912.4342
CPD19551	1.03791681	214.044472	CPD3424	20.903954	654.38457	CPD53625	8.1930011	879.244818
CPD1956	6.3654065	570.15502	CPD34243	22.3387499	671.510387	CPD53627	8.19908353	1075.10089
CPD1959	6.3703069	543.19313	CPD34244	22.3497001	676.46669	CPD53628	8.20574462	1279.37609
CPD19595	1.04598688	671.80635	CPD34247	22.4365715	632.440636	CPD53629	8.21445762	1075.49225
CPD1960	6.3457225	565.19935	CPD3425	20.848785	158.11906	CPD53631	8.23821111	827.55928
CPD1961	6.4007544	224.0683	CPD34259	22.4493634	741.617827	CPD53632	8.23869429	1012.46207
CPD1962	6.3707461	548.16693	CPD3427	20.88168	638.27358	CPD53633	8.16009448	742.35985
CPD1963	6.3746031	336.01432	CPD34270	22.4608788	374.280806	CPD53634	8.20679001	1068.30489
CPD19632	1.04159987	707.74116	CPD34274	22.5518891	944.6472	CPD53635	8.19416523	895.751033
CPD1964	6.4080733	602.09233	CPD3428	20.938131	896.56543	CPD53636	8.1877704	868.422054
CPD19667	1.06462004	711.780213	CPD34285	22.5500908	814.9223	CPD53638	8.20064453	890.423021
CPD1967	6.3241039	575.12078	CPD3429	20.927944	600.41668	CPD53639	8.18274001	639.43915
CPD1968	6.4585349	206.0583	CPD3430	20.951952	891.611	CPD53642	8.21884663	781.943185
CPD1971	6.3150545	293.06034	CPD34312	22.817666	451.368	CPD53643	8.25649519	1012.06283
CPD19717	1.06203949	494.95398	CPD34314	22.824201	594.48288	CPD53651	8.2272288	861.416033
CPD19718	1.09279956	528.03674	CPD34318	22.9094442	738.881567	CPD53652	8.28788355	854.748811
CPD1972	6.6446298	1096.2897	CPD34324	23.248816	650.2726	CPD53655	8.36715322	863.420718
CPD19727	1.12294915	501.113983	CPD34325	22.9581878	548.349281	CPD53662	8.37998679	1033.70525
CPD1973	6.3955256	354.09483	CPD34335	23.1925604	764.661162	CPD53666	8.4250965	1034.11667
CPD1974	6.3752822	189.04275	CPD34340	23.1698177	1299.57175	CPD53669	8.42855441	963.285811
CPD19741	1.15	179.06307	CPD34348	23.2543307	791.028211	CPD53670	8.43341257	972.675425
CPD19747	1.16227775	221.091111	CPD3435	21.053288	800.47	CPD53671	8.47037729	964.867662
CPD1975	6.4003206	157.1091	CPD34354	23.3239473	828.742869	CPD53676	8.39605313	860.753611
CPD19754	1.15599927	387.132965	CPD3436	20.947082	618.42756	CPD53679	8.32784993	1034.30932

CPD1976	6.4118964	324.09628	CPD34360	23.3311245	831.552088	CPD53684	8.44930499	814.214155
CPD19761	1.29149065	362.166955	CPD34361	23.3527311	608.437708	CPD53687	8.4257563	863.251267
CPD19763	1.04883481	139.004429	CPD34363	23.3347497	507.308937	CPD53688	8.42757285	1027.10127
CPD19768	1.28999868	84.02055	CPD34364	23.3245256	828.97164	CPD53689	8.45100366	976.25487
CPD1977	6.6494372	934.23567	CPD34365	23.3524659	712.45984	CPD53690	8.44576744	808.394808
CPD19774	1.29712271	96.02044	CPD34370	23.447	822.6316	CPD53691	8.44610513	864.08158
CPD19775	1.5524334	201.994315	CPD34371	23.2756121	812.903663	CPD53692	8.47987616	414.214178
CPD1978	6.3985836	159.12404	CPD3438	20.950997	456.79768	CPD53695	8.46294189	1026.49996
CPD1979	6.3915009	448.10043	CPD34384	23.5886625	410.3006	CPD53701	8.5305649	312.08515
CPD19790	1.59754886	115.062644	CPD3439	20.966037	632.27949	CPD53703	8.50425873	989.833291
CPD19799	1.52194357	512.115327	CPD34393	23.5876459	483.428775	CPD53704	8.51662411	1295.09616
CPD1980	6.4588605	317.14846	CPD34399	23.7909104	431.779013	CPD53705	8.70266289	977.246113
CPD1982	6.5315581	162.0327	CPD3440	20.812851	366.25806	CPD53711	8.47550166	1221.3129
CPD19823	1.64759075	362.1678	CPD34402	23.7979279	952.574527	CPD53716	8.50290898	809.03074
CPD19825	1.80840898	220.082525	CPD3441	20.855043	479.31609	CPD53717	8.5444953	1305.88519
CPD19840	1.85496631	220.082238	CPD34410	23.8170909	1021.70732	CPD53718	8.48343474	1044.70989
CPD1985	6.4826062	357.14018	CPD34414	23.9317781	826.464089	CPD53720	8.53944061	1642.39836
CPD19853	1.98507095	103.099175	CPD3442	20.975546	448.28281	CPD53723	8.53866077	958.285556
CPD1986	6.4844481	373.11369	CPD34421	24.1647291	615.3126	CPD53724	8.54886198	1045.50959
CPD1987	6.4408574	438.14841	CPD34426	24.2830034	950.565591	CPD53725	8.54979557	638.34794
CPD1988	6.3232801	406.09617	CPD34437	24.5782946	990.534147	CPD53728	8.56675226	1317.56888
CPD19883	3.08361111	333.105028	CPD34439	24.8378889	938.730089	CPD53731	8.56799773	1054.05531
CPD1989	6.5982784	530.16241	CPD34452	25.0688261	609.505012	CPD53733	8.56399814	1316.81978
CPD19890	3.42112974	338.075887	CPD3446	21.072872	832.45533	CPD53734	8.48125262	867.075486
CPD1990	6.5993726	678.64709	CPD34470	25.7764281	472.421836	CPD53743	8.60635756	865.592486
CPD19906	4.84647742	391.143918	CPD34471	25.805992	456.4398	CPD53750	8.66153015	820.228683
CPD1991	6.5244301	670.66039	CPD34476	26.3093009	826.519214	CPD53754	8.70235794	1074.87407
CPD1992	6.5204946	1302.3671	CPD3448	21.047098	852.53694	CPD53765	8.73935537	911.401259
CPD19921	5.65820371	626.28804	CPD34483	27.3346239	850.492006	CPD53766	8.95148706	1050.68138
CPD1993	6.793072	670.15574	CPD34485	27.5578736	680.46285	CPD53770	8.73924385	912.065725
CPD1994	6.5274373	678.14501	CPD34487	27.4759962	868.425038	CPD53772	8.79612128	1219.06279
CPD19967	6.44602987	408.102461	CPD34494	27.738323	892.555322	CPD53773	8.73970955	911.899943
CPD19968	6.44482665	424.085083	CPD34502	28.0570089	551.485243	CPD53778	8.79815836	991.252318
CPD1997	6.5496157	565.19991	CPD3451	21.045916	847.58356	CPD53780	8.83024381	999.043245
CPD19972	6.50349735	321.08501	CPD34516	29.3607524	463.410388	CPD53790	8.79296545	1033.87554
CPD1998	6.5977221	662.17561	CPD3452	21.052155	654.26856	CPD53791	8.76518869	1039.87957
CPD1999	6.5556459	404.20949	CPD3455	21.032884	816.46103	CPD53795	9.01264934	993.83913
CPD2000	6.4997283	224.06838	CPD34555	33.1793712	69.00345	CPD53796	8.84821627	999.242725
CPD20003	7.47756986	416.225706	CPD3456	21.103763	158.08802	CPD53802	9.0297894	997.440829
CPD2001	6.4975631	456.10458	CPD3457	21.101814	278.22452	CPD53803	8.77154302	1299.59762
CPD20013	7.22062904	479.147195	CPD3458	20.730384	388.29321	CPD53809	8.80622618	812.869445
CPD20018	7.05049592	408.171543	CPD3461	21.108935	322.19033	CPD53811	8.76755735	1034.47291
CPD2002	6.528638	293.0625	CPD34610	1.00909994	144.137197	CPD53813	9.18915202	1000.05059
CPD20020	7.18299334	499.195483	CPD3462	20.993619	376.26244	CPD53818	8.8062234	1131.32632
CPD2003	6.9376565	356.11011	CPD3463	21.137308	803.55575	CPD53820	8.81199941	982.841933
CPD20034	7.47819149	208.111783	CPD3464	21.104388	670.30426	CPD53821	8.81608465	976.852109
CPD20039	7.5475136	232.15767	CPD3465	21.107736	384.15509	CPD53823	8.82269722	975.049458
CPD2004	6.5416292	361.08606	CPD3466	21.214243	352.34214	CPD53825	8.81970043	816.707717
CPD2005	6.5551044	570.15497	CPD3468	21.205782	253.24049	CPD53829	8.77800595	1292.83979
CPD20051	7.65558003	337.0823	CPD3469	21.473772	582.84765	CPD53832	8.83492705	816.0411
CPD20052	7.67311605	135.055843	CPD34690	1.11616664	886.977158	CPD53833	8.81506468	1046.28185
CPD20053	7.61412415	264.112771	CPD34700	1.18092693	582.146528	CPD53834	8.83647526	1652.08271
CPD20059	7.73248445	208.11023	CPD34705	1.13324988	735.07515	CPD53835	8.81455971	1627.08512
CPD2006	6.5676085	362.15363	CPD3471	21.284601	263.22328	CPD53836	8.83140785	1220.56596
CPD20069	7.94825615	192.07955	CPD34711	1.70178667	293.173058	CPD53842	8.82466755	1307.10444
CPD2007	6.6067964	368.11028	CPD34718	1.27775202	202.046728	CPD53845	8.86268057	979.051536
CPD20075	8.01554703	350.141344	CPD34728	1.35244289	435.044425	CPD53849	8.85663399	1636.40736
CPD20079	8.10103102	452.784131	CPD3473	21.191825	299.27854	CPD53856	8.86082666	991.053418
CPD20081	8.17702522	503.0913	CPD3474	21.246001	759.53375	CPD53859	8.89618657	1636.57924
CPD20090	8.17220606	659.09584	CPD3476	21.225247	800.4695	CPD53861	8.85476452	1244.55282
CPD20091	8.17950323	519.07235	CPD34761	1.56771281	364.1048	CPD53864	8.95970638	811.704875
CPD20092	8.3361781	477.110125	CPD3477	21.273276	780.46733	CPD53866	9.17454533	1221.30899
CPD20096	8.17019772	629.15306	CPD34774	1.49623942	151.040617	CPD53869	8.89461589	1249.30242
CPD20097	8.17900354	604.07775	CPD3478	21.29861	617.89714	CPD53870	9.00906261	974.044063
CPD20098	8.17259997	694.13922	CPD3480	21.435144	243.25262	CPD53871	8.90294283	1252.79421
CPD2010	6.5585931	176.04695	CPD3482	21.461956	582.40674	CPD53876	8.82146983	1217.7841
CPD20100	8.1615574	332.053036	CPD3483	21.483063	582.61561	CPD53883	8.90067666	991.252436

CPD20102	8.19014672	535.041471	CPD34832	5.7440218	369.107288	CPD53886	8.99125329	980.655475
CPD20103	8.18885297	324.068043	CPD3484	21.283536	263.22336	CPD53889	9.1297862	1043.07844
CPD20107	8.19657221	306.2475	CPD34846	6.3317995	343.125988	CPD53890	9.09623942	1049.2851
CPD20108	8.19741905	317.09244	CPD3485	21.699852	429.37894	CPD53891	8.9110623	999.442233
CPD20111	8.17639692	327.23044	CPD34858	6.74655812	264.064611	CPD53892	8.9221055	1001.83615
CPD20114	8.21502416	434.14795	CPD3486	21.376274	341.32796	CPD53893	8.84159093	995.850254
CPD2012	6.5584624	319.1195	CPD34862	6.94870146	230.043225	CPD53896	8.9882368	817.716145
CPD20120	8.18416187	184.04085	CPD34870	6.92016587	372.105412	CPD53899	9.01707689	1054.28609
CPD20124	8.19685329	389.042771	CPD34873	7.29883228	472.19555	CPD53901	9.01484577	815.545507
CPD20128	8.20966818	350.14745	CPD3488	21.350699	715.50746	CPD53906	9.02063238	815.213918
CPD20129	8.20486211	239.028857	CPD34888	8.08706548	456.196091	CPD53907	8.998921	1247.30433
CPD20132	8.22472143	264.112743	CPD3489	21.351136	720.46229	CPD53908	9.02314203	786.8718
CPD20135	8.53616986	918.246074	CPD34890	7.9426995	530.145767	CPD53911	9.03330123	980.4631
CPD20137	8.23557401	917.742457	CPD34895	7.80985335	1000.20703	CPD53914	9.05669722	1307.59896
CPD20138	8.16376681	934.228044	CPD3490	21.382995	595.88405	CPD53915	9.02805582	1046.28431
CPD20140	8.24250101	933.72186	CPD34917	8.23408404	416.161778	CPD53916	9.07807378	1046.47954
CPD20144	8.3036986	208.112494	CPD34922	8.2469006	742.169054	CPD53918	9.01128304	1045.6812
CPD2015	6.6103341	338.09988	CPD34925	8.38907981	444.162162	CPD53919	9.05560042	1307.85515
CPD20150	8.47078563	696.213178	CPD34926	8.48892201	528.155538	CPD53920	9.09242889	1223.06667
CPD20152	8.42147829	398.169945	CPD34929	8.48257642	264.0757	CPD53921	9.46834379	822.71704
CPD2016	6.5739444	326.19293	CPD3493	21.37704	593.40602	CPD53924	9.29715711	985.456
CPD20169	8.25790918	826.38938	CPD34938	8.72822522	1300.35049	CPD53925	9.08959625	1041.46601
CPD2017	6.5762515	610.15518	CPD34945	8.87203686	466.239017	CPD53926	9.0244597	995.051567
CPD20172	8.79600592	380.158588	CPD3495	21.3875	228.20741	CPD53927	9.10599142	1041.07075
CPD20175	8.5012954	396.1562	CPD34950	8.86111215	403.14935	CPD53930	9.0989876	1041.26609
CPD20177	8.8287214	340.131394	CPD34952	8.88138313	310.123277	CPD53931	9.07892773	1041.66614
CPD20179	8.87842929	411.178186	CPD3497	21.394204	210.19667	CPD53932	9.09587648	1302.08234
CPD2018	6.5768088	391.14725	CPD3498	21.410627	374.25966	CPD53934	9.09987437	1056.27662
CPD20181	8.98784348	472.067017	CPD3499	21.342282	824.534	CPD53935	9.09466521	1301.5811
CPD2019	6.6443097	283.16243	CPD34994	10.3472948	208.11081	CPD53940	9.08310174	1320.83813
CPD20190	9.22721959	208.113	CPD3500	21.471304	520.49346	CPD53941	9.00228147	977.848591
CPD20192	9.21740888	511.1931	CPD35001	10.4722541	800.2169	CPD53943	9.10640871	985.2594
CPD20193	9.17454617	365.16896	CPD35003	10.5678174	1108.30643	CPD53944	9.20840504	1205.87843
CPD2020	6.7140052	663.14257	CPD35004	10.6178854	970.2754	CPD53945	9.14388082	986.831694
CPD20201	9.30785597	792.306614	CPD35014	11.210993	476.1516	CPD53946	9.11043817	1056.07617
CPD20205	9.39738213	762.30475	CPD35016	11.2433637	467.146082	CPD53947	9.19791903	1047.48401
CPD2021	6.6034764	266.13667	CPD35017	11.2445736	625.214186	CPD53948	9.1766356	1644.7154
CPD20213	9.59244481	302.1264	CPD35019	11.3169926	474.152427	CPD53950	9.14107707	987.032723
CPD20216	9.63340656	258.11628	CPD3502	21.430632	532.49217	CPD53951	9.21981544	1055.47403
CPD20217	9.63739633	497.16254	CPD35029	12.0937241	459.156442	CPD53952	9.1675327	986.433
CPD2022	6.6583011	655.65512	CPD3503	21.452094	197.174	CPD53955	9.14958615	1644.38577
CPD20222	9.70590947	129.057262	CPD35031	12.2826402	636.171893	CPD53958	9.14271737	973.439091
CPD20223	9.80120444	192.0312	CPD35036	12.9601986	473.17954	CPD53961	9.15038193	993.433215
CPD20226	9.81173671	792.317912	CPD35040	13.2449268	466.678614	CPD53962	9.13518346	1243.55936
CPD20227	9.80310382	652.27257	CPD35046	14.0322541	511.188736	CPD53968	9.16428083	1305.60951
CPD2023	6.6543057	1272.3563	CPD3505	21.448587	558.71692	CPD53970	9.1467779	1645.04902
CPD20231	9.88800647	614.26952	CPD35052	14.419751	481.177585	CPD53971	9.15641873	1243.80899
CPD20234	9.95281869	525.211036	CPD35069	14.9551205	1034.02647	CPD53973	9.16421564	1045.08943
CPD20237	9.92484429	122.060433	CPD3507	21.44644	558.51182	CPD53974	9.09478168	973.841992
CPD2024	6.6650973	647.16667	CPD35073	14.9429229	1044.36548	CPD53977	9.0604591	1222.30064
CPD2025	6.6692643	500.15219	CPD35076	14.8601871	1003.49216	CPD53978	9.0098851	1059.2778
CPD20250	10.0563657	333.146617	CPD3508	21.476579	244.21887	CPD53979	9.14975453	1222.8051
CPD20252	10.0168034	218.091731	CPD3510	21.458705	141.11441	CPD53980	9.18483401	982.042941
CPD20257	10.1870323	646.262178	CPD35104	16.015777	373.249611	CPD53985	9.20802614	1300.59366
CPD2026	6.663626	655.15281	CPD35109	16.2427709	315.219789	CPD53992	9.22972009	1062.47265
CPD20266	10.2588529	159.0689	CPD3511	21.448051	766.14758	CPD53995	9.36055492	976.64148
CPD2027	6.7170207	589.63398	CPD3512	21.460108	155.13105	CPD53997	9.25194023	915.0253
CPD20271	10.2578621	584.259729	CPD3513	21.452766	301.23878	CPD53998	9.32859609	1174.53077
CPD20277	10.3070038	389.109617	CPD35137	16.7059573	575.366483	CPD54007	9.36392237	935.035192
CPD2028	6.7782807	597.12219	CPD3514	21.457485	671.48116	CPD54011	9.4309757	1243.81059
CPD20280	10.3649968	415.188643	CPD35157	16.821862	369.228883	CPD54012	9.39600031	965.245967
CPD20281	10.3972146	394.12804	CPD3516	21.415332	262.22996	CPD54015	9.17119133	1246.05748
CPD20282	10.3840076	230.092433	CPD35162	17.3164327	697.32254	CPD54016	9.3575493	1262.56784
CPD20283	10.3907433	188.0791	CPD35164	17.3211683	376.281914	CPD54023	8.86276604	980.252766
CPD20285	10.5891651	376.1168	CPD3517	21.46426	317.21358	CPD54024	9.43280503	1249.55394
CPD20286	10.2762411	616.251329	CPD35184	17.8366001	738.19498	CPD54028	9.45366929	839.545789
CPD20289	10.4447826	692.262833	CPD35188	17.8974284	473.314457	CPD54030	9.48198066	1244.06334

CPD2029	6.5277856	386.12229	CPD35193	17.7976339	351.219845	CPD54031	9.46406662	1247.80639
CPD20293	10.6314313	678.281357	CPD35221	19.1578999	448.250708	CPD54033	9.43732985	1014.64663
CPD20296	10.7103336	453.189633	CPD3523	21.460144	363.20939	CPD54035	9.49063987	1018.03834
CPD20299	10.9027767	660.272033	CPD35234	19.3452998	525.34529	CPD54037	9.35010798	972.851329
CPD2030	6.6856098	1140.3151	CPD3524	21.462634	414.03522	CPD54038	9.1891381	972.455986
CPD20303	10.9253866	315.135025	CPD35266	20.4270656	357.79995	CPD54046	9.53499187	1213.0437
CPD20304	10.9233387	334.10865	CPD35268	20.4277297	357.84505	CPD54048	9.10271353	996.04407
CPD20306	10.9753556	602.273441	CPD35270	20.3841248	715.0224	CPD54049	9.43681985	1239.5581
CPD20309	11.1364431	600.2576	CPD3528	21.48301	573.8707	CPD54052	9.54966361	967.039933
CPD2031	6.9090545	589.13815	CPD35281	20.3596283	472.336292	CPD54053	9.53379859	1075.09914
CPD20312	11.230561	646.270767	CPD35289	20.7306921	503.355892	CPD54054	9.5475623	1212.79174
CPD20319	11.3103702	619.255029	CPD3529	21.477893	710.08445	CPD54056	9.542539	970.433693
CPD2032	6.713236	934.25595	CPD35297	20.8581667	363.27605	CPD54059	9.60820921	1252.06261
CPD20322	11.5458551	599.286433	CPD35299	21.0837262	668.284033	CPD54062	9.64350146	1246.06708
CPD20323	11.621808	586.280633	CPD3530	21.477129	324.30452	CPD54064	9.55811246	869.065456
CPD2033	6.6812841	405.15641	CPD35309	21.1077794	1006.52496	CPD54074	9.60012723	1227.06986
CPD20331	11.8358337	602.272267	CPD35313	21.1344998	511.38882	CPD54076	9.55696347	1261.81987
CPD20334	11.8796824	614.273154	CPD35317	21.2794058	383.30196	CPD54077	9.58739138	1010.45806
CPD20337	12.052997	434.14815	CPD3532	21.509954	392.27589	CPD54079	9.66166077	1238.31111
CPD20338	12.060166	258.116217	CPD35325	21.5431251	794.586462	CPD54080	9.70324866	1255.3178
CPD20339	12.2000008	464.15798	CPD35344	21.7336666	626.273373	CPD54083	9.707455	985.061623
CPD2034	6.6670962	426.15202	CPD35350	21.8301989	614.274212	CPD54084	9.63921839	1231.32501
CPD20340	12.235502	614.272325	CPD35351	21.4776556	714.29038	CPD54087	9.68455021	1237.56246
CPD20348	12.6924023	495.19936	CPD35352	21.7932071	477.376644	CPD54090	9.71339833	1223.8252
CPD2035	6.6393646	357.19861	CPD3537	21.570571	632.41009	CPD54094	9.71811952	1253.82181
CPD20350	12.6946226	434.146925	CPD35379	22.4086441	582.406089	CPD54095	9.7266695	1248.32387
CPD20351	12.6988342	493.19595	CPD3538	21.578342	551.85817	CPD54104	9.30002949	810.551925
CPD2036	6.7297973	772.20399	CPD35386	22.396913	886.610656	CPD54107	9.8359356	1226.8237
CPD20361	13.721696	598.26115	CPD35393	22.5569896	456.753389	CPD54110	9.82100386	1215.06491
CPD20366	14.2040187	159.0686	CPD35398	22.4736372	1011.67155	CPD54111	9.82071273	974.055014
CPD2037	6.7659943	491.14037	CPD3540	21.577666	610.45625	CPD54112	9.80354253	1241.80747
CPD2038	6.7739915	486.18368	CPD3541	21.563864	627.45509	CPD54113	9.78534152	988.463987
CPD20383	15.2832495	256.180526	CPD3543	21.58165	974.5559	CPD54117	9.82363356	973.854809
CPD2039	6.9068439	582.11886	CPD3545	21.58223	990.55057	CPD54118	9.82335601	1212.31311
CPD20393	15.8471983	470.28826	CPD35458	23.1379327	565.426191	CPD54122	9.29053327	988.854155
CPD20397	15.9993358	648.40055	CPD3546	21.587624	985.59551	CPD54123	9.88258338	1226.57216
CPD20398	16.0380008	129.057443	CPD35463	23.3553854	850.492725	CPD54124	9.90414568	1251.06481
CPD2040	6.8278953	574.12965	CPD3547	21.581586	1006.5241	CPD54126	9.94176527	981.258445
CPD2041	6.8067112	1110.3032	CPD35479	23.8030896	409.928278	CPD54128	9.92149826	951.659358
CPD20412	16.8205389	338.24275	CPD35486	23.8217075	409.975378	CPD54129	9.91414188	951.856069
CPD20413	16.9011509	316.20335	CPD3549	21.672846	600.41565	CPD54137	10.1064199	191.0436
CPD2042	6.9475379	610.15411	CPD3550	21.604385	582.7016	CPD54139	10.1726659	1212.56602
CPD2043	6.8167719	477.16183	CPD3551	21.675773	582.61604	CPD54140	10.1760002	1212.06477
CPD20430	18.1128167	317.23365	CPD35511	24.268377	383.33907	CPD54143	10.0533825	1250.81362
CPD2044	6.7564799	368.11003	CPD3552	21.845908	922.60209	CPD54147	10.4678189	418.219227
CPD20448	19.1867664	658.41262	CPD3553	21.648803	582.40684	CPD54186	16.3193336	409.281908
CPD20457	19.3456001	522.35314	CPD3555	21.658249	291.20573	CPD54190	17.1388859	1044.07364
CPD2047	6.8322661	978.26124	CPD35551	26.191144	547.433079	CPD54203	18.1810005	501.27406
CPD20484	20.5223557	640.28757	CPD3556	21.845323	1222.8142	CPD54219	19.9033998	422.266873
CPD2049	6.809114	218.09271	CPD35577	27.6592501	828.483575	CPD54221	19.9195577	449.277133
CPD20498	20.943414	692.274825	CPD3559	21.702395	564.39557	CPD54224	20.412949	598.402157
CPD2050	6.8366639	528.61788	CPD35600	28.6921448	521.381557	CPD54275	24.4482889	432.352964
CPD2051	6.8368314	516.09003	CPD3561	21.742202	282.19974	CPD54322	33.4114753	324.17048
CPD20529	21.4700548	819.462257	CPD3563	21.589971	244.21847	CPD54323	33.4828458	91.9993556
CPD2053	6.8145161	439.14134	CPD3564	21.645898	730.42903	CPD54413	1.0032998	466.9577
CPD2054	6.8006149	701.28635	CPD3565	21.643129	725.47083	CPD54442	1.07916604	386.208242
CPD20547	21.8053262	548.243712	CPD35651	33.1662491	396.747538	CPD54444	1.07493335	187.02516
CPD2057	6.8528978	394.06972	CPD35655	33.2296122	136.961647	CPD54465	1.13000062	565.2201
CPD2058	6.8378753	734.20188	CPD3566	21.652191	263.22802	CPD54473	1.14516684	504.244617
CPD2060	6.8237466	456.03729	CPD3567	21.661225	1037.7044	CPD54480	1.15245474	145.157664
CPD20604	23.075715	377.314912	CPD3568	21.67577	527.37295	CPD54481	1.15371437	390.265257
CPD20606	23.1600797	642.246146	CPD3569	21.669981	529.84487	CPD54482	1.15959987	207.09678
CPD2061	6.8862684	176.04683	CPD3570	21.667368	310.15715	CPD54490	1.17487498	476.226912
CPD2062	6.9243222	448.09951	CPD3571	21.677313	527.86824	CPD54501	1.32192306	382.142092
CPD20621	23.4291137	869.474011	CPD3572	21.780286	384.36918	CPD54502	1.61835986	333.097914
CPD20626	23.7869421	528.363908	CPD3573	21.570171	654.26909	CPD54516	1.56026667	476.228987
CPD2063	6.8386125	378.09215	CPD35732	1.05259999	395.0009	CPD54541	1.69587616	521.122937

CPD2064	6.8485037	194.0578	CPD3574	21.761381	993.67622	CPD54544	1.69683332	505.1296
CPD20647	24.6771894	954.58875	CPD3577	21.768099	507.83194	CPD54568	2.11128062	566.665014
CPD20648	24.7720379	630.448069	CPD35776	1.13163834	570.115682	CPD54571	1.97823218	258.230167
CPD2065	6.8684534	375.08883	CPD3578	21.768831	505.85565	CPD54575	2.06274858	573.658775
CPD2066	6.8934178	417.16248	CPD35795	1.06724104	422.067808	CPD54578	2.08126862	266.276114
CPD2067	6.8957306	331.16478	CPD3580	21.708843	733.45326	CPD54582	2.1099433	478.155331
CPD20672	25.4858474	334.251207	CPD35808	1.27214501	372.991571	CPD54587	2.04670931	463.165736
CPD20674	25.7228225	802.535606	CPD3581	21.7683	505.35525	CPD54595	2.15081306	573.155791
CPD20675	25.7874137	790.51033	CPD35811	1.26054481	165.078145	CPD54596	2.11698723	597.123886
CPD20690	26.7777492	682.962425	CPD35818	1.36254314	197.115211	CPD54598	2.13099884	733.130112
CPD2070	6.9471197	948.25194	CPD3582	21.785298	330.27557	CPD54602	2.15323549	526.669544
CPD20706	27.7198221	627.368824	CPD35829	1.15833954	421.051429	CPD54605	2.15244861	506.892533
CPD2071	6.9474333	224.13511	CPD3583	21.779062	742.42886	CPD54607	2.14882983	518.669327
CPD2073	6.9323427	764.21247	CPD35832	1.57770076	247.106443	CPD54609	2.17034175	124.052186
CPD2074	6.948993	501.08469	CPD3584	21.782584	539.40335	CPD54613	2.15714342	517.673457
CPD20755	33.1554009	130.013255	CPD35844	1.75500421	416.0896	CPD54614	2.17260165	521.45007
CPD20761	33.1324546	574.8105	CPD35845	1.68558401	482.106837	CPD54615	2.18006493	240.062073
CPD2077	6.9465973	531.99072	CPD3585	21.834503	903.61022	CPD54618	2.1690964	306.097927
CPD2079	6.9036069	236.06237	CPD35856	2.18642318	442.15346	CPD54622	2.22430229	586.21776
CPD20795	33.5394996	71.9300889	CPD3586	21.805182	560.34242	CPD54625	2.18588857	388.033222
CPD2081	6.890485	290.16567	CPD35868	5.43966605	317.147783	CPD54631	2.1442348	489.725867
CPD2082	6.9199516	398.1106	CPD3587	21.784673	544.35919	CPD54638	2.25154601	471.671227
CPD2083	6.9497753	516.01941	CPD3588	21.849344	325.37005	CPD54641	2.26670033	734.15106
CPD2084	6.9246375	159.06865	CPD35888	6.90503615	590.649852	CPD54643	2.30887751	518.997533
CPD2085	6.9391085	539.16964	CPD35897	7.50353852	248.052765	CPD54646	2.32989125	211.043537
CPD2086	6.9265592	382.11995	CPD3590	21.822783	970.58797	CPD54647	2.3399995	385.121817
CPD2087	6.9850626	338.09948	CPD35901	7.54316827	273.119436	CPD54648	2.34784647	487.051408
CPD20887	1.04025121	373.925517	CPD35903	7.60807866	421.195587	CPD54649	2.35280044	503.03408
CPD2089	6.9557168	403.21874	CPD35906	7.69775022	754.194525	CPD54658	2.22520805	349.254429
CPD2090	6.9494872	233.98432	CPD3591	21.824027	600.416	CPD54695	4.03975654	473.247188
CPD2091	7.0295925	408.17001	CPD35912	7.80362837	386.16111	CPD54696	4.40753104	326.138817
CPD20922	1.05380004	562.77974	CPD3592	21.825988	400.27827	CPD54702	4.62908121	476.085657
CPD2093	6.9255277	422.17143	CPD35927	8.14936875	528.178291	CPD54703	4.64707853	234.108677
CPD2094	6.9240475	292.10971	CPD35928	8.17528542	1160.192	CPD54704	4.6315881	571.159818
CPD2095	6.970507	902.14175	CPD3593	21.82054	600.63033	CPD54708	4.6665396	453.12785
CPD20967	1.06221053	206.006316	CPD35940	8.66459111	1292.33971	CPD54729	4.97879982	438.20995
CPD2097	6.9553336	447.17467	CPD3595	21.773982	402.27289	CPD54739	5.5027166	337.102407
CPD2098	6.9692699	918.11246	CPD35952	8.83645181	406.110418	CPD54741	5.5624222	378.109243
CPD2099	6.9501811	275.00891	CPD35953	8.85100048	341.1384	CPD54743	5.6058837	322.165278
CPD21001	1.07782341	175.003635	CPD35962	9.52266398	414.1537	CPD54755	5.62326327	361.04685
CPD21005	1.08105889	250.033312	CPD35963	9.05133291	655.204578	CPD54772	5.89518378	383.173218
CPD21011	1.12583342	632.968133	CPD3597	21.860619	949.65322	CPD54778	6.02450136	585.23023
CPD21019	1.1407034	201.078537	CPD3599	21.828018	582.61599	CPD54780	6.05460618	438.077625
CPD2102	6.9730013	405.0988	CPD3600	21.883667	923.09936	CPD54782	6.09823117	692.288285
CPD21026	1.54028086	162.05372	CPD36004	10.6601141	434.213038	CPD54789	6.14146004	307.006511
CPD21034	1.21955714	218.087529	CPD3601	21.862451	993.67696	CPD54791	6.15736861	624.296238
CPD21037	1.53904183	424.0688	CPD36014	11.6127992	419.21517	CPD54803	6.44099659	734.202188
CPD2104	6.9497129	386.12161	CPD36028	12.9264223	472.677562	CPD54807	6.53066413	375.1155
CPD21040	1.1126192	203.034462	CPD3603	21.86964	483.84287	CPD54811	6.62888681	692.288086
CPD21047	1.49644283	71.074188	CPD36037	13.3044311	502.688427	CPD54818	6.85739755	443.21653
CPD2105	6.9527151	224.19948	CPD3604	21.791065	582.40699	CPD54820	6.86011465	308.32712
CPD21051	1.45335824	109.001836	CPD36044	13.7078188	472.173167	CPD54837	6.92788201	460.187744
CPD21054	1.60008844	112.028422	CPD3605	21.80194	180.11657	CPD54843	6.91932852	355.120778
CPD21058	1.5644	535.15368	CPD36053	14.2904937	941.326057	CPD54844	6.95577078	1025.26148
CPD21059	1.56625003	173.049612	CPD36054	14.2282166	191.024562	CPD54850	6.95019656	1102.68708
CPD2106	6.9534528	224.06812	CPD36077	16.207444	463.861011	CPD54860	6.95071363	1102.88857
CPD21060	1.3660885	161.068294	CPD36079	16.397111	634.2623	CPD54862	6.95122623	590.165544
CPD21067	1.5632174	501.107248	CPD36080	16.5746521	525.29968	CPD54867	6.94769894	856.169677
CPD21068	1.5343752	313.086362	CPD36084	17.1278	405.28391	CPD54900	7.01248872	1020.65924
CPD21069	1.58507422	159.0968	CPD36087	17.3247701	475.152269	CPD54902	7.03096423	1021.05643
CPD2107	6.9543543	486.04591	CPD36090	17.3721114	636.481533	CPD54904	7.02570864	1020.85855
CPD21071	1.86791365	332.075856	CPD3610	21.798046	564.39515	CPD54907	7.02843813	851.212622
CPD21074	1.31503362	275.044831	CPD36102	17.6659733	598.401654	CPD54911	7.03368877	850.55031
CPD21075	1.57157159	321.079829	CPD3611	21.859319	291.20563	CPD54914	7.04700316	173.0862
CPD21079	1.56700078	85.9993444	CPD36112	18.202625	211.115275	CPD54931	7.07561071	848.54985
CPD2108	6.9352602	413.0819	CPD3612	21.861268	485.81875	CPD54940	7.12470083	1017.84952
CPD21080	1.34481558	352.146233	CPD36121	18.4319863	412.330489	CPD54945	7.08342738	798.181587

CPD21082	2.35190172	201.994025	CPD36125	18.9255536	1259.17831	CPD54978	7.22062562	1018.25031
CPD21087	1.64240107	260.136785	CPD36129	19.4522319	438.349488	CPD55007	7.12844662	1344.09431
CPD2109	6.9482136	206.05787	CPD3613	21.805733	637.43039	CPD55019	7.10033454	546.170929
CPD2110	6.9497209	241.04849	CPD36145	19.9755003	423.33505	CPD55021	7.13152417	1075.67616
CPD2111	6.9590757	408.10326	CPD3615	21.868589	349.31851	CPD55023	7.12322819	1343.59362
CPD21117	3.3303418	400.09296	CPD3616	21.879282	970.5834	CPD55025	7.12653251	1343.84199
CPD21121	3.58536371	400.0933	CPD36165	20.3822213	738.282489	CPD55026	7.12750308	1344.34347
CPD2113	6.9927184	424.07765	CPD3617	21.858711	954.60566	CPD55054	7.12943066	896.562657
CPD2114	6.9593029	794.22273	CPD3618	21.844824	354.2745	CPD5507	1.1060608	945.16439
CPD21149	5.77661197	352.09875	CPD3619	21.889825	500.33306	CPD55092	7.15649968	1256.54423
CPD2115	7.030832	194.10981	CPD36207	21.830305	393.327792	CPD55093	7.14799177	840.52825
CPD2116	7.1721787	174.13951	CPD36238	22.1587915	519.419937	CPD55102	7.15144009	451.110778
CPD21174	5.8513984	628.074	CPD3624	21.894675	495.37744	CPD55111	7.20581896	440.1044
CPD2118	6.990561	309.15823	CPD36250	22.5491109	906.072211	CPD55133	7.22851182	1061.86425
CPD21192	6.20436719	248.15482	CPD3627	21.893271	903.6093	CPD55136	7.24796567	885.053733
CPD21196	6.05500123	824.10835	CPD3629	21.919524	983.60061	CPD55140	7.23632694	884.885856
CPD2120	7.0040338	387.24596	CPD36293	22.9777789	411.334444	CPD55141	7.24607667	885.387242
CPD21205	6.10059937	294.114979	CPD36294	23.0741249	694.552775	CPD55142	7.2316708	1062.06244
CPD2121	6.8232304	146.03659	CPD3630	21.92616	249.07891	CPD55144	7.23500346	1062.46224
CPD2122	7.0416082	796.29595	CPD3631	21.939623	329.18636	CPD55155	7.25243066	1303.07146
CPD2123	7.0249613	200.15266	CPD36326	23.6485456	963.622509	CPD55180	7.31589756	416.13177
CPD21238	7.16400171	170.0476	CPD3634	21.959882	463.80647	CPD55236	7.46039891	1251.81033
CPD2124	7.0317506	244.1772	CPD3635	21.931103	361.20446	CPD55241	7.46221459	834.5406
CPD21243	6.93807485	320.142908	CPD36357	24.9283622	838.661337	CPD55244	7.44900893	834.041729
CPD21251	7.53939849	248.103275	CPD3636	21.960627	910.58207	CPD55246	7.45732713	1251.30924
CPD21255	7.39940885	398.127389	CPD3637	21.815364	282.19759	CPD55276	7.56400196	1036.05466
CPD21257	7.63967618	330.125912	CPD3639	21.935499	464.15307	CPD55284	7.63479443	842.709892
CPD2126	7.0795388	449.25952	CPD3640	21.963236	905.62696	CPD55295	7.62463764	1170.32288
CPD21267	7.70753317	226.12095	CPD3641	21.936907	793.35039	CPD55297	7.66338779	842.046067
CPD2127	6.9867627	764.21319	CPD3642	21.986	628.46037	CPD55302	7.64944008	797.3817
CPD2128	7.2294915	426.16277	CPD3643	21.955213	410.16562	CPD55306	7.679509	844.380436
CPD21280	7.86400433	522.150117	CPD3644	21.98415	650.44006	CPD55310	7.7264509	797.217871
CPD21286	7.42111005	396.154553	CPD3646	21.98702	288.26587	CPD55313	7.68566616	788.181
CPD2129	7.0852276	410.18996	CPD3649	22.000778	267.25651	CPD55318	7.73832495	999.847733
CPD2130	7.1736709	221.06573	CPD36496	33.1565	410.255475	CPD55319	7.68226631	797.715864
CPD21301	8.01452544	251.079662	CPD36498	33.1578177	409.755127	CPD55327	7.69775069	957.05834
CPD2131	7.0897718	766.2918	CPD3650	22.018399	967.58407	CPD55336	7.72639946	998.24855
CPD21314	8.07334844	230.043	CPD3652	22.001052	546.50662	CPD55337	8.11676018	887.083687
CPD2132	7.1222183	176.04697	CPD3653	22.256916	828.49868	CPD55338	7.74312184	624.285533
CPD2133	6.8690767	373.13654	CPD3654	21.972217	469.21674	CPD55346	8.03944348	856.7417
CPD21330	8.29792238	544.189181	CPD3655	22.01545	972.54132	CPD55353	7.84256634	641.813471
CPD21338	8.57763943	334.151767	CPD3656	22.007517	258.19712	CPD55362	7.83074079	641.937986
CPD2134	7.0535451	734.20126	CPD3657	22.009316	608.43779	CPD55365	7.86216471	864.409167
CPD21342	8.36529116	433.12226	CPD3658	21.998459	451.34967	CPD55388	7.96218076	845.048757
CPD21346	8.74816826	694.189068	CPD3662	22.060124	433.27967	CPD55390	7.91416448	868.421083
CPD21349	8.62680718	636.2787	CPD3664	22.05787	861.60024	CPD55458	8.13089427	1080.48124
CPD2135	7.0652725	194.05819	CPD3665	22.00247	279.25616	CPD55470	8.14211225	1295.78042
CPD21350	8.64177889	208.11182	CPD3666	22.051326	882.53384	CPD55479	8.17237059	856.579891
CPD2136	7.087778	394.0685	CPD3667	22.098191	742.46608	CPD55480	8.16650989	906.263514
CPD21363	9.0224161	309.066127	CPD3668	22.195631	795.5116	CPD55511	8.17950162	639.314088
CPD2137	7.0781781	212.03794	CPD3669	22.056308	653.42642	CPD55512	8.2135043	744.64787
CPD21370	9.06201962	204.039651	CPD36696	1.11542079	204.007769	CPD55518	8.19079216	1039.10515
CPD21376	9.28486967	380.156938	CPD3670	22.073727	624.25943	CPD55530	8.18238547	1046.95553
CPD21377	9.37785028	794.326767	CPD36707	1.57055572	572.081789	CPD55535	8.19399424	1094.92154
CPD2138	7.0609534	376.14022	CPD36725	1.38215008	108.020858	CPD55561	8.20742302	1283.3686
CPD2139	7.2784098	405.23468	CPD3673	21.990427	463.80747	CPD55568	8.18741154	1094.52331
CPD21391	9.4801947	145.05322	CPD3674	22.198687	800.46671	CPD55587	8.18820722	742.07868
CPD21395	9.6049992	348.11938	CPD3675	22.18283	816.44516	CPD55602	8.23031338	896.410977
CPD2140	7.0982258	208.14558	CPD36752	2.67680884	295.109125	CPD55610	8.20790972	1319.12105
CPD21400	10.0877886	239.07707	CPD3676	22.110479	351.31303	CPD55613	8.20545623	895.583025
CPD2141	7.2550551	226.16014	CPD36768	3.95499518	190.073367	CPD55673	8.19614702	1334.62173
CPD21416	9.87400036	415.12572	CPD3677	22.13865	373.29756	CPD55677	7.90250193	1070.90822
CPD21417	9.92139798	743.24724	CPD3678	22.235614	726.46856	CPD55685	8.24512707	1015.26036
CPD2142	7.0756664	378.09248	CPD36793	6.90773038	354.1002	CPD55689	8.41184626	1083.30093
CPD21425	9.92750054	602.77455	CPD3680	22.158608	784.47266	CPD55717	8.2702235	907.594244
CPD21428	10.0054757	527.177	CPD3681	22.114175	412.27966	CPD55723	8.31014156	922.429657
CPD21435	10.0483343	1937.60082	CPD36813	7.72817977	550.202927	CPD55750	8.34926156	863.088178

CPD21438	9.98390941	353.183667	CPD3682	22.104254	407.32472	CPD55751	8.41752371	821.886738
CPD2144	7.1233925	408.10328	CPD3683	22.155017	822.53218	CPD55772	8.38562464	884.258338
CPD21445	10.0816004	652.27158	CPD36838	8.6053746	786.203063	CPD55779	8.21492772	886.916356
CPD21446	10.0450726	792.317533	CPD3684	22.152636	744.48038	CPD55780	8.39059627	739.933178
CPD21449	10.1111515	344.1681	CPD36849	8.64431591	346.134446	CPD55782	8.3073655	733.92935
CPD2145	7.1266089	447.17466	CPD3686	22.152413	817.57384	CPD55784	8.37730262	885.08718
CPD21450	10.120531	577.289085	CPD36864	9.46862521	606.198163	CPD55797	8.43825785	1033.30218
CPD2146	7.1214808	403.14731	CPD36874	9.71137547	827.182213	CPD55834	8.51341579	1072.11806
CPD21465	10.5488358	248.052256	CPD3688	22.168747	327.25375	CPD55839	8.42589258	804.382131
CPD21467	10.3951661	318.120883	CPD36898	11.9754984	548.15531	CPD55842	8.44511945	981.079212
CPD21468	10.5443541	126.0311	CPD36900	12.1858743	260.07525	CPD55849	8.42133295	1084.11444
CPD2147	7.1206844	424.07745	CPD36909	13.996844	658.235489	CPD55854	8.429918	804.215185
CPD21472	10.395145	358.101314	CPD3692	22.187271	713.46746	CPD55857	8.44371064	981.281686
CPD21473	10.5732848	212.031875	CPD3693	22.17146	759.57422	CPD55862	8.404842	1026.29915
CPD21474	10.5464454	230.043348	CPD3694	22.1821	356.29022	CPD55868	8.36413446	1083.70547
CPD2148	7.1090491	388.21564	CPD36949	15.3970002	393.2272	CPD55871	8.43180107	1083.90852
CPD21482	10.3862281	708.24132	CPD3696	22.191617	330.27715	CPD55878	8.43899696	807.563475
CPD2149	7.1358525	224.06804	CPD36966	16.6119001	533.78994	CPD55884	8.37429702	1083.50685
CPD21490	10.7471639	708.260667	CPD3697	22.191938	312.26615	CPD55890	8.43183571	972.873942
CPD21491	10.7438333	616.2852	CPD3698	22.171698	238.2274	CPD55900	8.44040274	987.67033
CPD21494	10.7657926	244.09794	CPD36988	17.6119849	624.25601	CPD55902	8.4330051	733.07314
CPD21496	10.8450005	186.044757	CPD3700	22.2012	420.2486	CPD55910	8.45800215	987.069214
CPD2150	7.1515882	463.14482	CPD37003	18.2837265	1262.92697	CPD55926	8.56311742	1088.06767
CPD21500	10.9215	342.096867	CPD3702	22.321367	938.59265	CPD55938	8.6850366	1087.48105
CPD21503	10.9327996	508.20754	CPD3705	22.167558	844.49474	CPD55951	8.60958015	741.937371
CPD21512	11.2600013	690.25266	CPD37052	20.3901104	379.844678	CPD55961	8.54230074	870.761337
CPD21513	11.2575503	415.238127	CPD3706	22.210638	410.19792	CPD55962	8.52924092	1044.90943
CPD21523	11.6223331	690.252833	CPD37062	20.6377093	858.4592	CPD55967	8.53637097	1044.71199
CPD21527	11.7235543	413.224078	CPD3707	22.340571	933.63722	CPD55974	8.65951258	1200.31593
CPD2153	7.2002089	460.14842	CPD3709	22.247183	368.25194	CPD55976	8.55575219	1119.29684
CPD2154	7.2014264	206.13489	CPD3710	22.210603	798.45366	CPD55984	8.55406275	933.082236
CPD21548	11.8674012	210.12424	CPD3711	22.072442	866.55617	CPD55988	8.55830186	1128.33054
CPD2156	7.2020812	408.16758	CPD37115	21.8496335	739.289563	CPD55989	8.56138438	1128.12715
CPD21584	12.8834422	647.240533	CPD3713	22.258038	363.29894	CPD55998	8.53287487	1127.932
CPD2159	7.201057	369.16162	CPD37130	22.1433751	381.737713	CPD56018	8.60515093	889.914625
CPD21593	13.3973427	676.237313	CPD3714	22.249278	773.55004	CPD56029	8.56591514	589.163255
CPD2160	7.18099	372.12007	CPD3715	22.25037	778.51146	CPD56035	8.55453845	1087.6736
CPD21600	13.826545	553.241776	CPD3716	22.286305	424.28092	CPD56051	8.58987551	427.081262
CPD21615	14.8988734	570.238238	CPD37165	22.5432999	881.96256	CPD56055	8.61533633	935.106644
CPD21619	14.9051458	1394.52178	CPD3717	22.136184	828.49892	CPD56065	8.60111977	631.047525
CPD2162	7.1908298	332.14413	CPD37186	23.0387231	536.524509	CPD56069	8.59261798	258.040669
CPD21621	14.9696739	810.235506	CPD3719	22.366437	324.22858	CPD56073	8.59151254	1087.86989
CPD21622	14.9609562	742.246046	CPD37197	23.0327146	905.650071	CPD56075	8.64337503	497.2504
CPD21628	14.9789995	675.589049	CPD3720	22.261257	346.27346	CPD56084	8.64069605	1359.83492
CPD21635	15.055174	614.395617	CPD3721	22.320067	686.29423	CPD56089	8.74760058	1075.07722
CPD21639	15.198791	292.1206	CPD3723	22.264178	823.54015	CPD56090	8.7099978	1074.67295
CPD2164	7.1931307	327.18967	CPD37232	23.7210767	716.6349	CPD56091	8.71156514	879.904457
CPD2165	7.2950483	182.08651	CPD3724	22.225586	816.45003	CPD56098	8.71333368	1343.58958
CPD21665	16.2501458	614.395493	CPD3725	22.417788	784.47237	CPD56107	8.69099748	396.10166
CPD21666	16.2725022	595.251113	CPD37256	23.8802871	638.274123	CPD56109	8.81067014	977.05048
CPD21677	16.6713564	525.30178	CPD3729	22.345003	638.39365	CPD56125	8.71315303	1062.87581
CPD21678	16.6362456	536.29588	CPD37297	27.7133154	474.196262	CPD56127	8.74049846	1367.59993
CPD2168	7.4535227	338.09985	CPD3730	22.344207	734.47877	CPD56129	8.74100252	1367.85018
CPD21685	16.940083	316.2047	CPD3732	22.238083	800.46657	CPD56132	9.06111031	980.655775
CPD2169	7.3366041	146.03652	CPD3733	22.452657	598.61436	CPD56135	8.74859853	1367.09951
CPD217	1.012053	316.958	CPD3734	22.226383	795.51114	CPD56144	9.03229977	984.46407
CPD2170	7.2880845	415.18739	CPD3735	22.340928	729.52309	CPD56150	8.78022165	1040.47421
CPD21700	17.5314987	444.980233	CPD37369	33.1637741	252.228379	CPD56151	8.73889068	1315.09901
CPD2171	7.3053633	704.22824	CPD3738	22.543474	782.45801	CPD56156	8.82117324	816.53932
CPD21712	17.7940322	739.284789	CPD3740	22.376635	267.25579	CPD56161	8.82910035	976.25197
CPD2172	7.3950951	340.13028	CPD3741	22.301069	368.2537	CPD56165	8.8210735	818.868187
CPD21723	18.0070834	372.24523	CPD3742	22.257237	306.27667	CPD56171	8.82641649	1220.81527
CPD21726	17.9643539	852.447731	CPD3743	22.343775	654.37158	CPD56183	8.87356054	1087.89749
CPD2173	7.2946765	360.09029	CPD3744	22.423964	961.65091	CPD56184	8.88212902	1088.09834
CPD21733	18.0403838	700.309225	CPD3745	22.404093	616.41232	CPD56190	8.85392773	1636.24705
CPD21736	18.1481792	702.290282	CPD3747	22.406496	564.39532	CPD56195	8.88037352	1087.6955
CPD21739	18.225636	290.244982	CPD37475	1.25649423	318.045909	CPD56210	8.90164764	835.1981

CPD2174	7.31457	264.13513	CPD3748	22.378563	622.40377	CPD56211	8.8438776	832.703838
CPD21746	18.3823417	780.304171	CPD3749	22.421694	966.60662	CPD56218	8.91896807	1253.04565
CPD2175	7.3050941	299.17479	CPD3750	22.417103	598.4052	CPD56219	9.03801723	1002.43716
CPD21750	18.4109466	375.1741	CPD3751	22.407165	602.89169	CPD56230	8.93389624	1244.31524
CPD21759	18.602598	652.253312	CPD3752	22.410769	600.91417	CPD56231	8.8998981	1253.29466
CPD2177	7.3165158	699.27112	CPD3753	22.451291	690.45332	CPD56243	9.02523783	1009.44815
CPD21770	18.8088004	505.1384	CPD37530	3.03072459	157.07257	CPD56244	9.04420383	1065.48204
CPD21774	18.6606663	624.257711	CPD3754	22.438881	685.49721	CPD56251	9.03042864	980.0667
CPD2178	7.3222957	425.16525	CPD37571	6.07194552	343.067125	CPD56259	8.99240163	817.384767
CPD2180	7.3304768	431.2719	CPD3758	22.552598	641.47165	CPD56266	9.03157735	1194.06384
CPD21806	19.449617	846.508072	CPD37580	6.49370752	282.073971	CPD56277	9.34862876	1247.80477
CPD21810	19.4694	371.14246	CPD3759	22.37559	744.4817	CPD56282	9.07244877	984.662827
CPD21811	19.37975	279.255707	CPD3760	22.689117	536.8525	CPD56283	9.16830699	1219.06666
CPD21821	19.7470618	660.25544	CPD37601	6.52492213	403.14607	CPD56300	9.07725038	984.862908
CPD21829	19.8268178	542.18594	CPD3762	22.51631	578.40078	CPD56302	9.09739664	1301.83177
CPD2183	7.3757401	161.04812	CPD37624	7.74638403	368.079982	CPD56303	9.2064369	965.048729
CPD21842	20.5113671	1001.56927	CPD3763	22.557571	580.88076	CPD56309	9.0113914	1218.05319
CPD21845	20.5029316	672.278238	CPD3764	22.679279	782.45826	CPD56317	9.16249288	1222.54389
CPD2185	7.3900078	419.10968	CPD37640	8.26743761	353.333456	CPD56332	9.16282945	1044.69006
CPD21850	20.6933026	686.294026	CPD37647	8.61946839	985.249682	CPD56334	9.15222231	1624.41616
CPD21854	20.7247491	702.288855	CPD3765	22.528212	646.42747	CPD56338	9.18033848	1064.683
CPD21855	20.6577201	836.483957	CPD37650	8.58546416	467.240629	CPD56346	9.14741789	1064.08105
CPD21856	20.7070035	670.26544	CPD3766	22.624984	293.26834	CPD56358	9.18333266	1330.10109
CPD2186	7.3919879	408.19899	CPD3767	22.507034	641.69108	CPD56366	9.18065804	1047.68411
CPD21868	21.0760241	668.284818	CPD3768	22.63118	580.41118	CPD56383	9.3882472	975.254475
CPD21885	21.4359444	520.49515	CPD3769	22.453658	798.44523	CPD56389	9.28137262	995.046829
CPD2189	7.4490525	824.25717	CPD37693	11.5847085	979.327036	CPD56393	9.23967012	975.65437
CPD21890	21.5678093	772.511963	CPD3770	22.453699	968.57837	CPD56399	9.23632803	1362.3498
CPD21893	21.5772173	712.454977	CPD37702	11.7617447	235.169863	CPD56401	9.2407488	846.222167
CPD21895	21.6438737	584.421007	CPD37706	12.0908968	459.157029	CPD56403	9.21191022	990.23061
CPD21898	21.7501989	356.138988	CPD3771	22.592911	622.27936	CPD56406	9.14044904	1241.54147
CPD21899	21.7225864	716.304992	CPD37711	12.247813	417.79385	CPD56429	9.28261311	1303.34306
CPD2190	7.4788748	376.04475	CPD3772	22.589182	641.47224	CPD56433	9.13982592	1006.05615
CPD21901	21.9517648	636.25834	CPD37721	13.066671	510.686342	CPD56438	9.31118441	1220.55633
CPD21906	21.8328475	614.27457	CPD37724	13.1225971	487.17933	CPD56454	9.09617486	1231.06011
CPD2191	7.4180466	230.04234	CPD37728	13.2585993	467.18092	CPD56455	9.33211078	976.839709
CPD21910	22.0061005	258.19771	CPD3773	22.61491	558.86558	CPD56458	9.31029505	1211.5535
CPD21911	21.9734488	434.801127	CPD37731	13.7446685	537.201578	CPD56461	9.3448591	1168.53479
CPD21915	21.9448804	738.287341	CPD3774	22.619238	556.39247	CPD56468	9.3780037	1568.42607
CPD21924	22.1157567	648.254481	CPD3775	22.62077	602.40183	CPD56475	9.39225151	1406.37247
CPD21927	22.2335843	811.318963	CPD3776	22.655727	255.25611	CPD565	1.007305	522.9108
CPD2193	7.4638889	464.69654	CPD3777	22.622629	556.88885	CPD56502	9.40682038	1206.55541
CPD21932	22.3623144	658.240794	CPD3778	22.852748	963.66758	CPD56513	9.43278761	1283.56709
CPD21934	22.3906102	676.281239	CPD3780	22.64001	597.44549	CPD56525	9.48545699	1017.44311
CPD21936	22.408667	654.313737	CPD3782	22.659575	376.22241	CPD56530	9.4507339	1006.45486
CPD2194	7.4244814	339.15268	CPD3783	22.657772	558.12269	CPD56532	9.42783775	998.444753
CPD21940	22.3990494	670.26775	CPD3784	22.652195	370.0956	CPD56534	9.44868944	1014.44785
CPD2195	7.424544	344.10872	CPD3786	22.666332	553.1684	CPD56544	9.57328203	997.645555
CPD21951	22.8719002	706.260467	CPD37862	18.854833	540.328283	CPD56547	9.27011009	1250.05669
CPD21954	23.0481859	666.268486	CPD3787	22.792497	536.85185	CPD56556	9.53712534	1074.69589
CPD21956	23.1040695	1320.46555	CPD3789	22.693925	534.87568	CPD56565	9.54660316	1066.28856
CPD21957	23.0203102	831.589733	CPD3790	22.644997	1051.7146	CPD56571	9.54934834	1333.10633
CPD2196	7.4277625	180.0367	CPD37917	20.1431426	379.250657	CPD56572	9.5690823	1209.05061
CPD2197	7.4878151	507.17449	CPD37918	20.1362963	706.367514	CPD56575	9.23624754	1261.53395
CPD21974	23.3260457	706.260345	CPD3792	22.817451	590.2518	CPD56595	9.56170117	1062.47023
CPD2198	7.5064094	537.18326	CPD3793	22.691629	537.36259	CPD56601	9.56642392	1211.05952
CPD21981	23.4670299	772.512751	CPD3794	22.706445	190.13732	CPD56609	9.69621166	990.65275
CPD21983	23.6251477	620.236095	CPD37943	20.4485444	385.298671	CPD56619	9.68429064	989.848255
CPD21984	23.6447188	598.24286	CPD3796	22.715056	385.29598	CPD56635	9.73212434	979.867129
CPD21985	23.609523	656.284669	CPD3797	22.934708	919.64225	CPD56640	9.78983349	962.647525
CPD21989	23.7094539	548.242909	CPD3798	22.713219	588.23708	CPD56653	9.72923269	1255.81532
CPD21993	23.9406482	889.636383	CPD37980	21.3977284	684.277888	CPD56654	9.82272465	1251.31215
CPD21997	23.9919246	628.289335	CPD3799	22.719883	558.37484	CPD56688	10.2687581	1226.07184
CPD21999	23.9655711	842.514125	CPD3800	22.698687	782.458	CPD56690	10.4693347	400.210011
CPD2200	7.5154757	479.23537	CPD38012	21.7622	710.84588	CPD56702	12.4956674	820.063671
CPD22002	24.2730285	950.559461	CPD38018	21.7816923	761.237438	CPD56712	14.1369828	304.23302
CPD22005	24.2967615	628.288376	CPD3802	22.723033	553.41907	CPD56714	14.5250004	545.311341

CPD22006	24.3175968	679.246392	CPD38022	21.8544759	354.274636	CPD56716	14.9658981	464.44375
CPD2201	7.5844075	346.10857	CPD38029	22.0101427	786.8872	CPD56738	19.0074627	1258.92687
CPD22015	24.6999401	283.289911	CPD3803	22.748812	826.48435	CPD57009	1.14133481	580.140967
CPD22017	24.8859119	604.273504	CPD38031	22.0848665	988.5233	CPD5705	1.1030351	252.1165
CPD2202	7.4887189	787.24358	CPD3804	22.705295	580.4114	CPD57054	1.58659991	665.06463
CPD22020	25.0713635	1214.31446	CPD38042	22.2057274	708.253028	CPD57105	2.10306639	142.045671
CPD22027	25.1267509	608.8075	CPD3805	22.779818	514.84022	CPD5714	1.024511	212.96674
CPD2203	7.5407882	328.09555	CPD3806	22.768186	974.55605	CPD5720	1.0170646	547.82131
CPD2205	7.5413152	435.21266	CPD3808	22.780092	517.81986	CPD57219	6.87400739	433.123517
CPD22051	25.8473188	636.296395	CPD38080	22.6083392	866.042633	CPD5722	1.0131051	379.89841
CPD22053	25.8970803	652.290596	CPD3809	22.987346	875.6164	CPD57232	7.06138955	798.18145
CPD2206	7.590112	908.25381	CPD3810	22.780922	512.86233	CPD57238	7.08818316	790.195387
CPD22069	26.3871424	636.296588	CPD3811	22.848609	964.66603	CPD57242	7.14410317	1024.08311
CPD2207	7.580781	462.11241	CPD3813	22.811965	514.34874	CPD57248	7.14873009	1257.29736
CPD22077	27.6256809	648.437684	CPD3814	22.82625	686.29396	CPD57249	7.17111332	1096.28662
CPD22079	27.7104895	702.484714	CPD38154	23.6424273	828.355679	CPD57264	7.26448271	868.71626
CPD2208	7.5895408	903.29788	CPD3816	22.860189	592.48824	CPD57279	7.28581217	840.199357
CPD22080	27.7282382	534.389544	CPD3817	22.812836	272.24849	CPD57287	7.6323685	751.186527
CPD22082	27.7313433	383.186392	CPD3818	22.819349	509.39321	CPD57288	7.60370807	1016.23532
CPD22083	27.7251794	68.0630692	CPD3819	22.834067	592.26872	CPD57296	7.7054969	1345.30812
CPD22085	27.7278885	70.07861	CPD3820	22.797563	969.60864	CPD5731	1.0312498	335.83892
CPD22087	27.7204184	574.603257	CPD3823	23.065981	896.55004	CPD57335	8.01888918	1013.8459
CPD22088	27.7286017	56.063085	CPD38239	27.7097458	222.233538	CPD57344	8.23124373	843.889556
CPD2209	7.5643908	434.21023	CPD3826	22.842341	492.82663	CPD57349	8.2572086	843.054037
CPD22096	28.5334381	652.272262	CPD3828	22.856602	490.85037	CPD5735	1.0282382	387.84815
CPD2210	7.5888934	662.20453	CPD38292	33.2414988	122.046013	CPD57350	8.25230552	865.421725
CPD2211	7.5919501	555.09811	CPD3831	22.933181	510.70953	CPD57360	8.22768279	1012.26304
CPD22110	29.3829002	468.377668	CPD3833	22.913028	465.36777	CPD57378	8.35062175	1036.10318
CPD22112	29.3861678	745.557604	CPD38338	1.02944881	294.951944	CPD57379	8.27333295	858.081713
CPD22118	29.9215156	700.402929	CPD3834	22.921597	470.32329	CPD5738	1.0227062	276.94262
CPD2212	7.5904232	500.15266	CPD3835	22.904367	255.25644	CPD57387	8.42491971	856.08403
CPD22124	30.6620102	429.3709	CPD38367	1.09683334	530.130308	CPD57388	8.41624935	811.062662
CPD22129	30.6301952	708.497136	CPD3837	22.881188	626.27488	CPD5741	1.0339166	339.85009
CPD22137	31.6670024	588.471367	CPD38382	1.10041405	527.107471	CPD57418	8.55936294	1128.52885
CPD2214	7.6256926	692.21521	CPD38384	1.14211065	377.220178	CPD5742	1.0269914	357.02997
CPD22141	32.6304367	484.327552	CPD3839	22.976689	485.83117	CPD57421	8.53989794	1035.68006
CPD22151	33.1216011	285.92011	CPD3840	22.941068	470.81349	CPD5744	1.024353	303.88588
CPD22164	33.1231428	150.136157	CPD38400	1.01360504	71.0739391	CPD57442	8.94975786	1002.24269
CPD22169	33.1636421	244.910044	CPD38404	1.50271331	271.106414	CPD57447	8.76539767	1300.09546
CPD2217	7.633168	475.29798	CPD38405	1.62380917	409.15588	CPD57494	8.99733456	1225.8145
CPD2218	7.6493155	371.21439	CPD3841	22.953116	345.22565	CPD57502	8.87165307	818.704743
CPD2219	7.6783744	302.12566	CPD38417	1.58044101	151.049314	CPD5751	1.0374166	639.73364
CPD2221	7.733382	487.16993	CPD38418	1.58987529	170.02816	CPD5753	1.0317806	407.9137
CPD2222	7.7118779	488.29261	CPD3842	22.960234	520.84232	CPD57545	9.12610813	864.064
CPD2224	7.72084	368.1109	CPD38434	2.17020461	175.00672	CPD5755	1.1132864	325.07598
CPD2225	7.7462281	396.09026	CPD38438	2.28711766	540.149095	CPD57556	9.11120342	1056.67324
CPD2226	7.9117932	776.21	CPD38455	5.44027961	147.071312	CPD57564	9.17105418	1200.3145
CPD2227	7.7617613	530.16331	CPD3847	22.972511	1024.6497	CPD57566	9.16872726	1330.60276
CPD2228	7.7492555	404.07586	CPD38473	6.93745937	366.151086	CPD57569	9.1771447	1216.54681
CPD2229	7.7670056	771.25694	CPD38476	6.66883213	208.110267	CPD57573	9.30210106	1219.56517
CPD2230	7.755497	187.11989	CPD38481	6.83863648	187.033555	CPD57578	9.18057855	1231.3124
CPD2231	7.7546735	169.10958	CPD38482	6.87388871	760.202478	CPD5758	1.0529441	458.86766
CPD2232	7.8205392	561.18407	CPD38483	6.84116693	140.083525	CPD5760	1.0409998	589.82572
CPD2234	7.859937	516.01859	CPD38489	6.99789877	330.13083	CPD57618	9.50443952	959.2481
CPD22341	1.09958379	449.90688	CPD38491	7.03352214	560.184616	CPD5762	1.0370001	441.79354
CPD2235	7.8535848	236.06234	CPD38495	7.18516644	236.110617	CPD57620	9.22096591	1226.06729
CPD2236	7.9449748	398.11689	CPD38504	7.35208955	601.237545	CPD57624	9.34997082	1016.86075
CPD22360	1.12620021	486.03985	CPD38511	7.57736318	370.158363	CPD57627	9.24242995	975.853378
CPD2237	7.8589365	159.06838	CPD38515	7.75560061	599.25742	CPD5764	1.0428995	305.94356
CPD22386	1.15533323	518.163256	CPD3852	22.987468	553.43182	CPD57641	9.40545966	995.452925
CPD2239	7.8946469	746.20038	CPD38527	8.07634077	338.10065	CPD57661	9.54977532	970.035062
CPD22390	1.28605955	325.1004	CPD38537	8.25099825	612.102454	CPD57678	9.54399936	1208.55051
CPD22393	1.27038568	332.070379	CPD3854	23.082583	377.31507	CPD57680	9.51116061	963.84468
CPD22400	1.29882282	434.026864	CPD38544	8.80703681	924.236533	CPD57682	9.55542919	1302.84536
CPD22408	1.38134631	343.990193	CPD3855	23.02074	426.29662	CPD57685	9.55114287	1303.09109
CPD2241	8.026843	699.249	CPD38554	8.04396066	423.17414	CPD57716	9.73582846	1203.05877
CPD22411	1.38261399	460.003195	CPD38555	8.51133998	321.0854	CPD57718	9.88259049	980.859617

CPD2242	7.9083099	346.15912	CPD3856	23.003969	875.61571	CPD57728	9.77673817	988.06387
CPD22423	1.49426731	89.0477526	CPD3857	22.990287	421.34081	CPD5773	1.0367696	243.85387
CPD22428	1.33097429	218.091325	CPD38572	8.65230329	84.020996	CPD57752	10.2532426	1226.82016
CPD22429	1.5642499	176.046775	CPD38574	8.90242541	733.861857	CPD57833	15.0228276	923.51162
CPD2244	7.8870529	519.32372	CPD38575	8.88072557	365.171527	CPD57914	26.3466957	724.520286
CPD22445	1.60008351	375.155669	CPD38583	9.05549379	924.232515	CPD5806	1.0489655	211.84016
CPD22447	2.00433238	313.173876	CPD38584	9.15575015	201.030142	CPD58069	1.31055541	630.1214
CPD2245	7.7145894	533.17953	CPD38585	9.15169249	294.111092	CPD58074	1.34570175	504.048138
CPD2246	7.9741883	901.28236	CPD3859	23.20892	743.53849	CPD58120	2.06456591	354.104044
CPD2247	8.0374523	906.24401	CPD38597	9.6655087	126.031686	CPD58126	2.04437097	611.127475
CPD22479	5.51080114	217.131467	CPD3860	23.037551	880.57165	CPD58129	2.10279502	655.197471
CPD2248	7.9721011	722.20418	CPD38604	9.19886862	802.706543	CPD58135	2.11511733	304.0946
CPD2249	7.9734585	1494.409	CPD38611	9.88499396	526.23724	CPD5814	1.0502223	666.74423
CPD22493	6.33865873	222.108871	CPD38614	10.119968	411.811512	CPD58147	2.14425017	548.128933
CPD2250	7.9712379	766.68165	CPD38616	10.1972504	844.2927	CPD58174	2.23975036	571.714187
CPD2251	7.9761142	774.66872	CPD3862	23.279843	748.49427	CPD58178	2.23999938	586.721071
CPD22517	7.83333611	373.135236	CPD38620	10.2527797	625.714944	CPD58193	2.26685672	274.064379
CPD2252	8.0120217	368.11052	CPD38621	10.291907	518.2353	CPD58197	2.21233891	479.153243
CPD22521	8.00191254	781.686387	CPD38625	10.3711661	411.8111	CPD5821	1.0519375	394.86601
CPD22522	8.05823775	206.135979	CPD38648	10.7511602	828.295134	CPD58233	2.48956606	574.235811
CPD2253	7.970091	766.17971	CPD3865	23.093704	536.52734	CPD58237	2.46914286	544.225343
CPD2254	7.7338901	218.09298	CPD38655	11.311167	979.32585	CPD5826	1.0505	478.82737
CPD22540	9.06984906	958.258573	CPD38656	11.3732487	507.264113	CPD5828	1.0554628	358.9011
CPD22541	9.14134188	330.132145	CPD38659	11.546223	491.230856	CPD58322	4.64200027	612.142311
CPD2256	7.9730024	773.66871	CPD3866	23.095862	536.72926	CPD58332	4.63838412	547.216488
CPD22560	9.77042736	776.213986	CPD38660	11.5786926	473.839562	CPD58333	4.63854423	279.297278
CPD22563	9.94699931	602.4851	CPD38670	12.2100277	453.22135	CPD58336	4.64232962	541.716031
CPD2257	7.9833984	192.07819	CPD38672	12.2222867	411.141114	CPD58338	4.66492273	639.160958
CPD2258	7.95078	610.15369	CPD3869	23.107535	710.23119	CPD58340	4.6603097	348.065446
CPD22584	12.2480233	274.131876	CPD3870	23.127783	700.20489	CPD5837	1.0440001	381.76154
CPD2259	7.9646699	180.1147	CPD38701	13.3484293	957.321186	CPD5841	1.0379394	376.91962
CPD22591	13.1079137	471.671092	CPD38707	13.6315718	503.191043	CPD58416	5.18385576	440.20001
CPD2261	7.9780106	403.14717	CPD38709	13.6360901	486.6759	CPD58421	5.31510454	294.09873
CPD22614	15.2002554	1048.07251	CPD3871	23.082801	324.26284	CPD58426	5.46578868	333.106243
CPD2262	7.9454721	286.04761	CPD3872	23.113567	778.21915	CPD5843	1.0515715	660.80876
CPD22638	17.8198511	351.2215	CPD3873	23.126567	620.26366	CPD58453	6.72012573	486.103175
CPD2264	7.9839169	408.10314	CPD38732	14.347501	957.324017	CPD58495	6.89706147	306.831762
CPD22640	17.8718541	154.100253	CPD38735	14.4075257	1005.37496	CPD58517	7.3156595	493.178978
CPD22646	18.1923073	724.271607	CPD3874	23.103337	831.58956	CPD5856	1.0526662	592.79155
CPD2265	8.0209584	212.03477	CPD38741	14.5073649	502.6886	CPD58572	8.19132915	859.57615
CPD22656	18.5562704	702.290033	CPD38742	14.4742793	503.190222	CPD58577	8.25144409	1012.66313
CPD22663	19.1517126	630.302064	CPD3875	23.201091	693.52092	CPD58600	8.25320886	889.75492
CPD2267	8.0048149	422.11846	CPD3876	23.135034	923.51564	CPD5863	1.0759172	412.87173
CPD22671	19.2792323	394.2572	CPD38768	15.0711815	706.217827	CPD5864	1.0416212	459.87182
CPD22672	19.3821879	383.248707	CPD3877	23.148053	564.37831	CPD58668	8.74344922	1366.8514
CPD22673	19.6983197	363.252437	CPD38775	15.2993029	705.280333	CPD5867	1.0559404	193.02469
CPD2268	8.0210664	577.21133	CPD3878	23.128943	514.36969	CPD5868	1.0533	348.8815
CPD22688	20.3346615	654.369794	CPD38781	15.3627698	618.2365	CPD58689	8.81565352	1299.84394
CPD2269	7.9460996	772.20069	CPD38789	15.794508	353.2357	CPD5869	1.0580351	215.03954
CPD22695	20.6002036	753.47273	CPD38792	15.9022859	463.168714	CPD58701	8.82581243	1227.80109
CPD2270	8.0700568	393.10599	CPD3880	23.131228	852.52418	CPD58704	8.8376028	1041.47869
CPD2272	8.0720733	743.18709	CPD3881	23.143677	564.85072	CPD58736	9.05999863	548.1553
CPD22722	21.569807	608.438936	CPD38814	16.5926001	463.86249	CPD58745	9.09590952	1302.33072
CPD2273	8.0244928	415.24044	CPD38819	16.6518525	720.830857	CPD5876	1.0689109	347.87794
CPD2274	8.1152964	876.23497	CPD3882	23.16557	282.15536	CPD58778	9.3520979	578.163136
CPD22743	22.4643458	670.294312	CPD38822	16.7784999	388.317812	CPD5878	1.0705427	175.88467
CPD2275	8.0746861	759.6652	CPD3883	23.234832	281.94897	CPD58781	9.40734069	1215.31528
CPD22756	22.1114014	670.269775	CPD38847	17.3168549	483.1398	CPD58796	9.38996568	1214.56344
CPD22762	22.0191064	706.251773	CPD3885	23.150577	282.11031	CPD5880	1.0476192	369.94479
CPD22776	22.8762723	684.277595	CPD38850	17.4144951	738.282367	CPD5881	1.025901	264.10154
CPD2278	8.0844014	692.19362	CPD3886	23.136624	563.32937	CPD58812	9.55009874	548.15272
CPD2279	8.0124904	751.67623	CPD38861	17.9052496	638.459388	CPD58834	9.51670553	995.452429
CPD2280	8.0689836	1464.3982	CPD38866	18.1278	337.1118	CPD5885	1.0649994	212.06343
CPD22806	23.5705999	616.416679	CPD3887	23.139351	564.75478	CPD58851	11.4369992	205.061488
CPD2281	8.0281827	751.17488	CPD38886	18.6236054	739.284714	CPD58852	11.4612852	207.057514
CPD22812	23.8329902	988.572457	CPD3889	23.166663	326.32895	CPD58857	12.7786675	219.074822
CPD2282	8.0590859	759.16477	CPD38894	19.0065001	405.3234	CPD5887	1.0599549	210.96625

CPD22826	25.0838666	1319.52404	CPD3890	23.147377	563.11012	CPD5888	1.0500041	365.97705
CPD2283	8.0623854	224.14074	CPD38900	19.2904826	642.235231	CPD58886	20.4559981	538.35815
CPD22831	25.1121327	1264.52117	CPD3891	23.113129	836.54517	CPD5889	1.0771111	415.92861
CPD22835	25.1076234	628.266289	CPD38918	19.3241901	716.304167	CPD58903	21.9931427	507.318579
CPD22838	25.1237048	609.508508	CPD3892	23.147681	303.83084	CPD58905	22.3025994	533.3342
CPD22842	25.1708772	604.275216	CPD38925	19.3973572	630.272122	CPD5894	1.0859301	428.84635
CPD2285	8.1565828	618.2156	CPD38933	19.5545715	1031.48791	CPD5895	1.08125	323.91895
CPD22858	25.8739966	762.584512	CPD38934	19.5587554	344.121	CPD5897	1.0771932	135.93219
CPD2287	8.1414407	453.09258	CPD38935	19.6304204	1491.77608	CPD5902	1.1002133	302.99328
CPD2288	8.1054287	513.38791	CPD38936	19.574	1344.45279	CPD5903	1.1064287	565.08109
CPD22883	27.7160473	124.12353	CPD3894	23.147433	303.25952	CPD5905	1.1128203	368.88912
CPD22885	27.7216981	94.07793	CPD38943	19.6560832	1326.51042	CPD5907	1.0974827	511.13997
CPD2289	8.1136999	452.52196	CPD38948	19.7063	1294.48722	CPD5908	1.1178543	217.01757
CPD2290	8.113489	452.33608	CPD38957	19.702709	1465.76486	CPD5910	1.1007273	381.24931
CPD22900	28.9311024	613.784256	CPD38962	19.9241994	612.260025	CPD5912	1.1427645	189.1051
CPD2292	8.1163036	226.16733	CPD38967	20.1383337	720.244722	CPD5913	1.1041572	174.10804
CPD2293	8.1246325	563.34997	CPD38969	20.0213593	348.2644	CPD5915	1.108002	535.88622
CPD22949	33.0509998	125.95611	CPD3900	23.133841	584.82731	CPD5917	1.1020965	202.13892
CPD2295	8.1558548	311.15786	CPD3901	23.151415	264.2452	CPD5919	1.117407	251.96645
CPD22954	33.0975001	217.157444	CPD3902	23.152167	584.73415	CPD5920	1.1747715	552.05279
CPD22956	33.1264667	559.807973	CPD3903	23.142266	568.54673	CPD59234	2.17524923	402.11655
CPD2296	8.1606281	305.09014	CPD3904	23.136032	281.27212	CPD5924	1.1298379	119.156
CPD22974	33.4632232	100.956862	CPD39048	21.2324437	762.318011	CPD5925	1.123833	745.98916
CPD2298	8.1417435	604.275	CPD3906	23.167179	361.2129	CPD5926	1.128857	648.95889
CPD2299	8.1964376	378.093	CPD39063	21.5302221	684.5745	CPD5929	1.1296155	87.1152
CPD22991	33.4574824	114.00842	CPD3907	23.149595	563.26756	CPD59292	5.39937635	415.145825
CPD2300	8.1568337	320.09249	CPD39072	21.541375	443.292425	CPD5931	1.1285349	610.05671
CPD23009	1.05115444	140.01942	CPD3908	23.166749	380.33805	CPD5932	1.1324528	267.93922
CPD2302	8.1873551	373.13692	CPD39081	21.8979995	752.590557	CPD5935	1.131263	663.10573
CPD23023	1.01193497	194.053606	CPD39085	21.9301913	288.265667	CPD59353	6.64415773	360.149486
CPD2303	8.2333275	766.69619	CPD3910	23.163497	340.30973	CPD59377	6.94899962	510.711633
CPD2304	8.2366457	154.09876	CPD3911	23.166293	371.24221	CPD59386	7.10471359	326.115557
CPD2305	8.2097125	178.05691	CPD3912	23.145864	342.31734	CPD5939	1.1516082	692.04264
CPD2306	8.2136432	1031.2842	CPD3914	23.180463	965.6818	CPD5940	1.1177011	142.06816
CPD2307	8.2239855	503.17777	CPD39147	22.3691427	1061.54577	CPD5942	1.1252658	403.86959
CPD23076	1.04662501	557.82905	CPD3915	23.159472	246.23346	CPD59455	7.61956374	589.13301
CPD2309	8.2692726	1346.371	CPD39150	22.3718998	1272.515	CPD59469	7.69988446	663.643778
CPD2311	8.3204922	772.2054	CPD3917	23.192047	664.28442	CPD5947	1.2225392	493.14212
CPD2312	8.2489985	530.163	CPD3919	23.361507	704.46961	CPD59495	7.80071098	372.106043
CPD2313	8.2510033	1527.4029	CPD3920	23.194945	664.45356	CPD5950	1.151	544.04574
CPD2315	8.1415492	1302.355	CPD3922	23.069735	382.27114	CPD5951	1.1361336	650.10144
CPD2317	8.269104	765.20104	CPD39226	22.9274998	762.287017	CPD5952	1.3656184	575.04398
CPD23174	1.07582176	141.018894	CPD3926	23.221035	792.51988	CPD5953	1.2408134	559.07069
CPD2318	8.2623896	779.17412	CPD3927	23.160606	787.56205	CPD59544	7.81886042	590.195383
CPD23180	1.0778494	278.04041	CPD39274	23.3196666	672.260817	CPD5955	1.2111562	68.026008
CPD2319	8.2478342	1527.906	CPD3928	23.209546	808.50091	CPD5956	1.1384126	86.037737
CPD23191	1.06033485	595.895355	CPD39285	23.5028575	790.015757	CPD5958	1.1608748	481.09814
CPD23199	1.10470958	275.120078	CPD3930	23.213503	438.12684	CPD5959	1.1856141	116.01265
CPD2320	8.1175174	1140.303	CPD3932	23.25617	548.52423	CPD5961	1.3440099	514.06826
CPD23207	1.23624241	74.0703571	CPD3933	23.357445	693.52159	CPD5962	1.1521548	484.09236
CPD23209	1.15564871	204.110745	CPD39339	23.8808749	746.297663	CPD5963	1.1619999	204.03153
CPD2321	8.1727989	448.10075	CPD3934	23.262017	470.35104	CPD59643	8.18279626	878.71904
CPD23212	1.2229775	111.998306	CPD39359	24.1775559	1018.56062	CPD5965	1.154463	306.07217
CPD23214	1.28438067	103.062777	CPD3936	23.293883	267.25062	CPD5966	1.1462843	254.09079
CPD2322	8.2493343	1535.8915	CPD39360	24.1791994	1356.5075	CPD5967	1.1636052	528.08669
CPD23224	1.56039994	216.05168	CPD39369	24.3494285	1323.50601	CPD5968	1.1617989	443.98646
CPD23229	1.56775034	304.052925	CPD3938	23.198718	679.31221	CPD59686	8.1831153	886.204677
CPD23230	1.25655639	389.162232	CPD39382	24.8410001	841.559156	CPD5969	1.1561308	360.0507
CPD2324	8.1444978	700.15697	CPD3939	23.2695	465.39154	CPD59722	8.37611674	1240.58092
CPD23241	1.73379578	285.163889	CPD39409	25.5333322	596.300467	CPD5977	1.3442511	122.04498
CPD23249	1.70759038	287.963887	CPD39410	25.6101422	1234.54581	CPD5978	1.1603333	176.04037
CPD2325	8.2525798	898.27256	CPD39421	25.8093716	618.244	CPD5979	1.3842465	72.021548
CPD23253	1.58411852	157.099406	CPD3943	23.320546	823.54389	CPD5980	1.1511343	424.06322
CPD2326	7.9505508	978.26221	CPD39438	27.0703761	411.368538	CPD59817	8.51822782	1339.36505
CPD2327	8.2150192	781.6767	CPD3944	23.331199	844.47537	CPD5982	1.1486429	243.08689
CPD23271	2.51240491	357.090782	CPD39448	27.689339	390.0995	CPD5983	1.1837229	438.06131
CPD2329	8.2697137	582.15753	CPD39457	27.7118283	378.233325	CPD5984	1.149255	180.0631

CPD2330	8.250697	1508.4259	CPD39460	27.7175528	180.184933	CPD5985	1.1702002	321.10314
CPD2331	8.2489478	1535.3892	CPD3947	23.334484	828.49795	CPD5988	1.1660559	588.06658
CPD2332	8.2358719	736.21881	CPD39476	27.9508871	735.525733	CPD59918	9.0893059	1230.57059
CPD23322	6.44099769	486.045392	CPD3948	23.273398	743.53841	CPD59922	9.02888387	970.039586
CPD23324	6.43718124	212.035733	CPD3950	23.340192	622.3979	CPD59936	9.22132362	224.0911
CPD2333	8.2694216	778.67279	CPD3951	23.329878	238.22836	CPD59943	8.90020256	999.43973
CPD23337	7.11281949	488.24728	CPD3952	23.366661	267.24647	CPD59944	9.61414825	1230.57967
CPD2334	8.1560381	781.17968	CPD3953	23.364886	784.47286	CPD59949	9.37930346	980.450243
CPD23342	6.72231292	328.156283	CPD39541	1.02075792	298.032022	CPD59957	9.45716418	1201.84313
CPD2335	8.2654565	773.6893	CPD3955	23.292788	338.24796	CPD5996	1.1928925	221.07512
CPD23356	7.70461366	190.097488	CPD3956	23.377013	779.51978	CPD59962	9.35588789	968.84723
CPD23365	8.10925944	474.507074	CPD3957	23.379281	404.34483	CPD5997	1.178139	384.10459
CPD2337	8.2729091	773.19127	CPD3958	23.385191	426.32669	CPD5998	1.5385656	167.05458
CPD2338	8.2465473	171.12441	CPD39586	1.10866632	622.9444	CPD59986	8.99624684	1237.78679
CPD23385	8.24667891	498.204138	CPD39588	1.24292588	646.0999	CPD59994	9.54645832	967.4381
CPD23388	8.32001681	218.093992	CPD39592	1.11599994	1122.0214	CPD6000	1.4831254	239.07788
CPD23397	8.90894462	335.175936	CPD39617	1.10150614	459.014356	CPD6004	1.219196	291.1282
CPD2340	8.5327648	286.04741	CPD39618	1.19491364	214.00885	CPD60040	19.9075723	569.2419
CPD23402	8.65579071	346.1104	CPD3962	23.35781	704.46986	CPD60041	19.9051665	507.320125
CPD2341	8.2585699	784.17747	CPD39621	1.18721223	105.043171	CPD60042	19.9214992	591.27199
CPD23413	9.06512474	212.082423	CPD39622	1.44907651	478.016469	CPD60043	19.9020855	404.2559
CPD2342	8.256701	776.19907	CPD3963	23.465302	359.35464	CPD60045	20.3058217	449.27849
CPD2344	8.3469365	610.15433	CPD3964	23.394982	699.51259	CPD60052	21.0363001	424.28264
CPD2345	8.215728	232.175	CPD39641	3.21985659	295.199843	CPD6006	1.198333	281.17791
CPD2346	8.2877576	653.25168	CPD3966	23.472109	754.50177	CPD60061	21.9959999	502.360355
CPD23467	10.0050843	422.205243	CPD39663	6.43285098	794.221871	CPD6007	1.4416564	379.96987
CPD23478	10.6690635	264.137057	CPD39665	6.61193273	312.086792	CPD6009	1.1673072	341.1326
CPD2348	8.3348538	456.16516	CPD3968	23.472581	812.50402	CPD6011	1.1801824	359.14166
CPD2349	8.2891025	765.69451	CPD3969	23.620582	572.39137	CPD6012	1.1621313	158.04711
CPD2351	8.3037612	218.09221	CPD39702	8.51176428	126.0314	CPD6017	1.2133713	348.02124
CPD2352	8.3639556	394.18278	CPD39707	8.15244571	350.2263	CPD6019	1.1172785	267.16791
CPD2353	8.3529176	555.26751	CPD3971	23.42981	435.41935	CPD60257	1.14050006	408.273538
CPD2354	8.3714526	426.1857	CPD39711	8.24311498	320.090012	CPD6028	1.2968013	445.72613
CPD2355	8.3257447	607.37536	CPD39712	8.27185493	539.156343	CPD6032	1.2931621	311.97406
CPD23551	13.357169	220.109767	CPD39715	8.45614274	172.103829	CPD60322	2.04556755	296.090514
CPD23556	13.4973125	234.160658	CPD3972	23.432533	660.44437	CPD6034	1.3031439	311.79988
CPD23562	13.6654269	632.406371	CPD3973	23.487383	655.48652	CPD60369	2.13460678	431.138622
CPD2359	8.4313136	787.2851	CPD3974	23.401238	404.34644	CPD60379	2.48345656	431.13805
CPD2360	8.4676332	1110.3104	CPD3975	23.226593	326.32795	CPD6040	1.4590886	197.08747
CPD23602	15.2652245	332.197913	CPD39767	10.3873749	844.292312	CPD60425	5.19399135	132.076086
CPD2361	8.3893698	1505.8852	CPD3977	23.529482	360.33295	CPD60434	5.65443074	356.074271
CPD2363	8.3910005	500.15227	CPD39779	11.4345366	490.178071	CPD60436	5.72516666	138.077467
CPD2364	8.4057261	224.14049	CPD39783	11.6471115	448.149989	CPD60437	5.83955284	146.075278
CPD2365	8.4011037	758.68406	CPD3979	23.50756	454.12052	CPD6044	1.3690272	434.02396
CPD2366	8.4009487	750.19553	CPD39794	12.6338328	868.284258	CPD6046	1.3593545	425.93709
CPD2367	8.3957485	1478.415	CPD3980	23.55902	547.87955	CPD60461	6.70732826	710.358389
CPD2369	8.3703583	706.20973	CPD3981	23.537509	377.34128	CPD60462	6.7171529	710.863114
CPD2370	8.396403	766.67175	CPD39822	14.3066661	644.246011	CPD6047	1.2971847	244.00089
CPD23709	19.0782551	746.4203	CPD39830	14.3741158	691.569714	CPD6050	1.4153058	139.01853
CPD2372	8.209459	772.19878	CPD39836	14.6970094	720.267422	CPD6055	1.4350959	201.99528
CPD2373	8.3981053	766.17089	CPD3984	23.539613	382.30165	CPD60555	7.2261138	184.020075
CPD2374	8.3969994	758.18224	CPD3986	23.546326	281.27241	CPD6057	1.3660111	465.97215
CPD2375	8.3964106	885.28768	CPD3987	23.561626	264.24347	CPD6059	1.2001608	220.01892
CPD23758	20.3339822	429.317191	CPD39872	15.3263334	662.229683	CPD6061	1.4533858	116.01126
CPD2376	8.4027441	750.69499	CPD39874	15.4879654	462.355529	CPD6065	1.4475307	153.00086
CPD2377	8.4128369	751.19024	CPD39880	15.7895271	353.236644	CPD6066	1.43919	470.0788
CPD2378	8.4394724	868.26191	CPD39889	16.7580018	664.2535	CPD6069	1.3880724	285.91033
CPD23780	20.8861427	775.3225	CPD3989	23.605586	585.90865	CPD6070	1.4136671	393.98261
CPD23781	21.0010687	594.249769	CPD3990	23.540933	549.86233	CPD6071	1.4608968	320.95773
CPD2379	8.3994512	448.10014	CPD39908	17.2111297	632.246975	CPD60728	8.669998	1359.58776
CPD2380	8.4599162	619.12075	CPD39909	17.2754166	471.182792	CPD60765	8.93588651	770.36745
CPD2381	8.4333762	1346.3704	CPD3991	23.574474	611.4605	CPD6078	1.5496535	156.00591
CPD2382	8.4677031	347.15868	CPD39916	17.3166251	456.941137	CPD60785	8.85082564	1023.09505
CPD23827	21.6677639	562.225353	CPD39917	17.2971867	468.341875	CPD6079	1.4247561	196.04197
CPD23828	21.6678219	550.258412	CPD39926	17.5971434	744.304543	CPD60812	9.1361348	1234.29336
CPD2383	8.2481119	692.1691	CPD3993	23.526012	616.41699	CPD6082	1.3029911	362.04695
CPD23834	21.7968996	566.25525	CPD39933	18.2854856	407.299614	CPD60822	9.10864721	1241.04447

CPD2384	8.2512285	692.66657	CPD39934	17.8559999	482.266478	CPD6083	1.5666363	403.14808
CPD23842	22.1202887	782.459875	CPD39941	18.2232861	704.301357	CPD60853	9.2058335	1242.5424
CPD2385	8.4569085	311.19106	CPD3996	23.617769	755.48058	CPD6086	1.5669006	367.1518
CPD23859	22.4164994	550.25941	CPD39965	19.5344799	712.259483	CPD6088	1.4301867	123.03327
CPD2386	8.4386753	319.10746	CPD39967	19.199143	672.593743	CPD6090	1.4738808	168.02673
CPD2387	8.4471739	316.14533	CPD39969	19.19728	672.500186	CPD60909	18.1828018	479.28868
CPD2388	8.4984511	821.30705	CPD39976	19.3379999	710.731683	CPD6091	1.4446424	299.01951
CPD23880	23.1549092	652.289573	CPD3998	23.648471	588.55278	CPD60913	19.0563805	1258.67723
CPD23894	23.4439891	810.49148	CPD39980	19.3576251	957.342475	CPD60915	19.9151429	273.134186
CPD2390	8.4573851	236.14198	CPD4001	23.364313	642.24629	CPD60916	19.9056668	386.246122
CPD2391	8.4865032	787.28655	CPD4002	23.67347	307.2874	CPD60922	21.032667	509.339611
CPD23918	24.3198324	642.240867	CPD40022	20.3198181	511.407509	CPD6093	1.5641249	289.12791
CPD2392	8.4088716	781.17675	CPD4003	23.88021	810.49048	CPD6094	1.5581251	78.015313
CPD2393	8.4806767	676.19895	CPD40044	20.460713	327.276957	CPD6099	1.4709474	149.15918
CPD23930	25.4416478	636.294225	CPD4005	23.673336	452.21204	CPD6100	1.5501323	237.07832
CPD23935	25.6657935	266.2601	CPD40064	21.1625015	375.170291	CPD6101	1.5352657	149.05081
CPD2394	8.5472755	1448.4022	CPD4007	23.689699	930.79082	CPD6106	1.495905	510.10484
CPD2396	8.5527843	1508.4227	CPD40075	21.061175	690.266211	CPD61077	1.05214696	351.9668
CPD23971	31.5350468	569.486558	CPD4010	23.732657	931.17113	CPD61135	2.11317449	640.2309
CPD2398	8.5109992	843.29126	CPD4011	23.637287	567.43435	CPD6114	1.4833264	165.06372
CPD2399	8.6923365	435.21167	CPD40111	21.692624	904.3853	CPD6116	1.5639523	132.03149
CPD2400	8.5913034	250.17584	CPD40119	21.8140393	770.57235	CPD6119	1.2941286	159.08938
CPD2401	8.5790178	492.25167	CPD4012	23.681812	925.57573	CPD612	1.017391	188.1511
CPD24014	33.2169729	81.0166133	CPD4013	23.683371	930.53116	CPD6121	1.5517972	160.03787
CPD24015	33.2422161	132.964836	CPD4014	23.692585	584.44335	CPD6122	1.574891	246.12109
CPD2402	8.601751	516.14741	CPD40157	22.144729	746.315691	CPD6123	1.5765951	410.03153
CPD2403	8.5807991	393.10439	CPD4016	23.697075	946.50387	CPD6124	1.5693152	313.09038
CPD2404	8.6023331	757.24075	CPD40165	22.1617999	1004.52707	CPD6125	1.5613862	420.03991
CPD2405	8.7055538	389.09103	CPD40199	22.4575715	441.327943	CPD61258	9.00737407	1222.02561
CPD2406	8.5860143	762.19643	CPD4020	23.693363	668.28413	CPD6126	1.5654419	330.03755
CPD24069	1.03213333	253.992947	CPD4021	23.70515	998.51782	CPD6128	1.5726428	396.99127
CPD24071	1.02652525	503.88295	CPD4022	23.704451	988.49341	CPD61285	9.01386075	815.7141
CPD2408	8.6083872	302.98203	CPD4023	23.671218	572.39112	CPD6129	1.5661999	91.010075
CPD24089	1.04000002	587.843186	CPD4025	23.752089	541.88391	CPD6130	1.5642024	113.04527
CPD2409	8.5820538	706.20926	CPD4026	23.696323	497.2717	CPD61302	9.31858686	973.642355
CPD2411	8.6086594	261.95654	CPD4027	23.751299	316.29581	CPD6131	1.6864753	170.02155
CPD24114	1.06199892	178.976025	CPD4028	23.742746	543.86038	CPD61325	9.81927572	1216.56604
CPD2413	8.6039596	351.02421	CPD4029	23.911389	435.35614	CPD6135	1.5758333	817.97043
CPD2414	8.5994252	270.00723	CPD40295	23.1885737	923.515582	CPD6136	1.4922424	544.05523
CPD2415	8.6081692	355.18166	CPD4030	23.72087	452.21214	CPD6137	1.5487702	490.01966
CPD24161	1.05984616	364.984262	CPD4032	23.714863	528.36402	CPD6145	1.3807833	427.19458
CPD2417	8.6028185	162.03144	CPD4033	23.419855	435.41925	CPD6146	1.5753075	650.96607
CPD2418	8.6222699	209.00785	CPD4035	23.726428	523.40806	CPD6148	1.5855887	227.02481
CPD24190	1.0872144	166.014579	CPD40350	23.9707145	442.3656	CPD6150	1.5701473	376.05034
CPD24201	1.148938	160.084305	CPD4038	23.911655	889.63726	CPD6151	1.5727085	388.01583
CPD2422	8.6072352	141.01503	CPD40395	25.4017985	674.25685	CPD6153	1.5787399	244.00232
CPD24222	1.13392323	546.067808	CPD40402	25.817854	523.324657	CPD6157	1.3823262	528.08837
CPD24227	1.14041184	417.149929	CPD4043	23.850184	521.84822	CPD6158	1.654929	293.2171
CPD2423	8.6156001	335.10068	CPD4044	23.808372	952.57123	CPD6161	1.3982474	257.10129
CPD2425	8.6059991	161.02425	CPD4045	24.101667	845.60927	CPD617	1.009186	265.1521
CPD24259	1.08781278	322.974646	CPD4046	23.856118	479.38328	CPD6170	1.5108977	54.011582
CPD2427	8.5955489	423.99028	CPD4047	23.850108	269.27182	CPD6171	1.4431537	269.94061
CPD2428	8.6257789	464.14389	CPD4048	23.850937	519.37361	CPD6173	1.7074069	352.0145
CPD2429	8.602885	210.01231	CPD4049	23.927169	291.25251	CPD6177	1.7314077	236.07922
CPD24292	1.72476279	116.0687	CPD4050	23.83061	484.33766	CPD6178	1.5593465	371.99079
CPD24311	1.71944633	336.05579	CPD4054	23.92968	977.68291	CPD6181	1.702606	355.99419
CPD24312	1.94152413	143.0568	CPD4059	23.865808	810.48959	CPD6186	1.5877983	341.13194
CPD24313	1.8017769	155.049529	CPD4061	23.939766	499.83495	CPD61869	8.62800853	1087.87048
CPD2432	8.6971046	623.25951	CPD4062	24.105425	399.36739	CPD6189	1.8373728	186.06465
CPD24324	2.3399553	103.09883	CPD4063	23.953352	600.4169	CPD619	1.004003	274.9539
CPD2434	8.6607604	530.1636	CPD4064	24.000839	477.82353	CPD6191	1.204829	130.0137
CPD2436	8.6857986	486.1378	CPD40647	6.66310454	164.047314	CPD61923	10.2300947	466.137833
CPD24364	5.01288613	242.045912	CPD4065	23.990759	440.31085	CPD61927	10.297499	333.085483
CPD2437	8.6428683	448.17101	CPD4066	23.970085	435.35625	CPD6193	1.8322032	109.00176
CPD2438	8.7957876	218.09158	CPD40664	6.9026634	208.10925	CPD6194	1.8436566	322.06176
CPD2439	8.6752654	224.06825	CPD4067	23.938239	710.5051	CPD6195	1.8290011	350.09354
CPD24392	6.06870083	145.053285	CPD4068	23.99311	933.65738	CPD6198	1.8611252	321.0971

CPD2440	8.6121367	732.18752	CPD40681	7.38249919	314.122833	CPD620	1.018817	95.99861
CPD2441	8.7167253	808.31257	CPD40697	7.76652605	351.155989	CPD6200	1.8389962	73.026088
CPD2442	8.736398	623.25952	CPD40704	7.84874982	331.144475	CPD6204	1.6426965	221.01758
CPD2443	8.7471721	727.23138	CPD40725	8.20212017	574.222971	CPD62042	18.1847778	421.245844
CPD24432	7.41625656	286.104671	CPD40736	8.56147062	272.160909	CPD62043	18.2442865	387.298
CPD24434	7.64638051	442.289213	CPD40737	8.41613632	451.204171	CPD62044	18.8965006	612.29005
CPD2444	8.7995421	500.15253	CPD40740	8.60048913	742.223136	CPD62048	19.3088582	503.286486
CPD24452	8.12199102	298.12944	CPD4077	24.101029	788.50677	CPD62054	19.7909263	545.38835
CPD24453	8.34211123	310.120785	CPD40773	9.14785761	962.648323	CPD62056	19.9065718	534.2834
CPD2446	9.2067379	230.04348	CPD40774	9.16118224	962.849081	CPD62058	19.8915714	448.764229
CPD2448	8.8788857	537.21338	CPD40778	9.06572089	988.253857	CPD62059	19.8992856	779.952986
CPD24480	7.81857487	950.232691	CPD4079	24.110272	586.45395	CPD62060	19.9118341	566.359917
CPD2449	8.576256	542.17685	CPD40791	9.15190875	268.131145	CPD62061	19.9015001	518.306633
CPD2450	8.832584	387.15218	CPD40792	9.15420269	386.09706	CPD62062	19.9050006	507.514867
CPD2451	8.8302058	355.22088	CPD4080	24.033473	894.59229	CPD62064	19.8994285	929.584557
CPD2452	8.8302219	360.17423	CPD4081	24.105246	396.28521	CPD62066	19.9048758	526.795162
CPD2453	8.848491	246.09783	CPD4082	24.113375	889.6333	CPD62068	19.9455049	659.256629
CPD24557	10.5766469	418.093436	CPD40821	9.99779857	472.22779	CPD62070	19.9226255	597.289687
CPD24568	10.9601263	678.2552	CPD40827	10.286183	553.272429	CPD62071	19.9073334	518.8065
CPD2459	8.9657835	334.15185	CPD4083	24.251125	713.53197	CPD62075	19.9091671	534.781683
CPD2460	8.9805342	792.28275	CPD40832	10.315091	553.272533	CPD62086	20.413629	422.266714
CPD24604	14.7511454	688.274	CPD4084	24.185977	622.27978	CPD62091	20.6224286	612.2933
CPD2461	8.9551778	606.20348	CPD4086	24.252372	644.26066	CPD62093	20.6254287	607.336843
CPD2464	9.0440286	572.26066	CPD4087	24.142382	391.32908	CPD62097	21.0418722	406.271975
CPD24640	17.5380597	640.287673	CPD4088	24.106445	845.60982	CPD6210	2.0939564	285.13234
CPD2465	9.029717	479.12204	CPD40880	10.6409232	519.231933	CPD62109	21.5698334	545.3428
CPD24654	18.3808322	688.275933	CPD40882	10.9563339	462.208067	CPD62110	21.8014999	590.382237
CPD2466	9.0114573	408.17634	CPD40883	10.9566361	426.187782	CPD62112	21.9881667	992.650833
CPD24672	19.5392277	282.23034	CPD40884	11.1625002	995.322667	CPD62114	21.9958749	523.2944
CPD24677	19.8399284	1012.51611	CPD40889	11.7141975	708.26315	CPD62116	22.0068574	575.309014
CPD2468	8.8240866	942.27409	CPD4089	24.192681	579.38561	CPD6212	1.7541124	109.00239
CPD24681	20.3688562	357.267189	CPD4090	24.209515	578.88186	CPD62121	22.5417499	521.3333
CPD24686	20.4834472	428.311745	CPD40906	12.4287169	518.685917	CPD62124	22.9149804	875.615583
CPD24689	20.6491998	693.228687	CPD4091	24.190217	850.56509	CPD6214	1.8825593	186.06426
CPD2469	9.032946	696.22615	CPD4092	24.184514	377.33841	CPD622	1.011	419.8867
CPD24696	21.2346609	686.29359	CPD4093	24.259354	311.35386	CPD6221	2.1485428	281.14467
CPD24706	21.0816658	600.4133	CPD40930	13.9131812	661.557834	CPD6222	2.1680952	162.0525
CPD24709	21.0897154	690.26799	CPD4094	24.208381	576.40079	CPD6223	1.6912917	159.02037
CPD2471	9.0088968	937.3202	CPD4095	24.271977	669.5045	CPD6228	2.0831661	182.03937
CPD24715	21.1627667	693.230741	CPD4096	24.208172	382.30029	CPD623	1.012883	265.9171
CPD2472	9.0368997	764.32449	CPD40965	14.7358739	588.223063	CPD6234	2.2898517	348.07597
CPD24722	21.7530985	674.272825	CPD4097	24.203662	622.28094	CPD6237	2.4861236	187.06306
CPD24726	21.7853315	805.333814	CPD40970	14.8783743	1462.50917	CPD6245	2.5255478	358.08718
CPD24728	22.0167081	716.305273	CPD40979	14.9735199	676.809333	CPD6246	2.234338	345.07874
CPD24729	22.0217227	716.30435	CPD4098	24.232197	735.48887	CPD625	1.02164	306.9477
CPD24731	22.1240428	626.272388	CPD4099	24.239943	756.42428	CPD6253	2.5341777	165.07998
CPD24732	22.1586098	789.337507	CPD40994	15.2460927	618.240075	CPD6262	3.2061593	145.10851
CPD24734	22.1999337	347.305	CPD40995	15.245135	314.291643	CPD62624	7.86200519	646.311943
CPD24736	22.1999999	692.247118	CPD4100	24.373508	674.46869	CPD62625	7.8545117	646.813917
CPD2474	9.1283633	399.2434	CPD41004	15.5056669	963.461867	CPD627	1.010208	249.9406
CPD24750	22.7248117	730.3219	CPD4101	24.203764	740.44782	CPD62706	9.19551815	1207.31302
CPD24752	22.6809243	553.169327	CPD41011	15.9395322	472.172925	CPD62784	20.4576245	521.335637
CPD24758	22.9149821	664.240825	CPD4102	24.240798	824.20742	CPD628	1.0117	144.1628
CPD2476	9.1275162	162.1246	CPD4103	24.23972	556.40591	CPD6281	5.1060831	181.04432
CPD24763	22.8985396	255.256271	CPD41033	16.5127774	734.283775	CPD6283	5.1004215	98.072294
CPD2477	9.144605	305.1295	CPD4104	24.259087	956.54821	CPD6285	4.7855942	280.09392
CPD24770	23.1342752	642.467894	CPD4105	24.262567	878.2721	CPD6289	5.2722243	280.10438
CPD24776	23.2398287	730.32128	CPD41050	17.1617142	738.1935	CPD629	1.018169	349.8784
CPD2478	9.19542	592.1636	CPD4106	24.279832	470.09766	CPD6294	5.3501506	354.10441
CPD24786	23.7576074	666.2686	CPD4107	24.270507	801.58266	CPD6295	5.4467568	180.04209
CPD2479	9.1816337	634.26179	CPD41078	18.1556649	394.235882	CPD630	1.034316	105.0417
CPD2480	9.2239303	196.11005	CPD4109	24.223077	822.21177	CPD6300	5.7166543	196.1546
CPD24801	24.7922703	454.327833	CPD4110	24.301498	396.28089	CPD6301	5.8587225	394.08798
CPD24804	25.0774613	609.1656	CPD41111	18.246861	770.278443	CPD6306	5.8417701	392.07294
CPD2482	9.285454	516.15007	CPD41113	18.4698334	748.297017	CPD63084	1.10599602	539.17638
CPD24829	26.7638167	696.439581	CPD41118	18.5477053	993.484986	CPD631	1.162821	315.999
CPD24839	27.7270931	468.323557	CPD4112	24.323678	632.1464	CPD6310	5.847997	967.06603

CPD2484	9.2971225	1150.3236	CPD41127	18.7471113	700.275578	CPD63122	1.65737485	389.202825
CPD24842	28.7584878	639.5269	CPD4113	24.230349	845.607	CPD6314	5.8427143	951.09227
CPD24846	30.0177263	784.513692	CPD41133	19.1330148	716.3031	CPD6315	5.8513435	816.16129
CPD2485	9.2965319	1525.4876	CPD41135	19.0555714	738.285543	CPD6316	5.8543058	497.16156
CPD2486	9.300867	1150.8244	CPD41139	19.1766672	612.2624	CPD63167	2.12480431	633.17492
CPD24864	33.1051161	203.141473	CPD4114	24.333703	627.18785	CPD6318	5.8532229	713.02959
CPD2487	9.2938888	1562.3806	CPD4116	24.348439	340.29218	CPD6319	5.929655	318.12791
CPD24874	33.1167706	193.151712	CPD41164	19.4641433	654.2697	CPD632	1.017333	433.8402
CPD2488	9.3042239	388.10121	CPD4117	24.26831	801.5833	CPD6320	6.0872081	135.06558
CPD2489	9.306504	292.0411	CPD41175	19.5588333	1200.58442	CPD6321	6.0274797	610.15525
CPD2490	9.2987496	773.70753	CPD4118	24.354534	358.3083	CPD6322	6.0554283	296.1466
CPD2491	9.3015019	773.22858	CPD4120	24.267805	806.54129	CPD6323	5.986225	200.11021
CPD24910	1.01556524	154.02662	CPD41201	19.8540143	700.279967	CPD63238	5.17399624	454.205486
CPD2492	9.3025443	771.25709	CPD41208	19.9633332	1318.53653	CPD6325	6.0588475	399.15247
CPD2494	9.2979467	1530.4426	CPD4122	24.358661	448.27855	CPD6326	6.1013387	355.12575
CPD2495	9.3005299	1032.2181	CPD41238	20.586416	84.0871167	CPD63265	5.8743995	498.23252
CPD24952	1.07371177	286.005394	CPD4124	24.362267	625.47713	CPD63267	5.91159895	573.27792
CPD2496	9.3025219	530.16342	CPD4125	24.365869	630.43674	CPD6327	6.0900728	730.11825
CPD2497	9.3049999	396.09002	CPD4126	24.340802	757.5596	CPD6329	6.0922179	248.07789
CPD2498	9.3045312	350.1014	CPD4127	24.387396	762.52004	CPD633	1.014857	211.9945
CPD2499	9.3012498	531.36689	CPD41285	21.1951668	1383.48797	CPD6330	6.1341996	389.12988
CPD2500	9.2888206	368.11065	CPD41297	21.2692274	375.170118	CPD63331	7.04716953	168.091267
CPD25008	1.09222602	270.984627	CPD4130	24.461182	798.49009	CPD6335	6.2125111	341.16624
CPD2501	9.1808405	776.21364	CPD41308	21.501497	598.278825	CPD6336	6.30115	1258.341
CPD25011	1.31662602	502.966361	CPD4131	24.457226	944.54575	CPD63374	8.10200179	708.36156
CPD25015	1.22806997	171.978119	CPD4132	24.454383	295.28732	CPD6339	6.3519494	376.1693
CPD2503	9.2971654	776.45298	CPD4133	24.635308	581.45102	CPD6340	6.5637725	575.61971
CPD25036	1.43663153	52.0109308	CPD4135	24.488776	444.35558	CPD6343	6.4248694	448.10007
CPD25038	1.52609304	224.056229	CPD4137	24.743307	841.66007	CPD6347	6.443531	398.1558
CPD2504	9.2938669	224.07026	CPD4139	24.527591	600.41734	CPD6349	6.4180936	254.11646
CPD2505	9.2392833	838.18655	CPD4140	24.500499	718.49733	CPD63495	8.37379317	1036.30406
CPD2506	9.307746	602.09238	CPD41404	22.0546921	766.281314	CPD6350	6.4450006	454.12316
CPD25075	2.97367336	222.047218	CPD41407	22.3566667	1329.49023	CPD63542	8.55455879	384.1381
CPD25094	6.55588028	387.164812	CPD41410	22.3669999	736.6299	CPD6356	6.5730079	125.08267
CPD25109	6.93968002	224.06786	CPD41415	22.3746667	618.254383	CPD6357	6.5989288	226.16663
CPD2511	9.3439796	404.07587	CPD41417	22.3925	1244.5195	CPD6359	6.4557851	248.15758
CPD25113	7.40757803	146.036312	CPD4142	24.513361	713.53508	CPD636	1.039537	85.08968
CPD2512	9.3011631	746.31418	CPD4147	24.583766	537.42505	CPD6360	6.7793348	307.10526
CPD2513	9.3267448	822.32946	CPD4149	24.612961	669.50931	CPD6364	6.8480755	425.1644
CPD25131	9.15999103	312.121921	CPD4152	24.619896	674.47225	CPD6365	6.9079383	357.08974
CPD25137	9.17958918	750.2053	CPD4153	24.70002	630.44338	CPD63676	9.5465593	518.14366
CPD2514	9.3426378	960.37065	CPD4154	24.789159	498.35575	CPD6370	6.9588895	380.10208
CPD2515	9.3349268	504.33901	CPD4155	24.839977	841.65994	CPD63712	9.89485668	560.15228
CPD25155	11.6693426	126.031172	CPD4156	24.689265	625.48113	CPD6372	6.88735	220.05851
CPD2517	9.3524615	206.05874	CPD41563	23.5752729	1298.52682	CPD6374	6.9615242	397.1063
CPD25188	15.0791955	464.177105	CPD4157	24.763151	586.41223	CPD6377	7.0432757	401.2245
CPD2519	9.4040062	722.20466	CPD41577	23.6869981	1344.53342	CPD63771	18.1947998	541.21222
CPD25194	16.6850308	284.194833	CPD4158	24.840431	454.32894	CPD63774	18.1958	358.21506
CPD25195	16.6028454	292.208825	CPD4160	24.820578	625.4882	CPD63776	18.1888576	376.224157
CPD2520	9.4131239	758.70243	CPD4161	24.908209	652.44451	CPD63779	18.190667	563.2402
CPD2521	9.4143362	758.20149	CPD4162	24.885075	812.50424	CPD6378	7.1007849	417.16422
CPD2522	9.4200591	1495.4771	CPD41629	24.189304	623.824817	CPD63790	18.7618335	394.2372
CPD2523	9.4247268	1030.3127	CPD4163	24.882386	807.54791	CPD63791	18.7603349	493.305933
CPD25230	20.366539	376.241167	CPD4164	24.855023	630.45411	CPD638	1.016318	333.9055
CPD25232	20.0915821	369.287071	CPD4166	24.905487	766.4656	CPD6380	7.0825889	370.20652
CPD2524	9.4123493	1120.3175	CPD4167	24.981934	636.47048	CPD63814	19.8841995	456.75082
CPD25242	20.4448167	636.259291	CPD4168	24.881954	537.42484	CPD63816	19.8936001	702.9243
CPD2525	9.4261182	1500.4325	CPD41695	25.8871652	652.509067	CPD63819	19.903	1034.11452
CPD25273	22.146445	306.278323	CPD41696	25.8907015	652.600257	CPD63820	19.9012	787.93998
CPD25278	22.2273993	788.48228	CPD4171	25.065169	841.66016	CPD63821	19.9016	1033.61106
CPD2528	9.4299006	1120.8132	CPD4172	24.783087	542.38168	CPD63822	19.909	1098.58996
CPD25294	23.0111547	414.294031	CPD41725	27.7209937	390.280014	CPD63828	19.9025999	788.4424
CPD25302	22.863156	564.347464	CPD4173	24.954198	605.92473	CPD6383	7.001977	212.03547
CPD2532	9.4453913	1105.3091	CPD4174	24.925372	410.30114	CPD63833	19.9047999	771.9632
CPD2534	9.4440505	1465.4655	CPD4175	24.971412	405.34417	CPD63835	19.9056	826.52098
CPD2535	9.4459977	1470.4221	CPD4176	24.988135	295.28688	CPD63843	19.9061999	1088.56084
CPD2536	9.5263628	773.26178	CPD4177	24.979489	498.35535	CPD6385	7.1603396	441.16106

CPD25363	26.8863165	588.461842	CPD41779	32.5671079	914.718013	CPD63857	19.9225717	587.260643
CPD25365	27.3360872	777.537287	CPD41784	33.1361836	190.144888	CPD63869	20.4551668	404.256233
CPD25367	27.6392545	567.422086	CPD4179	25.036377	1000.5371	CPD6387	7.0432832	158.09364
CPD25369	27.7114972	556.593518	CPD41805	1.04618108	160.128206	CPD63871	20.4541672	1159.72623
CPD2537	9.4409982	1002.2084	CPD4181	24.998757	932.546	CPD63887	21.0320008	451.300733
CPD2540	9.4888662	389.07156	CPD4182	25.061226	605.27756	CPD63890	21.0627999	589.2764
CPD2541	9.4491266	743.21952	CPD4183	25.042301	948.52013	CPD6390	7.3325973	358.16086
CPD2542	9.4437913	1105.809	CPD4184	25.013468	927.58993	CPD63913	21.7500398	585.424671
CPD2543	9.4487507	500.15322	CPD4185	24.79656	493.39897	CPD63916	22.023178	507.51092
CPD2545	9.445215	743.69638	CPD4186	25.116127	665.33829	CPD63918	22.0004	382.27192
CPD2547	9.4777376	741.24788	CPD4188	25.078971	1302.5342	CPD6392	7.4054392	345.08478
CPD2548	9.6141818	792.18825	CPD4189	25.049791	586.4443	CPD6393	7.395317	415.23883
CPD2549	9.2965432	338.1013	CPD4191	25.086342	608.59409	CPD63934	22.5426003	516.37368
CPD25505	1.03386892	292.952833	CPD41912	1.17471459	293.019243	CPD63935	22.546167	400.282133
CPD2551	9.4565554	320.09005	CPD41914	1.21904194	222.049747	CPD6394	7.4596752	465.19863
CPD2552	9.3852841	456.15527	CPD4192	25.008778	610.88311	CPD6396	7.4525441	362.10008
CPD2553	9.4765591	277.03632	CPD41924	1.30166619	376.052622	CPD63963	23.8452009	535.35902
CPD2554	9.442964	778.16633	CPD4193	25.075428	609.59357	CPD6397	7.4103929	360.08127
CPD2555	9.4612502	746.20235	CPD4194	25.038146	607.89781	CPD640	1.020444	517.8012
CPD2558	9.4563529	381.08463	CPD41949	2.00229019	237.100343	CPD6400	7.4611889	445.25042
CPD25597	1.15636536	436.965325	CPD4195	25.078174	608.50197	CPD6401	7.5894501	201.08264
CPD2560	9.4588063	572.08226	CPD4197	25.078339	1271.614	CPD6402	7.7419907	435.14386
CPD25602	1.54395469	129.043212	CPD4198	25.085934	606.28446	CPD6403	7.560508	386.10476
CPD25606	1.52282443	341.13149	CPD41980	5.0493751	158.083375	CPD6410	7.7813363	351.15298
CPD25608	1.55429985	323.03113	CPD41982	5.05824999	408.179	CPD6415	7.9861331	516.14798
CPD2561	9.4270656	404.07659	CPD41988	5.23619992	383.1606	CPD64163	1.72357058	417.03876
CPD2562	9.4668986	746.43565	CPD4199	25.127033	628.56659	CPD642	1.029346	142.0415
CPD25625	2.31005683	223.068763	CPD41992	5.48966658	221.239217	CPD6425	8.1740951	366.14242
CPD25628	2.09614406	285.13285	CPD4200	25.10345	764.2408	CPD6427	8.2004597	129.05741
CPD2563	9.4596657	524.11162	CPD42004	5.89100017	242.1139	CPD6429	8.1835625	90.046848
CPD2567	9.5582805	711.23553	CPD42008	6.05466685	406.151378	CPD643	1.02448	357.9155
CPD25679	7.63480733	759.6738	CPD4201	25.098373	696.25258	CPD6430	8.2280054	766.19671
CPD25692	8.30410581	376.172133	CPD42017	6.30640002	224.101	CPD6433	8.3117767	206.05866
CPD2570	9.5639282	366.08085	CPD4203	25.101821	686.22173	CPD6434	8.2651854	780.6741
CPD2571	9.569629	898.27365	CPD42034	6.66960227	364.063928	CPD6436	8.1434421	700.65766
CPD2573	9.3894059	460.22918	CPD42035	6.75836181	442.0787	CPD644	1.024599	78.98273
CPD25733	9.61809029	603.638825	CPD42039	6.74779926	486.0751	CPD6440	8.1712123	206.05933
CPD2574	9.5669573	716.19134	CPD4204	25.127763	832.22694	CPD6444	8.4576174	294.1645
CPD2575	9.5451328	389.07141	CPD42041	6.7490014	618.0666	CPD6448	8.5561795	474.31688
CPD2576	9.6050025	510.12066	CPD42047	6.83079901	695.23988	CPD645	1.015088	463.8619
CPD2577	9.7300749	438.1889	CPD42050	6.84337534	96.0565875	CPD646	1.039707	278.9369
CPD25770	15.0001786	674.737156	CPD42054	6.86199858	728.2402	CPD6465	8.9029975	370.19502
CPD2580	9.5966865	456.19736	CPD42059	6.97261158	520.192995	CPD6466	8.9104082	211.08576
CPD25800	18.9244849	724.259971	CPD4206	25.219277	631.50924	CPD6467	8.9988877	467.1099
CPD2581	9.6075601	478.18809	CPD4207	25.110646	749.50134	CPD64686	1.17499981	189.05982
CPD2582	9.6793344	247.0746	CPD4208	25.112126	754.46249	CPD647	1.026367	235.8975
CPD2583	9.6541958	743.25281	CPD42082	7.32419985	354.17742	CPD6471	9.0728251	392.10869
CPD25831	20.4607685	791.511208	CPD42089	7.43342987	454.144543	CPD6473	9.0740182	387.15245
CPD2584	9.6082332	224.14062	CPD42092	7.46819545	368.07944	CPD6474	9.1388567	422.26589
CPD25856	21.9628413	910.583408	CPD42098	7.54412428	136.052413	CPD64746	2.20380118	463.16196
CPD25858	21.8921543	423.26068	CPD42099	7.54771455	154.063443	CPD6477	9.1611277	576.19631
CPD25860	21.8110794	950.56245	CPD4210	25.094795	778.35518	CPD6479	9.2660071	588.25685
CPD2587	9.6122076	434.16726	CPD42105	7.61900004	140.083538	CPD648	1.018929	501.8321
CPD25880	23.2212714	592.48165	CPD42106	7.62266678	158.094067	CPD6485	9.3844926	1016.2544
CPD2589	9.7037272	217.20319	CPD4211	25.097606	644.2393	CPD6486	9.42033	491.19677
CPD25890	23.1639752	836.546822	CPD42114	7.67539647	287.17464	CPD6488	9.309845	815.28228
CPD25898	23.663833	690.266672	CPD4212	25.109137	998.57905	CPD6489	9.3369262	982.35369
CPD2590	9.7228155	173.17687	CPD42123	7.84907257	229.031646	CPD6493	9.4169391	664.27074
CPD2591	9.7335773	521.14276	CPD42131	7.90912194	497.228425	CPD6494	9.4700982	373.09571
CPD25915	25.086082	1214.99401	CPD4215	25.118807	783.40444	CPD6495	9.4441434	436.13578
CPD25916	25.0409898	948.518187	CPD42150	8.00923476	133.052925	CPD6497	9.3875484	776.21367
CPD2592	9.7483332	1021.3612	CPD42155	8.17699986	1158.14328	CPD6498	9.4126559	456.15827
CPD2593	9.7599398	902.28256	CPD42158	8.17599886	729.104567	CPD64993	9.45339849	998.64762
CPD25948	27.6840893	634.673921	CPD42159	8.17616497	571.086333	CPD6503	9.6112168	206.13492
CPD2595	9.745348	396.09125	CPD42161	8.18900106	785.06648	CPD6505	9.6258553	434.16797
CPD25951	27.7152443	612.473319	CPD42164	8.3558802	529.15205	CPD6507	9.8792922	458.21455
CPD25954	27.7633751	628.44725	CPD42169	8.20837358	697.165413	CPD6508	9.9306493	210.16032

CPD2597	9.7329461	776.2125	CPD42171	8.22780744	1126.20574	CPD651	1.020667	167.9145
CPD2598	9.7253114	404.0764	CPD42178	8.34299992	314.18266	CPD6511	9.8766659	502.15971
CPD25981	28.9741049	612.687731	CPD42184	8.45674632	379.186874	CPD6513	9.9307315	301.13619
CPD2599	9.7601559	771.25702	CPD42193	8.52027087	384.142138	CPD6514	9.9289444	328.09587
CPD2602	9.7367102	780.26596	CPD4220	25.191817	454.32927	CPD6516	9.9805591	600.25707
CPD2603	9.7611417	530.1632	CPD42201	8.52478248	970.24016	CPD652	1.026319	319.8594
CPD2604	9.6032011	618.20833	CPD42202	8.64893505	594.208213	CPD6522	10.121504	339.19342
CPD26059	1.85136165	236.076846	CPD42206	8.57908818	778.196655	CPD6523	10.198445	210.08971
CPD2609	9.8249753	185.13612	CPD42207	8.80942863	648.259329	CPD6524	10.250399	630.26833
CPD2610	9.8770881	776.31526	CPD42215	8.98212369	574.222363	CPD6526	10.276274	231.18124
CPD2611	9.8922028	572.08199	CPD42220	9.04261794	325.188188	CPD6529	10.500293	662.2587
CPD2613	9.8787041	389.07287	CPD4223	25.253674	636.47017	CPD6533	10.750656	484.19207
CPD2614	9.892054	746.20261	CPD42235	9.26197382	338.180957	CPD6535	10.879627	676.27229
CPD2615	9.8893962	381.08492	CPD42237	9.32579743	594.28714	CPD6536	11.205741	199.15886
CPD2616	9.8905019	741.24664	CPD42239	9.4829631	325.1215	CPD6538	11.231461	190.13637
CPD2617	9.8951501	500.1523	CPD4224	25.251796	964.60936	CPD6539	11.365996	630.26628
CPD2618	9.9181321	447.14086	CPD4225	25.035227	608.90268	CPD654	1.014745	289.9744
CPD2619	9.909735	480.21029	CPD42256	9.61985125	486.207443	CPD6545	11.750876	400.17064
CPD2620	9.9204414	648.27736	CPD42258	9.60859874	779.3467	CPD6546	11.762513	290.18872
CPD2621	9.9339712	602.27307	CPD4226	25.250131	283.28784	CPD6548	11.7181	230.04331
CPD2623	9.9250893	320.10864	CPD42260	9.66269714	396.142763	CPD655	1.017643	320.0166
CPD2624	9.9397889	548.22149	CPD42264	9.58315601	451.201437	CPD6555	12.024972	172.14065
CPD26241	1.07238459	319.958408	CPD42266	9.67000136	641.286833	CPD6556	12.091922	697.24884
CPD26257	1.0666442	210.019788	CPD42267	9.66955441	426.186956	CPD6557	12.293665	644.24648
CPD2627	9.9639019	711.24389	CPD42273	9.89539895	356.19258	CPD6558	12.293169	243.18399
CPD26275	1.29719139	622.025221	CPD42274	9.96120314	826.263733	CPD6563	12.507378	539.22581
CPD2628	9.9502663	212.15113	CPD42276	9.93250008	284.162167	CPD6565	12.524742	481.24974
CPD26281	1.57176008	260.13692	CPD42277	10.0681554	446.212883	CPD657	1.029299	451.8274
CPD26283	1.82113574	52.0116944	CPD42278	9.93414441	486.2078	CPD6584	13.516768	197.17572
CPD2629	9.9709073	716.19267	CPD42280	9.94000317	251.0874	CPD6586	13.807269	227.19057
CPD2631	10.258377	206.05983	CPD42281	9.94100178	259.07405	CPD6589	13.916398	157.14635
CPD2633	10.005587	614.16142	CPD42285	9.99712405	270.181437	CPD659	1.048001	139.9999
CPD2634	9.6813705	492.12615	CPD42286	9.99985663	432.234814	CPD6591	13.968789	224.18682
CPD26342	7.55569951	224.106529	CPD4229	25.302584	537.44949	CPD6592	13.997324	549.30627
CPD26346	7.61642652	386.125708	CPD4230	25.296881	542.40877	CPD6595	14.400743	406.24958
CPD2635	10.045365	506.10848	CPD42304	10.1761998	284.16186	CPD6601	14.771735	251.22271
CPD26359	8.4349097	511.19779	CPD42307	10.2077145	214.069814	CPD6602	14.813948	987.49492
CPD2636	9.8735417	979.27684	CPD42309	10.2115005	407.214713	CPD6604	15.037648	270.10408
CPD26370	8.88078793	359.17485	CPD4231	25.29308	769.55866	CPD6605	14.930712	503.30851
CPD2638	9.7503079	987.25791	CPD42310	10.2166667	210.124633	CPD6606	14.927214	251.1178
CPD26387	9.73290149	999.2626	CPD42311	10.220713	412.167171	CPD6609	14.982241	1005.2575
CPD2639	10.048812	736.22088	CPD42314	10.2743326	295.084267	CPD66094	9.1898012	1032.2714
CPD2640	10.055146	736.45412	CPD42316	10.2672845	558.227657	CPD661	1.030966	251.8765
CPD26403	9.99576275	339.202989	CPD42317	10.3198548	313.224486	CPD6613	15.188878	243.25409
CPD2641	10.046052	507.10483	CPD42318	10.4699999	761.337567	CPD6614	15.291204	315.2797
CPD2642	10.036999	979.76522	CPD4232	25.318	561.44612	CPD6617	15.362967	464.17696
CPD2643	10.091901	982.27128	CPD42321	10.4958002	168.11304	CPD6618	15.334598	248.17253
CPD2645	10.044401	499.11854	CPD42322	10.4913333	132.0923	CPD662	1.029474	555.7738
CPD2646	10.042301	977.31405	CPD42327	10.5540106	617.291129	CPD6621	15.423202	190.14235
CPD26460	16.5870237	283.12263	CPD42328	10.5788289	431.232117	CPD6623	15.446184	464.17741
CPD26462	16.7508031	604.289369	CPD4233	25.385545	774.52673	CPD6626	15.423368	316.20502
CPD26466	16.9472	356.31297	CPD42338	10.9451999	250.07862	CPD6629	15.859083	267.21922
CPD2647	10.04277	491.12998	CPD42339	10.9575985	484.19298	CPD6630	15.88061	370.31805
CPD26479	18.4116121	317.2335	CPD42340	10.9567996	444.19796	CPD6632	16.179783	180.11567
CPD2648	10.011335	368.1108	CPD42341	10.9545007	761.337617	CPD6633	16.072555	436.18923
CPD2649	9.8969431	456.19849	CPD42346	11.1228272	601.301442	CPD6639	16.178213	464.19075
CPD2651	10.057617	681.23844	CPD42347	11.1325568	284.161756	CPD664	1.032643	523.7709
CPD26518	21.0492014	868.515621	CPD4235	25.307694	410.3027	CPD6641	16.254128	243.21704
CPD2652	10.066992	233.19766	CPD42353	11.504167	483.15935	CPD6645	16.393888	276.07625
CPD2653	10.121001	1049.3474	CPD42354	11.5004991	305.1993	CPD6647	16.547088	552.26951
CPD26537	22.0357984	866.55559	CPD4236	25.33639	405.3455	CPD665	1.028611	80.98034
CPD2654	10.133185	224.06864	CPD42368	12.0881254	503.270588	CPD6655	16.658695	380.12755
CPD2655	10.17587	618.26437	CPD4237	25.44338	1026.5487	CPD6660	16.96739	269.23502
CPD2656	10.221674	662.25744	CPD42370	12.1593739	721.351138	CPD6662	16.965077	255.28962
CPD2657	10.173365	950.27488	CPD42376	12.2558332	429.2478	CPD6663	16.95545	335.22245
CPD2658	10.16167	986.32017	CPD42389	12.9508427	503.1889	CPD6666	17.132909	269.23369
CPD2659	10.163519	338.1001	CPD42393	13.3207878	503.190267	CPD6668	17.145484	1044.3249

CPD26608	27.37157	782.497477	CPD42398	13.3926003	552.2899	CPD6669	17.174227	1392.4306
CPD2661	10.182337	957.24469	CPD42399	13.4254281	168.114214	CPD6671	17.195001	644.28411
CPD2664	10.186065	949.75493	CPD42401	13.4604992	323.20905	CPD6675	17.119657	315.21686
CPD26659	33.2027776	273.048375	CPD42410	13.8045994	559.2989	CPD6676	17.081865	342.22271
CPD2666	10.181186	706.2101	CPD4242	25.497464	953.86916	CPD6677	17.50643	479.30047
CPD26664	33.1447098	254.936983	CPD42423	14.3583362	691.482657	CPD6683	17.493678	626.27226
CPD26667	33.2396428	231.172583	CPD42425	14.4549953	503.19052	CPD6686	17.681772	464.25391
CPD2667	10.097507	952.25912	CPD42426	14.4143315	465.166883	CPD669	1.038894	216.0364
CPD2668	10.221508	476.12638	CPD42434	14.9341905	381.22832	CPD6690	17.687816	219.12313
CPD2669	10.188055	947.30354	CPD4244	25.483888	334.25124	CPD6691	17.717658	626.27276
CPD2670	10.193368	492.09958	CPD42443	14.9583492	677.57726	CPD6692	17.816343	181.08855
CPD2671	10.189147	484.11317	CPD4245	25.487162	959.82649	CPD6693	17.83454	157.10973
CPD2672	10.200782	515.13218	CPD4246	25.478268	612.68816	CPD6696	17.738059	618.29993
CPD2675	10.248419	335.10048	CPD4248	25.483231	612.47516	CPD6697	17.922643	295.2508
CPD2676	10.227456	170.1405	CPD42485	16.4383997	345.23172	CPD6700	17.813431	420.22166
CPD2677	10.263934	199.19041	CPD42487	16.5610001	768.502733	CPD6702	17.85533	818.47762
CPD2679	10.351451	736.22051	CPD4249	25.486483	953.60684	CPD6704	17.97042	430.24213
CPD26790	1.4988463	458.107647	CPD42490	16.6994051	388.31572	CPD6705	17.979824	648.40036
CPD26798	1.11225097	590.041558	CPD4250	25.487692	756.51972	CPD6707	17.921489	1040.0752
CPD26799	1.10012314	198.0395	CPD42505	17.2759494	305.236712	CPD6709	17.837855	157.11057
CPD2680	10.170506	630.26711	CPD42519	17.6042002	766.28558	CPD671	1.02984	631.7777
CPD26815	1.10985484	271.107357	CPD4252	25.478759	997.63189	CPD6712	18.009894	430.24365
CPD2683	10.312425	157.1821	CPD42528	17.8347536	279.255582	CPD6717	18.203775	234.16296
CPD26830	1.17768782	220.058021	CPD42529	17.7738027	371.246517	CPD6720	18.281733	246.218
CPD26832	1.01946779	265.087038	CPD4253	25.496442	958.56337	CPD6726	18.423142	669.41938
CPD2684	10.328633	499.11912	CPD42530	17.8545013	446.24215	CPD6740	18.773136	285.30191
CPD2685	10.3291	507.10592	CPD42531	17.8668	403.30676	CPD6743	18.943008	297.26578
CPD26855	1.2226372	343.114525	CPD42537	17.957857	472.355114	CPD6745	18.891328	716.37691
CPD26856	2.17307089	274.064336	CPD42539	17.8652615	371.24668	CPD6746	18.884347	629.46946
CPD2686	10.328824	977.31467	CPD4254	25.482608	594.46437	CPD675	1.026351	281.8974
CPD2687	10.344005	676.20125	CPD42540	17.9661338	279.25546	CPD6750	18.910556	334.21428
CPD26872	1.55820611	230.088356	CPD4255	25.503755	974.53536	CPD6751	19.00331	382.25238
CPD26874	2.04472499	155.05016	CPD42561	18.7136	393.32388	CPD6759	19.137983	502.34566
CPD2688	10.350413	477.09929	CPD42565	19.0145712	438.350043	CPD676	1.0261	601.7645
CPD2689	10.36469	982.27014	CPD4257	25.532699	1016.5211	CPD6764	19.294033	832.47177
CPD26904	6.37510344	348.081631	CPD42574	19.2210885	731.32272	CPD6766	18.954836	160.08881
CPD26906	5.89069092	345.14211	CPD4258	25.48393	953.99157	CPD6767	19.236353	182.16555
CPD2691	10.062448	514.16853	CPD42590	19.3384003	431.30064	CPD6773	19.380655	409.33688
CPD2692	10.437789	736.21971	CPD42596	19.3939999	730.31954	CPD6774	19.359853	390.25931
CPD26926	7.23460792	342.119844	CPD4260	25.580635	1026.5486	CPD6776	19.455037	454.22251
CPD2693	10.389776	706.20964	CPD42604	19.6484	510.33214	CPD6777	19.447491	887.52005
CPD26933	8.22857052	154.097936	CPD4261	25.577504	606.28407	CPD6778	19.449973	892.4756
CPD2694	10.453378	492.09983	CPD42614	19.7745792	722.27066	CPD6782	19.523453	640.28534
CPD2695	10.478529	947.30307	CPD42619	19.9003339	365.240867	CPD6783	19.56075	730.39432
CPD2696	10.389333	952.2599	CPD4262	25.613568	498.3855	CPD6787	19.621016	550.34789
CPD2697	10.478849	484.11414	CPD4263	25.513384	960.10578	CPD679	1.028278	471.8116
CPD26975	10.8180484	402.142088	CPD4264	25.620813	790.52137	CPD6793	19.845326	408.26554
CPD26980	11.8588019	708.2633	CPD4265	25.527925	959.82845	CPD6794	19.882194	734.42151
CPD26981	12.1317752	599.286822	CPD42660	20.5688776	154.099371	CPD6795	19.860082	434.25013
CPD26987	13.2017651	569.235856	CPD4267	25.570624	1016.5212	CPD6796	19.895045	744.40845
CPD26997	14.9913059	677.054587	CPD4268	25.696673	283.28874	CPD6798	20.088861	734.42254
CPD2700	10.581578	952.25852	CPD42691	21.2448013	698.294871	CPD680	1.031866	365.8598
CPD2701	10.593883	461.15441	CPD4271	25.727695	758.43999	CPD6800	20.003002	368.11767
CPD27012	16.018333	720.249478	CPD42715	21.68	678.26558	CPD6807	20.091567	467.38414
CPD27014	16.6510703	260.213443	CPD4273	25.723757	742.46464	CPD6810	20.219539	644.39322
CPD2702	10.583068	706.20907	CPD42747	22.3787211	877.67544	CPD6811	20.232027	359.18079
CPD27020	16.6997607	531.339914	CPD4275	25.736571	390.45052	CPD6813	20.381805	406.32782
CPD27021	16.6550698	242.202589	CPD42752	22.4515008	714.305767	CPD6814	20.238748	718.37915
CPD2703	10.66749	947.30208	CPD4276	25.733319	737.50618	CPD6815	20.089577	368.11774
CPD2704	10.610457	221.17664	CPD4278	25.739355	390.27753	CPD6816	20.144078	204.18595
CPD27042	18.4508127	319.251148	CPD42780	22.6435184	720.279917	CPD6818	20.180198	690.39872
CPD27047	19.512766	302.135192	CPD4279	25.728997	695.53934	CPD682	1.039833	493.7454
CPD2707	10.694411	246.12637	CPD4281	25.745596	560.37155	CPD6821	20.231467	814.45809
CPD2708	10.771832	977.31333	CPD42812	23.18154	764.66062	CPD6822	20.060785	242.20141
CPD27086	21.7611782	738.286813	CPD4283	25.749577	148.01597	CPD6823	20.355406	467.38375
CPD2709	10.7678	736.21999	CPD4284	25.757386	428.23381	CPD6828	20.357915	430.2441
CPD27094	21.9744978	668.284741	CPD4285	25.767672	435.33913	CPD683	1.028556	355.8407

CPD2710	10.775923	507.1051	CPD4287	25.752641	480.2536	CPD6832	20.347887	1006.5439
CPD27102	22.0642938	664.250094	CPD4288	25.907746	432.37599	CPD6835	20.239084	718.37842
CPD2711	10.756079	982.27036	CPD4290	25.826574	782.56554	CPD6837	20.302904	697.43933
CPD27113	22.5226839	668.284037	CPD42914	28.6468335	593.46065	CPD6838	20.33038	518.35884
CPD27116	22.9541902	704.223863	CPD4292	25.638102	769.56399	CPD6839	20.278066	511.40745
CPD27126	23.0517158	688.249831	CPD42920	29.3296462	708.4936	CPD6841	20.361964	640.28942
CPD2713	10.7481	235.19236	CPD4293	25.775969	463.35937	CPD6842	20.339298	359.1828
CPD2714	10.880459	484.11343	CPD42937	33.1092329	221.181025	CPD685	1.0425	573.8403
CPD27145	23.536648	701.231711	CPD42938	33.141154	228.929985	CPD6851	20.889351	728.41746
CPD2715	10.841587	706.2095	CPD4294	25.801307	636.9225	CPD6853	20.519962	402.22707
CPD2716	10.874104	952.25979	CPD4297	25.799765	774.52677	CPD6854	20.545046	371.33998
CPD27163	24.3828646	625.477537	CPD4300	25.876842	370.30799	CPD6855	20.544971	544.84907
CPD2717	10.877613	492.10069	CPD4301	25.878573	392.46302	CPD6856	20.393413	582.4041
CPD27170	24.7420475	652.290519	CPD4302	25.8121	639.90364	CPD6857	20.531332	677.43752
CPD2718	10.856337	947.30392	CPD43020	1.05281244	218.019781	CPD686	1.029148	203.9071
CPD27182	25.3940194	590.478772	CPD4306	25.922234	454.36095	CPD6862	20.642231	522.83647
CPD27183	25.5064463	606.588843	CPD43061	1.50767564	359.138567	CPD6864	20.482591	340.25826
CPD2719	10.923102	630.26775	CPD4307	25.932812	494.26743	CPD6869	20.974809	988.53646
CPD27199	26.4898816	753.534567	CPD43084	1.71649938	92.0135833	CPD6873	20.982636	392.24016
CPD2720	10.955356	461.15502	CPD43088	1.86760405	169.056754	CPD6875	20.812568	476.32613
CPD27206	27.7328753	336.098435	CPD4310	25.919195	553.30613	CPD6878	20.841723	272.21107
CPD27209	27.7274985	412.263072	CPD43106	5.71099981	383.16108	CPD6880	20.651061	360.24453
CPD27210	27.7186494	366.104493	CPD43107	5.72660002	286.15224	CPD6881	20.926411	614.40291
CPD27212	27.7172785	54.0471286	CPD43118	6.67212494	469.162087	CPD6891	21.108712	817.47131
CPD2722	11.037402	736.21937	CPD4312	26.019799	753.53558	CPD6896	21.069348	957.55887
CPD2725	11.181608	409.19417	CPD43126	7.31769358	370.159086	CPD6898	20.993461	716.41607
CPD2727	11.178449	414.14996	CPD4313	26.01685	821.52372	CPD6901	21.061615	985.59142
CPD2728	11.197225	215.05982	CPD4314	26.02405	731.5535	CPD6903	21.130256	340.14954
CPD2729	11.201616	230.04289	CPD43141	7.8279998	941.254688	CPD6905	21.115762	196.11393
CPD2731	11.228078	199.15984	CPD43144	7.84214176	141.080043	CPD6906	21.093178	354.27837
CPD2732	11.243097	208.14604	CPD4315	26.036739	569.4913	CPD6907	20.97947	376.2633
CPD2735	11.440371	258.2287	CPD4316	26.107082	826.44201	CPD6908	21.037429	336.26759
CPD2736	11.530633	406.25703	CPD4317	26.079294	309.30298	CPD6909	21.137615	808.51766
CPD2738	11.544404	187.19235	CPD4318	26.120421	836.46778	CPD6913	21.245594	764.49013
CPD27388	1.32117432	367.044195	CPD43183	8.65079968	946.71878	CPD6916	21.291449	762.45744
CPD2739	11.592897	480.12836	CPD43187	8.82774367	339.132043	CPD6919	21.419087	376.31565
CPD2740	11.70099	230.04379	CPD4319	26.091277	592.58631	CPD6922	21.409034	335.31699
CPD2741	11.602895	475.17171	CPD4320	26.22376	770.82764	CPD6924	21.433889	171.10864
CPD27417	1.34350058	389.8831	CPD43207	9.12099931	996.21652	CPD6927	21.463407	341.18092
CPD2742	11.595427	553.09126	CPD4321	26.224774	765.76504	CPD6929	21.459929	635.39983
CPD2743	11.663201	235.19269	CPD43211	9.12962497	490.12545	CPD6934	21.447918	771.14442
CPD27439	2.0983686	341.113364	CPD43212	8.93302143	126.031412	CPD6935	21.463466	359.19846
CPD2746	11.744273	395.21489	CPD43215	9.15540087	409.19334	CPD6936	21.448877	164.15093
CPD27460	6.59785304	372.175971	CPD43217	9.18499861	526.20332	CPD6940	21.484381	338.30075
CPD27462	6.57568393	567.132167	CPD43228	9.23339261	1064.28381	CPD6942	21.523736	341.18154
CPD27463	6.40813206	227.0595	CPD4323	26.21202	784.45297	CPD6943	21.569184	648.39173
CPD27471	6.91813574	470.068214	CPD43237	9.66866631	646.240783	CPD6945	21.529724	870.49191
CPD27477	7.81302466	224.068854	CPD4325	26.19452	768.47898	CPD6947	21.582929	969.59918
CPD27478	8.07291318	224.066975	CPD43258	10.1999953	616.70856	CPD6949	21.59728	932.54864
CPD2748	11.897037	220.06728	CPD43260	10.2843605	270.18165	CPD6950	21.488747	614.40441
CPD27485	8.65723575	174.13898	CPD43262	10.2707991	287.0969	CPD6956	21.643177	746.40188
CPD2749	11.928812	276.12204	CPD4327	26.1245	283.28762	CPD6960	21.675054	588.38568
CPD2750	11.917301	419.21514	CPD4330	26.262899	769.3022	CPD6962	21.569011	684.27646
CPD27502	12.7335916	503.679167	CPD43305	12.1651281	616.20776	CPD6963	21.701556	442.3267
CPD27505	13.9392464	660.242626	CPD43336	13.4281981	132.09288	CPD6967	21.666168	654.26941
CPD27508	14.8108228	688.275564	CPD43354	14.3391641	1426.51107	CPD697	1.033057	487.7825
CPD2751	11.988016	245.23493	CPD43372	14.9935985	855.87854	CPD6970	21.795101	398.30099
CPD2752	12.030176	114.10361	CPD4340	26.202003	585.95653	CPD6977	21.856923	908.56951
CPD27532	16.1456768	688.275311	CPD43436	18.2047703	365.40016	CPD6978	21.901256	368.3748
CPD2754	12.021357	190.15622	CPD4345	26.21274	306.21952	CPD6983	21.967109	406.26588
CPD27540	17.4818166	716.305312	CPD4346	26.203358	566.43291	CPD6985	21.90347	744.4018
CPD27544	17.800853	427.167314	CPD43460	19.22	943.361771	CPD6987	21.918289	456.81348
CPD27547	18.1353785	674.224086	CPD4348	26.201567	710.47639	CPD6989	21.933867	451.17479
CPD2755	12.053526	201.20815	CPD4349	26.19756	584.44375	CPD6991	21.967455	926.55936
CPD27564	19.5862377	688.239157	CPD4350	26.209974	334.25082	CPD6992	22.01037	756.44366
CPD27569	19.9987274	449.233655	CPD4351	26.20682	728.48532	CPD6996	22.052351	441.79304
CPD27587	20.9228106	612.260007	CPD4352	26.215157	746.49808	CPD700	1.033464	325.8202

CPD2759	12.261363	261.2297	CPD4353	26.215262	791.55434	CPD7002	21.920283	400.27992
CPD27593	21.5326593	370.26665	CPD4354	26.23179	805.54468	CPD7003	22.131997	410.36019
CPD2760	12.364958	559.22623	CPD43547	21.8961134	744.301114	CPD7020	22.427622	598.70032
CPD27607	21.9849438	584.227706	CPD43548	21.8731667	650.26475	CPD7023	22.324631	419.32146
CPD2761	12.272688	276.13381	CPD4355	26.199824	763.52341	CPD7028	22.101871	410.16678
CPD27612	22.0221452	868.531529	CPD4356	26.23309	867.56654	CPD703	1.032364	439.804
CPD27616	22.114537	407.324893	CPD43570	22.1004535	905.628044	CPD7030	22.330679	624.90429
CPD27619	22.8115855	730.320033	CPD4358	26.250944	764.86227	CPD7034	22.424256	662.27585
CPD2762	12.259572	542.19977	CPD4359	26.244363	769.06485	CPD7044	22.433232	706.43502
CPD27626	22.9718752	582.251494	CPD4361	26.250886	768.47895	CPD7050	22.787794	726.46932
CPD2763	12.324076	294.14783	CPD4362	26.302846	768.98585	CPD7051	22.446436	324.23294
CPD2766	12.346255	305.23583	CPD43621	22.9291974	1043.57058	CPD7052	22.370866	784.47261
CPD2767	12.40626	434.15986	CPD4363	26.259036	764.18126	CPD7056	22.538356	578.9009
CPD2768	12.415217	291.06623	CPD43631	23.1077095	281.272175	CPD7061	22.339441	638.611
CPD27681	25.1569363	644.239495	CPD4366	26.296151	764.28455	CPD7068	22.697476	732.44498
CPD27684	25.3330876	410.30327	CPD4369	26.300282	784.45267	CPD7069	22.721676	598.41168
CPD2769	12.414306	186.11766	CPD43762	32.3197803	882.496064	CPD707	1.040629	309.8325
CPD27691	25.2345474	778.355279	CPD4377	26.211554	585.74322	CPD7071	22.719302	293.26675
CPD2770	12.371647	564.1804	CPD43788	1.15909837	166.112067	CPD7072	22.773941	1007.691
CPD2771	12.419361	289.06703	CPD4381	26.418447	731.55255	CPD7076	22.779322	512.36481
CPD27716	27.7296179	399.163121	CPD4382	26.388725	826.44513	CPD7077	22.857387	493.34092
CPD2772	12.512232	570.2471	CPD4384	26.336593	836.46648	CPD7080	22.856728	490.34959
CPD2775	12.552897	486.20957	CPD4385	26.498617	849.24303	CPD7082	22.934999	404.29548
CPD2776	12.510347	189.11856	CPD4386	26.388019	388.35226	CPD7083	22.94318	470.81308
CPD27774	33.2764111	297.88061	CPD4387	26.419043	904.45299	CPD7089	22.93258	924.59687
CPD2779	12.650612	204.13651	CPD4388	26.44838	853.45397	CPD7090	22.889497	626.27344
CPD2780	12.760872	227.22421	CPD43881	1.58350305	277.140675	CPD7091	23.021982	429.38066
CPD2781	12.871164	160.04171	CPD43896	2.98291808	464.051708	CPD7094	22.96529	1019.6929
CPD27813	1.05041355	234.040362	CPD4390	26.459204	972.44019	CPD7095	22.998818	668.28373
CPD2782	12.850828	206.0932	CPD4391	26.372901	768.47868	CPD7096	23.020191	360.28943
CPD27837	1.01004993	403.03814	CPD43921	5.98579964	129.15064	CPD7097	23.003053	368.2853
CPD27839	1.00299127	231.036862	CPD4393	26.618388	785.61465	CPD7101	23.030234	463.82128
CPD27840	1.00373298	197.116507	CPD43942	6.93308967	791.715792	CPD7102	22.940615	626.27517
CPD27842	1.02300984	349.051908	CPD4395	26.490215	753.53498	CPD711	1.034385	669.7483
CPD27845	1.01663506	197.241275	CPD4396	26.490126	829.64109	CPD7110	23.151239	562.97817
CPD27848	1.03883395	377.995188	CPD43966	7.47272478	464.095714	CPD7124	23.177313	281.94403
CPD2785	13.243436	465.18347	CPD4397	26.562865	344.32596	CPD7125	23.172789	439.22894
CPD27852	1.01988874	248.038767	CPD4399	26.706749	741.58655	CPD7131	22.997621	421.341
CPD2788	13.129231	250.12197	CPD43996	8.21257243	340.14738	CPD7133	23.181403	360.29137
CPD27883	1.04835003	300.21645	CPD4400	26.575932	774.52621	CPD7135	23.168308	377.31547
CPD2789	13.359831	465.18196	CPD44004	8.61623204	456.16836	CPD7136	23.156508	382.27137
CPD27898	1.06033353	547.994747	CPD4401	26.634069	722.13458	CPD7139	23.723114	883.55252
CPD2790	13.31332	464.68064	CPD4402	26.641445	743.22724	CPD7146	23.286609	764.47473
CPD2791	13.349998	242.2366	CPD44028	9.17558375	1207.30013	CPD7148	23.327001	592.26987
CPD27913	1.06130071	341.09328	CPD4404	26.650607	706.16455	CPD7149	23.375318	267.24655
CPD27917	1.06922226	423.187222	CPD4405	26.727896	665.94345	CPD715	1.034249	715.742
CPD27920	1.07954998	226.072175	CPD4407	26.437769	836.46785	CPD7153	23.188045	281.98991
CPD27921	1.07863919	348.100421	CPD44076	9.95639946	372.13978	CPD7154	23.38455	421.36671
CPD27923	1.08214996	291.21575	CPD4408	26.673938	701.2055	CPD7158	23.406898	729.54871
CPD27925	1.0856	299.0886	CPD4410	26.717389	663.96594	CPD7161	23.492398	609.90337
CPD27929	1.12900757	521.0855	CPD4411	26.754752	550.41584	CPD7163	23.490106	660.44355
CPD2793	13.3715	286.26131	CPD4413	26.769014	567.42012	CPD7168	23.536848	564.54708
CPD27932	1.1012948	240.147503	CPD4415	26.853832	756.48147	CPD7175	23.69789	511.26558
CPD27934	1.09475454	197.116744	CPD4416	26.903756	382.35333	CPD7176	23.544002	1077.7339
CPD27935	1.09247351	153.126682	CPD4420	26.905757	950.52845	CPD7179	23.641671	563.89767
CPD27936	1.10300005	167.105187	CPD44207	16.4718302	284.116433	CPD718	1.0371	423.8117
CPD27938	1.09831252	497.122319	CPD4421	26.928324	934.56152	CPD7181	23.681125	353.32783
CPD27943	1.09697061	408.930955	CPD44219	16.8042	802.2841	CPD7189	23.712213	931.91271
CPD2795	13.4412	180.11506	CPD4422	26.914393	929.6055	CPD7190	23.55375	567.63811
CPD27950	1.11794107	751.046359	CPD4423	27.005693	638.9376	CPD7193	23.701183	1015.5043
CPD27952	1.12711874	325.157538	CPD4424	26.993334	908.54776	CPD7197	23.754999	584.26665
CPD27955	1.1213333	825.08028	CPD4426	27.051423	344.32717	CPD720	1.0428	340.923
CPD2796	13.513223	290.18821	CPD4428	27.097518	616.92477	CPD7201	23.778685	528.36417
CPD27962	1.12736365	642.126109	CPD4429	26.87084	855.51595	CPD7202	23.77977	292.24147
CPD27963	1.1273	519.21597	CPD4430	27.226789	795.57929	CPD7204	23.827861	894.60273
CPD27965	1.13774808	305.043563	CPD4431	27.216645	594.91088	CPD7211	23.937608	608.4408
CPD27969	1.11942099	474.039032	CPD4432	26.839807	860.47185	CPD7215	24.066204	295.28639

CPD27973	1.1589988	213.112906	CPD4433	27.104557	882.45449	CPD7218	24.045908	417.28029
CPD2798	13.631947	316.19008	CPD4434	27.205793	800.53997	CPD7221	24.093138	407.30813
CPD27991	1.14493741	225.075625	CPD4435	27.246117	898.42863	CPD7222	24.077551	444.35949
CPD27993	1.15359992	230.089904	CPD4437	27.35755	782.49489	CPD7224	24.077364	423.27599
CPD2800	13.725541	349.22998	CPD4438	27.340343	598.4564	CPD7226	24.022276	480.80477
CPD28002	1.14691671	490.037342	CPD44387	20.9177435	354.27737	CPD7227	24.099679	604.45544
CPD28003	1.15607693	267.0973	CPD4439	27.465704	960.96256	CPD7230	24.206287	576.90381
CPD28005	1.21968399	505.197169	CPD4440	27.460601	976.55094	CPD7232	24.338412	311.3542
CPD28008	1.12550002	668.108149	CPD4441	27.458501	955.62137	CPD7238	24.317609	648.11701
CPD2801	13.66103	290.18822	CPD4442	27.459496	1028.5652	CPD7240	24.355501	516.27231
CPD28015	1.38395914	151.049481	CPD4443	27.462343	960.57741	CPD7242	24.349808	738.58715
CPD28016	1.67037433	422.072031	CPD4444	27.457694	614.48812	CPD7243	24.355086	269.27161
CPD28018	1.25241812	412.180794	CPD4447	27.620118	723.56886	CPD7246	24.445317	939.592
CPD2802	13.736204	342.28754	CPD4448	27.590354	556.08691	CPD7247	24.481235	225.17216
CPD28020	1.30117884	258.091967	CPD4449	27.71645	686.50776	CPD7257	24.675976	837.62289
CPD28033	1.18444256	118.027972	CPD44491	22.2217212	826.614683	CPD7259	24.91926	493.39884
CPD28038	1.16560004	145.110305	CPD4451	27.724251	629.49732	CPD7262	24.640326	600.41718
CPD28039	1.18779014	244.07872	CPD4452	27.689641	395.32027	CPD7265	24.765058	581.45273
CPD2804	13.769433	227.19517	CPD4453	27.910744	922.56049	CPD7267	24.874862	828.4822
CPD28043	1.19146929	466.075809	CPD4454	27.718309	634.45502	CPD7268	24.746937	542.3817
CPD28046	1.20924647	255.095663	CPD4456	27.73615	650.43103	CPD7269	24.897173	608.40461
CPD28048	1.18449946	159.12606	CPD4457	27.840374	858.59264	CPD7278	25.114	770.44578
CPD28049	1.18469179	211.092538	CPD4459	27.753247	724.49038	CPD7280	25.10648	607.09857
CPD28052	1.36235982	259.123727	CPD4462	27.734442	82.077967	CPD7284	25.131033	606.97354
CPD28055	1.20651139	397.989211	CPD4463	27.784949	744.48078	CPD7285	25.149521	583.41821
CPD28056	1.17590602	228.050195	CPD4464	27.794289	739.51154	CPD7286	24.906207	812.50528
CPD28057	1.21695016	165.193055	CPD44666	24.1946451	622.73826	CPD7289	25.098328	607.88697
CPD28058	1.28201334	597.983043	CPD4468	27.898956	694.96467	CPD7295	25.209077	652.44558
CPD2806	13.816665	268.25021	CPD44692	24.9891997	1047.6009	CPD7300	25.519925	960.93721
CPD28061	1.22264995	173.141885	CPD4471	27.866399	567.42198	CPD7307	25.471173	995.62952
CPD28066	1.06255752	414.986136	CPD4473	27.952792	694.96444	CPD7310	25.475962	612.78472
CPD2807	13.857291	379.25465	CPD4475	28.159707	1054.5797	CPD7321	25.731166	802.77901
CPD28074	1.254928	489.014278	CPD4476	28.165139	986.59226	CPD7323	25.729327	519.42486
CPD28076	1.49717496	103.062807	CPD44769	30.7148013	609.176056	CPD7327	25.742468	112.12385
CPD28077	1.40472632	292.01229	CPD4477	28.156938	1002.5665	CPD7328	25.718327	637.92816
CPD28083	1.53685169	339.058667	CPD4479	28.139855	939.53605	CPD7331	25.919894	494.27229
CPD28085	1.56728121	195.054347	CPD4480	28.163588	981.63689	CPD7332	25.918836	516.2423
CPD28089	1.56023529	275.294947	CPD4482	28.378042	772.74168	CPD7339	26.067628	946.56315
CPD2809	13.91581	660.24254	CPD4484	28.259003	551.18975	CPD7345	26.260063	787.45954
CPD28091	1.5623	357.10708	CPD4485	28.377893	906.47165	CPD7347	26.250937	867.56663
CPD28094	1.56277778	153.078833	CPD4486	28.37763	828.45344	CPD7353	26.258601	585.74279
CPD28097	1.56706668	159.126087	CPD44898	1.34591347	464.053219	CPD7355	26.219359	584.9632
CPD2810	14.046267	273.26657	CPD4490	28.321268	771.00949	CPD7359	26.230095	770.722
CPD28101	1.56213334	306.231013	CPD44905	1.20721881	223.070067	CPD7370	26.281819	764.02868
CPD28103	1.60061412	361.134777	CPD44907	2.0751771	412.17805	CPD7374	26.386436	410.33473
CPD28107	1.56766678	289.074456	CPD4491	28.365209	771.83855	CPD7377	26.331389	388.34956
CPD2811	14.14844	229.23982	CPD4492	28.296857	334.25136	CPD738	1.027417	287.9941
CPD28112	1.61500007	120.057353	CPD4493	28.330287	765.88312	CPD7388	26.691905	972.57576
CPD28113	1.61735711	283.241	CPD4494	28.391713	974.45498	CPD739	1.054696	342.9236
CPD28114	1.62250006	316.177017	CPD44946	5.29849972	190.026758	CPD7391	26.7327	309.30187
CPD28117	1.42947565	318.042929	CPD4495	28.355791	765.7747	CPD7396	26.771058	567.62691
CPD28118	1.68807683	272.001885	CPD44967	6.43010519	206.062157	CPD7398	26.763266	584.42507
CPD28119	1.69427463	179.007728	CPD4497	28.344393	771.08384	CPD7403	26.754417	668.92559
CPD2812	14.238984	379.25659	CPD4500	28.359134	807.55879	CPD7408	26.923523	741.59192
CPD28120	1.68540192	251.983547	CPD45004	7.49740163	479.161739	CPD7412	27.061007	770.49256
CPD28123	1.69938954	210.961078	CPD4503	28.32683	765.53751	CPD7417	27.32744	572.89765
CPD28133	1.82328461	185.991464	CPD4504	28.365775	586.45925	CPD7420	27.472259	1018.5344
CPD28139	1.96880003	276.240605	CPD45047	8.2151993	261.09922	CPD7428	27.6393	535.15765
CPD2814	14.286065	289.26121	CPD4505	28.318139	770.49459	CPD7430	27.732439	96.093067
CPD28140	1.95821061	159.125237	CPD4507	28.344945	786.46783	CPD7432	27.799454	738.46797
CPD28142	1.9811	173.141385	CPD4509	28.369156	730.50927	CPD744	1.040367	273.8661
CPD28146	2.05306332	401.19061	CPD4510	28.357716	586.66974	CPD7443	28.370822	771.73474
CPD28147	2.02484994	228.049035	CPD45101	9.5735565	1010.26182	CPD7449	28.421172	869.5832
CPD2815	14.406251	287.28077	CPD4511	28.366005	855.46972	CPD7451	28.350905	770.83739
CPD28152	2.23289915	118.0264	CPD4512	28.376029	869.58268	CPD7453	28.395011	765.87156
CPD28157	2.3362792	93.0381727	CPD4513	28.377107	838.48201	CPD7455	28.459535	786.70863
CPD28158	2.33103876	209.06549	CPD4516	28.414479	765.77427	CPD7456	28.464063	727.57252

CPD28167	2.49230219	337.075038	CPD4519	28.433569	771.2423	CPD7460	28.646665	614.40163
CPD2817	14.545756	158.13012	CPD4523	28.477113	826.55405	CPD7462	28.916478	880.4744
CPD28170	2.55485871	244.048629	CPD4524	28.379756	974.4565	CPD7471	29.030111	797.75472
CPD28171	2.60024959	260.083505	CPD4528	28.392201	830.45548	CPD7472	28.994397	797.10444
CPD28177	2.82699542	184.046135	CPD45299	19.5220821	407.339233	CPD748	1.042544	189.9044
CPD28178	2.77727592	216.078885	CPD4532	28.503841	826.55541	CPD7485	29.10004	556.43024
CPD2818	14.535744	294.10675	CPD4533	28.67009	666.41408	CPD7487	29.104307	551.47346
CPD28180	2.88663171	323.060137	CPD4535	28.815192	444.35397	CPD7488	29.10685	864.49689
CPD28184	3.12594687	380.081284	CPD4536	28.932452	854.47122	CPD7490	28.957311	792.90394
CPD28187	3.21545006	99.10394	CPD4537	28.991593	881.48423	CPD7491	29.00194	1000.4723
CPD28190	3.48109921	247.023984	CPD4543	28.982214	797.02587	CPD7493	29.053372	854.47213
CPD28197	3.57019747	231.049655	CPD4545	28.955664	812.72659	CPD7495	29.127574	880.47577
CPD28198	3.56567843	191.057414	CPD4546	28.967185	792.15171	CPD7499	29.367927	463.41495
CPD28199	3.5624316	270.120384	CPD4548	28.959168	334.25102	CPD7503	29.516832	753.52126
CPD2820	14.605546	244.07743	CPD4549	29.007585	812.85502	CPD7504	29.753406	758.49997
CPD28205	3.77397975	333.1431	CPD4553	28.983541	792.79744	CPD7508	30.054932	375.36855
CPD28208	3.91238387	190.075946	CPD4554	28.956249	594.46402	CPD7509	29.958751	553.49679
CPD28221	4.87438739	178.022125	CPD4556	29.059949	797.75387	CPD7511	29.993076	380.32542
CPD28224	5.20212837	385.193564	CPD4557	28.958592	756.51932	CPD7513	29.989945	358.3426
CPD2823	14.719863	471.67075	CPD45600	33.3576094	233.994142	CPD7516	30.337654	994.53654
CPD28230	5.17104937	134.073665	CPD4561	28.960916	774.53152	CPD7519	30.388097	775.22571
CPD28236	5.30364209	473.165771	CPD4563	28.957037	612.68853	CPD752	1.0431	295.8019
CPD28238	5.37577913	471.1944	CPD4564	28.962543	791.55476	CPD7520	30.334629	937.95821
CPD2824	14.715828	472.17349	CPD4565	28.962931	612.47531	CPD7522	30.659322	609.17637
CPD28241	6.00672758	96.0929625	CPD4566	28.968984	833.57745	CPD7523	30.663896	616.49471
CPD28243	5.50543598	221.107638	CPD4567	28.972159	796.51065	CPD7524	30.685426	978.56807
CPD28250	5.73959599	374.21458	CPD4568	28.965447	895.59914	CPD7526	30.837184	534.67299
CPD28251	5.7407625	405.126803	CPD4569	28.99179	812.48316	CPD7527	30.82046	571.45492
CPD28253	5.74492606	410.081593	CPD45726	1.50070529	149.063433	CPD7528	30.851105	741.53969
CPD2826	14.710266	287.28138	CPD4576	28.96245	613.69326	CPD7530	30.831735	534.59057
CPD28263	5.84078272	162.031139	CPD45777	1.70952653	285.061848	CPD7532	30.850402	682.4922
CPD28264	5.89792333	341.168289	CPD4579	28.914731	864.49654	CPD7536	30.88258	630.10673
CPD28265	5.83566686	355.16475	CPD4580	29.032024	881.48461	CPD7537	31.498372	772.8485
CPD28267	5.8356499	298.06888	CPD4584	29.018005	1000.4706	CPD7541	31.608091	831.47517
CPD2827	14.785908	243.25665	CPD45841	6.06100025	203.07486	CPD7548	31.983375	337.33241
CPD28273	5.87801036	342.09644	CPD45858	6.54819742	423.9724	CPD7549	32.130582	604.49899
CPD28275	5.86859975	366.07114	CPD45905	6.97589252	326.114036	CPD7558	32.342427	798.86776
CPD28280	5.47575899	234.134286	CPD4591	29.057739	932.48401	CPD7565	32.608155	468.35173
CPD28283	5.94306532	179.099273	CPD4594	29.236356	512.40569	CPD757	1.034417	685.7293
CPD28284	5.97492852	261.120686	CPD4596	29.467495	339.3848	CPD7571	33.046297	312.89891
CPD28287	6.05009615	316.141889	CPD4597	29.479023	339.38477	CPD7573	33.059755	237.90056
CPD28288	6.00823305	353.182882	CPD45974	8.5775278	478.099505	CPD7577	33.068875	177.94446
CPD2829	14.93036	364.19241	CPD4598	29.380077	468.3778	CPD7579	33.07345	89.999958
CPD28292	6.06080638	382.13746	CPD4600	29.405235	724.47069	CPD7581	33.082076	170.95528
CPD28296	6.09655191	335.065211	CPD4601	29.418469	650.45102	CPD7582	33.072951	214.97188
CPD28298	6.11433737	312.084635	CPD4602	29.414436	708.49324	CPD7585	33.052466	179.95233
CPD2830	14.741668	199.22801	CPD4603	29.470708	737.54	CPD7587	33.093799	183.97941
CPD28300	6.13883122	503.25755	CPD4605	29.680456	424.35255	CPD7589	33.093334	249.19227
CPD28302	6.15008065	306.168589	CPD4606	29.672772	419.39487	CPD7591	33.102044	196.16617
CPD28303	6.16293842	412.053741	CPD4607	29.96997	834.52495	CPD7592	33.104097	302.21283
CPD28304	6.17481806	514.124387	CPD4608	30.250509	662.44604	CPD7593	33.102546	251.19661
CPD2831	14.913764	232.18106	CPD4609	29.964429	805.53264	CPD7594	33.11676	233.1817
CPD28312	6.3368729	468.104394	CPD4610	29.971405	715.55733	CPD7599	33.111909	223.9843
CPD28313	6.33131548	614.212826	CPD4615	30.194442	311.31879	CPD7603	33.112471	230.16202
CPD28314	6.35587243	452.132278	CPD4616	30.275674	962.59272	CPD7605	33.11456	224.98309
CPD28315	6.34715297	268.094946	CPD4617	30.335139	572.46853	CPD7607	33.112	349.88593
CPD28318	6.40166637	235.04682	CPD4618	30.34192	936.83971	CPD761	1.048882	332.8909
CPD2832	14.908441	570.24521	CPD4621	30.338585	952.551	CPD7611	33.120054	207.93933
CPD28320	6.40720631	219.06845	CPD4622	30.340491	1004.5642	CPD7615	33.125786	234.90986
CPD28321	6.39906379	447.173327	CPD4623	30.34034	590.48959	CPD7620	33.126744	233.87969
CPD28322	6.43079977	366.15869	CPD4624	30.338263	936.57807	CPD7621	33.1275	389.87635
CPD28323	6.46086515	428.129893	CPD4625	30.336076	931.62173	CPD7628	33.129222	585.79976
CPD28325	6.46383889	309.140505	CPD4630	30.632979	630.10528	CPD7629	33.125941	276.9104
CPD28326	6.47483364	352.125378	CPD4632	30.633748	957.63571	CPD7632	33.127432	402.87569
CPD28327	6.45987822	397.1581	CPD4634	30.669871	962.59268	CPD7634	33.131263	365.88042
CPD28329	6.53320858	557.192242	CPD4638	30.745186	668.4995	CPD7635	33.133737	496.83574
CPD2833	14.891919	468.27189	CPD4640	30.8257	557.46222	CPD7636	33.137435	428.85676

CPD28330	6.54739815	452.19965	CPD4642	30.829984	529.43574	CPD7638	33.123228	363.85508
CPD28333	6.57688735	574.555778	CPD4644	30.835838	550.36277	CPD7642	33.130455	593.78924
CPD28336	6.57739845	794.024385	CPD4645	30.837836	534.38951	CPD7645	33.129059	579.80409
CPD28337	6.57691085	767.071655	CPD4647	30.895781	337.33449	CPD7648	33.12244	413.87332
CPD28339	6.57547394	1164.07988	CPD4649	30.852258	529.43586	CPD765	1.037692	409.7834
CPD2834	14.906903	262.11585	CPD4651	31.27631	311.31877	CPD7650	33.124072	249.93051
CPD28340	6.5780005	631.9937	CPD4653	31.349729	983.65194	CPD7654	33.137	436.85383
CPD28341	6.57820005	352.042675	CPD4654	31.338044	988.60804	CPD7655	33.134897	401.88765
CPD28342	6.57979873	574.053785	CPD4656	31.500416	767.79334	CPD7657	33.141453	140.10667
CPD28343	6.57713113	944.61092	CPD46567	1.10852374	158.070043	CPD7659	33.144444	241.94541
CPD28344	6.57822092	759.08155	CPD4659	31.484642	772.7469	CPD766	1.0438	248.9273
CPD28345	6.58461646	271.018077	CPD4660	31.504539	570.4649	CPD7660	33.134412	568.81078
CPD28346	6.58360043	431.106107	CPD4663	31.487791	588.47296	CPD7664	33.132286	360.37218
CPD28347	6.57915546	616.021258	CPD4664	31.480358	767.55263	CPD767	1.041167	275.8649
CPD28348	6.58209945	370.05316	CPD4665	31.480872	772.5095	CPD7673	33.130394	199.9332
CPD2835	14.89878	486.28243	CPD46655	8.54236445	966.248119	CPD7674	33.132889	623.77193
CPD28351	6.56247408	180.041905	CPD4667	31.484226	840.49701	CPD7675	33.131151	366.8761
CPD28352	6.58379905	464.997485	CPD4668	31.479656	788.48375	CPD7677	33.137	494.83569
CPD28353	6.5910838	302.96722	CPD4669	31.500282	830.47059	CPD768	1.043783	121.9171
CPD28354	6.59242418	286.9947	CPD46715	16.0838367	1015.5261	CPD7681	33.135379	438.8565
CPD28355	6.60589326	407.172171	CPD46719	16.1912786	499.271411	CPD7682	33.140294	374.36913
CPD2836	14.892839	270.10219	CPD4672	31.556202	772.74777	CPD7684	33.148208	255.072
CPD28369	6.67625173	342.0961	CPD4674	31.705681	741.53822	CPD7686	33.134123	73.101875
CPD2837	14.914951	324.22941	CPD4677	31.709537	746.49658	CPD7687	33.144185	214.04071
CPD28373	6.7033684	326.100563	CPD46788	19.7612386	457.339511	CPD7691	33.1411	263.92793
CPD28375	6.83552303	410.155011	CPD4679	31.71192	562.45702	CPD7693	33.162175	95.966096
CPD28376	6.7355251	674.106918	CPD4687	32.053558	622.39975	CPD7694	33.152851	207.03124
CPD28379	6.75433078	146.142867	CPD4689	32.035672	674.4327	CPD7696	33.155116	49.988708
CPD2838	14.909542	306.21213	CPD4691	32.061501	606.42549	CPD7697	33.150903	169.99829
CPD28380	6.76357531	282.07349	CPD4692	32.065835	601.46902	CPD7698	33.150668	266.02238
CPD28381	6.74136639	706.044663	CPD4693	32.117558	945.63644	CPD7703	33.162207	264.92163
CPD28382	6.75654797	230.9823	CPD4694	32.114526	950.59266	CPD7704	33.145822	398.86384
CPD28387	6.78628839	557.132353	CPD4700	32.340514	793.56891	CPD7707	33.160289	130.0103
CPD28388	6.76206586	343.1268	CPD4701	32.334491	798.52527	CPD7710	33.162348	416.84975
CPD2839	14.918529	600.2953	CPD4702	32.337202	614.48967	CPD7711	33.174514	188.04489
CPD28392	6.83694823	279.147235	CPD4703	32.336389	814.49872	CPD7714	33.216198	139.9549
CPD28394	6.85877076	306.1685	CPD4705	32.334676	866.51446	CPD7717	33.170634	239.21078
CPD28395	6.85491029	350.150327	CPD4706	32.33523	856.48442	CPD7718	33.178001	112.09967
CPD28396	6.8378329	179.047692	CPD4708	32.374959	799.86517	CPD7724	33.195438	232.06399
CPD28397	6.86289501	432.10501	CPD4709	32.311085	798.76575	CPD7732	33.128432	194.14696
CPD28398	6.88323982	756.210438	CPD47119	1.0161305	594.919586	CPD7739	33.272643	189.97122
CPD28399	6.86300092	756.445419	CPD4714	32.589352	428.36169	CPD774	1.045286	457.7638
CPD2840	14.953018	693.39224	CPD4718	32.711868	490.3428	CPD7740	33.261576	150.02727
CPD28400	6.87770638	389.091575	CPD4719	32.795645	683.19447	CPD7744	33.303617	108.94644
CPD28401	6.88784821	810.137843	CPD47208	6.32900127	295.106067	CPD7745	33.280754	98.024736
CPD28402	6.8877046	359.123786	CPD4722	32.878951	726.4818	CPD7749	33.308463	177.98354
CPD28403	6.8860028	378.025928	CPD47225	6.86771615	640.1579	CPD7750	33.544219	184.97941
CPD28404	6.86388933	594.157605	CPD4723	32.870188	710.50839	CPD7754	33.279648	317.87677
CPD28405	6.90475989	775.18798	CPD47259	7.80725774	378.094812	CPD7757	33.212216	124.97887
CPD28406	6.90677713	213.060611	CPD4726	32.861957	688.52479	CPD7758	33.421827	298.87414
CPD28408	6.94339845	590.138254	CPD4728	32.867994	726.4827	CPD7759	33.330531	337.89915
CPD28409	6.95685384	391.071667	CPD4732	33.076954	248.18862	CPD7760	33.356847	338.89596
CPD2841	14.940191	352.26103	CPD4733	33.040169	222.98124	CPD7762	33.389635	375.81346
CPD28410	6.89885623	410.152836	CPD47333	9.67275227	931.833209	CPD7768	33.501935	246.93179
CPD28411	6.95873795	335.103296	CPD4734	33.022667	364.10019	CPD7773	33.545528	111.00966
CPD28413	6.9564356	761.211781	CPD4736	33.029946	180.92124	CPD7776	33.485642	119.96254
CPD28414	6.90835675	226.119779	CPD4738	33.028001	199.96561	CPD7777	33.520459	336.86887
CPD28415	6.94574563	299.080888	CPD4739	33.055086	248.90709	CPD7779	33.381395	87.004118
CPD28417	6.90699755	405.195257	CPD4743	33.061789	200.91074	CPD7783	33.540675	248.03526
CPD28418	6.93522223	353.111933	CPD4744	33.087461	111.00861	CPD7785	33.48483	252.9025
CPD2842	14.932137	658.35514	CPD4749	33.010092	68.987115	CPD779	1.042049	289.8403
CPD28421	6.99692941	344.111392	CPD4750	33.081197	183.97885	CPD7793	33.536008	150.02633
CPD28422	6.97048493	339.153036	CPD4752	33.053842	201.98891	CPD7797	33.544297	95.004096
CPD28424	6.96226	310.315232	CPD4753	33.059975	90.999527	CPD7798	33.57772	69.984427
CPD28426	6.96459923	250.084365	CPD4754	33.069767	173.94937	CPD780	1.04075	285.9974
CPD28428	6.98209032	393.128618	CPD4755	33.061593	210.93935	CPD7800	33.502854	375.81268
CPD28429	6.9999445	332.090463	CPD4756	33.0754	97.954677	CPD7804	33.478325	317.87992

CPD2843	14.919554	698.34802	CPD4758	33.083751	305.89124	CPD7807	33.579251	111.01005
CPD28432	7.0692788	145.052963	CPD4759	33.081676	290.88723	CPD7816	33.500782	325.91066
CPD28433	7.04636137	162.031353	CPD4760	33.07436	69.984707	CPD7820	33.392498	194.98578
CPD28436	7.0499568	220.02236	CPD47664	23.0440044	920.643943	CPD7822	33.618377	111.96413
CPD28439	7.0520441	324.132525	CPD4767	33.066105	132.92314	CPD7824	33.631958	161.96198
CPD2844	14.918393	496.30271	CPD4768	33.080047	212.94245	CPD783	1.038444	609.7252
CPD28444	7.07032103	475.1709	CPD4769	33.106795	123.95658	CPD7831	33.651383	151.9672
CPD28449	7.18880319	420.16697	CPD4774	33.085089	139.98608	CPD7833	33.641309	108.01358
CPD2845	14.972265	365.14391	CPD4779	33.079673	110.01248	CPD784	1.034769	530.8331
CPD28451	7.19185465	312.084016	CPD4781	33.09783	203.14173	CPD7840	33.598092	183.97874
CPD28452	7.15081247	505.22986	CPD4782	33.110547	259.19665	CPD7842	33.635832	204.99028
CPD28453	7.23183569	842.21032	CPD4784	33.087268	257.18673	CPD7843	33.69204	116.96211
CPD28455	7.18730785	519.194626	CPD47858	1.10795103	529.114789	CPD785	1.036063	571.7457
CPD28456	7.18348023	524.149843	CPD47863	1.67167531	198.034675	CPD786	1.043778	508.8422
CPD28457	7.18944623	484.15758	CPD47866	1.32185964	247.103711	CPD787	1.04531	123.9153
CPD28458	7.19549028	270.059183	CPD4787	33.089478	217.15784	CPD7884	1.0028448	257.03385
CPD28459	7.19125952	475.218805	CPD4790	33.0889	98.963015	CPD7894	1.008902	274.12484
CPD2846	14.931415	334.24947	CPD4791	33.079678	180.01775	CPD792	1.043923	205.8785
CPD28460	7.19157134	395.181471	CPD4793	33.089864	121.07486	CPD7922	1.0818877	186.06922
CPD28465	7.35379359	400.13964	CPD47934	8.47936334	972.274773	CPD7966	1.0025693	324.95342
CPD28469	7.20948024	432.122095	CPD4794	33.361306	185.97306	CPD797	1.047067	225.8669
CPD2847	14.931344	357.15748	CPD47941	9.50942176	1208.3021	CPD8007	1.0167757	326.95338
CPD28472	7.08700181	392.131979	CPD4796	33.098	273.19606	CPD8026	1.0119913	134.98961
CPD28474	7.09162204	427.168244	CPD4797	33.106999	266.21169	CPD8030	1.0320802	395.87791
CPD28476	7.20810236	361.23413	CPD4798	33.097166	234.1778	CPD8036	1.0299001	553.7843
CPD28479	7.24829257	362.157176	CPD4799	33.093667	262.19599	CPD8039	1.031639	403.82077
CPD2848	14.970817	674.25871	CPD4801	33.095666	142.95214	CPD8045	1.0316111	357.83661
CPD28481	7.26956297	248.052653	CPD4803	33.117979	235.89742	CPD8046	1.03025	587.81485
CPD28482	7.24846331	900.251837	CPD4805	33.101777	189.12622	CPD8058	1.0362143	525.75516
CPD28485	7.24819906	132.1112	CPD4806	33.094714	155.14148	CPD8060	1.0522856	383.96447
CPD28486	7.2671551	380.167268	CPD4807	33.095727	288.20579	CPD8073	1.0306155	437.93335
CPD28487	7.25530406	268.100185	CPD4808	33.108447	221.18428	CPD8091	1.0468559	389.90617
CPD28492	7.27550609	364.0785	CPD4809	33.108483	169.15803	CPD8096	1.1062205	370.9741
CPD28497	7.27045599	217.094682	CPD4810	33.102684	219.92732	CPD8099	1.0412143	363.78285
CPD2850	14.983233	1000.2586	CPD4811	33.217427	155.97652	CPD810	1.057211	241.8572
CPD28502	7.31113166	162.032137	CPD4812	33.10292	179.13976	CPD8114	1.0496001	432.84465
CPD28503	7.34619471	366.058375	CPD4813	33.109426	207.17251	CPD8130	1.048	375.91273
CPD28504	7.35352787	540.127978	CPD4816	33.115625	192.14163	CPD8132	1.0349744	419.9233
CPD28506	7.37311877	760.108406	CPD4819	33.091466	246.17339	CPD8139	1.0609995	462.84116
CPD28508	7.39473113	158.035989	CPD482	1.021464	704.1747	CPD814	1.04705	641.7881
CPD28509	7.40818284	304.11601	CPD4820	33.097111	100.95822	CPD8141	1.056909	498.81521
CPD28518	7.42209715	286.99496	CPD4821	33.117959	235.1943	CPD8147	1.0522499	279.93061
CPD2852	15.012579	315.27714	CPD4822	33.108542	316.22465	CPD8153	1.0488946	209.96751
CPD28520	7.41536428	354.058895	CPD4823	33.112407	289.2159	CPD8158	1.0394837	335.95716
CPD28521	7.41568742	762.040919	CPD48258	24.3932857	762.5196	CPD8164	1.0577715	164.9609
CPD28522	7.42319471	302.967565	CPD4827	33.105018	243.17346	CPD8167	1.0500103	255.97545
CPD28524	7.41488084	449.001335	CPD4828	33.115333	205.1534	CPD8168	1.0532223	264.91849
CPD28526	7.42133253	400.137956	CPD4829	33.122833	255.16621	CPD8169	1.0602381	206.19939
CPD28527	7.43206847	692.000247	CPD4830	33.105069	151.94039	CPD8172	1.0532055	398.865
CPD2853	14.990859	171.16085	CPD4831	33.120693	433.84994	CPD8180	1.0788139	170.99555
CPD28530	7.43584009	923.332935	CPD4833	33.117213	139.10912	CPD8183	1.0648333	300.87496
CPD28532	7.43639523	922.66629	CPD4834	33.1105	277.21801	CPD8191	1.0847695	170.00937
CPD28533	7.4335631	923.001412	CPD4835	33.108185	245.18706	CPD8193	1.0886539	211.94868
CPD28536	7.48131017	350.063858	CPD4836	33.117046	241.15902	CPD8199	1.0839332	355.06196
CPD28540	7.54950112	294.08704	CPD4838	33.104334	237.18737	CPD8203	1.0891112	324.92169
CPD28541	7.59114666	756.20608	CPD4839	33.115175	328.22586	CPD8205	1.1218223	191.04642
CPD28542	7.57832332	551.303607	CPD4840	33.115196	153.12613	CPD8213	1.0962287	202.21602
CPD28543	7.58099296	343.125045	CPD4841	33.129292	202.14479	CPD8215	1.1154232	281.07263
CPD28548	7.5977513	327.203511	CPD4842	33.079524	222.98163	CPD8217	1.175904	199.03754
CPD2855	15.026284	255.25574	CPD4843	33.128077	229.15546	CPD8226	1.4327856	86.0361
CPD28551	7.66874477	438.206967	CPD4844	33.127532	227.14326	CPD8229	1.129943	354.07267
CPD28554	7.65126315	325.172667	CPD4845	33.119476	453.84551	CPD8231	1.1247119	516.11393
CPD28557	7.70882423	590.194625	CPD4847	33.112154	176.12969	CPD8236	1.1119189	438.12879
CPD28558	7.72495365	585.242331	CPD48484	6.97667381	202.0329	CPD8243	1.1265874	530.03346
CPD28559	7.70623128	458.176062	CPD4849	33.115805	265.21192	CPD8248	1.1381615	111.04358
CPD2856	15.017793	675.98017	CPD4850	33.115339	264.00803	CPD825	1.037335	206.9809
CPD28560	7.73311292	445.123322	CPD4851	33.124898	150.17564	CPD8254	1.1500665	567.17984

CPD28563	7.75362766	852.069246	CPD4852	33.117544	265.92078	CPD8256	1.1415852	563.21019
CPD28565	7.72784563	385.100325	CPD4853	33.116643	220.16727	CPD8257	1.1170094	203.05573
CPD28568	7.7332527	820.128005	CPD4854	33.216213	231.17292	CPD826	1.045389	424.8839
CPD28569	7.72899704	302.967445	CPD48545	9.15930377	619.13205	CPD8261	1.1505554	388.22016
CPD2857	15.021685	677.97977	CPD4855	33.118816	395.8765	CPD8262	1.2916833	607.07159
CPD28571	7.74125564	479.011293	CPD4859	33.119039	314.21277	CPD8264	1.1426	549.19138
CPD28572	7.74089378	384.0692	CPD4860	33.130661	167.91433	CPD8265	1.1367996	562.22511
CPD28573	7.74634789	176.046632	CPD4861	33.125714	431.85033	CPD8266	1.1185583	396.99329
CPD28574	7.72727396	206.057997	CPD4862	33.223984	111.00799	CPD8269	1.1240802	131.05757
CPD28577	7.73362833	286.995037	CPD4863	33.127848	342.89813	CPD8270	1.1377691	627.12775
CPD28579	7.72720139	822.07081	CPD4865	33.270172	297.87945	CPD8272	1.1559996	259.18475
CPD2858	15.039981	326.00052	CPD4867	33.123345	361.84679	CPD8277	1.1860638	246.12129
CPD28581	7.74413853	437.995721	CPD48675	18.8577991	294.21931	CPD8280	1.16214	463.01071
CPD28584	7.76042994	356.110857	CPD4868	33.120867	847.71719	CPD8282	1.1373127	531.21179
CPD28586	7.71069015	194.058179	CPD4869	33.118526	114.95246	CPD8285	1.1625407	209.13888
CPD28587	7.73136938	414.082606	CPD4871	33.125218	411.85595	CPD8287	1.1273226	744.3618
CPD28589	7.75564626	448.099965	CPD4872	33.111499	225.18706	CPD8289	1.1413173	261.1322
CPD28590	7.74370335	756.20968	CPD4873	33.129875	432.85156	CPD829	1.060563	344.8851
CPD28592	7.74637794	390.057567	CPD4875	33.121317	313.21622	CPD8292	1.1616954	297.13517
CPD28595	7.74821069	509.024711	CPD4876	33.207958	225.12802	CPD8293	1.2771294	232.11916
CPD28597	7.75772608	397.080045	CPD4881	33.121733	181.15755	CPD8294	1.1570004	349.14125
CPD28598	7.70986438	448.186907	CPD4882	33.134118	439.3465	CPD8297	1.1577368	289.12556
CPD28602	7.80839861	219.058525	CPD4883	33.122359	441.87075	CPD8299	1.1580445	245.13883
CPD28605	7.81366508	547.166217	CPD4886	33.125572	274.90756	CPD8301	1.1697725	112.09854
CPD28609	7.82959694	594.158645	CPD4888	33.125678	109.00201	CPD8303	1.15935	177.11476
CPD28613	7.85314864	325.11578	CPD4889	33.127694	239.09669	CPD8304	1.1316045	431.16568
CPD28614	7.84253523	345.062685	CPD4890	33.127452	257.97643	CPD8311	1.1878128	279.20091
CPD28615	7.8295215	316.057005	CPD4892	33.12635	238.95945	CPD8313	1.1486748	188.11668
CPD28616	7.84716911	511.197806	CPD4893	33.127402	150.02804	CPD8314	1.1614166	309.10672
CPD28617	7.86420605	624.169733	CPD4894	33.128152	78.983353	CPD8321	1.1666249	224.01175
CPD28618	7.87277615	290.091894	CPD4895	33.128564	587.80577	CPD8323	1.16212	92.049624
CPD28619	7.8295443	439.145009	CPD4899	33.122192	281.89911	CPD8326	1.4720846	305.14639
CPD2862	15.049039	570.2769	CPD48995	1.03870708	478.063141	CPD8327	1.1727225	253.07597
CPD28620	7.86715685	621.124974	CPD4900	33.127333	253.93571	CPD8331	1.5196582	618.03944
CPD28621	7.86700041	621.627583	CPD4902	33.125345	368.91663	CPD8338	1.4141224	72.022257
CPD28622	7.89724326	629.121905	CPD49021	1.089098	356.054211	CPD834	1.0484	191.9045
CPD28623	7.87128671	694.091518	CPD49036	1.34961654	271.99083	CPD8341	1.140277	460.01059
CPD28624	7.88353667	1226.28942	CPD4904	33.13215	627.77137	CPD8342	1.2055489	285.12959
CPD28627	7.86286113	287.131689	CPD4905	33.127267	279.98493	CPD8346	1.2446483	197.08628
CPD28628	7.87639247	500.112235	CPD4906	33.131072	733.74213	CPD8348	1.2056241	290.01282
CPD28629	7.79975671	508.100979	CPD4907	33.12731	197.93297	CPD8353	1.1938187	206.04442
CPD2863	15.041462	249.99161	CPD4908	33.1294	356.89601	CPD8354	1.5067285	417.30625
CPD28630	7.93929464	962.26803	CPD4909	33.131583	477.82303	CPD8356	1.1825672	301.12376
CPD28633	7.95110191	984.24967	CPD4911	33.129455	495.83597	CPD8360	1.1602085	480.06967
CPD28636	7.72374229	380.167627	CPD49120	8.56958691	746.25549	CPD837	1.068363	364.8779
CPD28637	8.05080201	362.157556	CPD49122	8.57815654	248.051716	CPD8371	1.5590296	85.089358
CPD2864	15.01074	287.28107	CPD49124	8.57718	381.109947	CPD8372	1.2353761	70.006069
CPD28642	8.02965998	573.138687	CPD4913	33.13512	93.037828	CPD8396	1.7953813	220.02375
CPD28643	8.02993343	432.106718	CPD4914	33.132143	387.88003	CPD8400	1.1987746	470.07831
CPD28646	8.03279989	395.18027	CPD49145	8.62427781	932.757513	CPD8403	1.4453495	253.08211
CPD28647	8.02724013	932.257105	CPD49149	9.32458853	940.224823	CPD8407	1.4453386	223.06874
CPD28648	7.94245862	192.147393	CPD4917	33.128519	379.88258	CPD8408	1.4419496	145.00626
CPD28649	8.03668464	1108.32482	CPD4918	33.129133	457.84949	CPD841	1.052045	195.8566
CPD2865	15.133304	213.2452	CPD4919	33.130683	116.99017	CPD8412	1.3817439	231.98641
CPD28650	8.06851133	353.168341	CPD4920	33.131951	109.12961	CPD8420	1.589246	84.021692
CPD28651	8.02883482	946.272689	CPD4921	33.128433	65.011958	CPD8424	1.4645988	339.11602
CPD28652	8.03283441	581.12515	CPD4923	33.129068	109.09385	CPD8425	1.4275696	125.03326
CPD28654	8.04196039	282.0711	CPD4924	33.132842	511.82472	CPD8432	1.285093	70.0059
CPD28656	8.07721793	564.148571	CPD4925	33.132448	356.39293	CPD8434	1.5545695	188.03421
CPD28659	8.10513507	728.386943	CPD4926	33.128036	169.05733	CPD8436	1.5676138	206.04521
CPD2866	15.042929	324.22885	CPD4927	33.126229	373.87661	CPD8441	1.5580001	267.04128
CPD28660	8.09600135	727.88596	CPD4929	33.131	660.77455	CPD8442	1.5485192	405.13005
CPD28663	8.11924795	474.20951	CPD4930	33.133379	376.88885	CPD8443	1.5427946	309.10451
CPD28664	8.08081006	362.157013	CPD4932	33.137764	73.025891	CPD8453	1.564	419.14616
CPD28667	8.12384207	458.214057	CPD4933	33.128167	607.78226	CPD8454	1.5614815	387.12466
CPD28669	8.126778	344.146056	CPD4935	33.125132	541.81293	CPD8456	1.5625	307.23575
CPD28670	8.13949636	442.182182	CPD4936	33.139122	190.14607	CPD8459	1.56425	169.07238

CPD28672	8.17649072	516.217463	CPD4937	33.129647	404.86484	CPD8460	1.5193676	204.11128
CPD28673	8.148366	460.763221	CPD4940	33.13305	595.79254	CPD8461	1.5609384	319.15057
CPD28674	8.14599468	493.251156	CPD4942	33.127265	314.91082	CPD8463	1.8477386	426.18386
CPD28675	8.14104709	262.120332	CPD4943	33.139416	167.14107	CPD8464	1.5694688	231.1203
CPD28676	8.14000073	264.06487	CPD4946	33.135194	399.87223	CPD8466	1.5671923	259.1866
CPD28677	8.16733234	198.070767	CPD4947	33.135742	210.18286	CPD8467	1.5714604	245.13498
CPD28679	8.15456844	498.207059	CPD4950	33.122094	240.19034	CPD8468	1.5555669	188.11485
CPD28681	8.15512536	566.127225	CPD4951	33.144765	48.992335	CPD8469	1.4737226	204.0243
CPD28682	8.16176332	316.9826	CPD4953	33.1369	502.83401	CPD8470	1.567875	460.15708
CPD28683	8.14409589	463.01583	CPD4954	33.137857	611.77924	CPD8471	1.5648666	275.14735
CPD28685	8.16400072	644.030075	CPD4955	33.135905	371.87828	CPD8473	1.5625588	191.15076
CPD28687	8.18598748	320.089794	CPD4956	33.144844	52.012032	CPD8474	1.6661896	317.15475
CPD28688	8.1783691	545.2113	CPD4960	33.132203	327.89315	CPD8475	1.5650667	802.17624
CPD28689	8.1601924	916.26135	CPD4962	33.129177	569.80979	CPD8477	1.5673125	550.03901
CPD2869	15.209745	365.14387	CPD49634	1.0375	217.042756	CPD8478	1.5694376	315.14513
CPD28692	8.16173867	1078.31419	CPD49635	1.03137517	233.019875	CPD848	1.055903	252.9913
CPD28694	8.17530625	301.010677	CPD4964	33.137684	392.87274	CPD8480	1.5658888	214.13327
CPD28696	8.15710708	778.109065	CPD49641	1.04213198	314.024033	CPD8486	1.5669999	644.0933
CPD28697	8.16064759	350.063465	CPD49647	1.0633014	496.96366	CPD8488	1.5683333	268.14994
CPD28699	8.12578285	494.169864	CPD49648	1.05437506	426.064688	CPD8493	1.2260089	375.15838
CPD2870	15.115112	349.97955	CPD4965	33.136322	52.077186	CPD8494	1.5843342	447.19893
CPD28700	8.17181037	550.166836	CPD49650	1.05512488	396.00895	CPD8497	1.5760248	218.126
CPD28701	8.19600417	489.151522	CPD49652	1.07083362	182.123306	CPD8498	1.5542038	535.95409
CPD28702	8.12833775	417.164247	CPD49653	1.06680113	325.93244	CPD8499	1.638317	269.08915
CPD28703	8.18673283	790.068379	CPD49658	1.08816243	234.030482	CPD8501	1.5717138	618.03258
CPD28704	8.1802938	738.199365	CPD49659	1.08481249	318.091969	CPD8508	1.6358167	337.14947
CPD28705	8.18039705	680.15807	CPD4966	33.13525	306.91754	CPD851	1.045227	378.8973
CPD28706	8.1799084	628.058055	CPD49663	1.08428624	443.0593	CPD8514	1.6106293	287.19459
CPD28707	8.18420107	146.142693	CPD49667	1.08716668	364.2481	CPD8516	1.5875417	267.93967
CPD28708	8.18355818	511.197767	CPD49669	1.086125	364.415362	CPD8517	1.5794807	158.10449
CPD28711	8.18711047	388.07775	CPD4967	33.134524	603.7845	CPD852	1.054696	294.9308
CPD28712	8.18378661	146.036221	CPD49670	1.10183921	186.049044	CPD8520	1.6134669	492.0302
CPD28713	8.1826494	259.983365	CPD49672	1.11830817	113.047908	CPD8537	1.7015727	473.21094
CPD28714	8.15888093	368.076015	CPD49678	1.10759998	149.069475	CPD8541	1.6032449	52.01111
CPD28715	8.18479871	322.01934	CPD49679	1.09692255	483.138685	CPD8542	1.7574271	290.07608
CPD28716	8.20900031	646.189418	CPD4968	33.136133	142.14558	CPD8543	1.7089349	131.04158
CPD28719	8.21414595	421.99337	CPD49682	1.10611127	348.253739	CPD8548	1.5083697	132.04783
CPD2872	15.244805	475.27408	CPD49683	1.10306059	485.300124	CPD8551	2.0318711	258.08935
CPD28720	8.21579569	385.100445	CPD49685	1.106882	349.419382	CPD8556	1.6209535	183.05246
CPD28721	8.21294395	289.094695	CPD49689	1.12255467	651.093399	CPD8557	1.9191538	187.10712
CPD28722	8.1745304	307.1078	CPD4969	33.135333	384.89405	CPD8560	1.9237461	188.1142
CPD28723	8.20278405	758.130457	CPD49699	1.14285011	189.131775	CPD8562	2.0019229	238.09778
CPD28724	8.19913244	361.010267	CPD4970	33.132647	530.81818	CPD8565	2.3259936	82.033464
CPD28726	7.86237217	420.11523	CPD49700	1.13399989	705.176755	CPD8567	2.1262256	321.17423
CPD28727	8.24685665	598.121871	CPD49701	1.14200021	671.169	CPD8568	2.0864455	216.14859
CPD28729	8.24114108	555.126886	CPD49702	1.14633332	988.208256	CPD8571	2.1142609	282.12111
CPD2873	15.190207	251.22383	CPD49703	1.1495999	309.180093	CPD8582	2.1902489	296.09684
CPD28730	8.25393888	726.197539	CPD49704	1.14811116	818.172744	CPD8585	2.337524	332.10294
CPD28734	8.27632279	478.094825	CPD49707	1.15133671	305.080722	CPD8589	2.4703849	218.12596
CPD28738	8.46909052	86.000616	CPD49709	1.14875001	709.300175	CPD8597	2.5099772	315.16925
CPD28739	8.2735445	126.031105	CPD49711	1.15172727	754.275627	CPD8599	2.5709038	245.13726
CPD2874	15.207664	698.34804	CPD49713	1.15305002	291.195	CPD860	1.058334	246.919
CPD28740	8.43079772	108.020572	CPD49714	1.23242424	307.186327	CPD8600	2.5916453	202.13059
CPD28741	8.27936558	374.100489	CPD49715	1.14776927	725.127	CPD8604	2.7729524	341.10966
CPD28743	8.48652711	104.011325	CPD49716	1.15604997	321.2059	CPD8606	2.723475	232.14093
CPD28744	8.2813044	358.126565	CPD49718	1.153125	739.311413	CPD8609	2.7283828	188.11464
CPD28745	8.31036829	248.053025	CPD4972	33.12703	717.76147	CPD8613	2.873826	245.1367
CPD28746	8.2695531	397.194411	CPD49720	1.15599993	360.252347	CPD8616	2.9555474	259.15234
CPD28748	8.28677251	353.168008	CPD49721	1.16178945	282.1327	CPD8618	3.0334911	280.1418
CPD28749	8.31647796	318.134159	CPD49724	1.15858856	274.095618	CPD8622	3.104605	202.13039
CPD2875	15.216136	693.39256	CPD49726	1.1609445	163.099983	CPD8624	3.0662448	208.09386
CPD28752	8.45085063	144.041258	CPD49727	1.16523528	788.195894	CPD8625	3.1300347	232.14144
CPD28754	8.31603889	84.0205875	CPD49728	1.15929417	950.251982	CPD8626	3.1667797	188.11453
CPD28758	8.30389752	902.24695	CPD4973	33.134862	488.81962	CPD8630	3.4263846	260.13555
CPD28759	8.31906084	962.267633	CPD49732	1.1712944	446.218453	CPD8631	3.4304596	242.12524
CPD2876	15.209366	352.26044	CPD49739	1.29441207	126.030294	CPD864	1.0487	256.9635
CPD28760	8.3541062	491.236661	CPD4974	33.131353	446.84459	CPD8642	3.7803172	137.04672

CPD28763	8.38905571	520.213037	CPD49740	1.28953828	709.157792	CPD8647	4.2576013	252.11052
CPD28766	8.44769688	578.163416	CPD49741	1.29639997	364.182995	CPD8648	4.2521509	230.16162
CPD2877	15.19587	357.15762	CPD49744	1.31284205	614.145968	CPD8652	4.375606	317.16011
CPD28772	8.41899429	428.115491	CPD49745	1.31065003	285.0607	CPD8653	4.4456883	279.12436
CPD28773	8.43341672	466.053883	CPD49749	1.31975032	547.104042	CPD8656	4.6442434	293.13777
CPD28777	8.81241796	816.211558	CPD4975	33.134353	481.33751	CPD8658	4.7330821	222.09957
CPD28779	8.441049	259.984037	CPD49750	1.33898793	579.169575	CPD8660	5.0015914	236.11743
CPD28781	8.43293632	450.079547	CPD49751	1.32244426	591.086678	CPD8662	5.3335781	162.03142
CPD28782	8.43076266	316.9828	CPD49752	1.31424998	750.068175	CPD8664	5.0958822	162.0848
CPD28784	8.44853603	493.027106	CPD49753	1.31068769	336.151	CPD8665	5.0878387	331.17574
CPD28786	8.42818006	445.123564	CPD49755	1.3147139	504.047757	CPD8666	5.0849282	528.23116
CPD28787	8.43753873	410.084365	CPD49756	1.32070003	264.13118	CPD8667	5.100162	378.05994
CPD28788	8.43450148	848.16148	CPD49758	1.32739983	501.063185	CPD8668	5.1200259	346.12329
CPD2879	15.275661	274.19305	CPD4976	33.132563	465.83749	CPD8669	5.1136747	362.09741
CPD28791	8.45104887	176.04663	CPD49760	1.3361878	308.157869	CPD8670	5.1212998	341.16782
CPD28792	8.48520053	398.086141	CPD49761	1.25259212	419.072471	CPD8672	5.2979574	616.26921
CPD28793	8.47104902	452.005155	CPD49762	1.37413605	155.049311	CPD8675	5.3950017	276.11172
CPD28794	8.45814147	850.094714	CPD49764	1.4127066	533.018682	CPD8677	5.4010403	294.12194
CPD28796	8.45239715	380.07396	CPD49765	1.35404946	434.035757	CPD8678	5.425097	309.12749
CPD28798	8.64362404	350.063538	CPD49774	1.32096069	149.176622	CPD8679	5.4544303	389.17922
CPD28801	8.45607605	459.1405	CPD4978	33.135	405.8783	CPD8680	5.4853972	395.17239
CPD28804	8.47383626	415.111223	CPD49780	1.5574167	446.21845	CPD8684	5.7068441	402.21361
CPD28805	8.50033438	512.187883	CPD49782	1.52143242	174.126409	CPD8685	5.7623605	373.22024
CPD28808	8.45770506	190.121971	CPD49783	1.55949999	202.21619	CPD8687	5.7863987	293.13762
CPD2881	15.344013	314.29202	CPD49784	1.56314997	182.12132	CPD8691	5.8643381	503.25692
CPD28811	8.50399603	454.181229	CPD49786	1.56375	163.098935	CPD8692	5.876641	345.22265
CPD28812	8.18299596	416.131837	CPD49787	1.56294735	146.035242	CPD8697	5.9851342	264.14651
CPD28813	8.5276925	263.0532	CPD49789	1.562625	172.132494	CPD8700	5.9415228	313.17336
CPD28815	8.5311725	324.056518	CPD49790	1.56365	189.131495	CPD8704	6.0396416	477.16233
CPD2882	15.290464	208.1805	CPD49792	1.56394742	234.035553	CPD8706	6.0736608	392.02289
CPD28820	8.52612719	142.098075	CPD49793	1.56988885	135.065033	CPD8707	6.0336693	239.13925
CPD28821	8.50737439	302.042991	CPD49794	1.56724997	224.186538	CPD8709	6.0565033	493.13473
CPD28826	8.53090026	344.14511	CPD49796	1.58278851	207.088957	CPD871	1.050915	337.839
CPD28827	8.53211091	339.190178	CPD49797	1.57106665	251.100227	CPD8715	6.0817144	1068.2189
CPD28828	8.52370231	347.266376	CPD49799	1.57769223	685.081954	CPD8717	6.3663761	402.11611
CPD28829	8.54688923	614.036944	CPD4980	33.130417	560.81588	CPD8718	6.1007113	526.12355
CPD28834	8.56715378	290.091792	CPD49803	1.75533031	377.12562	CPD8720	6.0792992	321.085
CPD28838	8.59999122	358.130922	CPD49804	1.58857156	456.220364	CPD8721	6.1406107	327.21429
CPD28839	8.57538067	1240.34474	CPD49805	1.60989905	162.05037	CPD8724	6.1087437	244.17533
CPD2884	15.379087	475.27514	CPD49807	1.46422834	595.126499	CPD8726	6.1276771	291.12098
CPD28841	8.61520823	254.081769	CPD49808	1.59124998	284.152008	CPD8727	6.1153619	529.26165
CPD28843	8.6001462	1270.35705	CPD49809	1.60249962	231.052294	CPD8734	6.2310078	540.29948
CPD28844	8.57305927	1108.32094	CPD4981	33.137611	364.38171	CPD8735	6.1916021	359.20596
CPD28847	8.63073348	581.615767	CPD49812	1.62374953	269.14305	CPD8738	6.1793797	402.20955
CPD2885	15.470283	229.23988	CPD49815	1.60205276	559.072726	CPD874	1.055964	180.9549
CPD28852	8.63289032	581.114311	CPD49816	1.59714993	493.052535	CPD8741	6.5230969	1126.2986
CPD28854	8.63789019	573.128668	CPD4982	33.137424	343.8687	CPD8743	6.2715419	555.23153
CPD28856	8.59282391	398.133275	CPD49821	1.61678963	354.105253	CPD8744	6.2848222	560.1878
CPD28859	8.62402142	604.151138	CPD49826	1.63728573	384.190093	CPD8745	5.9732829	285.16644
CPD28860	8.65564696	946.2735	CPD49827	1.65449984	579.134125	CPD8750	6.3262293	502.20684
CPD28861	8.66834352	492.111706	CPD49829	1.67072132	378.202133	CPD8754	6.3681701	275.12782
CPD28862	8.74298581	800.217107	CPD4983	33.130267	258.93145	CPD8756	6.3476063	239.13911
CPD28865	8.65825111	946.534869	CPD49831	1.68409284	417.042355	CPD8758	6.3622559	294.15718
CPD28869	8.67834327	451.2046	CPD49832	1.68609018	194.9859	CPD876	1.067636	430.8463
CPD28870	8.66716511	500.097783	CPD49833	1.69519613	385.086953	CPD8760	6.3948579	376.07811
CPD28871	8.68014161	670.863986	CPD49834	1.68344974	291.1947	CPD8761	6.4281413	262.13111
CPD28872	8.66637492	432.106556	CPD49835	1.69695014	485.072595	CPD8763	6.4748774	385.04307
CPD28875	8.70946835	886.251737	CPD49836	1.66750422	516.191423	CPD8766	6.5365485	772.20324
CPD28876	8.7192792	493.249579	CPD49839	1.69341167	317.102794	CPD8767	6.5875495	301.19827
CPD2888	15.550208	987.49521	CPD4984	33.151522	210.97059	CPD8769	6.4958269	327.21032
CPD28881	8.71874734	192.150265	CPD49840	1.6991001	419.05388	CPD8770	6.5112873	264.14626
CPD28882	8.72184397	916.262295	CPD49842	1.70130051	666.15336	CPD8771	6.5242319	186.1309
CPD28883	8.71969881	498.20651	CPD49843	1.70952979	194.053553	CPD8772	6.5545376	586.12889
CPD28885	8.74058341	456.152225	CPD49848	1.72073342	501.051373	CPD8774	6.4803721	226.16692
CPD28886	8.72362942	257.086753	CPD4985	33.12441	155.0417	CPD8775	6.4538097	297.14556
CPD28888	8.75139691	405.06562	CPD49851	1.74788873	725.134578	CPD8780	6.537622	345.19506
CPD2889	15.578	743.44309	CPD49852	1.69967368	517.034412	CPD8782	6.5362472	343.21907

CPD28892	8.74720198	132.093413	CPD49853	1.73871448	281.093279	CPD8783	6.5651716	359.20403
CPD28893	8.75505015	397.07983	CPD49855	1.74044418	361.0499	CPD8784	6.6838887	663.64281
CPD28894	8.75324791	756.210255	CPD49856	1.83054481	630.126681	CPD8789	6.513904	477.22003
CPD28899	8.75487103	594.158319	CPD49859	1.72721926	447.114675	CPD879	1.052524	301.982
CPD2890	15.589292	238.15359	CPD4986	33.13684	347.86332	CPD8790	6.6166115	244.17707
CPD28902	8.75419912	185.105013	CPD49862	1.76427262	383.022409	CPD8792	6.6613372	301.19842
CPD28904	8.76059544	231.11036	CPD49867	1.8430003	307.1882	CPD8799	6.7844024	408.10397
CPD28906	8.75688882	213.102333	CPD49868	1.84043281	291.19355	CPD8802	6.6932322	262.13192
CPD28908	8.76619884	440.166607	CPD49869	1.84790472	152.031044	CPD8808	6.5094197	376.17284
CPD28911	8.77325416	400.172319	CPD4987	33.136659	429.36003	CPD881	1.047091	408.9074
CPD28912	8.79539974	510.17337	CPD49873	1.89903259	367.045823	CPD8810	6.7235706	407.17187
CPD28913	8.77476278	528.180171	CPD49877	1.98543603	189.130182	CPD8812	6.7715716	706.24246
CPD28918	8.77781034	428.131919	CPD49881	1.97349746	393.126667	CPD8816	6.8238013	345.18826
CPD2892	15.542236	927.34525	CPD49883	1.95055873	315.07985	CPD8817	6.8334984	373.13601
CPD28922	8.77329954	423.17511	CPD49885	1.95961285	348.113407	CPD8821	6.8530027	180.04265
CPD28924	8.82641491	466.239273	CPD49887	1.97285694	155.160041	CPD8823	6.9312784	206.23206
CPD28930	8.80555879	190.137481	CPD4989	33.136074	295.95886	CPD8827	6.9652785	386.19103
CPD28931	8.8258015	1314.383	CPD49893	1.97260414	142.0452	CPD8829	6.8963324	447.17279
CPD28934	8.85262301	1046.57569	CPD49894	2.10687315	583.68904	CPD8830	6.9198595	598.14696
CPD28937	8.83749919	144.04117	CPD49899	2.03081756	447.116108	CPD8837	6.9739172	370.21855
CPD28938	8.84590944	376.083236	CPD4990	33.133214	393.87666	CPD884	1.0571	346.8953
CPD28939	8.68981545	698.251354	CPD49901	2.04621936	301.024575	CPD8843	7.0417765	274.15196
CPD28940	8.84219863	322.16204	CPD49902	2.03879348	229.069993	CPD8845	7.0981886	649.32299
CPD28942	8.84378461	96.0933105	CPD49903	2.02563344	471.67318	CPD8853	7.0571152	212.03432
CPD28943	8.83984443	114.102826	CPD49905	2.0355887	369.143282	CPD8854	6.9788218	401.22495
CPD28944	8.84453816	800.21502	CPD49908	2.14842603	270.232954	CPD8855	7.2410193	206.06034
CPD28945	8.85299624	962.26821	CPD49915	2.05789578	266.0785	CPD8864	6.9095524	352.12986
CPD28946	8.84293613	160.108038	CPD49917	2.09555634	437.135319	CPD8867	7.2287214	321.16819
CPD28948	8.855862	508.096807	CPD49918	2.02761714	449.151267	CPD8868	7.4493009	436.05837
CPD2895	15.629463	586.27688	CPD49920	2.04729953	492.6971	CPD8870	7.3544637	414.24447
CPD28950	8.84519869	180.055875	CPD49922	2.03973173	274.064633	CPD8873	7.3840847	351.17811
CPD28952	8.8584954	500.110021	CPD49926	2.05030237	500.68406	CPD8875	7.376941	378.19139
CPD28953	8.89319474	587.314107	CPD4993	33.156995	254.93672	CPD8877	7.3674231	329.23047
CPD28956	8.87639736	588.18349	CPD49930	2.07969419	377.1269	CPD8881	7.4072332	301.14189
CPD28957	8.87614752	558.17288	CPD49932	2.08389523	462.668433	CPD8882	7.2135031	230.04313
CPD28958	8.87600059	729.259069	CPD49935	2.17430171	339.119464	CPD8888	7.5306031	448.10156
CPD28959	8.89316922	484.157683	CPD49939	2.08462178	554.682863	CPD8889	7.4733357	610.15443
CPD2896	15.631469	414.20425	CPD4994	33.137187	497.81478	CPD8891	7.5781385	358.21995
CPD28960	8.88272987	623.220147	CPD49942	2.0623134	482.197619	CPD8892	7.6010066	491.14353
CPD28961	8.87618312	628.175887	CPD49946	2.01390061	666.1792	CPD8893	7.6123062	331.20918
CPD28964	8.90125881	275.18794	CPD49956	1.9897666	164.058667	CPD8894	7.6427241	1480.3925
CPD28966	8.90879572	1254.36179	CPD49957	2.1059054	322.136791	CPD8895	7.6381967	767.66026
CPD28967	8.918145	669.14932	CPD4996	33.135889	351.38069	CPD8896	7.6553113	315.21415
CPD28968	8.91725745	653.177226	CPD49965	2.11294824	552.154676	CPD8897	7.6212653	458.26983
CPD28969	8.91842819	661.162206	CPD49967	2.09505339	163.042945	CPD8899	7.6451629	173.09807
CPD28970	8.90688396	1108.32377	CPD49969	2.16612811	258.231194	CPD890	1.056667	310.9036
CPD28971	8.9494459	654.155123	CPD4997	33.135818	416.35587	CPD8900	7.6624567	354.18744
CPD28972	8.91539748	1284.37303	CPD49970	2.10984998	566.169662	CPD8901	7.694683	301.19884
CPD28973	8.91904907	661.663711	CPD49972	2.13638698	565.667289	CPD8903	7.7447553	459.2665
CPD28974	8.9151335	916.262847	CPD49973	2.10408997	579.175235	CPD8907	7.853581	359.2046
CPD28976	8.92006797	852.26774	CPD49974	2.12420065	697.20786	CPD8908	7.7889141	345.22569
CPD28977	8.91954968	669.649765	CPD49977	2.14416975	688.716076	CPD8909	7.7410871	359.12253
CPD2898	15.664775	517.31595	CPD4998	33.137324	372.88748	CPD891	1.050876	414.8573
CPD28980	8.96724606	262.120842	CPD49980	2.10790196	595.152929	CPD8910	7.8472207	373.22095
CPD28981	8.96698157	502.185837	CPD49981	2.15898978	556.133656	CPD8911	7.8777373	331.20903
CPD28986	9.02571201	910.212786	CPD49982	2.12096986	562.1844	CPD8912	8.0012326	774.17115
CPD28987	9.03132968	613.634344	CPD49983	2.14095196	354.103625	CPD8914	7.9593016	315.21488
CPD28989	9.03366683	594.368933	CPD49984	2.13899762	689.71682	CPD8915	7.9556134	250.14281
CPD28990	9.03105177	1242.23558	CPD49985	2.15122083	573.657964	CPD8916	7.9669222	301.19869
CPD28991	9.03664942	613.133435	CPD49986	2.10804095	366.167311	CPD8917	8.163264	438.07501
CPD28992	9.03519497	594.158345	CPD49987	1.87976706	185.066975	CPD8920	8.0767093	743.68494
CPD28993	9.02799984	910.715021	CPD49988	2.1471069	455.169595	CPD8922	8.1493931	871.26839
CPD28997	9.04276942	440.167677	CPD49989	2.0923292	578.1616	CPD8923	8.0450241	1524.4177
CPD28998	9.03949448	324.039185	CPD4999	33.131499	307.91089	CPD8925	8.084421	602.79591
CPD28999	9.03809635	316.053445	CPD49990	2.14300196	434.153046	CPD8926	8.0893923	359.20461
CPD2900	15.761108	309.22508	CPD49992	2.08944834	650.199935	CPD8927	8.0861588	603.29799
CPD29002	9.04079944	432.10606	CPD49993	2.15855584	584.129783	CPD893	1.053172	194.971

CPD29003	9.06594059	308.086047	CPD49994	2.13560652	696.705173	CPD8931	8.3524968	662.18346
CPD29004	9.03553659	934.124013	CPD49995	2.14866673	287.20888	CPD8932	8.2012862	200.01711
CPD29005	9.0449277	678.114157	CPD49996	2.14464361	382.379636	CPD8933	8.2013142	192.03205
CPD29006	9.06859325	728.266867	CPD49998	2.13438904	689.216661	CPD8934	8.2012512	270.97763
CPD29007	9.0737584	646.207006	CPD49999	2.13700074	611.128283	CPD8937	8.2362649	925.73403
CPD29008	9.06662625	782.684162	CPD50002	2.14561243	589.133506	CPD8938	8.2184997	528.2876
CPD29010	9.0759174	448.139925	CPD50003	2.1353083	525.66792	CPD894	1.0372	399.9688
CPD29011	9.06431539	782.190981	CPD50004	2.16432137	584.6611	CPD8940	8.2352979	926.23491
CPD29012	9.07934529	932.2576	CPD50005	2.13524201	429.14695	CPD8947	8.13364	685.65464
CPD29013	9.04868746	770.203828	CPD50006	2.17119688	538.157882	CPD8948	8.3247972	459.26549
CPD29014	9.08499811	493.089955	CPD50007	2.15705738	380.138412	CPD8949	8.6660095	496.18976
CPD29016	9.0862059	485.104	CPD50008	2.11678627	565.169	CPD8955	8.4540609	359.09857
CPD29019	9.13898996	560.151938	CPD50010	2.13533929	532.656886	CPD8956	8.4088123	1140.3142
CPD29020	9.14078654	478.094247	CPD50011	2.15695023	308.158415	CPD8958	8.4505787	736.21915
CPD29021	9.11086595	422.120713	CPD50013	2.11631729	740.1585	CPD8959	8.4436682	898.27168
CPD29022	9.11217337	346.106294	CPD50014	2.15699976	389.876	CPD896	1.054372	165.9465
CPD29023	9.11441674	417.162853	CPD50015	2.13243808	614.148625	CPD8960	8.4711841	208.14617
CPD29025	9.13294782	740.195615	CPD50016	2.1472006	646.101225	CPD8961	8.5099058	414.11794
CPD29026	9.13230114	902.2479	CPD50017	2.14889982	382.143905	CPD8962	8.5401628	335.18262
CPD29027	9.16592335	968.290231	CPD50018	2.16381436	167.05605	CPD8963	8.5125994	659.25417
CPD29028	9.15546633	193.044247	CPD50019	2.16352897	513.883029	CPD8966	8.5673329	743.1765
CPD29035	9.14422383	213.169133	CPD5002	33.143526	408.8526	CPD8967	8.5446273	365.19326
CPD29038	9.53505904	990.054692	CPD50022	2.1567999	390.133745	CPD8968	8.6192441	393.19066
CPD29039	9.26742876	326.169943	CPD50023	2.14183141	382.814488	CPD8971	8.6035843	396.15355
CPD2904	15.849154	287.25528	CPD50025	2.16555811	680.214338	CPD8974	8.6484699	335.18106
CPD29044	9.22795854	816.21088	CPD50027	2.15879943	61.0534571	CPD8979	8.6448768	364.16252
CPD29049	9.33805004	600.181468	CPD50028	2.16189466	462.070768	CPD8980	8.6711241	194.05764
CPD29050	9.352387	756.19035	CPD50029	2.1606491	543.128315	CPD8982	8.6497419	408.08175
CPD29051	9.36568431	212.171362	CPD50030	2.15811645	516.678259	CPD8986	8.6728928	319.01461
CPD29059	9.41074218	1124.29929	CPD50031	2.1517	352.14704	CPD8987	8.6644802	360.04105
CPD29066	9.93896793	194.053346	CPD50033	2.14935151	513.463125	CPD8988	8.6469002	206.05839
CPD29067	9.4667512	367.183719	CPD50034	2.16590014	247.2437	CPD8989	8.6638941	224.19946
CPD29069	9.49798946	512.1895	CPD50035	2.16431445	525.167042	CPD8990	8.9744085	330.12497
CPD2907	15.77862	648.4011	CPD50036	2.14833162	445.123887	CPD8997	8.8295419	338.19274
CPD29073	9.49556658	1094.29051	CPD50037	2.17122214	126.166178	CPD8998	8.9445122	503.17522
CPD29075	9.49688606	439.220622	CPD50039	2.16189999	247.105065	CPD9001	8.940642	448.10515
CPD2908	15.867464	271.10846	CPD5004	33.137055	473.82659	CPD9004	9.1496091	404.20027
CPD29082	9.57589587	500.10918	CPD50040	2.15802047	189.130058	CPD9019	9.2948582	206.05859
CPD29083	9.57552333	508.096174	CPD50041	2.16474941	453.119806	CPD9021	9.4601617	785.27185
CPD29084	9.58884642	962.267362	CPD50042	2.16279996	513.686145	CPD9026	9.4412059	434.17012
CPD29087	9.59391451	611.625017	CPD50043	2.15980003	505.1961	CPD903	1.06249	295.0584
CPD29089	9.60111403	603.138353	CPD50044	2.1614998	398.11971	CPD9035	9.5498936	182.12954
CPD2909	15.85921	510.2801	CPD50045	2.16005004	505.69728	CPD9037	9.7106587	722.20927
CPD29092	9.60144274	595.154222	CPD50046	2.16273492	433.116242	CPD9040	9.6988028	868.26342
CPD29093	9.60099224	1168.32535	CPD50047	2.16405253	397.791711	CPD9043	10.015182	458.21452
CPD29094	9.61298983	480.12168	CPD50049	2.16658829	265.277229	CPD9044	9.8804655	776.32226
CPD2910	15.843833	326.24649	CPD5005	33.287135	268.91255	CPD905	1.076243	410.0908
CPD29100	9.63506857	634.278479	CPD50050	2.16260001	513.184405	CPD9050	10.065602	659.25658
CPD29103	9.66645062	194.052756	CPD50053	2.16420011	144.04128	CPD9053	10.168997	1514.4271
CPD29105	9.6901428	388.111929	CPD50055	2.16455002	84.02085	CPD9055	10.174337	584.15057
CPD29109	9.67993743	372.138859	CPD50056	2.16440002	96.020705	CPD9058	10.241925	304.08275
CPD2911	15.821114	502.20175	CPD50057	2.16590007	520.173155	CPD9059	10.202755	215.15329
CPD29110	9.64196633	367.183467	CPD50058	2.16263178	513.382421	CPD906	1.069594	187.9046
CPD29111	9.67464297	126.030925	CPD50059	2.1677223	521.370056	CPD9060	10.258603	396.15363
CPD29112	9.40232716	248.052917	CPD5006	33.130902	447.83071	CPD9064	10.354198	917.29321
CPD29115	9.72948786	932.250631	CPD50060	2.16657807	260.032268	CPD9065	10.352254	469.10711
CPD29116	9.68405662	314.135935	CPD50061	2.12948701	350.080322	CPD9066	9.9311605	614.15983
CPD29117	9.68152634	332.145993	CPD50062	2.16283315	519.177983	CPD9069	11.03149	720.2269
CPD2912	15.803157	517.31625	CPD50063	2.16904999	409.15765	CPD907	1.099483	59.07354
CPD29122	9.71524996	827.678838	CPD50065	2.1654714	336.151868	CPD9071	11.18144	681.14887
CPD29124	9.71330946	819.192846	CPD50066	2.16669992	521.17085	CPD9075	11.745249	619.25267
CPD29131	9.73246629	411.096506	CPD50067	2.16399996	68.02602	CPD9078	11.867625	156.06944
CPD29133	9.7010162	800.21608	CPD50068	2.15718148	362.166164	CPD9079	11.852796	129.05735
CPD29134	9.72783611	427.067921	CPD50069	2.16665006	126.03044	CPD908	1.060794	260.9213
CPD29135	9.73086823	800.457408	CPD50071	2.16580003	521.672095	CPD9081	11.86869	162.98249
CPD29136	9.72779705	419.082035	CPD50072	2.18346069	395.1204	CPD9082	11.869305	138.03549
CPD29139	9.69043199	494.174692	CPD50073	2.17086663	516.389567	CPD9083	11.539126	235.19191

CPD29142	9.74445616	496.158985	CPD50074	2.16068446	389.8037	CPD9088	12.316998	290.07523
CPD29145	9.79200229	472.194071	CPD50077	2.16983331	162.1639	CPD909	1.056611	374.9045
CPD2915	15.931419	166.09907	CPD50079	2.1712001	325.265987	CPD9095	12.649782	186.07954
CPD29150	9.81659654	596.61958	CPD5008	33.134761	373.37409	CPD9099	12.636823	260.10245
CPD29151	9.80866541	1138.60243	CPD50080	2.16876462	521.871765	CPD9102	12.888542	646.22698
CPD29152	9.81209462	1138.31596	CPD50081	2.14395314	162.206164	CPD9105	13.59433	351.12897
CPD29153	9.81534785	852.268825	CPD50082	2.16661547	500.216969	CPD9106	13.590184	665.36232
CPD29154	9.8113335	478.170192	CPD50085	2.19047329	456.174732	CPD9108	13.582616	670.31632
CPD29157	9.81744993	588.63345	CPD50086	2.1657367	765.272111	CPD911	1.07297	173.8862
CPD29158	9.81519444	596.11855	CPD50087	2.1686001	70.04208	CPD9111	13.590903	270.17724
CPD29159	9.82354894	580.644867	CPD50088	2.17266492	114.03114	CPD9113	13.699681	290.18841
CPD2916	15.930837	220.10792	CPD50089	2.14968363	554.174837	CPD9116	13.741739	670.31577
CPD29160	9.81644326	580.14804	CPD5009	33.131763	427.83924	CPD9118	13.84643	665.36311
CPD29165	9.83299785	252.1333	CPD50090	2.1629162	704.691523	CPD912	1.066156	295.2101
CPD29168	9.85429941	770.206265	CPD50091	2.17056909	554.682979	CPD9120	13.779998	324.2278
CPD29169	9.92695748	418.18161	CPD50096	2.15449133	581.64106	CPD9122	14.624559	381.25073
CPD29170	9.88454769	573.12722	CPD50097	2.16707951	593.139527	CPD9125	14.323371	334.24969
CPD29171	9.91338524	521.28186	CPD50099	2.18210057	437.13551	CPD9126	14.325646	594.27935
CPD29172	9.87521988	581.1244	CPD5010	33.140554	213.91053	CPD9127	14.341865	576.26536
CPD29173	9.88340099	1108.30456	CPD50107	2.22483681	527.18985	CPD9129	14.590676	222.09605
CPD29174	9.88080744	581.62	CPD50108	2.12332751	339.117664	CPD913	1.128507	448.0549
CPD29175	9.88958135	573.628906	CPD5011	33.154014	400.86467	CPD9135	14.8933	254.12725
CPD29176	9.90473047	740.195674	CPD50112	2.21364707	291.193512	CPD9136	14.893818	545.33074
CPD29178	9.96496834	826.264827	CPD5012	33.141813	221.90506	CPD9139	14.894124	505.27833
CPD29184	9.94585629	576.193329	CPD50121	2.23276963	536.698423	CPD914	1.066714	296.8022
CPD29185	9.95964336	558.177958	CPD50123	2.24807929	360.25098	CPD9140	14.894932	324.38831
CPD29187	9.95489607	796.254315	CPD50124	2.23899518	449.151233	CPD9144	14.91166	578.27624
CPD29188	9.95311629	1078.29544	CPD50125	2.17389655	645.104078	CPD9145	14.920142	608.78679
CPD2919	15.940684	260.18665	CPD50128	2.23151477	666.173356	CPD9146	14.923856	608.28516
CPD29191	9.96841467	575.218417	CPD50129	2.25170167	413.15385	CPD9147	14.935827	600.79989
CPD29193	9.99946076	321.193485	CPD5013	33.142667	283.1755	CPD9148	14.901743	1374.7135
CPD29199	10.0561102	534.139461	CPD50130	2.25633039	266.078187	CPD9149	14.940905	514.31343
CPD2920	15.931715	159.06306	CPD50133	2.24961581	421.141206	CPD915	1.119593	107.015
CPD29201	10.1027452	477.252088	CPD50134	2.25380483	472.169015	CPD9152	14.931334	698.57606
CPD29202	10.1180565	418.184312	CPD50135	2.26112447	258.089287	CPD9153	14.909826	695.8422
CPD29205	10.1914621	458.214796	CPD50138	2.11246229	634.196771	CPD9154	15.097714	695.37182
CPD29208	10.1849939	245.126325	CPD5014	33.141923	269.91284	CPD9156	14.961959	703.82865
CPD2921	15.990836	317.29154	CPD50140	2.26747384	301.024306	CPD9157	14.944685	260.2134
CPD29211	10.2866218	330.131405	CPD50141	1.9156772	461.107357	CPD9159	14.904602	703.32694
CPD29212	10.278418	472.227425	CPD50143	2.26380162	229.06928	CPD9160	14.915244	352.42423
CPD29217	10.3503957	768.29678	CPD50144	2.28543792	155.049669	CPD9161	14.935774	760.31795
CPD29218	10.4007494	946.275508	CPD50146	2.23255546	342.031425	CPD9163	14.916152	349.17064
CPD29219	10.401284	916.26579	CPD50148	2.26995456	155.1596	CPD9168	15.093523	315.27691
CPD2922	15.937691	289.2034	CPD50153	2.3122568	315.0793	CPD9171	14.898183	560.26477
CPD29225	10.513498	432.1069	CPD5016	33.132364	358.87034	CPD9172	14.957506	578.27641
CPD29226	10.4817816	800.2174	CPD50160	2.33610684	321.204283	CPD9173	14.914791	632.19661
CPD29229	10.5279773	1138.31594	CPD50162	2.37785044	115.062486	CPD9177	15.047139	232.1808
CPD2923	16.033464	648.40082	CPD50166	2.37569939	447.11461	CPD9178	15.092251	596.2967
CPD29233	10.4570306	328.150927	CPD50170	2.29149644	142.045485	CPD9179	15.10087	262.11569
CPD2924	16.012995	168.08732	CPD50174	2.48053716	324.171669	CPD9182	15.069072	369.14205
CPD29243	10.5811251	381.370112	CPD50175	2.47493768	131.057131	CPD9186	15.150784	578.28248
CPD29254	10.5963846	381.199703	CPD50179	2.49924981	412.18418	CPD9187	14.957707	766.31102
CPD29256	10.5935087	386.154608	CPD5018	33.143348	273.95069	CPD9188	15.157663	496.30254
CPD29258	10.588125	391.132575	CPD50180	2.52448042	182.041383	CPD919	1.079659	140.9055
CPD2926	16.013705	414.20571	CPD50182	2.54794957	291.194395	CPD9190	15.193743	703.32811
CPD29260	10.6244706	346.162506	CPD50185	2.59035	296.05067	CPD9191	15.193013	695.84236
CPD29261	10.5868895	56.0625444	CPD50187	2.64487323	426.188479	CPD9192	15.205332	698.57496
CPD29263	10.5907441	402.1283	CPD5020	33.207283	244.90883	CPD9193	15.100418	658.35493
CPD29264	10.5893336	144.040889	CPD50200	2.85419796	249.118707	CPD9194	15.135565	750.29708
CPD29268	10.5979043	201.059941	CPD50210	3.05976742	321.203854	CPD9195	15.233444	676.3645
CPD2927	16.018422	296.27969	CPD50223	3.69507095	321.204271	CPD9196	15.211787	260.21307
CPD29271	10.6008892	319.983	CPD50226	3.9164998	249.120365	CPD9197	15.208556	349.17111
CPD29278	10.6990047	289.094114	CPD50228	4.05727102	292.152871	CPD9198	15.185903	334.24956
CPD29279	10.7020832	516.126611	CPD50229	4.08842861	292.302643	CPD9199	15.137735	687.35571
CPD2928	15.987005	238.15367	CPD50234	4.50568198	326.137138	CPD920	1.073842	226.9418
CPD29286	10.7169396	824.237063	CPD50235	4.58353146	391.140369	CPD9200	15.218084	514.31396
CPD2929	16.036205	287.26172	CPD5024	33.145048	310.93582	CPD9202	15.208427	703.82948

CPD29291	10.7705739	257.110883	CPD50247	4.6410689	533.727907	CPD9203	15.18986	760.3177
CPD29293	10.7638339	256.121667	CPD50250	4.61380564	468.220987	CPD921	1.06525	208.9515
CPD29296	10.8790049	432.198743	CPD50251	4.5908043	356.048486	CPD9214	15.582798	640.25259
CPD29297	10.9238566	354.1316	CPD50252	4.64980495	260.030518	CPD9223	15.756687	660.37551
CPD29298	10.9145281	336.119967	CPD50253	4.61754396	178.03389	CPD9225	15.756174	358.16792
CPD29300	10.963286	479.236586	CPD50255	4.64433323	283.25068	CPD9226	15.84688	632.40699
CPD29306	11.2608863	1030.29287	CPD50257	4.63699988	162.053245	CPD9227	15.772057	695.40793
CPD29308	11.175639	1368.39422	CPD50258	4.65794227	345.07326	CPD9229	15.83882	700.36309
CPD29309	10.992555	408.177267	CPD50259	4.64317658	533.226382	CPD9231	15.769851	292.2
CPD2931	16.041268	213.11847	CPD50260	4.63634076	170.047247	CPD9232	15.795332	252.2112
CPD29317	11.5679453	474.152664	CPD50261	4.6262088	403.167156	CPD9234	15.857352	234.19548
CPD29318	11.6259984	534.137685	CPD50262	4.63764993	261.12089	CPD9235	15.847565	263.12373
CPD29320	11.7254106	342.167788	CPD50263	4.6389475	144.041258	CPD9243	15.915602	198.13084
CPD29321	11.7201172	108.020422	CPD50264	4.63394684	322.173095	CPD925	1.074537	242.1753
CPD29324	11.7936049	503.270292	CPD50265	4.66144124	361.045555	CPD9253	16.040417	695.40807
CPD29325	11.7777705	508.2287	CPD50266	4.64526495	549.703569	CPD9256	16.027773	510.27906
CPD29328	11.9613288	308.008156	CPD50267	4.6461547	419.142947	CPD926	1.055512	207.967
CPD29330	12.0053059	555.221085	CPD50268	4.63610151	96.02082	CPD9260	16.103465	365.2563
CPD29333	12.1617991	360.084847	CPD5027	33.149973	317.87256	CPD9262	16.267601	690.27043
CPD29334	12.157245	256.120744	CPD50271	4.64027318	423.172918	CPD9264	16.230647	538.28082
CPD29335	12.2129975	571.250473	CPD50273	4.63810525	126.031032	CPD9265	16.275483	556.29017
CPD29336	12.2299443	606.158622	CPD50274	4.62929789	315.040444	CPD9268	16.216501	464.18128
CPD29337	12.3185985	302.07883	CPD50275	4.64259695	84.02091	CPD9271	16.288368	676.36319
CPD29338	12.3145557	219.0253	CPD50276	4.64752626	549.201805	CPD9273	16.299625	346.1655
CPD29340	12.3207508	211.039572	CPD50277	4.64981272	541.215169	CPD9274	16.424163	354.15256
CPD29342	12.369898	330.073645	CPD50278	4.63827958	411.156144	CPD9276	16.369331	636.37191
CPD29343	12.3894269	456.199686	CPD50279	4.63999987	576.160478	CPD9277	16.358224	312.26506
CPD29344	12.387058	300.063324	CPD5028	33.130276	374.86946	CPD9284	16.610757	600.25593
CPD29345	12.2309661	164.118514	CPD50280	4.64617607	674.135006	CPD9285	16.382337	556.29213
CPD2935	16.119616	477.2852	CPD50281	4.65896838	136.025827	CPD9286	16.491932	299.28112
CPD29353	12.4853333	196.144867	CPD50282	4.66785851	329.094093	CPD9288	16.502193	335.28088
CPD29362	12.6550807	706.248436	CPD50283	4.65246863	606.192827	CPD9290	16.490097	334.24837
CPD29368	12.6767984	196.145514	CPD50286	4.6332355	68.0250333	CPD9291	16.631381	284.1177
CPD2937	16.146083	194.09491	CPD50287	4.71497935	368.050976	CPD9292	16.492588	514.31432
CPD29373	12.8313886	467.292778	CPD5029	33.14075	217.90359	CPD9297	16.66752	676.36385
CPD29376	12.825296	832.724365	CPD50290	4.71630033	352.07641	CPD9298	16.665569	346.16534
CPD29377	12.8337475	194.17161	CPD50291	4.75463982	168.041795	CPD930	1.111028	308.0537
CPD2938	16.226816	648.40063	CPD50294	4.80325037	682.170092	CPD9300	16.647387	312.26453
CPD29385	13.0100691	676.237143	CPD5030	33.152416	489.8025	CPD9301	16.675171	354.15108
CPD29386	13.0287324	249.17612	CPD50300	5.18449206	264.146737	CPD9303	16.671973	671.40674
CPD29389	13.1467144	208.145771	CPD50301	5.23336753	408.118789	CPD9304	16.592099	738.3341
CPD29391	13.2533729	420.10665	CPD50308	5.3975898	977.2165	CPD9305	16.687965	560.24434
CPD29393	13.2841009	270.13866	CPD50309	5.40739757	788.19973	CPD9306	16.785799	648.40058
CPD29394	13.338916	674.222962	CPD5031	33.144192	418.85231	CPD9310	16.592208	1050.6073
CPD29397	13.3629276	319.213171	CPD50310	5.40752423	486.1151	CPD9311	16.649614	576.23429
CPD2940	16.194984	414.20481	CPD50312	5.40793483	502.587422	CPD9312	16.72895	531.54111
CPD29400	13.5381022	252.171826	CPD50314	5.40784837	950.25209	CPD9313	16.61911	268.1426
CPD29401	13.5438354	204.192362	CPD50315	5.40573351	626.147732	CPD9314	16.631412	352.42459
CPD29407	13.5685539	359.245467	CPD50317	5.40894485	494.100505	CPD9315	16.673806	598.26602
CPD29408	13.5934003	718.203053	CPD50318	5.40688963	522.599306	CPD9316	16.729363	514.31406
CPD29412	13.637599	690.25617	CPD50319	5.40804517	502.086847	CPD9317	16.739799	533.31554
CPD29414	13.6974107	650.222606	CPD5032	33.154076	252.22935	CPD9318	16.757274	334.24914
CPD29418	13.7430715	618.231893	CPD50320	5.41035251	523.101636	CPD932	1.069836	411.9526
CPD2942	16.306649	301.28379	CPD50323	5.42324674	306.168187	CPD9320	16.670546	533.78963
CPD29420	13.8086061	569.236525	CPD50326	5.5385495	454.2053	CPD9321	16.655446	260.2133
CPD29426	13.9431572	344.089637	CPD50327	5.53009784	375.097685	CPD9322	16.786814	642.26841
CPD29428	13.9572778	359.245617	CPD50329	5.60382941	469.413258	CPD9323	16.771057	404.25574
CPD29430	13.966999	368.164683	CPD5033	33.145117	319.91739	CPD9326	16.944271	276.21048
CPD29431	14.0973881	658.229673	CPD50330	5.60364821	468.221915	CPD9327	16.791695	284.11751
CPD29433	14.0159989	335.245282	CPD50333	5.5891433	469.5189	CPD9328	16.957592	328.26495
CPD29435	14.0386351	234.161955	CPD50336	5.63999857	178.030636	CPD9333	17.396145	422.26653
CPD29443	14.1933989	263.188715	CPD50338	5.66219695	170.04743	CPD9334	17.327366	462.25908
CPD29445	14.2011106	658.227811	CPD5034	33.177438	301.90428	CPD9335	17.259208	321.26594
CPD29447	14.2168415	195.0531	CPD50340	5.71128246	301.885889	CPD934	1.070161	464.0336
CPD29448	14.2086493	286.08405	CPD50341	5.70977853	402.180917	CPD9341	17.438809	462.2597
CPD2945	16.350084	477.28507	CPD50342	5.71224389	297.132806	CPD9343	17.467608	239.1145
CPD29451	14.2109957	370.037473	CPD50343	5.72116657	276.158228	CPD9344	17.599803	422.26812

CPD29453	14.2133505	211.02792	CPD50348	5.83935351	292.153565	CPD9346	17.691425	538.31154
CPD29454	14.2133004	203.041595	CPD5035	33.1335	423.85862	CPD9356	17.813397	189.07493
CPD29455	14.2330012	202.170567	CPD50351	5.84607754	290.138893	CPD9357	17.836869	346.21215
CPD29459	14.2719993	382.040214	CPD50352	5.84472695	597.266318	CPD9359	17.762543	326.06961
CPD29460	14.3111332	224.055456	CPD50354	5.9648389	385.193579	CPD9360	17.525056	239.11725
CPD29461	14.3083996	344.08946	CPD50356	6.00992807	802.215164	CPD9361	17.776149	422.26798
CPD29462	14.3132503	240.03124	CPD50358	6.01453768	176.046577	CPD9363	17.71327	277.13982
CPD29463	14.3379999	826.228983	CPD5036	33.1426	619.76483	CPD9364	18.029157	666.41076
CPD2947	16.432032	333.20769	CPD50360	6.01093626	132.073906	CPD9368	17.917039	181.08941
CPD29470	14.3616387	758.240214	CPD50362	6.00894053	265.293324	CPD9369	17.649414	324.2298
CPD29472	14.3693634	706.248018	CPD50364	6.02225329	597.267175	CPD9370	17.391347	239.11327
CPD29477	14.4818997	300.06334	CPD50367	6.04279424	336.178695	CPD9379	18.304669	514.32146
CPD2948	16.435862	317.23554	CPD50369	6.10450029	788.19865	CPD938	1.060634	307.9367
CPD29480	14.5416995	287.189	CPD5037	33.149716	132.12342	CPD9380	18.46026	294.2178
CPD29482	14.5590615	341.233727	CPD50371	6.1501585	306.873025	CPD9383	18.320835	648.40048
CPD29485	14.6079468	374.099853	CPD5038	33.151298	322.92042	CPD9385	18.483072	608.26345
CPD29487	14.7008495	316.09664	CPD50380	6.14653185	648.3136	CPD9386	18.537751	323.27987
CPD2949	16.434479	169.08868	CPD50381	6.14845294	153.08378	CPD939	1.080152	218.0193
CPD29491	14.6153163	381.228742	CPD50383	6.15076276	306.832111	CPD9390	18.641001	265.14128
CPD29492	14.7264263	704.271671	CPD50386	6.15208238	675.326618	CPD9392	18.644689	514.31559
CPD29496	14.7552198	720.266267	CPD50389	6.15360099	390.122215	CPD9393	18.633669	326.22588
CPD29497	14.8731813	524.331209	CPD5039	33.132038	388.88538	CPD9398	18.846927	1101.5681
CPD29499	14.9059821	334.25005	CPD50395	6.26972262	583.102713	CPD9399	18.855347	1468.7545
CPD2950	16.439121	277.23982	CPD50396	6.26623892	950.231508	CPD9400	18.923792	334.21628
CPD29503	14.8655392	306.197937	CPD50397	6.26250237	788.181243	CPD9404	19.04515	352.26022
CPD29505	14.9088888	1352.54429	CPD50399	6.26593602	1112.28375	CPD9405	19.080522	662.36739
CPD29506	14.9182883	1349.52299	CPD5040	33.141084	315.91291	CPD9406	19.116325	334.22995
CPD29507	14.9425257	514.196237	CPD50401	6.27621248	312.085086	CPD9408	19.097062	704.30641
CPD29508	14.9386473	514.697635	CPD50403	6.30538653	874.236892	CPD9409	19.118732	297.26583
CPD29509	14.9579999	681.072766	CPD50404	6.29828035	712.1836	CPD941	1.085058	248.0333
CPD2951	16.364854	166.06051	CPD50406	6.31968513	626.14195	CPD9411	19.121211	814.49976
CPD29510	14.9398574	1368.52267	CPD50407	6.32808787	218.1149	CPD9413	19.124833	708.27696
CPD29511	14.9415721	1372.52784	CPD50408	6.36599763	336.179245	CPD9417	19.287743	640.25418
CPD29517	14.9617995	314.07917	CPD5041	33.15164	228.93138	CPD942	1.060788	327.8941
CPD29518	14.9627694	352.027146	CPD50410	6.40719785	445.17036	CPD9420	19.354788	656.40184
CPD2952	16.462509	244.1032	CPD50415	6.51322603	138.078262	CPD9421	19.3512	312.11866
CPD29523	15.0094648	664.057417	CPD50416	6.51448963	320.1843	CPD9422	19.347942	232.18085
CPD29524	14.9699996	217.039035	CPD50418	6.51639342	276.158345	CPD9427	19.42676	654.37495
CPD29527	14.931573	677.485329	CPD50419	6.5144424	277.307978	CPD943	1.086927	184.0256
CPD29529	14.9720498	225.02513	CPD5042	33.154	300.91063	CPD9430	19.616548	371.73669
CPD2953	16.568404	598.26645	CPD50420	6.56960099	1274.33661	CPD9434	19.581309	371.24016
CPD29534	15.0083587	374.099559	CPD50422	6.57917483	626.146306	CPD9435	19.648215	390.22604
CPD29535	14.9586826	1033.51518	CPD50423	6.58581279	788.181318	CPD944	1.084	362.8759
CPD29536	15.0200591	236.176435	CPD50424	6.58767931	583.604638	CPD9440	19.732079	224.17789
CPD29539	15.0838751	341.2346	CPD50426	6.58742251	575.116226	CPD9444	19.860636	371.73702
CPD2954	16.541722	276.13045	CPD50427	6.58579088	1112.28359	CPD9445	19.8802	582.31607
CPD29540	15.1099472	270.137011	CPD50428	6.58636728	950.232021	CPD9452	19.84661	187.11485
CPD29541	15.1241997	404.110515	CPD50429	6.57967097	664.129978	CPD9457	19.998504	640.28972
CPD29549	15.1381791	1033.51982	CPD50430	6.58794246	583.102865	CPD9459	20.050256	365.32577
CPD2955	16.576504	536.29546	CPD50433	6.66775163	152.049425	CPD946	1.084935	182.043
CPD29550	15.1969989	737.418807	CPD50434	6.69112443	674.18108	CPD9466	20.151689	390.09954
CPD29551	15.1904284	687.851414	CPD50436	6.7458535	336.178993	CPD9468	20.036462	686.29201
CPD29555	15.1917304	374.10034	CPD50438	6.75949923	799.686767	CPD9469	20.267691	674.26613
CPD29558	15.2667977	704.283227	CPD50439	6.85835115	799.195427	CPD947	1.0769	529.7978
CPD29559	15.299266	277.20702	CPD5044	33.133076	380.87663	CPD9472	20.328743	280.2399
CPD2956	16.516386	531.34002	CPD50441	6.76676744	807.174771	CPD9477	20.541533	580.39102
CPD29561	15.2989326	462.35444	CPD50442	6.77083526	413.074311	CPD948	1.091003	235.9947
CPD29562	15.3615263	444.343806	CPD50444	6.78256417	332.048264	CPD9483	20.458852	567.36999
CPD29566	15.5270499	289.203585	CPD50445	6.76462936	807.677044	CPD9484	20.352393	280.24011
CPD29567	15.5227143	289.352471	CPD50451	6.76407028	815.663838	CPD9488	20.532539	252.20883
CPD29568	15.5664001	373.261387	CPD50452	6.77555104	626.14782	CPD949	1.086614	138.9073
CPD29569	15.588357	725.388779	CPD50453	6.77619941	788.20015	CPD9490	20.500754	654.27191
CPD2957	16.526341	352.26106	CPD50454	6.77585086	464.095105	CPD9496	20.89655	394.25009
CPD29570	15.6235555	537.205978	CPD50455	6.75287134	464.2812	CPD9497	20.775886	578.2528
CPD29572	15.7609351	393.227987	CPD50456	6.77294019	421.06007	CPD9505	20.96055	660.25535
CPD29573	15.6922497	415.329283	CPD50459	6.76770354	815.161971	CPD9507	20.919496	391.34289
CPD29577	15.7540048	287.250593	CPD5046	33.203352	95.073473	CPD9510	21.039235	670.30159

CPD29578	15.802178	297.266154	CPD50465	6.76799692	340.033622	CPD9513	20.622519	432.28179
CPD29579	15.7629992	646.297555	CPD50467	6.78546574	872.153231	CPD9516	21.086688	692.22634
CPD2958	16.576969	284.11781	CPD50469	6.8156668	591.128311	CPD9517	21.068109	676.24964
CPD29580	15.8864598	521.275583	CPD50470	6.88271605	307.009675	CPD9518	21.043719	852.54117
CPD29585	15.8593838	572.271977	CPD50475	6.85347604	289.142467	CPD9519	21.082481	606.24783
CPD29586	15.8553994	393.22812	CPD50478	6.85364241	153.08403	CPD952	1.049906	173.0168
CPD2959	16.528481	496.30298	CPD5048	33.149733	183.01984	CPD9520	21.071219	713.31913
CPD29590	15.9844609	471.671438	CPD50480	6.863305	306.870889	CPD9523	21.098501	300.35884
CPD29595	16.0230997	335.245805	CPD50482	6.85265233	130.120433	CPD9524	21.100181	260.21374
CPD29596	15.9622895	297.266027	CPD50485	6.85114298	802.194329	CPD9525	21.191552	332.1425
CPD29599	16.0884656	462.354517	CPD50488	6.85999993	335.1211	CPD9526	21.129499	340.14789
CPD29601	16.0865458	420.248055	CPD5049	33.151955	372.36905	CPD9531	21.063727	692.23729
CPD29605	16.117516	395.243162	CPD50491	6.85799792	322.163453	CPD9534	21.557818	800.46923
CPD29606	16.1276362	355.252791	CPD50496	6.93041664	227.021488	CPD9538	21.393366	659.4252
CPD2961	16.610745	292.20412	CPD50501	6.93431856	416.094905	CPD9539	21.446349	612.25949
CPD29610	16.1636245	409.218812	CPD50503	6.94310579	168.042669	CPD9541	21.473904	279.81323
CPD29611	16.1969926	315.219517	CPD50504	6.94652092	1025.66169	CPD9549	21.445048	798.45289
CPD29614	16.2517367	359.245868	CPD50505	6.94184206	1025.0635	CPD955	1.083967	103.1
CPD29616	16.1986954	353.234087	CPD50508	6.96617548	854.718	CPD9550	21.769834	376.31803
CPD2962	16.610366	314.11483	CPD50509	6.97303023	854.884443	CPD9553	21.579029	754.50317
CPD29622	16.3661001	291.21957	CPD5051	33.14845	141.99361	CPD9563	21.7485	998.63438
CPD29626	16.4214298	957.343257	CPD50510	6.9546391	855.050346	CPD9566	21.878476	488.29769
CPD29629	16.4966313	572.262537	CPD50511	6.9514849	1025.46161	CPD9569	21.883596	582.40756
CPD2963	16.634236	301.27977	CPD50514	6.94806923	732.330064	CPD957	1.083784	454.1088
CPD29633	16.5390915	539.232773	CPD50515	6.94792443	1025.86159	CPD9573	22.078477	367.34422
CPD29634	16.567055	411.218222	CPD50518	6.9573366	854.5512	CPD9575	21.982025	410.16549
CPD29635	16.5652999	355.251475	CPD50519	6.98715182	772.185092	CPD9576	22.22996	574.4923
CPD29636	16.5658501	395.243205	CPD5052	33.143294	311.92871	CPD9579	22.085632	578.25334
CPD2964	16.623499	336.09726	CPD50520	6.95773468	854.2193	CPD9580	22.119197	658.30033
CPD29640	16.5754502	464.176	CPD50521	6.96217053	854.385106	CPD9581	22.129918	383.18632
CPD29647	16.7558003	710.25712	CPD50524	6.9966101	435.214867	CPD9582	22.231965	384.21741
CPD29648	16.839027	508.33645	CPD50526	6.94400933	732.189417	CPD9584	22.242721	646.91871
CPD29649	16.7715332	688.27558	CPD50528	7.02576024	850.715377	CPD9589	22.225786	902.50006
CPD2965	16.798366	269.23371	CPD5053	33.150068	128.97227	CPD9592	22.234545	297.26537
CPD29653	16.8418365	358.105375	CPD50531	7.03854651	758.191444	CPD9596	22.227696	789.33642
CPD29655	16.9426432	395.243394	CPD50532	7.03764806	336.179335	CPD9598	22.298461	324.22385
CPD29656	16.8712774	301.20775	CPD50535	7.07889074	802.193611	CPD960	1.090722	269.195
CPD29657	16.8746999	300.10085	CPD50536	7.09208736	964.246858	CPD9600	22.55938	622.28047
CPD29659	16.8703088	347.245345	CPD50537	7.08398377	848.8794	CPD9602	22.38646	636.25943
CPD2966	16.684977	616.41097	CPD50539	7.11175559	1126.29816	CPD9605	22.513906	355.34176
CPD29663	16.9221428	957.3416	CPD50540	7.0729988	848.382033	CPD9609	22.517049	378.26297
CPD29664	16.9439997	704.303467	CPD50544	7.08700363	1183.2762	CPD961	1.099357	380.0729
CPD29665	16.9740499	501.402185	CPD50546	7.08648305	1175.28862	CPD9612	22.349013	599.61881
CPD29669	17.0232502	427.217875	CPD50548	7.05112703	790.694017	CPD9619	22.792068	1007.6917
CPD29670	17.0455001	438.34875	CPD50550	7.10099855	626.146994	CPD9628	22.852254	495.80831
CPD29672	17.052521	349.259292	CPD50552	7.09776393	605.617788	CPD963	1.088943	239.8589
CPD29674	17.0396	514.36272	CPD50554	7.10089638	1183.7722	CPD9630	22.8194	926.42836
CPD29675	17.0284416	355.250995	CPD50555	7.03982132	1156.3103	CPD9631	22.836774	636.2337
CPD29676	16.9969873	395.243363	CPD50556	7.10385552	994.257905	CPD9635	22.878431	704.21898
CPD29678	17.091375	285.229475	CPD5056	33.163594	166.00563	CPD964	1.094846	454.0052
CPD29679	17.175618	305.236025	CPD50560	7.10464144	605.11663	CPD9641	22.851773	512.86198
CPD29681	17.1266	365.29113	CPD50562	7.09486421	597.630916	CPD9646	22.998503	664.25023
CPD29683	17.0894586	371.2459	CPD50563	7.09657118	1175.78644	CPD9649	23.080732	558.50174
CPD2969	16.692605	342.22091	CPD50566	7.10311915	524.091065	CPD965	1.599196	169.0499
CPD29692	17.166625	721.3209	CPD50568	7.10000049	597.129994	CPD9650	23.116668	620.47752
CPD29695	17.1648183	342.398945	CPD5057	33.150468	124.97897	CPD9656	23.145744	563.21475
CPD29696	17.1675998	341.235135	CPD50571	7.10613085	589.639887	CPD9658	23.165635	281.8282
CPD29698	17.1623634	363.383736	CPD50575	7.12733166	896.395278	CPD966	1.118946	187.0149
CPD2970	16.721774	632.40647	CPD50577	7.11275246	1229.31336	CPD9660	23.166858	643.47488
CPD29701	17.1558617	358.10503	CPD50578	7.11773377	1536.64089	CPD9661	23.225601	282.02725
CPD29702	17.0271319	239.052868	CPD50579	7.10912403	1537.13974	CPD9667	23.154403	866.5811
CPD29703	17.1891436	652.253443	CPD5058	33.149983	83.952918	CPD967	1.094046	349.0819
CPD29705	17.2907146	346.220043	CPD50581	7.11356371	1024.59633	CPD9678	23.314471	592.26809
CPD29706	17.242503	428.349044	CPD50582	7.11268199	1229.71228	CPD968	1.091564	213.0523
CPD29708	17.3108591	317.235233	CPD50585	7.10820933	1024.43187	CPD9683	23.348334	564.23706
CPD2971	16.763477	408.26546	CPD50586	7.11483385	1229.51186	CPD9684	23.35375	614.25113
CPD29710	17.3170498	457.301225	CPD50588	7.11111129	1229.11252	CPD9685	23.368831	300.19991

CPD29713	17.2050185	726.283471	CPD50589	7.12979814	832.206915	CPD9687	23.403164	631.91301
CPD29714	17.3298831	478.234967	CPD5059	33.140328	192.96777	CPD9692	23.589589	549.86319
CPD29715	17.2467138	375.277571	CPD50590	7.13071353	1075.27495	CPD9693	23.511999	690.47925
CPD29717	17.2535002	329.23477	CPD50591	7.12470437	841.198436	CPD9700	23.580895	587.88651
CPD29718	17.2628486	697.321068	CPD50592	7.12039427	1229.91254	CPD9703	23.622557	300.26445
CPD29719	17.2717741	361.259867	CPD50593	7.10845925	1024.25738	CPD9706	23.724048	511.26587
CPD2972	16.744105	599.35789	CPD50594	7.12036166	1536.89123	CPD9708	23.668228	740.44947
CPD29721	17.2793691	359.244516	CPD50595	7.12805141	895.895576	CPD971	1.090911	185.0125
CPD29724	17.2795625	470.681088	CPD50596	7.09955443	1018.05627	CPD9717	23.833511	636.26006
CPD29726	17.3061117	278.223722	CPD50597	7.12689569	1075.47552	CPD9719	23.828149	534.26406
CPD29727	17.3287772	409.315089	CPD50598	7.12798812	1075.0763	CPD972	1.099211	188.9857
CPD29729	17.1941444	393.228436	CPD50599	7.14805359	1261.04137	CPD9720	23.81471	519.87085
CPD2973	16.321119	671.4042	CPD5060	33.168583	176.95984	CPD9722	24.07934	396.28422
CPD29730	17.3114044	317.235308	CPD50600	7.1339919	1001.64791	CPD9727	24.272149	757.5587
CPD29731	17.3977776	644.4511	CPD50601	7.12987806	896.228811	CPD973	1.092719	352.0759
CPD29732	17.3960624	478.678669	CPD50602	7.11733482	1228.91191	CPD9733	24.200522	740.44682
CPD29734	17.3558289	305.235594	CPD50603	7.13878385	834.871306	CPD974	1.095433	427.0824
CPD29736	17.431106	385.318284	CPD50604	7.13199506	896.062294	CPD9749	24.933524	581.46201
CPD29741	17.4310913	478.235617	CPD50605	7.13060426	895.730522	CPD975	1.095971	167.0181
CPD29743	17.4889996	752.274867	CPD50606	7.14386113	840.695333	CPD976	1.093222	292.0561
CPD29744	17.5018998	501.401865	CPD50607	7.14598638	1253.05884	CPD9760	25.111164	713.24082
CPD29745	17.4954757	446.360617	CPD50608	7.1378264	1001.04925	CPD9762	25.131557	900.2141
CPD29747	17.51925	727.331745	CPD50609	7.15558794	840.36154	CPD9764	25.15227	647.24326
CPD29748	17.5181995	715.331393	CPD50610	7.10414735	1536.39131	CPD9766	25.104609	607.0944
CPD2975	16.802538	519.33167	CPD50611	7.13784144	834.373455	CPD977	1.102587	569.0577
CPD29753	17.5311993	443.35934	CPD50612	7.14489212	712.89279	CPD9775	25.290386	836.47456
CPD29755	17.5291669	388.2894	CPD50613	7.13886713	1251.56131	CPD9778	25.287136	763.52196
CPD29756	17.5355713	371.246014	CPD50614	7.14080869	1001.44733	CPD9779	25.287648	768.47801
CPD29758	17.5351499	388.115825	CPD50615	7.13381113	834.539912	CPD978	1.107504	481.0242
CPD29759	17.53935	254.05774	CPD50616	7.14111265	713.034239	CPD9780	25.29225	728.48794
CPD2976	16.66713	276.13088	CPD50617	7.14827476	1247.56168	CPD9784	25.293401	784.45207
CPD29763	17.5928535	614.237214	CPD50618	7.14460089	712.74973	CPD979	1.105375	440.9537
CPD29765	17.6228172	723.3572	CPD5062	33.158917	185.0352	CPD9791	25.523399	959.13486
CPD29767	17.6341577	515.417879	CPD50620	7.14893798	831.87473	CPD9793	25.498748	959.94631
CPD2977	16.702573	496.30319	CPD50621	7.16172512	838.365718	CPD9798	25.640673	628.26602
CPD29773	17.5771888	624.2575	CPD50622	7.14371768	1001.24883	CPD9801	25.854953	763.52342
CPD29775	17.6875628	494.665144	CPD50623	7.14079091	713.319379	CPD9802	25.966359	784.45297
CPD29779	17.7532854	731.1897	CPD50624	7.14986817	831.20996	CPD9803	25.722	768.47764
CPD2978	16.737155	648.40032	CPD50625	7.15057917	1247.81137	CPD9807	25.773368	578.25311
CPD29785	17.7945154	373.261	CPD50626	7.13943612	1000.84565	CPD981	1.093536	322.0667
CPD29788	17.8301667	379.308406	CPD50627	7.14229457	1256.79909	CPD9813	26.008859	889.51321
CPD2979	16.697775	352.2611	CPD50628	7.14968232	832.04133	CPD982	1.1065	515.1134
CPD29790	17.8295882	419.300553	CPD50630	7.17081812	464.096808	CPD9827	26.201493	585.65605
CPD29792	17.8778584	393.22965	CPD50631	7.15337377	1247.06122	CPD983	1.110182	380.2425
CPD29793	17.8730043	364.267375	CPD50632	7.14705439	998.2499	CPD984	1.106517	89.04813
CPD29794	17.86515	328.09463	CPD50633	7.16174788	1252.80774	CPD985	1.103502	259.0472
CPD29795	17.8704445	748.297911	CPD50634	7.14937629	997.85043	CPD9852	26.730294	666.44401
CPD29798	17.8681112	224.046978	CPD50635	7.14860131	998.049925	CPD9853	26.697829	702.20621
CPD29799	17.8767501	653.294995	CPD50636	7.14814273	998.44999	CPD986	1.180254	553.0804
CPD2980	16.787361	316.25794	CPD50637	7.14162092	1005.23989	CPD9865	27.46622	960.84684
CPD29800	17.8874002	325.3599	CPD50638	7.14797237	1247.3114	CPD9868	27.556128	791.5542
CPD29801	17.8896998	303.220205	CPD50639	7.14979007	831.37571	CPD9869	27.567479	796.50995
CPD29802	17.9796756	299.24545	CPD5064	33.15492	178.14206	CPD9870	27.55845	812.48409
CPD29803	17.8999361	348.276419	CPD50640	7.14907608	997.65062	CPD9873	27.702601	646.48402
CPD29806	17.9045504	362.26954	CPD50641	7.14983571	831.542035	CPD9876	27.768651	440.29238
CPD29807	17.9395006	620.44921	CPD50642	7.15017981	997.451033	CPD9885	28.198307	812.48432
CPD2981	16.723271	536.29526	CPD50643	7.14329599	834.706935	CPD9886	28.199759	796.50934
CPD29810	17.9814995	572.26265	CPD50645	7.14567617	1009.03463	CPD9887	28.204925	791.55404
CPD29814	18.1047714	423.33214	CPD50646	7.14987518	831.70822	CPD9897	28.367454	748.50907
CPD29816	18.0414988	226.188583	CPD50647	7.13966895	712.608121	CPD9899	28.370235	854.46071
CPD2982	16.77546	422.26563	CPD50648	7.14904761	713.177145	CPD990	1.151981	202.0459
CPD29822	18.1019285	341.235033	CPD5065	33.154141	133.98302	CPD992	1.125909	301.0221
CPD29824	18.1012497	600.294208	CPD50650	7.14761586	1008.83534	CPD9924	29.227693	507.44691
CPD29826	18.1646813	391.304867	CPD50651	7.1558162	1008.63406	CPD9930	29.403029	339.38543
CPD29827	18.0722839	299.246	CPD50652	7.14506166	713.461768	CPD9941	30.819438	529.63426
CPD29828	18.1475712	626.245986	CPD50653	7.15191183	837.866658	CPD9952	31.54466	591.4792
CPD2983	16.757283	531.33995	CPD50655	7.14645626	837.7	CPD997	1.126033	262.0098

CPD29830	18.1544459	399.334678	CPD50656	7.15399836	840.863458	CPD9977	32.951562	950.5961
CPD29833	18.1694001	1017.47437	CPD50657	7.16147693	1008.23638	CPD998	1.1334	364.0967
CPD29836	18.1914613	362.224854	CPD50658	7.14159741	1260.79139	CPD9985	33.098534	256.17743
CPD2984	16.77632	284.19747	CPD50659	7.19991723	1020.28849	CPD9992	33.107677	333.90028
CPD29841	18.195125	388.305925	CPD5066	33.150154	152.97215	CPD9993	33.108333	263.19997
CPD29845	18.1852501	515.417105	CPD50660	7.14574821	1009.23584	CPD9994	33.101728	270.17968
CPD29847	18.1912352	343.251071	CPD50661	7.19833122	696.18856	CPD9998	33.1185	150.11328
CPD29848	18.1941541	443.178631	CPD50663	7.13850239	1261.29334	CPD9999	33.125958	691.76131
